# The scale and evolutionary significance of horizontal gene transfer in the choanoflagellate *Monosiga brevicollis*

**DOI:** 10.1186/1471-2164-14-729

**Published:** 2013-10-25

**Authors:** Jipei Yue, Guiling Sun, Xiangyang Hu, Jinling Huang

**Affiliations:** Key Laboratory of Biodiversity and Biogeography, Kunming Institute of Botany, Chinese Academy of Sciences, Kunming, 650201 China; Key Laboratory of Economic Plants and Biotechnology, Kunming Institute of Botany, Chinese Academy of Sciences, Kunming, 650201 China; Department of Biology, East Carolina University, Greenville, NC 27858 USA

**Keywords:** Genome evolution, Choanoflagellates, HGT frequency, Eukaryotic evolution, Adaptation

## Abstract

**Background:**

It is generally agreed that horizontal gene transfer (HGT) is common in phagotrophic protists. However, the overall scale of HGT and the cumulative impact of acquired genes on the evolution of these organisms remain largely unknown.

**Results:**

Choanoflagellates are phagotrophs and the closest living relatives of animals. In this study, we performed phylogenomic analyses to investigate the scale of HGT and the evolutionary importance of horizontally acquired genes in the choanoflagellate *Monosiga brevicollis*. Our analyses identified 405 genes that are likely derived from algae and prokaryotes, accounting for approximately 4.4% of the *Monosiga* nuclear genome. Many of the horizontally acquired genes identified in *Monosiga* were probably acquired from food sources, rather than by endosymbiotic gene transfer (EGT) from obsolete endosymbionts or plastids. Of 193 genes identified in our analyses with functional information, 84 (43.5%) are involved in carbohydrate or amino acid metabolism, and 45 (23.3%) are transporters and/or involved in response to oxidative, osmotic, antibiotic, or heavy metal stresses. Some identified genes may also participate in biosynthesis of important metabolites such as vitamins C and K12, porphyrins and phospholipids.

**Conclusions:**

Our results suggest that HGT is frequent in *Monosiga brevicollis* and might have contributed substantially to its adaptation and evolution. This finding also highlights the importance of HGT in the genome and organismal evolution of phagotrophic eukaryotes.

**Electronic supplementary material:**

The online version of this article (doi:10.1186/1471-2164-14-729) contains supplementary material, which is available to authorized users.

## Background

While horizontal gene transfer (HGT) in prokaryotes has been extensively studied and its significance in prokaryotic evolution is well known, our knowledge about HGT in eukaryotes is relatively limited [1–4]. In eukaryotes, a large number of genes are of bacterial origin, many of which are derived from mitochondria or plastids through endosymbiotic gene transfer (EGT), whereas some others are from independent HGT events. A gene ratchet mechanism “you are what you eat” has been proposed to explain frequent gene transfer events in protists, especially those of phagotrophic lifestyles [5]. The list of HGT-derived genes in diverse protists becomes increasingly longer thanks to recent studies [6–9].

*Monosiga brevicollis* is a unicellular member of choanoflagellates, a group of free-living and phagotrophic microbial eukaryotes. Characterized by a central flagellum surrounded by a ring of 30–40 microvilli, choanoflagellates resemble sponge choanocytes morphologically [10]. Molecular phylogenetic analyses show that choanoflagellates form a distinct lineage that is closely related to animals [11, 12]. Because of their unique evolutionary position, choanoflagellates bear great significance in understanding the origin of animals. Genome of *M. brevicollis* has been sequenced and annotated [13], thus offering a good opportunity for comparative genomic studies to understand the evolution of choanoflagellates.

*Monosiga brevicollis* has structures to facilitate swimming and feeding. Its flagella can cause water current when in motion, which in turn propel itself to swim freely. Its microvillar collar helps hold bacteria and other detritus from water flow and then engulfs them as foodstuff. Because of their high feeding efficiency, *M. brevicollis* and other choanoflagellates play a critical ecological role in marine ecosystems, particularly related to global carbon cycle [14]. Previous studies identified over 100 algal genes in *M. brevicollis* genome, and it has been suggested that many of these genes were likely acquired from food sources and might have benefited *M. brevicollis* in food digestion and adaptation to environmental stresses [15–18]. Although these studies identified an impressive number of acquired genes in *M. brevicollis*, the major sources of these genes were all from eukaryotic groups, and those from prokaryotes were not extensively investigated.

Currently, several computational programs, including PhyloGenie [19], DarkHorse [20] and AlienG [21], are available for genome screening of horizontally acquired genes. PhyloGenie predicts acquired genes by extracting generated gene trees that match specific topological constraints [19], and it has often been used in HGT identification [16, 22–25]. DarkHorse is a similarity-based tool for rapid identification of HGT candidates at genome level. This program predicts acquired genes by re-ranking the matches in BLAST search based on their species relationships with the query [20]. This approach alleviates the over-reliance on top-scoring BLAST hits for HGT identification and has been used in several studies [16, 26, 27]. AlienG is a newly developed computational program for HGT identification [21]. Based on an assumption that sequence similarity is correlated to sequence relatedness, AlienG detects candidates of acquired genes by comparing sequence similarities of the query to distantly related organisms versus those to close relatives. This program has recently been used in detecting acquired virulence effector gene homologs in chytrids [28], algae-related genes in animals [29] and HGT-derived genes in the basal land plant *Physcomitrella patens*[30]. In this study, we performed a comprehensive analysis to identify acquired genes in *M. brevicollis* based on predictions from these three computational programs. Through this extensive study, we aim to understand the overall scope and role of HGT in the evolution of *Monosiga*.

## Results and discussion

### Genome screening for foreign genes in *M. brevicollis*

Although both PhyloGenie [19] and DarkHorse [20] have been successfully used in some studies [16, 27, 28, 31], their limitations are obvious. Because PhyloGenie samples top hits of BLAST search for phylogenetic tree construction, a large database may lead to biased taxonomic sampling when the top hits are from the same or closely related taxonomic groups. Likewise, DarkHorse only accepts the NCBI non-redundant (*nr*) database, and genomes absent from *nr* would be missed in the analysis, thus leading to a large pool of candidates with many false positives. To obtain more reliable prediction results, we created a customized database covering representative species for prediction of foreign genes using PhyloGenie. Additionally, other available eukaryotic genomes were added to the NCBI *nr* database for AlienG analyses.

Identification of HGT is always complicated by multiple issues, such as differential losses, insufficient taxonomic sampling, and phylogenetic artifacts due to data quality or long-branch attraction [23, 32–34]. For each predicted foreign gene, we performed additional manual inspection for shared indels, conserved amino acid positions, unique gene structure, alignment quality, and potential contamination [16, 31]. The possibility of potential contamination was largely eliminated by checking whether the adjacent genes on genomic scaffolds showed metazoan/fungal affiliation. We also considered phyletic distribution of the gene (e.g., distribution only in choanoflagellates, prokaryotes and/or algae) and performed further manual phylogenetic analyses. A potential HGT event was inferred if the subject choanoflagellate gene forms a monophyletic group with homologs from prokaryotes and/or algae (with 70% or higher bootstrap support), to the exclusion of sequences from fungi/metazoans. Here, the term “algae” is loosely defined to include organisms with primary, secondary or tertiary plastids. Because oomycetes and ciliates are often considered to be of photosynthetic ancestry [35], they were also deemed as algae in this study. These measures would effectively reduce the artifacts associated with the gene tree construction.

Determination of HGT direction is not always straightforward. Other than gene tree topologies, we also considered additional lines of evidence when determining the direction of HGT, such as behavioral ecology of transfer partners and phyletic distribution of the transferred genes. For genes that are only distributed in prokaryotes and *Monosiga,* or only in algae and *Monosiga*, HGT from prokaryotes or algae to *Monosiga* was concluded; for genes with algal affiliation and sometimes broad distributions in diverse eukaryotic lineages, HGT from algae to *Monosiga* was inferred. Such inference of HGT direction can be justified based on: 1) *Monosiga* is phagotrophic and consumes algae and bacteria as food [36, 37]; 2) bacteria and many algal groups are more ancient than *Monosiga*; HGT in reverse directions would require ancestors of some major bacterial or algal groups as recipients, or it might entail multiple secondary transfer events among bacteria and algae; both are less likely scenarios. We should note here that some previously defined autotrophic algae are actually mixotrophic [38, 39] and, therefore, the possibility that these mixotrophs acquired genes from *Monosiga* cannot be excluded. However, given its highly efficient feeding activities, *Monosiga* may far more frequently be predators than being prey.

**Figure 1:**
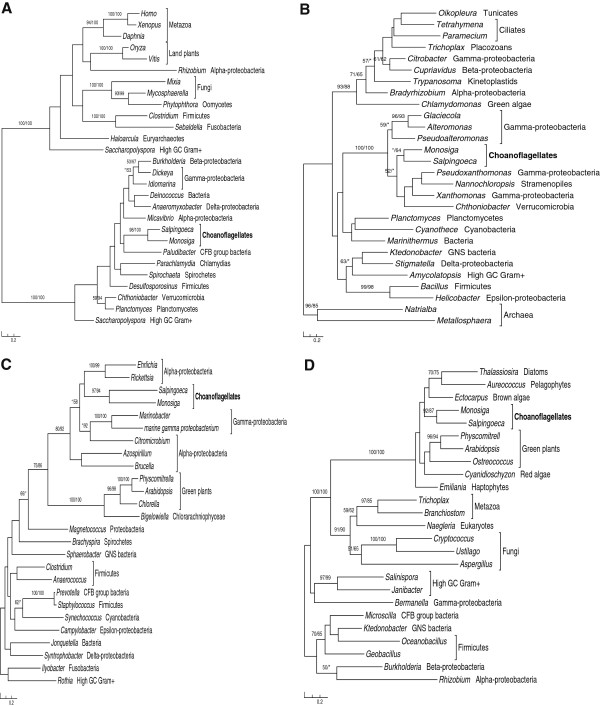
**Molecular phylogenies of bacterial or algal genes in** ***M. brevicollis*** **. A**. L-threonine 3-dehydrogenase (GenBank accession number: XP_001746273). **B**. D-beta-hydroxybutyrate dehydrogenase (GenBank accession number: XP_001744068). **C**. Metallo-beta-lactamase (GenBank accession number: XP_001747251). **D**. L-galactono-1,4-lactone dehydrogenase (GenBank accession number: XP_001748157). Numbers associated with branches show bootstrap values from maximum likelihood and distance analyses, respectively. Asterisks indicate bootstrap values lower than 50%. Taxonomic affiliations are shown after genus names, with choanoflagellates bolded.

In addition to the algal and bacterial affiliations, anomalous relationships among other taxa can be observed in most gene trees in our analyses, where multiple eukaryotic sequences sporadically branch with prokaryotic homologs (Figure 1; Additional file 1). Such anomalous relationships are somewhat expected, given the frequent HGT within and between domains [1, 40], EGT from mitochondria, plastids and other endosymbionts [41], as well as homologous replacements [42]. In theory, differential gene loss can always be invoked as an explanation alternative to HGT. Although we cannot confidently exclude the possibility of differential gene loss, the patchy distribution of most putatively transferred genes in distantly related taxa would otherwise invoke many gene losses in other groups, a less parsimonious scenario. It should be cautioned, however, that this list of putatively acquired genes in *Monosiga* will likely change when improved phylogenetic methods and larger taxonomic samplings become possible in future.

Upon further manual curation, 405 genes in *M. brevicollis* were found to be more closely related to sequences from prokaryotes and/or algae (Additional file 1), more than 80% of which contain introns (Additional file 1: Table S1). Interestingly, after comparing with our previous studies [31] and unpublished data, we found that 17 genes were absent from the candidate lists predicted by all three programs. Three of these genes were identified when we studied the evolutionary history of the branched aspartate-derived pathway [31]; 14 other genes were identified when we performed analyses on other candidates. Most of these missed genes have an alien index score (bit score ratio between the top hit from distantly related taxa and that from closely related taxa) less than 1.2, which is the default setting of AlienG. Increasing alien index would produce fewer false positives in the prediction, but might miss true positives [21].

**Figure 2:**
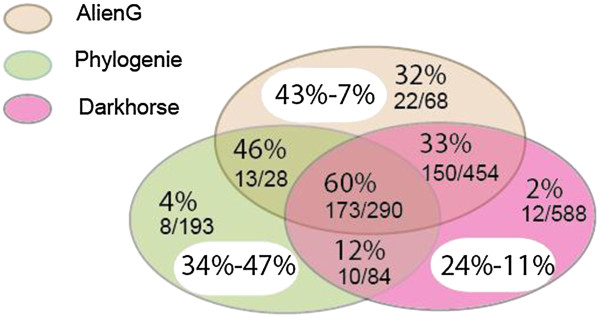
**Evaluation of three computational programs on prediction of prokaryotic and algal genes in** ***M. brevicollis*** **.**ÉFor AlienG, the alien index threshold was set to 1.2. For PhyloGenie, bootstrap value threshold for interested branches was set to 50%. Prediction results from three programs are shown in three different colors. The percentages in white ovals indicate positive rates (before hyphen) and false negative rates (after hyphen). The percentage in colored background indicates the positive rate for each part and is shown above. The numbers of foreign genes identified by manual curation (before slash) and originally predicted (after slash) are shown below.

Of the 388 remaining genes, 358 (92.3%) were predicted by AlienG, and 345 (88.9%) and 204 (52.6%) by DarkHorse and PhyloGenie, respectively (Figure 2). The positive rate of AlienG in HGT prediction (43%) is also higher than those of PhyloGenie (34%) and DarkHorse (24%) (Figure 2). Other than the algorithmic difference, the better performance of AlienG may be attributed to the larger customized database used in the analyses. Because these three programs are based on different algorithms, analyses using a combination of two or all three programs would increase the total number of acquired genes identified. It is also important to note that some transferred genes could still be missed due to the balance between prediction sensitivity and specificity [21], which is reflected in the parameter settings.

### Active feeding and gene acquisition in *Monosiga*

Of all 405 genes identified in our analyses, 240 were likely acquired from algae, 139 from bacteria, and 26 from either bacteria or algae. Because gene duplication may occur after HGT, we also estimated the number of HGT events by counting the acquired genes clustering together in the phylogenetic trees as a single event. The results suggested about 210 HGT events from algae, 100 from bacteria, and 20 from either bacteria or algae. Therefore*,* HGT from algae occurred nearly twice as frequently as those from bacteria. This raises an interesting question whether these algal genes resulted from past plastid (or algal) endosymbioses or from other sources. It is theoretically possible that the large number of algal genes detected in this study might have resulted from a historical plastid in *Monosiga* or choanoflagellates, even though no plastids or algal endosymbionts have ever been found in them. On the other hand, *M. brevicollis* is a protozoan species feeding on bacteria and microscopic algae. Based on the hypothesis “you are what you eat” [5], it is also likely that *M. brevicollis* acquired a large number of foreign genes from food sources.

Circumstantial evidences for the mechanism of gene acquisition may come from the details of HGT events and the lifestyles of recipient organisms. Although both active feeding and historical plastids (or algal endosymbionts) may explain the impressive number of algal genes in *M. brevicollis*[16], the numbers and sources of acquired genes through these two processes are different. Because any specific endosymbiont (including the plastid) will have a fixed gene pool, the number and sources of genes acquired from this endosymbiont are limited. By contrast, gene acquisition through feeding activities has no such strict limitation. Theoretically, phagotrophic protists could acquire a large number of foreign genes from diverse food sources over time, and their diet may be reflected in the sources (or donors) of acquired genes. The proportion of acquired genes in *Monosiga* genome (4.4%) is considerably higher than reported in many protozoan eukaryotes [8, 9, 40, 43, 44], but is in line with those reported in some other free-living microbial eukaryotes such as the red alga *Galdieria sulphuraria*[45] and bdelloid rotifers [46]. The potential donors for these acquired genes include diverse microscopic algal lineages such as green algae (*Micromonas* and *Ostreococcus*), diatoms (*Thalassiosira* and *Phaeodactylum*), haptophytes (*Emiliania* and *Isochrysis*), pelagophytes (*Aureococcus*), as well as numerous bacterial taxa, all of which are abundant and coexist in the same marine habitat with *M. brevicollis*. Given these considerations, we reason that many of the algal and bacterial genes identified in *Monosiga* are likely derived from food sources. However, because of the complication related to HGT identification (see above section), other scenarios cannot be definitely excluded. Such scenarios may include transfer events associated with parasites or other pathogens, viruses, mobile gene elements, phylogenetic artifacts, and misinterpretation due to insufficient taxon sampling.

### Acquired genes and the adaptation of *Monosiga*

HGT in prokaryotes has been extensively studied [1, 47] and its role in eukaryotic evolution has gained increasing appreciation. Like in prokaryotes, HGT in eukaryotes can confer adaptive traits to recipient organisms and allow them to utilize new resources or explore new niches. For instance, it has been suggested that anaerobic diplomonads were derived from an aerobic ancestor, and their adoption of an anaerobic lifestyle was facilitated by the acquisition of anaerobic metabolism-related genes from prokaryotes [8]. Comparative genomic analyses also identified 84 foreign genes in the diplomonad parasite *Spironucleus salmonicida*, suggesting an important impact of HGT on diplomonad genome evolution [48]. The role of algal genes in the adaptation of *M. brevicollis* has been discussed in previous studies [15, 16, 49]. A more complete list of acquired genes identified in this study allows better understanding of HGT in the evolution and adaptation of *Monosiga*.

**Figure 3:**
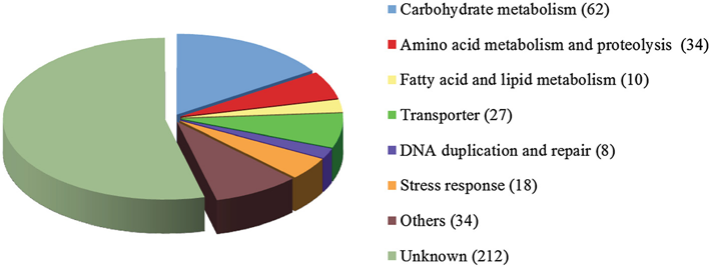
**Functional categories for genes acquired from algae and bacteria in** ***M. brevicollis*** **.**

Of all 405 genes identified in this study, 212 have unknown biological functions, but 89 of them do contain known domains. We categorized the remaining 193 genes according to their putative biological functions (Figure 3). About one third of them (32.1%, 62 genes) are related to carbohydrate metabolism, 28 of which were also identified in earlier analyses [15, 16, 31, 49] and 34 are newly reported in this study (Additional file 1). Because of the importance of carbohydrates as basic energy sources and structural components, carbohydrate metabolism is interwoven with multiple other biochemical processes. Thirteen genes identified in our analyses encode glycoside hydrolases, which are common enzymes and involved in nutrient uptake and plant cell wall degradation. Acquisition of genes encoding glycoside hydrolases has also been reported in other organisms including rumen ciliates and the rumen fungus *Orpinomyces*, where the acquired genes are critical for the recipient organisms to adapt to an anaerobic, carbohydrate-rich environment [50, 51]. Likewise, acquisition of multiple carbohydrate metabolism-related genes might allow *M. brevicollis* to digest diverse food sources.

The second largest functional category includes genes related to amino acid metabolism and protein degradation (Additional file 1). Among them, 12 acquired genes are related to proteolysis. Twenty-two genes are involved in the metabolism of amino acids, such as the biosynthesis of lysine, glutamate, histidine, and aspartate. In particular, acquired genes in *Monosiga* contributed greatly to the establishment of the branched aspartate-derived pathway that is responsible for the biosynthesis of methionine, isoleucine, threonine, and lysine [31]. All *Monosiga* genes specific to the diaminopimelic acid (DAP) pathway of lysine biosynthesis were acquired from either bacteria or algae [31]. By acquiring or improving capabilities of protein degradation and amino acid metabolism, *M. brevicollis* might ensure sufficient supply of amino acids. Ten other genes identified in our analyses are related to fatty acid and lipid metabolism (Additional file 1). In total, 106 acquired genes are related to metabolism of carbohydrates, proteins, or lipids, indicating foreign genes might have played an important role in basic and essential biological processes of *M. brevicollis*.

Some other HGT-derived genes are related to the biosynthesis of important metabolites. For example, L-galactono-1, 4-lactone dehydrogenase (Figure 1D) and 1, 4-dihydroxy-2-naphthoate octaprenyl-transferase are involved in the biosynthesis of vitamins C and K12, respectively. Given the antioxidant activities of vitamin C, acquisition of genes related to vitamin C biosynthesis might allow *M. brevicollis* to tolerate oxidative stress. Five other acquired genes are involved in oxidative stress response, two of which encode ascorbate peroxidase and have been reported previously [15] (Additional file 1). Because oxidative stress may damage cellular contents such as DNA, lipids and proteins, organisms developed various antioxidant defense mechanisms [52, 53]. Of the above six antioxidant-related genes, the osmotically inducible protein C (OsmC) and alkyl hydroperoxide reductase/thiol specific antioxidant (AhpC/TSA) protein families encode antioxidant enzymes as part of the enzymatic defense systems [54, 55], while the remaining four genes are involved in the biosynthesis of ascorbate, the ionized form of ascorbic acid (vitamin C), and belong to the non-enzymatic defense systems [56–58]. Additionally, several other identified genes are functionally related to resistance to heavy metal toxicity, osmotic stress, and pathogen infection (Additional file 1). For example, mercuric reductase might allow *M. brevicollis* to reduce mercury to nontoxic forms, and enterotoxin may be important in defense against pathogen infection. Acquisition of genes related to stress response would potentially facilitate *M. brevicollis* to adapt to various habitats, which might partly explain the wide distribution of *Monosiga* in marine ecosystems.

For protists engaging phagocytosis such as ciliates, food particles are firstly digested in phagolysosomes, and nutrients are then released and transported to the cytosol to be utilized in other metabolic processes [59]. Consequently, a complex transporter system is important for phagotrophic protists to shuffle metabolic products (e.g., amino acids, nucleotides, phosphates and sugars) and release nutrients from the phagolysosomes to the cytosol. For instance, genes encoding UDP-galactose translocator identified in our analyses are responsible for nucleotide and sugar transport [60, 61]. Thirteen of the 27 acquired transporter genes in *Monosiga* are responsible for ion transfer, such as the Ca^2+^/cation antiporter (CaCA) family participating in Ca^2+^ homeostasis and signaling [62] and the potassium inwardly-rectifying channel for maintenance of K^+^ homeostasis [63]. Intriguingly, a gene encoding multidrug efflux transporter, which confers resistance to toxins in bacteria and plants [64], was also found in *Monosiga* and may allow *Monosiga* to pump out toxic compounds. These transporter-related genes might represent an adaptation of *Monosiga* to a phagotrophic lifestyle and marine environments, where variable ion concentrations and toxic substances may be common.

Acquired genes may either introduce novel functions or replace pre-existing homologs. Introduction of novel functions or phenotypes may potentially aid the adaptation of recipient organisms to their environments [15]. Of the 405 identified genes, 192 have no identifiable homologs in another choanoflagellate *Salpingoeca rosetta*, representing HGT events after the divergence of *Monosiga* and *Salpingoeca*, or alternatively, HGT events prior to the divergence of the two organisms followed by gene loss in the latter. The remaining 213 genes in *M. brevicollis* are also present in *S. rosetta* (Figure 1A-D; Additional file 1), indicating that most genes identified in our analyses were acquired prior to the divergence of *Monosiga* and *Salpingoeca*. Many of these acquired genes fall into different categories discussed above, suggesting a possibly profound impact of HGT on the evolution of *M. brevicollis* and other choanoflagellates.

### The scale of HGT in *Monosiga*

Prokaryotic genomes are usually fluid as a result of pervasive and dynamic HGT events [65]. Such fluid genomes are often linked to the widespread distribution and tremendous metabolic variation of individual species. It has been suggested that individual prokaryotic organisms sample genes from a large global gene pool or pan-genome in response to shift in niches and resources [66, 67]. In eukaryotes, although acquired genes have been reported in many studies [7–9, 16, 44, 51, 68, 69], the overall scale of HGT in eukaryotes remains elusive. Because the evolutionary impact of HGT is largely correlated to the number of acquired genes, such a scale is critical for understanding genome evolution and speciation of recipient organisms.

To date, numerous cases of HGT have been reported in microbial eukaryotes, particularly phagotrophic microbes [3, 5, 70]. For example, about 20% of genes encoding plastid-targeted proteins in the chlorarachniophyte *Bigelowiella natans* were likely acquired through HGT events [7]. Fifteen HGT-derived genes were identified in diplomonad parasites [8] and 96 genes of prokaryotic origin in the parasite *Entamoeba histolytica*[9]. About 4.1% of ESTs from rumen ciliates were interpreted as derived from prokaryotes, most of which are related to the degradation of plant cell wall [51]. Several recent studies also indicate that up to 3.34% of protein-coding genes in the root-knot nematode *Meloidogyne incognita*[61]*,* at least 5% in the red alga *G. sulphuraria*[45] and 8-9% in the bdelloid rotifer *Adineta ricciae* were acquired from other organisms [46]. Although the methods and criteria used in above analyses might be different, available data indicate that the rate of HGT may vary among eukaryotic lineages.

Our analyses identified 405 putatively HGT-derived genes, which account for approximately 4.4% (405/9,200) of the *Monosiga* genome. This number is among the highest HGT frequencies reported for protozoan eukaryotes, but still substantially lower than that reported in bdelloid rotifers. It should be noted here that our analyses are largely based on initial genome screening using three computational programs, none of which predicts all the identified genes. This indicates that available computational programs may not be able to identify all acquired genes in a genome. Several other factors may lead to possible underestimation of the HGT scale in this study. For instance, many genes of patchy distribution, which is frequently associated with gene transfer [44], are not considered in our analyses. Additionally, anciently acquired genes, such as those acquired by the common ancestor of choanoflagellates and animals, and genes acquired from many other eukaryotic lineages are also not included in our data. In fact, the very dynamic nature of HGT can be evidenced by the ultimately bacterial origin of many algal genes in *Monosiga*, which suggests recurrent HGT among different lineages (i.e. HGT from bacteria to algae and then to *Monosiga*) [16]. This mirrors the suggestion that the patchy distribution of many genes may be attributed to frequent HGT and gene losses [44]. Therefore, we expect that the overall scale of HGT in *Monosiga* would be higher than our current finding, even though the evolutionary histories depicted for some identified genes may be different with more data becoming available.

## Conclusions

Based on the performance comparison of three common computational programs (i.e., PhyloGenie, DarkHorse, and AlienG) in HGT prediction, we recommend that a combination of two or all three programs be used to identify acquired genes. HGT contributes approximately 4.4% of the *Monosiga* genome. Many of the acquired genes in *Monosiga* are probably derived from food sources. Acquired genes are involved in different metabolic processes and stress responses, and they might have played a significant role in the adaptation of *M. brevicollis* to its environments.

## Methods

### Database selection

Predicted protein sequences of the choanoflagellate *M. brevicollis* were downloaded from the Joint Genome Institute (http://genome.jgi-psf.org/Monbr1/Monbr1.download.ftp.html). The NCBI *nr* protein sequence database was used in DarkHorse analyses, and two customized databases were constructed for PhyloGenie and AlienG analyses, respectively. The database for PhyloGenie analyses contained genomic or EST sequences of 260 representative taxa from all three domains of life, of which 15 were from archaea, 126 from bacteria, and 119 from eukaryotes. For AlienG analyses, the NCBI *nr* database was combined with genomic or EST sequences of 59 eukaryotic representative taxa that are absent from *nr*. Complete genome sequences of heterokont *Aureococcus anophagefferens*, haptophyte *Emiliania huxleyi*, and heterolobosean *Naegleria gruberi* were downloaded from the Joint Genome Institute. Annotated protein sequences of red algal *Cyanidioschyzon merolae* were downloaded from its genome project (http://merolae.biol.s.u-tokyo.ac.jp). ESTs were downloaded from the Taxonomically Broad EST Database (TBestDB) [71] and the NCBI dbEST database, and then translated into amino acid sequences over six frames using *transeq* in EMBOSS package after removing redundancy using miraEST [72].

### Parameter settings for PhyloGenie, DarkHorse, and AlienG

Parameter settings for each of the three analyses were determined after testing with multiple sample datasets. For analyses using PhyloGenie, BLAST search was carried out against the customized database. The expectation value (E-value) cutoff and the number for alignment display were set to 10^-10^ and 250, respectively. Phylogenetic trees were constructed using a maximum of 150 sequences, with sequence length coverage over 60% of the query. All trees showing a clade of choanoflagellates, prokaryotes (bacteria and archaea) or/and algae (green plants, glaucophytes, red algae, alveolates, cryptophytes, euglenids, haptophytes, chlorarachniophytes, and stramenopiles) were retrieved using the program *phat* included in the PhyloGenie package. Analyses using DarkHorse were performed with BLAST results against *nr* database as the input file; the filter threshold was set to 1% and the self-definition to choanoflagellates. For analyses using AlienG, BLAST search was performed against the comprehensive database described above. The default parameters were used except that E-value cutoff and the number for alignment display were set to 10^-5^ and 1,000 respectively. The following three types of hits were excluded from further analyses: 1) sequences from choanoflagellates, which were used to exclude self-sequences; 2) sequences with length coverage below 10%; 3) pseudo-sequences annotated as “artificial sequences”, “synthetic construct”, or “plasmids”.

### Phylogenetic analyses

Each HGT candidate predicted by the three computational programs was subject to further manual phylogenetic analyses. Homologous sequences were sampled from representative groups of three domains of life (bacteria, archaea, and eukaryotes). The comprehensive database built for AlienG analyses was used for sequence sampling. Protein sequence alignments were performed using both MUSCLE [73] and ClustalX [74], followed by cross-comparison and manual refinement. Gaps and ambiguously aligned regions were removed manually. The alignment data are available upon request. The optimal model of protein sequence substitution and rate heterogeneity for each dataset were chosen using ModelGenerator based on the AIC1 criterion [75]. Phylogenetic analyses were performed with a maximum likelihood method using PHYML 3.0 [76] and a distance method using *neighbor* of PHYLIP version 3.69 [77], with maximum likelihood distance calculated using TREE-PUZZLE [78]. Bootstrap analyses used 100 pseudo-replicates.

### Identification of acquired genes homologs in the choanoflagellate *S. rosetta*

The genome of the choanoflagellate *S. rosetta* was not available to the public when we initiated our analyses of *M. brevicollis*. To investigate whether the genes identified in *M. brevicollis* were also acquired by *S. rosetta*, we downloaded a total of 11,731 predicted protein sequences of *S. rosetta* from the Origins of Multicellularity Sequencing Project (Broad Institute of Harvard and MIT, http://www.broadinstitute.org) [79] and then identified the homologs based on sequence similarity comparison. The acquired genes in *M. brevicollis* were used as queries to search against the genome of *S. rosetta* with E-value cutoff set to 1e-40*.* The genes shared by *M. brevicollis* and *S. rosetta* were considered to be acquired prior to the split of *S. rosetta* and *M. brevicollis*.

## Electronic supplementary material

Additional file 1: Table S1: Algal and prokaryotic genes (405) identified in *M. brevicollis*. **Figure S1-S109.** Maximum likelihood trees for the algal and bacterial genes identified in *M. brevicollis*. Genes identified in our previous studies and some of those uniquely distributed in prokaryotes and/or algae besides choanoflagellates are not included. (PDF 5 MB)

## Abbreviations

HGT: Horizontal gene transfer
EGT: Endosymbiotic gene transfer.
