# Supplementary material for: The scale and evolutionary significance of horizontal gene transfer in the choanoflagellate Monosiga brevicollis
Source: BMC Genomics. 2013 Oct 25;14(1):729. doi: 10.1186/1471-2164-14-729 (PMC4046809; doi:10.1186/1471-2164-14-729)
Supplement: Supplementary file 1 — Additional file 1: Table S1: Algal and prokaryotic genes (405) identified in M. brevicollis. Figure S1-S109. Maximum likelihood trees for the algal and bacterial genes identified in M. brevicollis. Genes identified in our previous studies and some of those uniquely distributed in prokaryotes and/or algae besides choanoflagellates are not included. (PDF 5 MB) [file 12864_2012_5464_MOESM1_ESM.pdf]

The scale and evolutionary significance of horizontal gene transfer in the choanoflagellate *Monosiga brevicollis*

Jipei Yue Guiling Sun Xiangyang Hu Jinling Huang

Table S1. Algal and prokaryotic genes (405) identified in *Monosiga*. “^” indicates genes that were reported by earlier studies. Asterisks indicate genes that were detected in our other studies. “+” indicates genes that have homologs in another choanoflagellate *Salpingoeca rosetta*, which implies that the gene transfer may had occurred prior to the divergence of *Monosiga* and *Salpingoeca.* “y” indicates that the transferred gene has no homologs in *Monosiga* and should be gene addition to *Monosiga* genome. “~” indicates that the genes have at least one intron.

Gene product	Putative Donor	Putative Function	Gi Number	Introns	Gene addition	Homologs in Salpingoeca
1,4-alpha-glucan branching enzyme	Bacteria	Carbohydrate metabolism	167534449	~		+
6-phosphogluconolactonase^	Haptophytes/Diatoms	Carbohydrate metabolism	167536559	~		+
Alcohol dehydrogenase	Algae	Carbohydrate Metabolism	167526433	~		
Aldehyde reductase	Pelagophytes	Carbohydrate Metabolism	167519881	~		+
Aldo-keto reductase^	Chromalveolates	Carbohydrate metabolism	167522481	~		+
Alpha-galactosidase^	Chromalveolates	Carbohydrate metabolism	167519278	~		
Alpha-galactosidase^	Dinoflagellates	Carbohydrate metabolism	167535848	~		+
Alpha-galactosidase^	Land plants	Carbohydrate metabolism	167518688	~		
Alpha-galactosidase^	Pelagophytes	Carbohydrate metabolism	167521790	~		
Alpha-glucosidase	Haptophytes	Carbohydrate metabolism	167536698	~		
Alpha-L-rhamnosidase	Bacteria	Carbohydrate metabolism	167538489	~		
Alpha-N-arabinofuranosidase^	Haptophytes	Carbohydrate metabolism	167533923	~	y	
Alpha-rhamnosidase	Bacteroidetes	Carbohydrate metabolism	167524457	~		
Alpha-rhamnosidase^	Haptophytes	Carbohydrate metabolism	167524427	~		+
Alpha-rhamnosidase	Haptophytes	Carbohydrate metabolism	167533764	~		
Alpha-xylosidase	Bacteria	Carbohydrate metabolism	167536377	~		
Beta-galactosidase	Haptophytes	Carbohydrate metabolism	167525643			
Beta-glucanase^	Pelagophytes	Carbohydrate metabolism	167524340			
Beta-glucanase	Pelagophytes	Carbohydrate metabolism	167520023	~		
Beta-glucosidase^	Chromists	Carbohydrate metabolism	167537541	~		+
Beta-glucosidase^	Haptophytes	Carbohydrate metabolism	167522437	~		+
beta-mannosidase	Chromists	Carbohydrate metabolism	167523877	~		+
beta-mannosidase	Chromists	Carbohydrate metabolism	167522846	~		+
Beta-xylosidase^	Diatoms	Carbohydrate metabolism	167517889			+
Beta-xylosidase^	Haptophytes/Pelagophytes	Carbohydrate metabolism	167534300	~		
D-beta-hydroxybutyrate dehydrogenase	Bacteria	Carbohydrate metabolism	167519457	~		+
D-galactarate dehydratase^	Haptophytes/Bacteria	Carbohydrate metabolism	167535312	~	y	+
Exo-beta-1,3-glucanase	Haptophytes	Carbohydrate metabolism	167538294	~		
Galactokinase	Pelagophytes	Carbohydrate Metabolism	167535085	~		
Glucokinase	Chromists	Carbohydrate Metabolism	167537338	~	y	+
Gluconolactonase	Planctomycetes	Carbohydrate metabolism	167523807	~		+
Glycerol-3-phosphate dehydrogenase^	Land plants	Carbohydrate metabolism	167523371	~	y	+
Glycoside hydrolase	Bacteria	Carbohydrate metabolism	167524467	~	y	
Glycoside hydrolase	Chlamydiae/Verrucomicrobia	Carbohydrate metabolism	167516874	~		
Glycoside hydrolase	Bacteria	Carbohydrate metabolism	167525759	~		+
Glycoside hydrolase	Bacteria	Carbohydrate metabolism	167537858	~		
Glycoside hydrolase	Verrucomicrobia	Carbohydrate metabolism	167524693	~		

Glycoside hydrolase	Bacteria	Carbohydrate metabolism	167525968	~		
Glycosyl hydrolase	Bacteria	Carbohydrate metabolism	167525723	~		
Glycosyl hydrolase^	Haptophytes/Pelagophytes	Carbohydrate metabolism	167518261	~		+
Glycosyl hydrolase^	Dinoflagellates	Carbohydrate metabolism	167522924	~		
Glycosyl hydrolase^	Haptophytes	Carbohydrate metabolism	167535834	~		
Glycosyl hydrolase^*	Haptophytes	Carbohydrate metabolism	167535902	~		+
Glycosyl hydrolase^	Pelagophytes	Carbohydrate metabolism	167517467	~		
Glycosyl hydrolases^	Haptophytes	Carbohydrate metabolism	167520276	~		+
Heparinase II/III family protein^	Cyanobacteria	Carbohydrate metabolism	167516594	~	y	
Hexosaminidase^	Pelagophytes	Carbohydrate metabolism	167521882	~		+
Maltodextrin glucosidase	Gamma-proteobacteria	Carbohydrate metabolism	167521117	~		+
Mannitol-1-phosphate/altronate dehydrogenase	Alpha-proteobacteria	Carbohydrate metabolism	167537946		y	
Mannonate dehydratase	Prokayrotes	Carbohydrate metabolism	167524749		y	+
Mannonate dehydratase	Prokayrotes	Carbohydrate metabolism	167525781	~		+
Phosphoenolpyruvate carboxykinase	Bacteria	Carbohydrate metabolism	167519713	~	y	+
Xylosidase^	Chromists	Carbohydrate metabolism	167521708	~		+
Pirin-related protein	Algae	Carbohydrate metabolism	167538121	~		+
Dolichol phosphate mannose synthase	Dinoflagellates	Carbohydrate metabolism	167535637	~		+
Prolyl 4-hydroxylase	Algae	Carbohydrate metabolism	167524906	~		+
Pectin acetylesterase^	Haptophytes	Pectin degradation	167523529	~		+
Polygalacturonase^	Haptophytes/Diatoms	Pectin degradation	167521455	~		+
Polygalacturonase^	Algae	Pectin degradation	167536801	~	y	
Polygalacturonase	Algae	Pectin degradation	167519459	~	y	
Polygalacturonase	Algae	Pectin degradation	167521385	~		
Polygalacturonase^	Land plants	Pectin degradation	167523647	~		
Aspartate-semialdehyde dehydrogenase^	Algae	Amino acid biosynthesis	167527123	~	y	+
Bifunctional aspartate kinase/diaminopimelate decarboxylase^	Bacteria	Amino acid biosynthesis	167524549	~		+
D-Alanine aminotransferase^	Diatoms	Amino acid biosynthesis	167524583	~		+
Diaminopimelate epimerase^	Bacteria	Amino acid biosynthesis	167535696	~		
Dihydroxyacid dehydratase^*	Algae	Amino acid biosynthesis	167524042	~		+
Homoserine dehydrogenase^	Algae	Amino acid biosynthesis	167536236	~	y	+
Homoserine O-acetyltransferase^	Diatoms	Amino acid biosynthesis	167535206	~		
Amino acid aminotransferase^	Algae	Amino acid biosynthesis	167535603	~		+
Glutamate synthase^	Green plants	Amino acid biosynthesis	167519242	~		
Histidinol dehydrogenase^	Chromists	amino acid biosynthesis	167535912	~	y	+
Anthranilate synthase^	Chromists	Amino acid biosynthesis	167538764	~		+
Dihydrodipicolinate synthetase^	Bacteroidetes	Amino acid biosynthesis	167537054	~	y	+
3-dehydroquinate synthase	Bacteria	Amino acid biosynthesis	167537749	~		+
Kynurenine aminotransferase III^	Algae	Amino acid derivative metabolism	167521756	~		+
D-amino-acid dehydrogenase	Bacteria	Amino acid metabolism	167517429			+
L-threonine 3-dehydrogenase	Proteobacteria	Amino acid metabolism	167523873	~		+
Puromycin-sensitive aminopeptidase^	Chromalveolates	Amino acid metabolism	167537006	~		+
Arginase^*	Heterokonts	Amino acid metabolism	167522942	~	y	+
Glutaminase A	Bacteria	Amino acid metabolism	167522419	~	y	
Glutamate formiminotransferase	Algae	Amino acid Metabolism	167523543	~	y	
Methylenetetrahydrofolate reductase	Heterokonts	Amino acid Metabolism	167523328	~	y	+
Selenide, water dikinase	Bacteria/algae	Amino acid metabolism	167525178	~	y	+
Cysteine endopeptidase	Green algae	Proteolysis	167537940	~		+
Cysteine protease	Algae	Proteolysis	167522705	~		+
Leu/Phe-tRNA protein transferase	Chromalveolates	proteolysis	167536799	~		+
Metacaspase^	Plantae	Proteolysis	167537374	~		
Peptidase^	Alveolates	Proteolysis	167524318	~		+
Peptidase U34 dipeptidase	Algae/Bacteria	Proteolysis	167524214	~	y	
Serine carboxypeptidase^	Haptophytes/Pelagophytes	Proteolysis	167534856	~		+
Serine carboxypeptidase	Haptophytes/Pelagophytes	Proteolysis	167524356	~		+
Serine carboxypeptidase	Haptophytes	Proteolysis	167526595	~		+
Serine carboxypeptidase	Haptophytes	Proteolysis	167534702	~		+
Serine carboxypeptidase	Haptophytes	Proteolysis	167533602	~		+
Serine carboxypeptidase	Haptophytes	Proteolysis	167534625	~		+
Delta-5 desaturase	Pelagophytes	Fatty acid biosynthesis	167518353	~		+
Fatty acid desaturase^	Diatoms/Green plants	Fatty acid biosynthesis	167523184	~		+
Fatty-acyl-CoA synthetase	High GC Gram+	Fatty acid biosynthesis	167516368	~		+

Long chainfatty acid elongation enzyme^	Chromalveolates	Fatty acid biosynthesis	167538086	~		+
Fatty acid elongase	Haptophytes	Fatty acid synthesis	167526168	~		+
Esterase/lipase^	Haptophytes	Lipid metabolism	167520997			+
Acyl-CoA dehydrogenase	Bacteria	Lipid metabolism	167518089	~		+
Methyltransferase	Bacteria	Lipid metabolism	167515590			
Phosphatidate-sterol O-acyltransferase	Algae	Lipid metabolism	167525286	~		+
SAM-dependent methyltransferases	Bacteria	Lipid metabolism	167536306			
Apurinic/apyrimidinic endonuclease	Chromalveolata	DNA base excision repair	167522888	~		
DNA topoisomerase VI subunit B ^	Algae	DNA endoreduplication	167519843	~		
DNA topoisomerase VI subunit A	Algae	DNA endoreduplication	167526605	~	y	
Uracil-DNA glycosylase	Bacteria	DNA repair	167535167	~		+
Exodeoxyribonuclease V	Bacteria	DNA repair and homologous recombination	167537463	~		+
DNA ligase D	Bacteria/Algae	DNA repair and replication	167519499	~	y	+
PHP domain protein	Bacteria	DNA repair and replication	167517641	~	y	+
DNA glycosylase^	Algae	DNA replication, recombination, and repair	167535061			
Beta-lactamase	Bacteria	response to antibiotic	167536358	~		+
Metallo-beta-lactamase	Gamma-proteobacteria	response to antibiotic	167525833			+
L-2,4-diaminobutyric acid acetyltransferase	Alpha-proteobacteria	response to osmotic stress	167517491		y	
OsmC family protein	Bacteria	response to oxidative stress	167519803	~		+
Tellurium resistance protein	Bacteria/algae	Stress response	167520131	~		+
Protein phosphatase 2C	Haptophytes	Stress responses	167522497	~		+
Integral membrane protein TerC	Bacteria/algae	Tellurite resistance	167535450	~	y	+
Ascorbate peroxidase^	Green algae	Oxidative stress	167515916	~		+
Ascorbate peroxidase^	Algae	Oxidative stress	167521652	~		+
Antioxidant, AhpC/TSA family	Bacteria	Antioxidant	167515776	~		
Monodehydroascorbate reductase	Algae	Antioxidative response	167522575	~		+
Phytochelatin synthase	Algae/Bacteria	heavy metal detoxification	167537519	~		
Enterotoxin	Bacteria	Defense	167537376	~	y	+
Mercuric reductase	Algae/Bacteria	Mercuric stress	167519689	~		+
Major facilitator superfamily^	Diatoms	Xenobioic metabolism	167517553	~	y	+
Major facilitator superfamily	Diatoms	Xenobioic metabolism	167523663	~	y	+
Multidrug efflux transporter^	Haptophytes	Xenobiotic metabolism	167520702	~	y	
Ribonuclease H	Bacteria		167518678	~	y	
ABC transporter^	Green plants	Membrane transport	167522511	~		+
Mitochondrial carrier family^	Eulgenoids	Membrane transport	167521906	~		+
Mitochondrial inner membrane transporter^	Haptophytes	Membrane transport	167523769	~		+
Multidrug and toxin extrusion protein 1^	Haptophytes/Diatoms	Multidrug antitransport	167517106	~		+
UDP-galactose translocator	Algae	Nucleotide-sugar transporter	167527303	~		+
ABC phosphate/phosphonate transporter	Bacteria	Transporter	167533969	~	y	
Major facilitator transporter	Bacteria	Transporter	167518556		y	
Folate/Biopterin Transporter	Oomycetes	Transporter	167523823	~	y	+
Rhamnose-proton symporter	Bacteroidetes	Transporter (hexose)	167515428	~	y	+
Ca2 :Cation Antiporter (CaCA) Family	Chromists	Transporter (ion)	167516696			
CorA metal ion transporter	Diatoms	Transporter (ion)	167516550	~	y	
Flavin-containing monooxygenase	Bacteria	Transporter (ion)	167534098	~		
Ion channel protein	Algae	Transporter (ion)	167534102	~		+
Potassium inwardly-rectifying channel^	Chromalveolates	Transporter (ion)	167515450	~		+
P-type ATPase	Algae	Transporter (ion)	167534304	~		+
Sulphate transporter	Oomycetes	Transporter (ion)	167535659		y	+
Sulphate transporter	Oomycetes	Transporter (ion)	167538557	~		+
Sulphate transporter	Chromists	Transporter (ion)	167534551	~		+
Sulphate transporter^	Chromists	Transporter (ion)	167518067	~	y	+
TrkA (Na+/H+ antiporter)	Bacteria/algae	Transporter (ion)	167526916	~		
TrkA (Na+/H+ antiporter)	Bacteria/algae	Transporter (ion)	167537773	~		
Zinc transporter ZupT^	Pelagophytes	Transporter (ion)	167515920	~		
Sorbitol transporter	Green algae	Transporter (sugar)	167523619	~		+
Sorbitol transporter^	Green algae	Transporter (sugar)	167521355	~		+
ABC transporter	Red algae	Transportor	167520081	~		+
ABC transporter	Red algae	Transportor	167535768	~		+
ABC transporter	Red algae	Transportor	167517635	~		+
L-galactono-1,4-lactone	Algae	Vitamin C biosynthesis	167530871	~		+

dehydrogenase						
1,4-dihydroxy-2-naphthoate octaprenyl- transferase	Bacteria	Vitamin K2 biosynthesis	167519453		y	+
Alpha-L-arabinofuranosidase	Haptophytes	Amino sugar and nucleotide sugar metabolism	167533053	~	y	
Alpha-L-arabinofuranosidase*	Haptophytes	Amino sugar and nucleotide sugar metabolism	167519743	~	y	+
Asparaginyl-tRNA synthetase	Green plants	Aminoacyl-tRNA biosynthesis	167527404	~		+
N-acylglucosamine 2-epimerase	algae/bacteria	Aminosugars metabolism	167527127	~		
Fumarate reductase/succinate dehydrogenase	Planctomycetes	Anaerobic respiration	167526295	~		
Ribonuclease D	Bacteria	Structured RNA processing	167533742	~		
Sulfatase	Haptophytes	Sulfate metabolism	167527083	~		
Sulfotransferase	Diatoms/Green algae	Sulfate metabolism	167518043	~		+
Ser/Thr protein phosphatase	Chromists	Signal transduction pathways	167534824		y	
NUDIX hydrolase^	Diatoms	NUDIX hydrolysis	167518355	~		+
Disulfide oxidoreductase^	Chromalveolates	Cell redox homeostasis	167525441	~		+
Ubiquinone biosynthesis protein coq-8	Algae	ubiquinone biosynthesis.	167519152	~		+
Regulatory protein CII	Bacteria	Cell division and chromosome partitioning	167534509		y	
Regulatory protein CII	Bacteria	Cell division and chromosome partitioning	167526174	~		
Regulatory protein CII	Haptophytes/Bacteria	Cell division and chromosome partitioning	167536551			
Lysophospholipase	Bacteria/algae	glycerophospholipid metabolism	167516992	~	y	
Iduronate-2-sulfatase	Haptophytes	Glycosaminoglycan degradation	167524240	~		+
Mannosyltransferase^	Chlorarachniophytes	Glycosphingolipid biosynthesis	167523914			+
Methylenetetrahydrofolate dehydrogenase	Heterokonts	Glyoxylate and dicarboxylate metabolism	167523990	~		
Cytochrome B561	Algae	Energe metabolism	167516960	~	y	+
Inorganic polyphosphate/ATP-NAD kinase	Bacteria	Energe metabolism	167516892	~		+
Mitochondrial carrier protein	Algae	Energy transfer	167524336	~		+
2-nitropropane dioxygenase	Alpha-proteobacteria	nitrogen metabolism	167538159		y	
Flavodoxin	Bacteria	Nitrogen metabolism	167533501	~	y	
Isocitrate dehydrogenase (NAD+)^	Haptophytes	Tricarboxylic acid cycle	167517034	~		+
Isocitrate dehydrogenase	bacteria	Tricarboxylic acid cycle	167534021	~		+
Isocitrate dehydrogenase (NAD+)	Bacteria	Tricarboxylic acid cycle	167518099	~		+
Sn-1,2-diacylglycerol ethanolamine-and cholinephosphotranferases (ISS)^	Green algae	Phospholipid biosynthesis	167537529	~	y	+
Uroporphyrinogen III synthase/methyltransferase	Algae/cyanobacteria	Porphyrin biosynthesis	167515658	~		+
Glutathione S-transferas	proteobacteria	Posttranslational modification	167523232	~		
Peptidyl-prolyl cis-trans isomerase	Bacteria	Protein folding	167527426	~		
CMP/dCMP deaminase	Gamma-proteobacteria	nucleotide biosynthesis	167538365		y	
2OG-Fe(II) oxygenase	Algae	Unknown	167535670			+
Acetyltransferase	Alpha-proteobacteria	Unknown	167525825		y	
Acid ceramidase	Alveolates	Unknown	167517231	~		
Acid phosphatase	Algae	Unknown	167518307	~		+
Alcohol dehydrogenase class III	Haptophytes	Unknown	167521587	~		+
Alcohol dehydrogenase class III	Haptophytes	Unknown	167522892	~		+
Aldo-ketoreductase	Algae	Unknown	167536740	~		+
Aminoglycoside phosphotransferase	Bacteria	Unknown	167537263	~	y	+
Aminotransferase^	Dinoflagellates/ Green plants	Unknown	167521427			+
Arylsulfatase^	Haptophytes	Unknown	167521718	~		
Arylsulfatase^	Chromists/bacteria	Unknown	167524429	~		
Arylsulfatase B^	Pelagophytes	Unknown	167524284	~		+
BNR/Asp-box repeat protein	Bacteria/green algae	Unknown	167520678	~	y	
Calcineurin-like phosphoesterase^	Green algae	Unknown	167535073	~		+
Cupin 2 domain-containing protein	Gamma-proteoba	Unknown	167527452		y	

	cteria					
Dehydrogenase^	Diatoms	Unknown	167524741	~		+
FAD dependent oxidoreductase^	Haptophytes	Unknown	167525936	~		+
FAD-dependent pyridine nucleotide-disulphide oxidoreductase	Alpha-proteobacteria	Unknown	167523218			
Glycosyl transferase	Bacteria	Unknown	167525785	~	y	
Glycosyl transferase*	Bacteria	Unknown	167525775	~		
Glycosyl transferase	Bacteria	Unknown	167525773	~		
Glycosyl transferase*	Bacteria	Unknown	167523096	~	y	
Glycosyl transferase*	Bacteria	Unknown	167538246	~	y	
Glycosyl transferase*	Bacteria	Unknown	167533259	~		
Glycosyl transferase*	Bacteria	Unknown	167530881	~		
Glycosyl transferase*	Bacteria	Unknown	167533829	~		
Glycosyl transferase	Bacteria	Unknown	167520177	~	y	
Glycosyl transferase*	Bacteria	Unknown	167523100	~	y	
Glycosyltransferase	Delta-proteobacteria	Unknown	167522307		y	+
GTP-binding protein	Bacteria/algae	Unknown	167522240	~		
KH domain-containing protein	Algae	Unknown	167536547	~		
Lipoprotein	Bacteria	Unknown	167522473	~		
Methyltransferase	Alpha-proteobacteria	Unknown	167535810	~		
Methyltransferase	Bacteria	Unknown	167538335	~		
Methyltransferase^	Algae/Bacteria	Unknown	167519769	~		
Methyltransferase	Green algae	Unknown	167518566	~		+
Oxidoreductase	Algae	Unknown	167520798	~		+
Oxidoreductase	Algae	Unknown	167538446	~		+
Phospholipid scramblase^	Haptophytes/Pelagophytes	Unknown	167520670	~	y	
Phospholipid scramblase	Haptophytes/Pelagophytes	Unknown	167520860	~	y	
SCP-like extracellular protein	Bacteria	Unknown	167534469	~		
SCP-like extracellular protein	Bacteria	Unknown	167524445	~		
SCP-like extracellular protein	Bacteria	Unknown	167523453	~		+
Short chain dehydrogenase/reductase^	Algae/Bacteria	Unknown	167525395	~		
Short-chain dehydrogenase/reductase	Lentisphaera	Unknown	167515652	~		+
Short-chain dehydrogenase/reductase SDR	Bacteria	Unknown	167524876			
Short-chain dehydrogenase/reductase SDR	Bacteria	Unknown	167535923	~		
Short-chain dehydrogenease	Chromists	Unknown	167516176	~		+
Sialidase	Dinoflagellates/ Haptophytes	Unknown	167535145	~		+
Sialidase	Dinoflagellates/ Haptophytes	Unknown	167534965	~		+
S-layer domain protein^	Haptophytes	Unknown	167519719	~		+
Hypothetical protein	Bacteria	Unknown	167516190	~		
Hypothetical protein	Bacteria	Unknown	167516536	~		+
Hypothetical protein	Bacteria	Unknown	167526182	~		+
Hypothetical protein	Bacteria	Unknown	167524815	~		+
Hypothetical protein	Bacteria	Unknown	167526184	~		+
Hypothetical protein	Bacteria	Unknown	167524673	~		+
Hypothetical protein*	Bacteria	Unknown	167517815	~		+
Hypothetical protein	Bacteria	Unknown	167526357	~	y	+
Hypothetical protein	Bacteria	Unknown	167517094	~		
Hypothetical protein	Bacteria/algae	Unknown	167518634	~	y	
Hypothetical protein	Verrucomicrobia	Unknown	167525024	~	y	
Hypothetical protein	Bacteria	Unknown	167518772	~		
Hypothetical protein	Bacteria	Unknown	167520870	~		
Hypothetical protein	Bacteria	Unknown	167520868	~		
Hypothetical protein	Bacteria	Unknown	167520872	~		
Hypothetical protein	Bacteria	Unknown	167516916	~	y	+
Hypothetical protein	High GC Gram+	Unknown	167537457			
Hypothetical protein	Alpha-proteobacteria	Unknown	167534872		y	
Hypothetical protein	Bacteria	Unknown	167534537			
Hypothetical protein	Bacteria	Unknown	167536797	~		
Hypothetical protein	Bacteria	Unknown	167522609	~		
Hypothetical protein	Delta-proteobacteria	Unknown	167516134	~		+
Hypothetical protein	Bacteria	Unknown	167517251	~		
Hypothetical protein	Gamma-proteobacteria	Unknown	167525389	~	y	+
Hypothetical protein	Cyanobacteria	Unknown	167525585	~		+

Hypothetical protein	Verrucomicrobia	Unknown	167527348	~		
Hypothetical protein	Delta-proteobacteria	Unknown	167534158		y	
Hypothetical protein	Proteobacteria	Unknown	167537711			
Hypothetical protein	Proteobacteria	Unknown	167519308			
Hypothetical protein	Bacteria	Unknown	167538638	~	y	
Hypothetical protein	Bacteria	Unknown	167522445	~	y	
Hypothetical protein	CFB group bacteria	Unknown	167525577	~		+
Hypothetical protein	CFB group bacteria	Unknown	167523224	~		+
Hypothetical protein	CFB group bacteria	Unknown	167534971	~		+
Hypothetical protein	Bacteria	Unknown	167520300		y	
Hypothetical protein	Bacteria	Unknown	167520392			
Hypothetical protein	Bacteria	Unknown	167519501	~		
Hypothetical protein	Bacteria	Unknown	167521820	~	y	+
Hypothetical protein	Bacteria	Unknown	167524290	~		
Hypothetical protein	Beta-proteobacteria/g-proteobacteria	Unknown	167524517	~		+
Hypothetical protein	Beta-proteobacteria/g-proteobacteria	Unknown	167527414	~	y	
Hypothetical protein	Bacteria	Unknown	167519801	~		
Hypothetical protein	bacteria	Unknown	167538879	~		
Hypothetical protein	Bacteria	Unknown	167522044	~		+
Hypothetical protein	Bacteria	Unknown	167521780	~		+
Hypothetical protein	Bacteria	Unknown	167517359	~		+
Hypothetical protein	Bacteria	Unknown	167517361			+
Hypothetical protein	Bacteria	Unknown	167538949			+
Hypothetical protein*	Bacteria	Unknown	167538877	~	y	
Hypothetical protein	Bacteria	Unknown	167523489	~		+
Hypothetical protein*	Bacteria	Unknown	167517357	~		
Hypothetical protein	Bacteria	Unknown	167519272			
Hypothetical protein	Bacteria	Unknown	167516668	~	y	
Hypothetical protein	Bacteria	Unknown	167521495	~	y	
Hypothetical protein	Bacteria	Unknown	167525407	~	y	+
Hypothetical protein	Bacteria	Unknown	167524254	~		+
Hypothetical protein	Algae/bacteria	Unknown	167525365	~		+
Hypothetical protein	Bacteria/Algae	Unknown	167534180			
Hypothetical protein	Bacteria/Algae	Unknown	167520220			
Hypothetical protein	Bacteria/Algae	Unknown	167518652			
Hypothetical protein	Bacteria/Algae	Unknown	167534457	~		
Hypothetical protein	Bacteria/algae	Unknown	167524962			
Hypothetical protein	Bacteria/algae	Unknown	167515878	~		+
Hypothetical protein^	Diatoms	Unknown	167524352			
Hypothetical protein^	Haptophytes/Bacteria	Unknown	167527544	~	y	
Hypothetical protein^	Haptophytes	Unknown	167533720	~		
Hypothetical protein^	Pelagophytes	Unknown	167535836	~		
Hypothetical protein^	Haptophytes	Unknown	167516378	~	y	+
Hypothetical protein^	Chlorarachniophytes	Unknown	167515756	~	y	+
Hypothetical protein^	Diatoms	Unknown	167516746	~	y	
Hypothetical protein^	Green algae	Unknown	167519867	~	y	+
Hypothetical protein^	Haptophytes	Unknown	167521071	~		+
Hypothetical protein^	Haptophytes/Green algae	Unknown	167521419	~		
Hypothetical protein	Haptophytes/Green algae	Unknown	167519164			
Hypothetical protein^	Haptophytes	Unknown	167521523	~		+
Hypothetical protein^	Chlorarachniophytes	Unknown	167523014			
Hypothetical protein^	Diatoms/Green algae	Unknown	167524172	~	y	
Hypothetical protein^	Alveolates	Unknown	167517835			
Hypothetical protein^	Algae	Unknown	167534417	~		
Hypothetical protein^	Algae	Unknown	167535049			
Hypothetical protein	Algae	Unknown	167537418			
Hypothetical protein^	Haptophytes/Diatoms	Unknown	167538161	~		+
Hypothetical protein^	Diatoms	Unknown	167518856	~		+
Hypothetical protein	Diatoms	Unknown	167518035	~		+
Hypothetical protein^	Haptophytes	Unknown	167517175	~	y	+
Hypothetical protein^	Diatoms	Unknown	167519649			
Hypothetical protein^	Diatoms	Unknown	167523603	~		

Hypothetical protein^	Algae	Unknown	167524735	~	y	
Hypothetical protein^	Green algae	Unknown	167527896	~		+
Hypothetical protein^	Algae	Unknown	167519388	~		+
Hypothetical protein^	Haptophytes	Unknown	167519757	~		
Hypothetical protein^	Haptophytes/Diatoms	Unknown	167521636	~	y	
Hypothetical protein^	Green algae/Cyanobacteria	Unknown	167522192		y	
Hypothetical protein^	Green plants	Unknown	167522289	~	y	+
Hypothetical protein^	Haptophytes	Unknown	167525437	~	y	+
Hypothetical protein^	Algae	Unknown	167525685	~		+
Hypothetical protein	Algae	Unknown	167524144			
Hypothetical protein	Algae	Unknown	167537535		y	
Hypothetical protein	Haptophytes	Unknown	167515570	~	y	
Hypothetical protein	Algae	Unknown	167524298	~		+
Hypothetical protein	Algae	Unknown	167534947	~	y	+
Hypothetical protein	Haptophytes	Unknown	167533523		y	+
Hypothetical protein	Haptophytes	Unknown	167515422	~	y	+
Hypothetical protein	Euglenoids	Unknown	167523859	~	y	+
Hypothetical protein	Algae	Unknown	167516636	~	y	
Hypothetical protein	Algae	Unknown	167521998	~		
Hypothetical protein	Algae	Unknown	167537777	~		+
Hypothetical protein	Algae	Unknown	167522848	~	y	+
Hypothetical protein	Red algae	Unknown	167516356	~		+
Hypothetical protein	Green algae	Unknown	167534491	~	y	+
Hypothetical protein	Green plants	Unknown	167537477	~		+
Hypothetical protein	Algae	Unknown	167537588	~		
Hypothetical protein	Chromists	Unknown	167538097	~		
Hypothetical protein	Diatoms	Unknown	167516062	~		+
Hypothetical protein	Algae	Unknown	167519256	~		+
Hypothetical protein	Dinoflagellates	unknown	167519849	~	y	
Hypothetical protein	Green algae	Unknown	167525567	~	y	
Hypothetical protein	Haptophytes	Unknown	167527021	~		+
Hypothetical protein	Algae	Unknown	167534047	~	y	+
Hypothetical protein	Haptophytes	Unknown	167536775	~	y	+
Hypothetical protein	Haptophytes	Unknown	167520576	~		
Hypothetical protein	Algae	Unknown	167522591	~	y	
Hypothetical protein	Algae	Unknown	167533453	~		+
Hypothetical protein	Chromists	Unknown	167534124	~	y	
Hypothetical protein	Cryptophytes	Unknown	167538169	~		+
Hypothetical protein	Green algae	Unknown	167515382	~		+
Hypothetical protein	Green algae	Unknown	167538595	~		+
Hypothetical protein	Chromists	Unknown	167526275	~		+
Hypothetical protein^	Chromists	Unknown	167538724	~	y	+
Hypothetical protein	Chlorarachniophytes	Unknown	167534439	~		+
Hypothetical protein	Chromists	Unknown	167535947	~		+
Hypothetical protein	Chromalvelates	Unknown	167523044	~		+
Hypothetical protein	Algae	Unknown	167516234	~	y	
Hypothetical protein	Algae	Unknown	167522493	~	y	
Hypothetical protein	Haptophytes	Unknown	167515494		y	
Hypothetical protein	Chromists	Unknown	167524032	~		+
Hypothetical protein	Algae	Unknown	167516576	~	y	+
Hypothetical protein	Chromalvelates	Unknown	167520320	~		
Hypothetical protein	Algae	Unknown	167533027		y	
Hypothetical protein	Algae	Unknown	167515760		y	
Hypothetical protein	Algae	Unknown	167521007			
Hypothetical protein*	Algae	Unknown	167521009		y	
Hypothetical protein	Algae	Unknown	167521005	~		
Hypothetical protein*	Algae	Unknown	167535041	~		+
Hypothetical protein	Algae	Unknown	167523256	~	y	
Hypothetical protein	Algae	Unknown	167523038			
Hypothetical protein	Algae	Unknown	167526812			
Hypothetical protein	Algae	Unknown	167538173	~	y	
Hypothetical protein	Algae	Unknown	167527334	~	y	+
Hypothetical protein	Algae	Unknown	167516376	~		+
Hypothetical protein	Algae	Unknown	167516700	~	y	+
Hypothetical protein*	Algae	Unknown	167518201	~	y	+
Hypothetical protein	Algae	Unknown	167516194	~		
Hypothetical protein	Algae	Unknown	167520782			
Hypothetical protein	chromalveolata	Unknown	167519226		y	
Hypothetical protein	Chromalveolata	Unknown	167520778			
Hypothetical protein	Chromalveolates	Unknown	167536443		y	
Hypothetical protein	Chromalveolates	Unknown	167535035			
Hypothetical protein	Chromists	Unknown	167538191	~	y	

Hypothetical protein	Haptophytes	Unknown	167518490	~		
Hypothetical protein	Haptophytes	Unknown	167538306	~		+

****

Maximum likelihood trees for the algal and bacterial genes identified in *Monosiga* (see text and supplemental table for details). Numbers for bootstrap support values above 70% were shown near the branches. The cycle in orange color points to the clade containing sequences of *Monosiga*, bacteria, or/and algae.

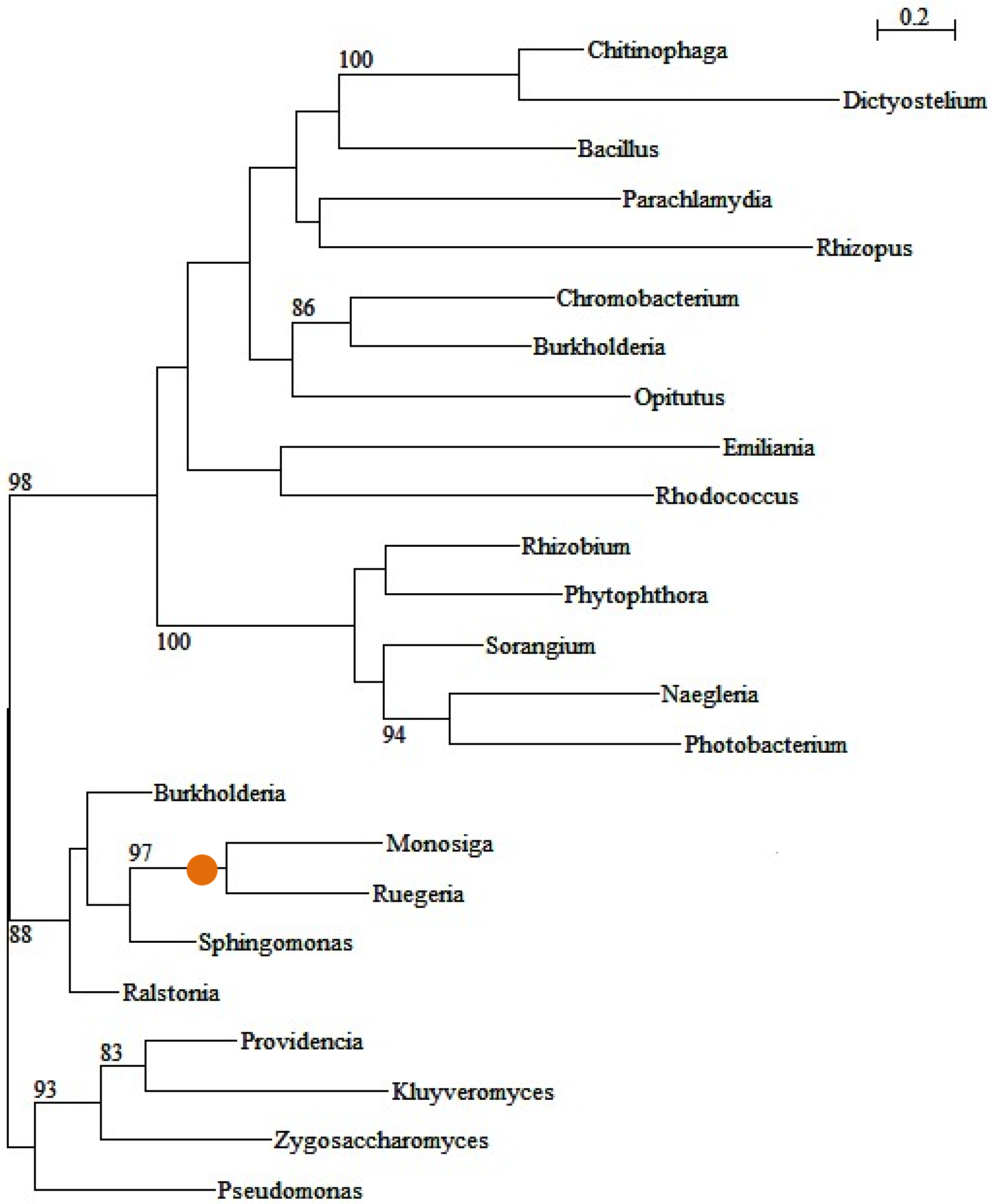

Figure S1. Phylogeny of 2-nitropropane dioxygenase (GI No. 167538159).

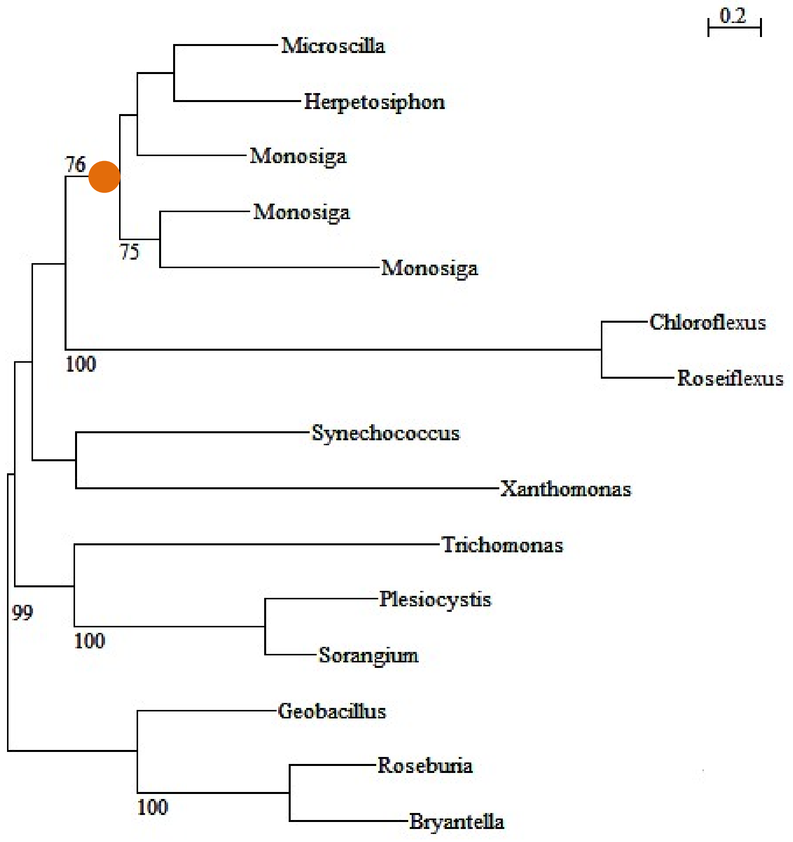

Figure S2. Phylogeny of SCP-like extracellular protein (Gi No. 167534469, 167524445, and 167523453). *Monosiga* only has homologs in bacteria.

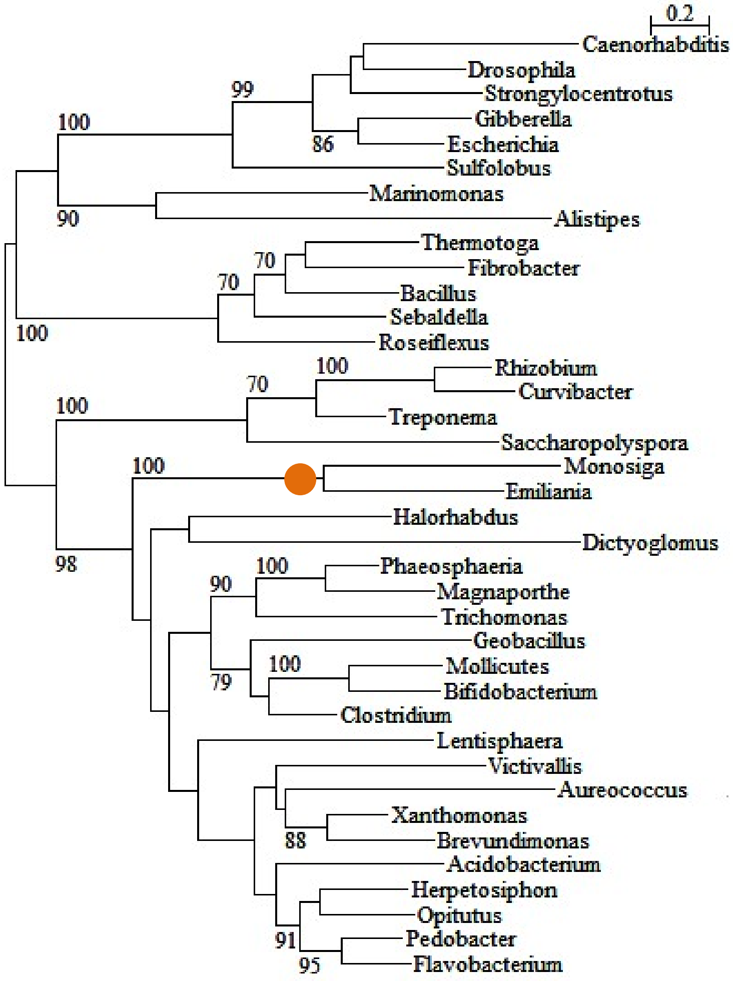

Figure S3. Phylogeny of beta-galactosidase (GI No. 167525643).

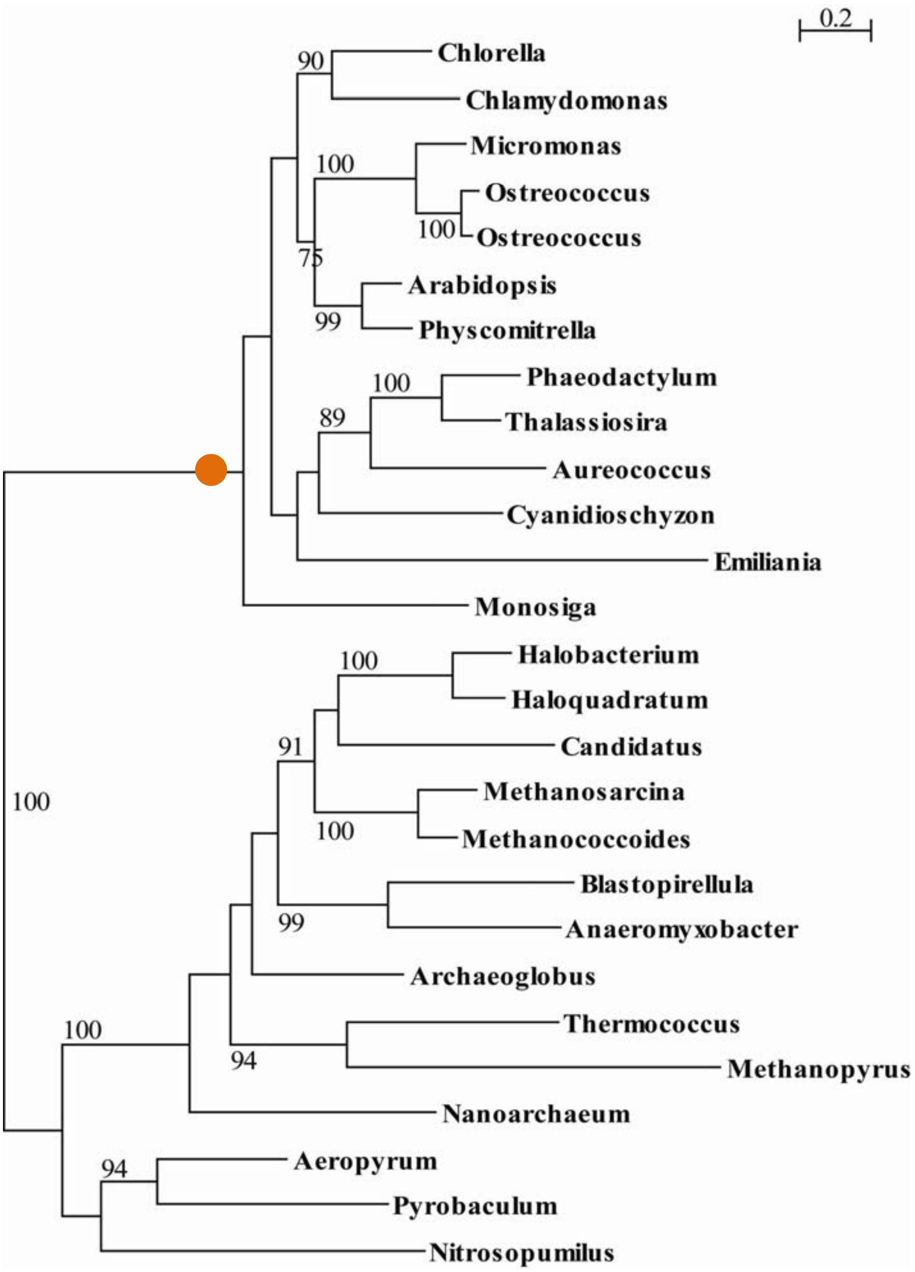

Figure S4. Phylogeny of DNA topoisomerase VI subunit B (GI No. 167526605).

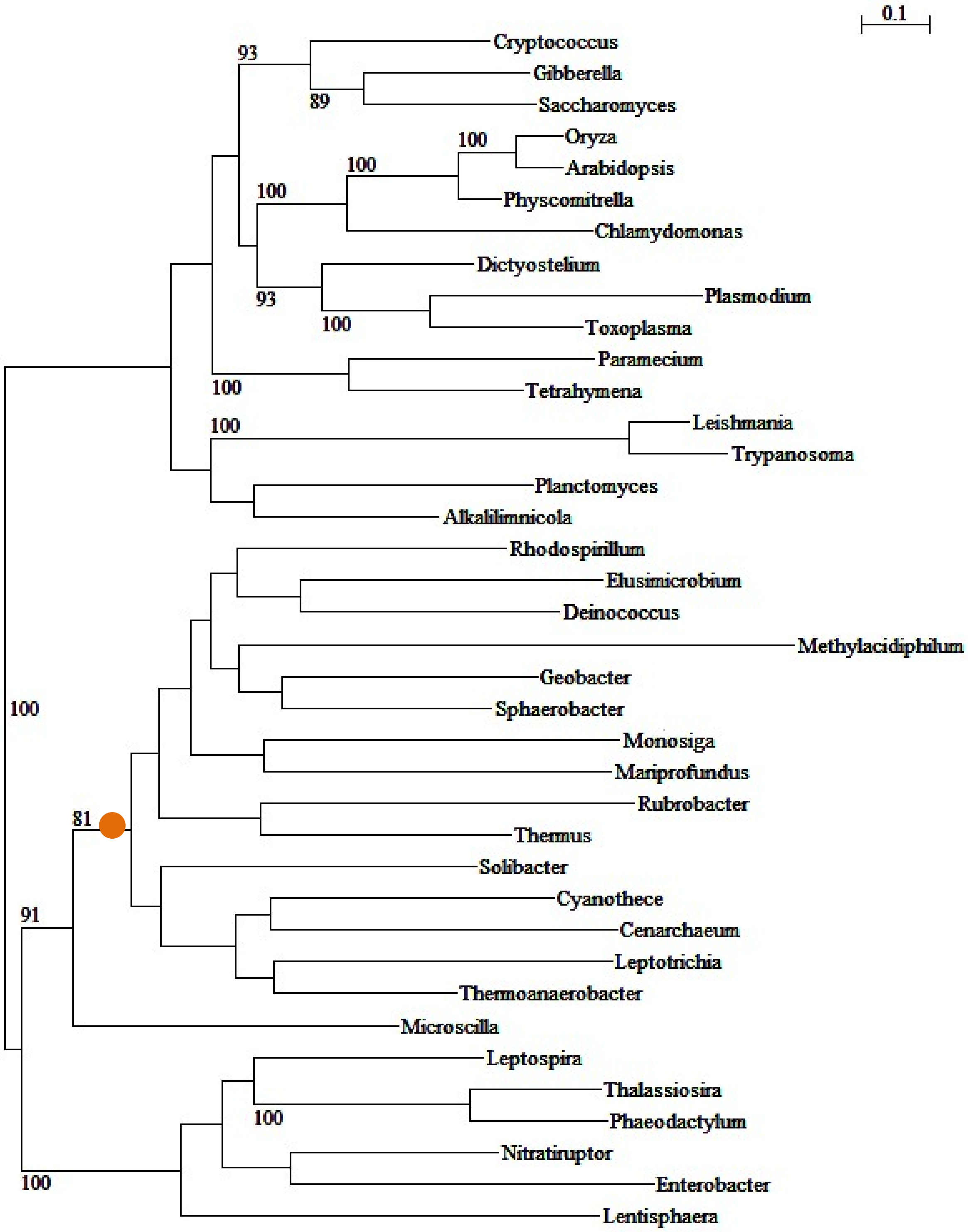

Figure S5. Phylogeny of phosphoenolpyruvate carboxykinase (GI No. 167519713).

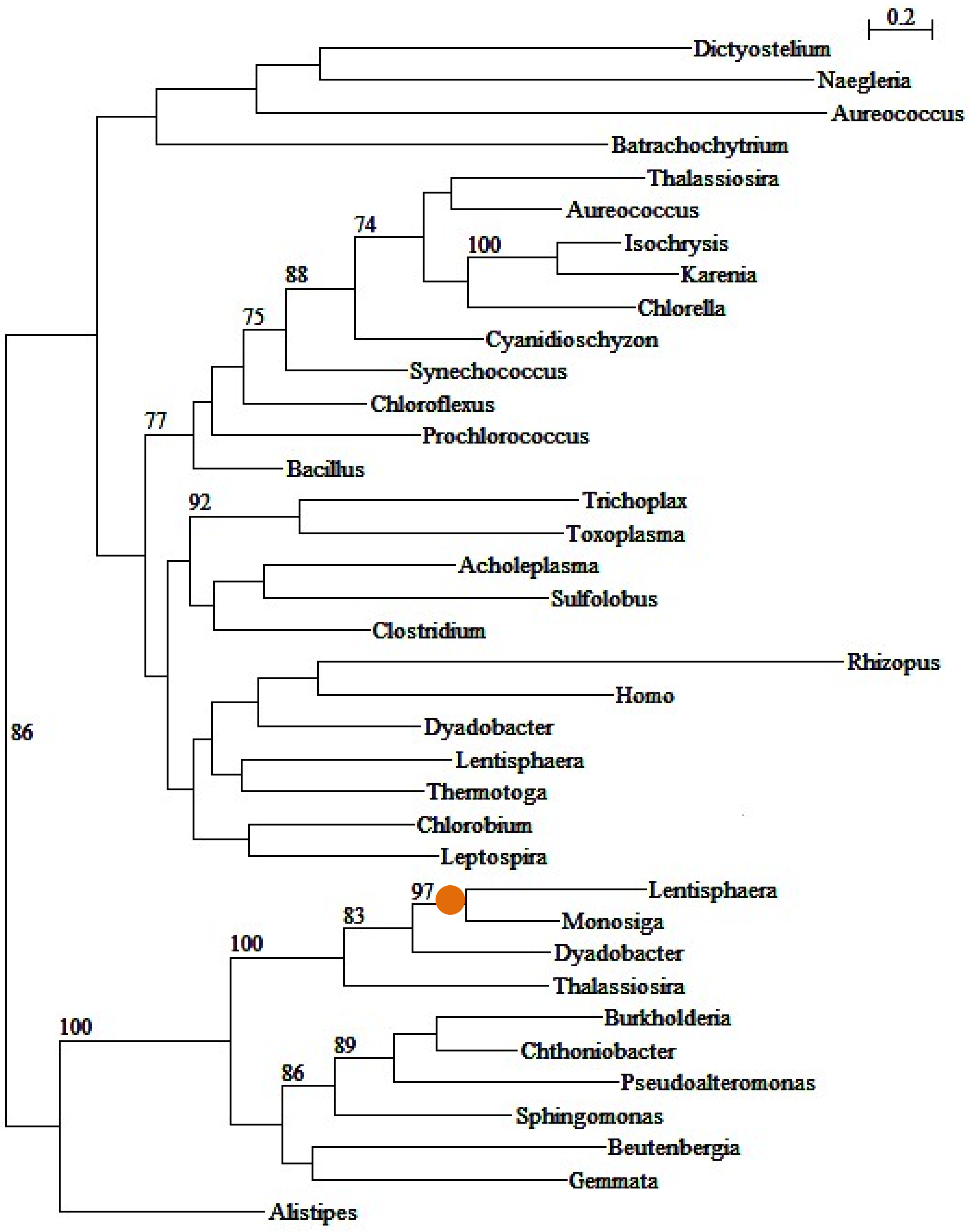

Figure S6. Phylogeny of short-chain dehydrogenase/reductase (GI No. 167515652).

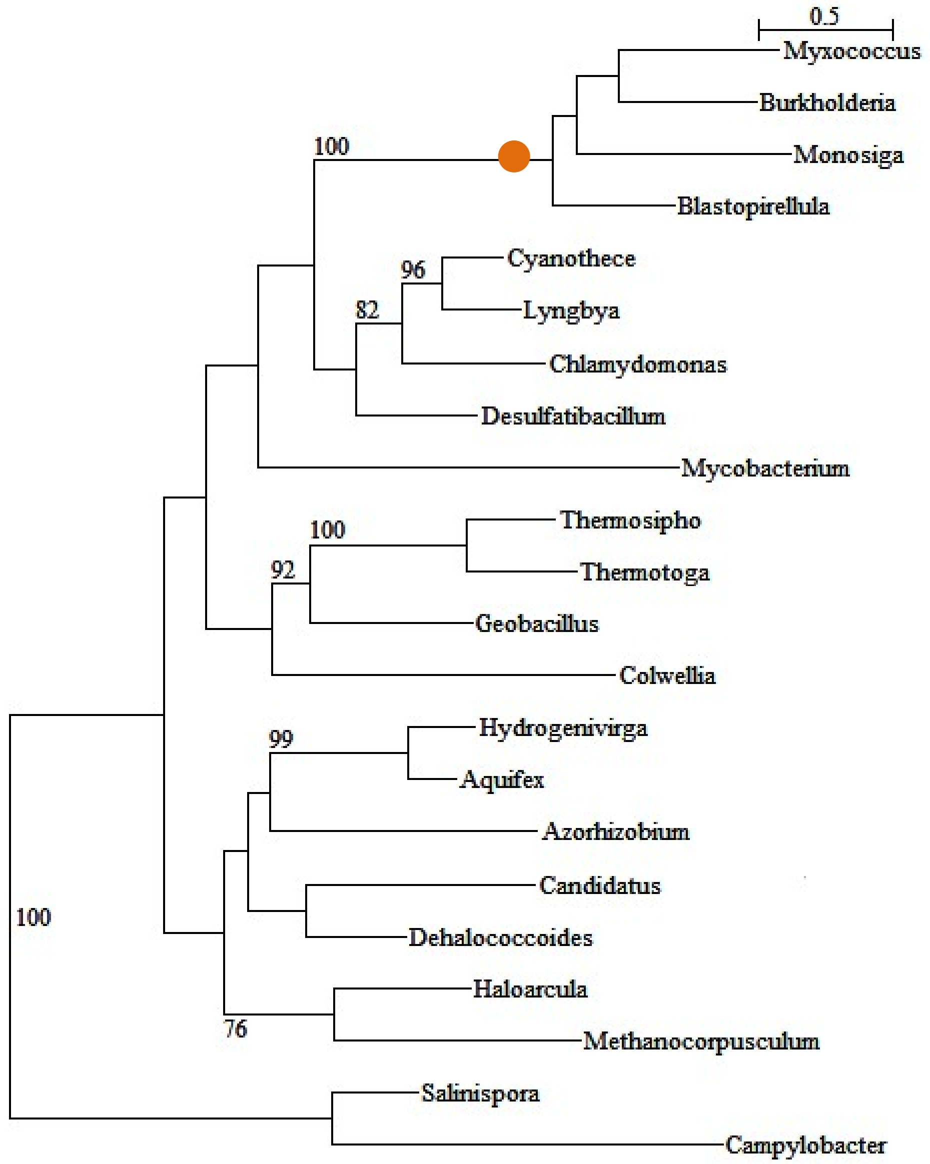

Figure S7. Phylogeny of one hypothetical protein (GI No. 167518772).

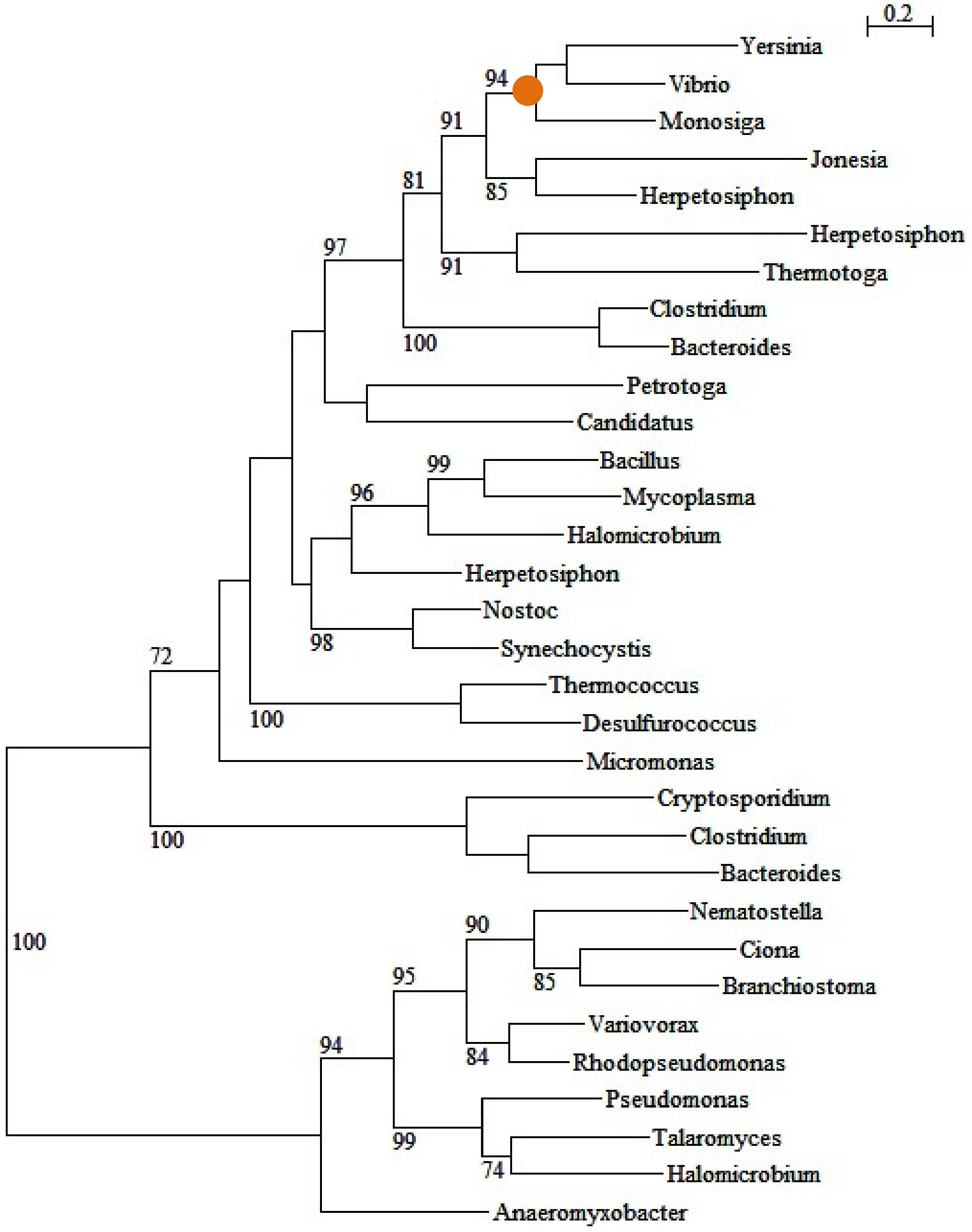

Figure S8. Phylogeny of maltodextrin glucosidase (GI No. 167521117).

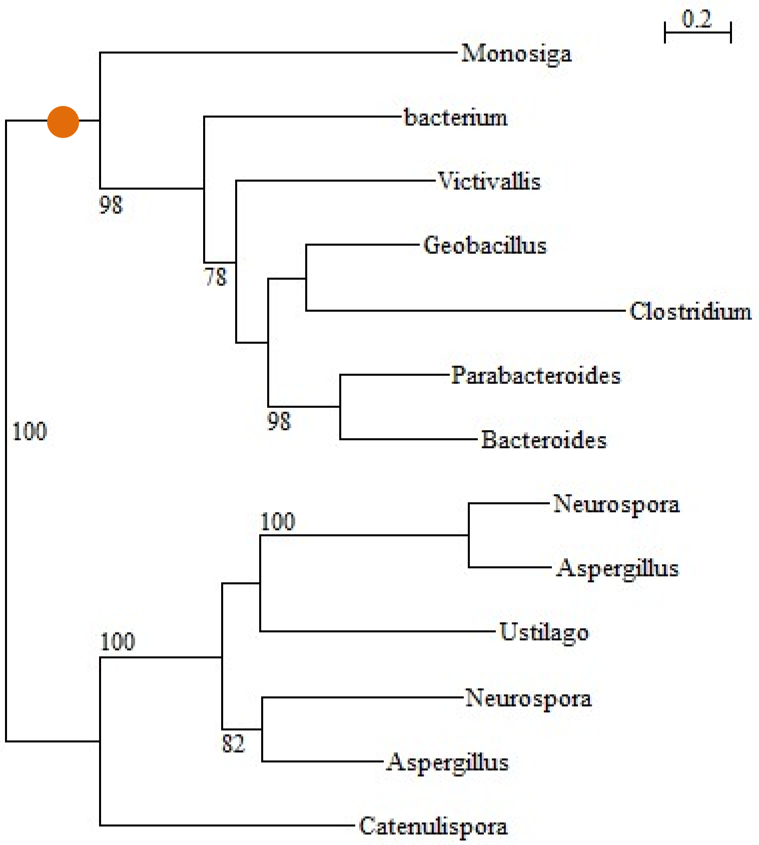

Figure S9. Phylogeny of glutaminase A (GI No. 167522419).

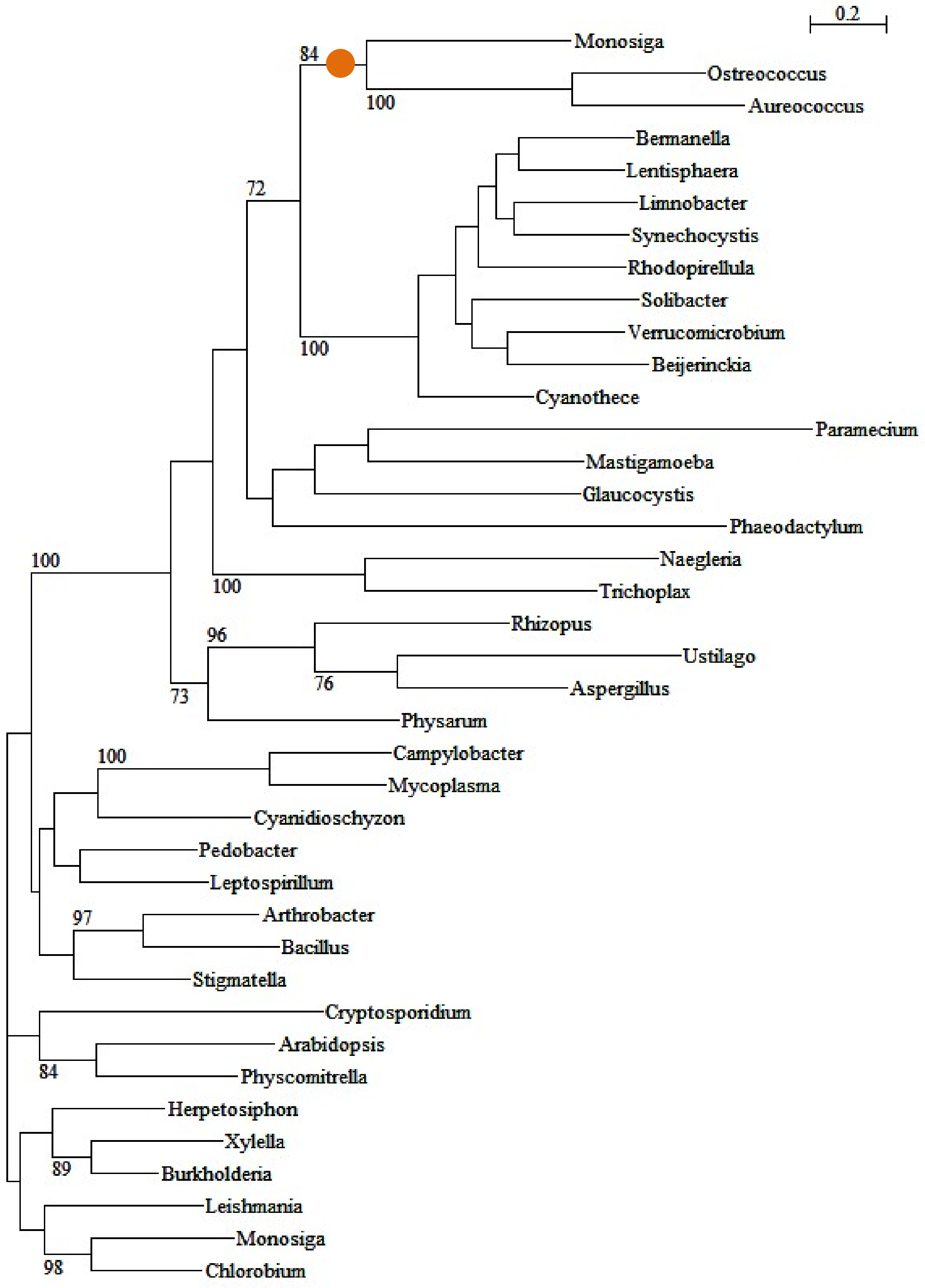

Figure S10. Phylogeny of alcohol dehydrogenase (GI No. 167526433).

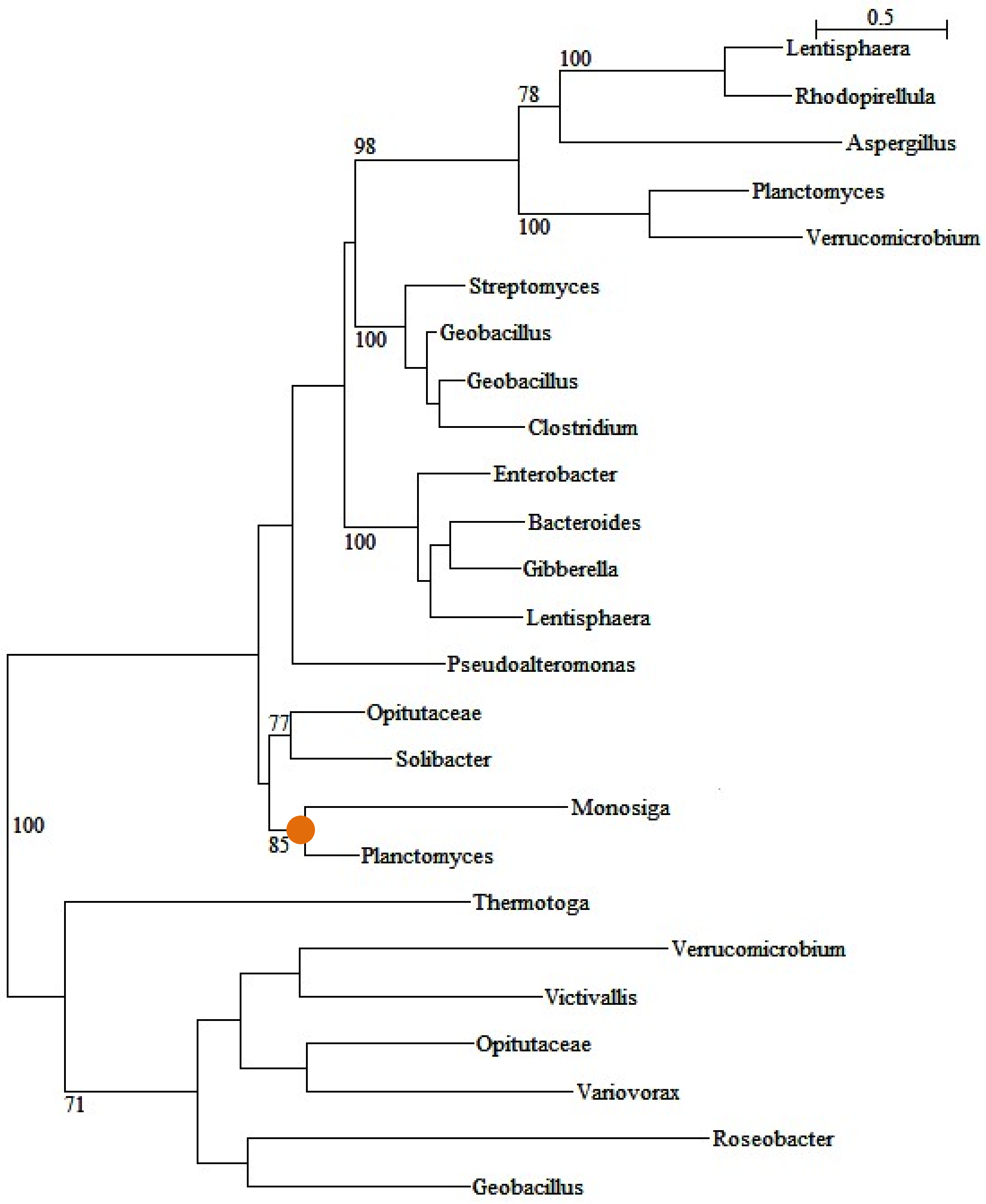

Figure S11. Phylogeny of fumarate reductase/succinate dehydrogenase (GI No. 167526295).

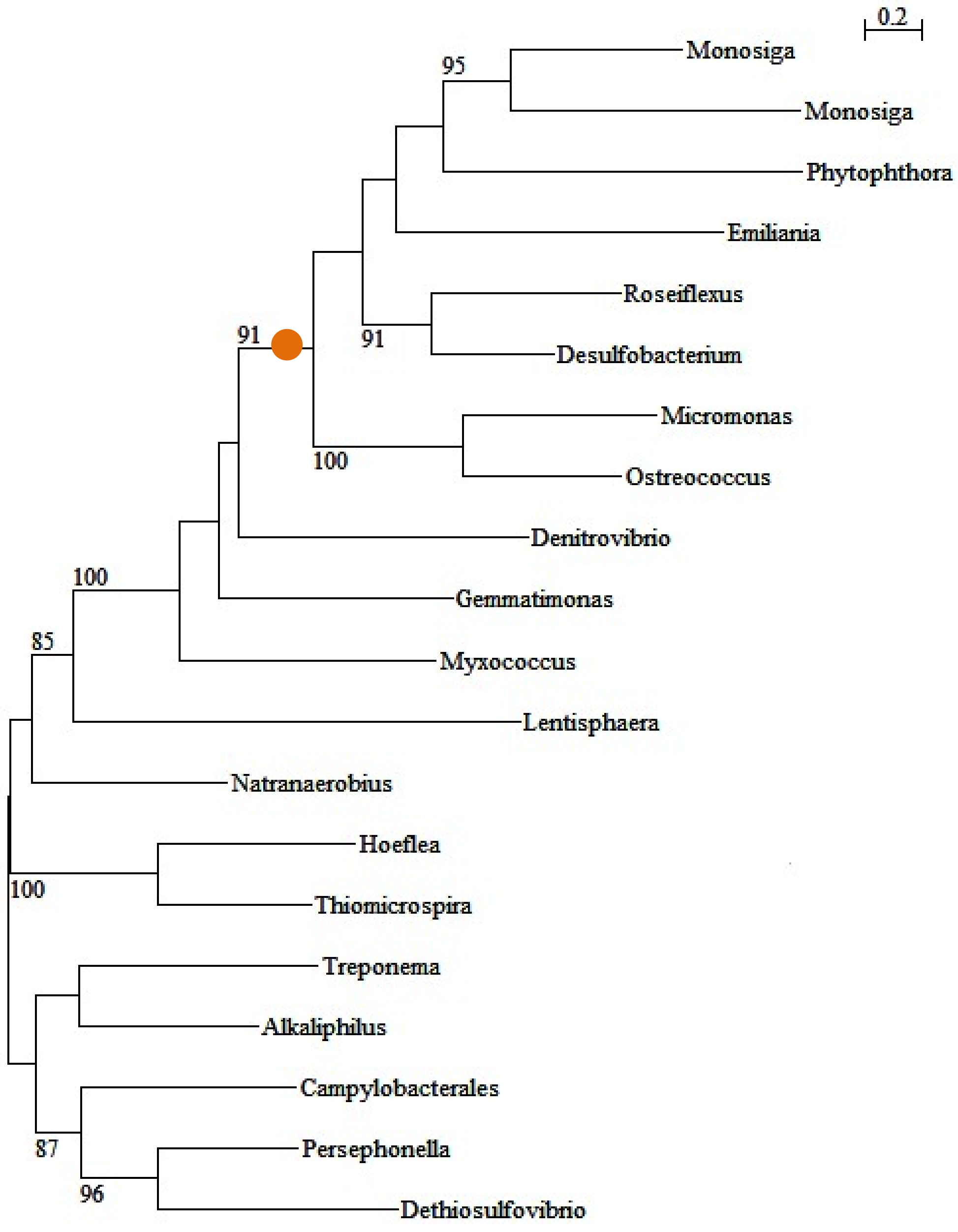

Figure S12. Phylogeny of TrkA (Na^+/H^+ antiporter) (Gi No. 167526916 and 167537773).

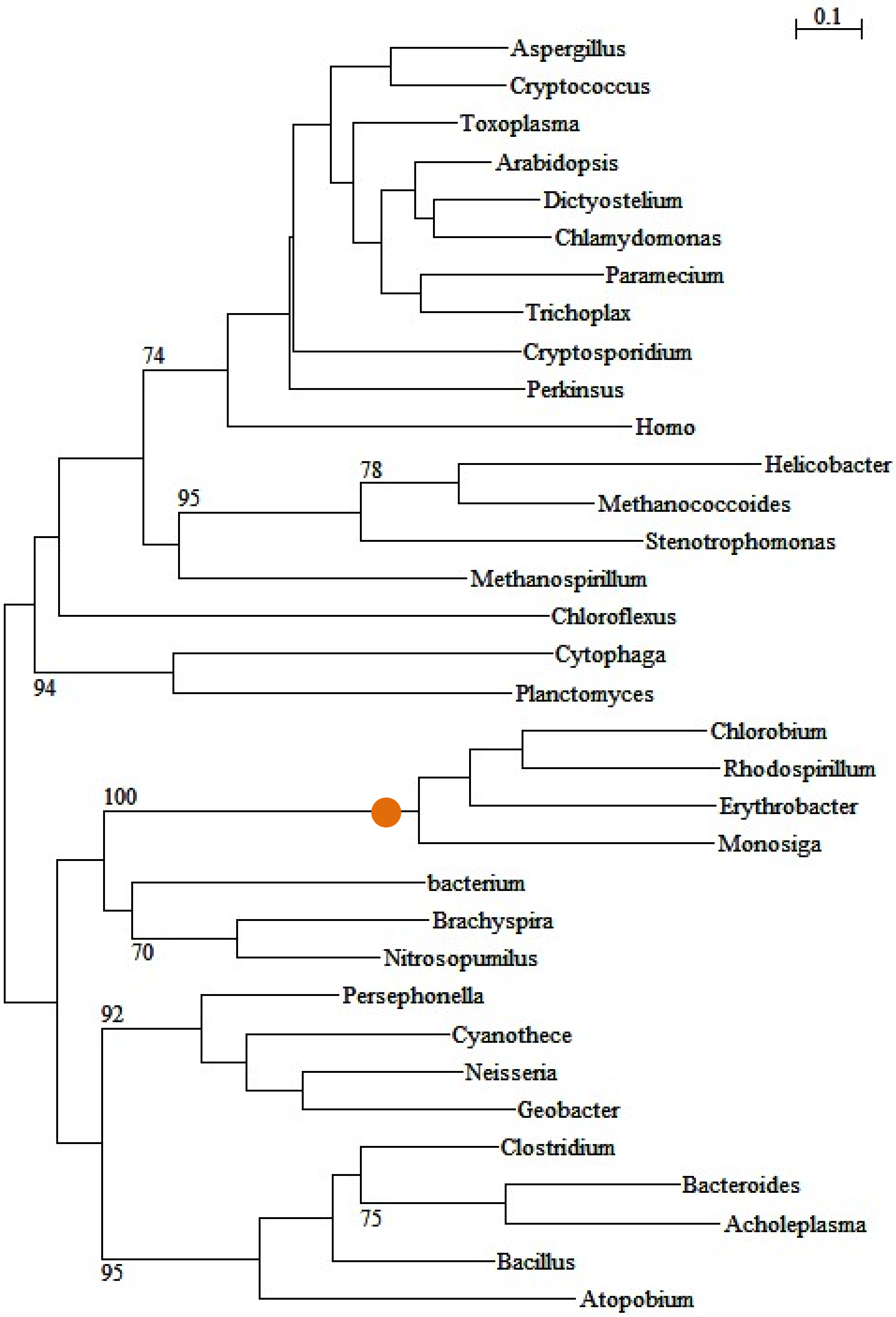

Figure S13. Phylogeny of peptidyl-prolyl cis-trans isomerase (GI No. 167527426).

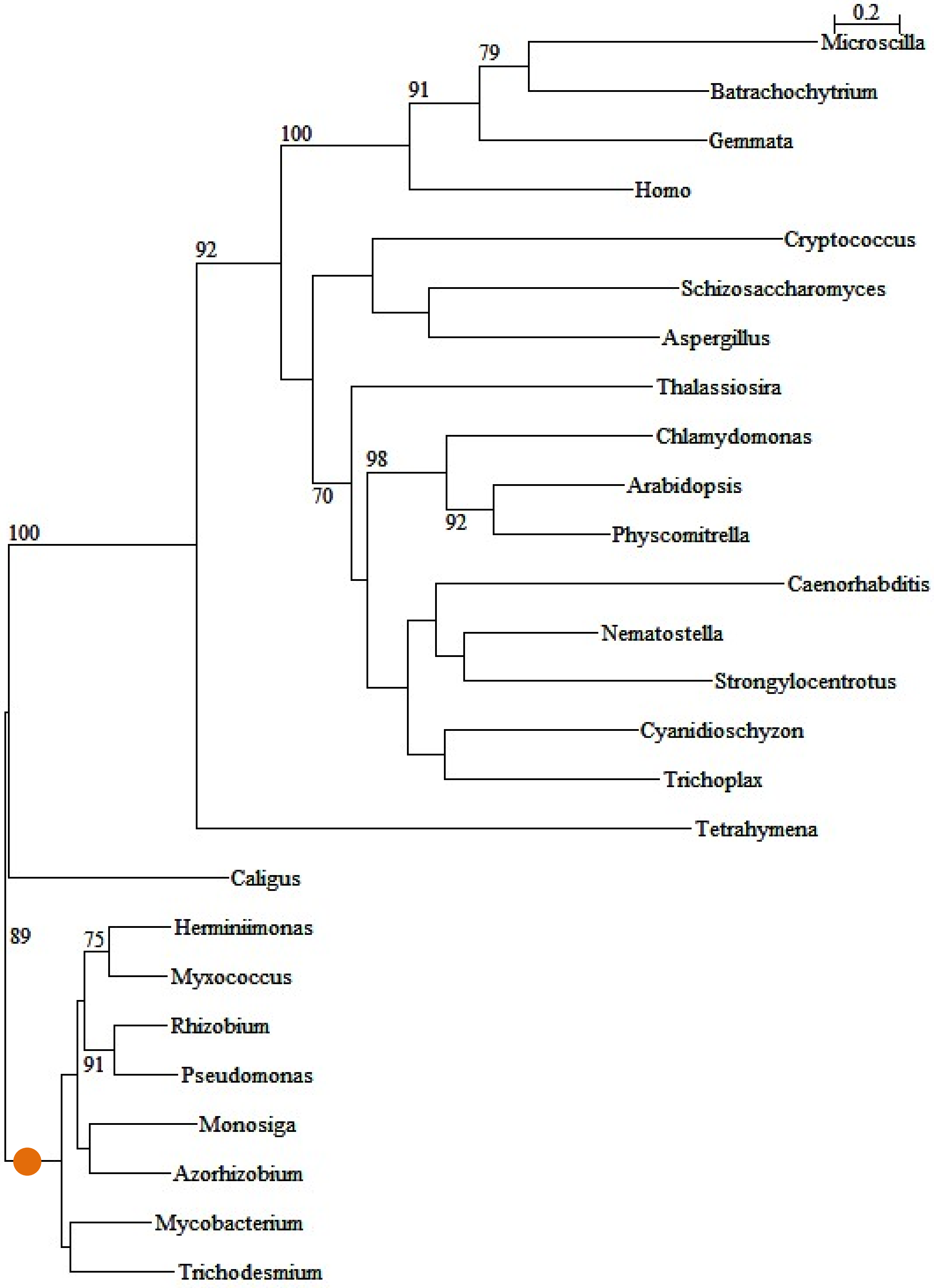

Figure S14. Phylogeny of flavin-containing monooxygenase (GI No. 167534098).

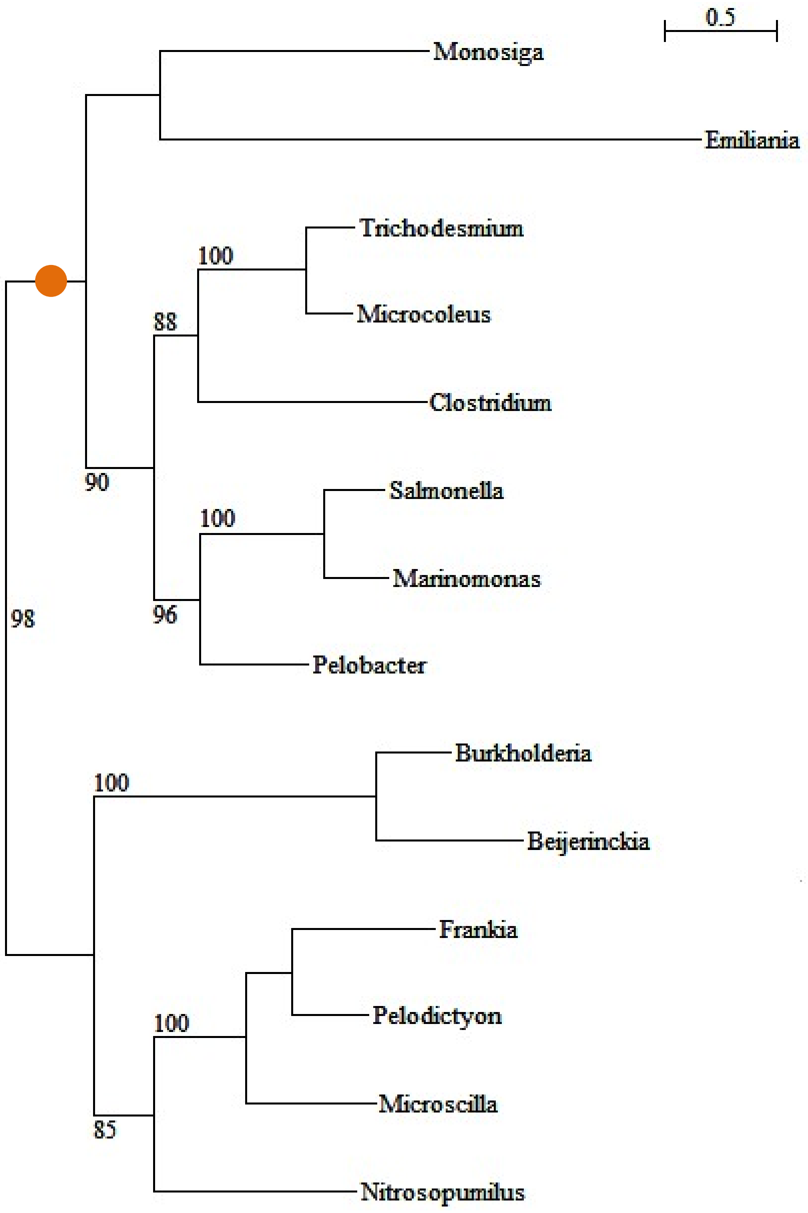

Figure S15. Phylogeny of regulatory protein CII (GI No. 167536551).

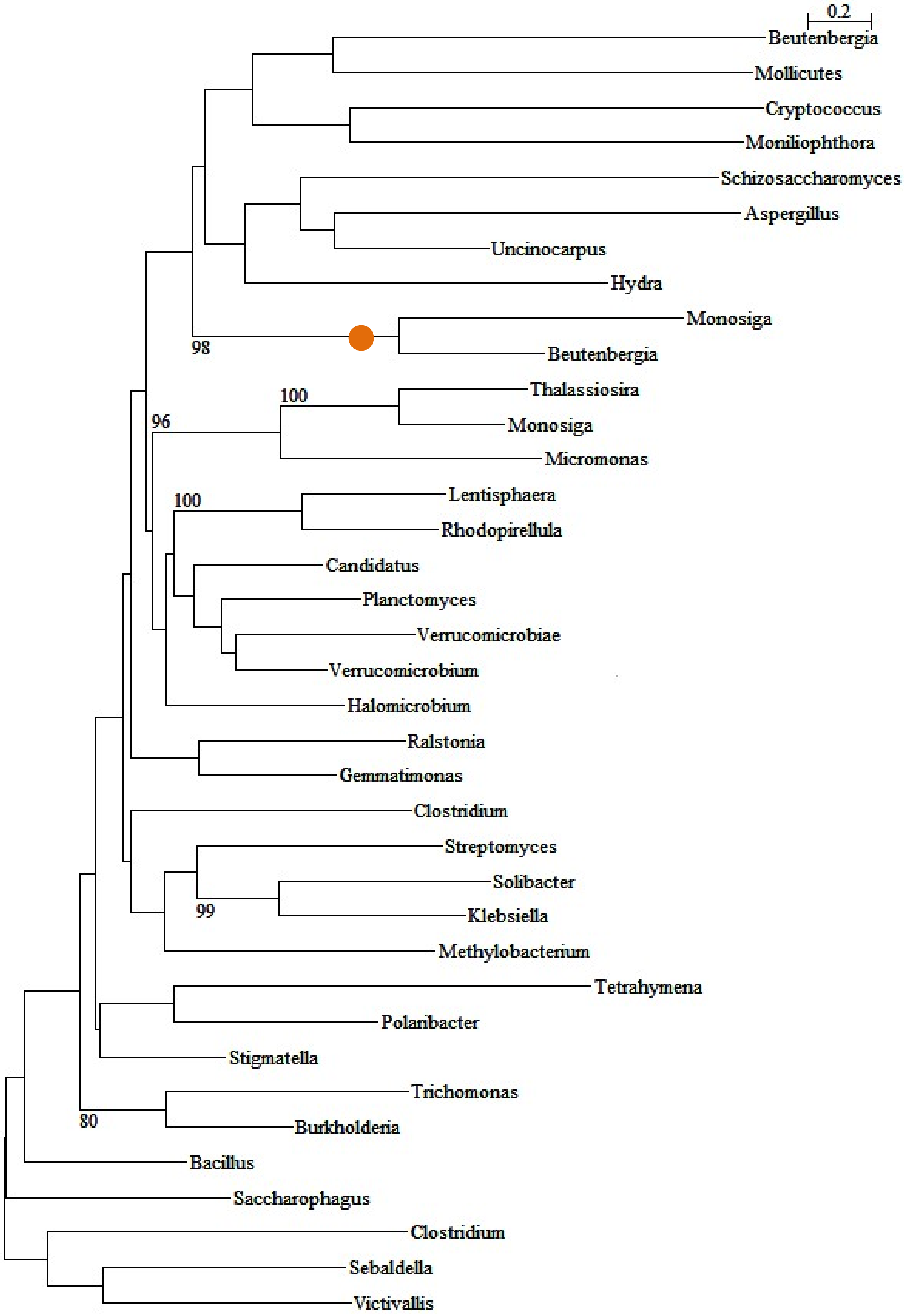

Figure S16. Phylogeny of one hypothetical protein (GI No. 167537457).

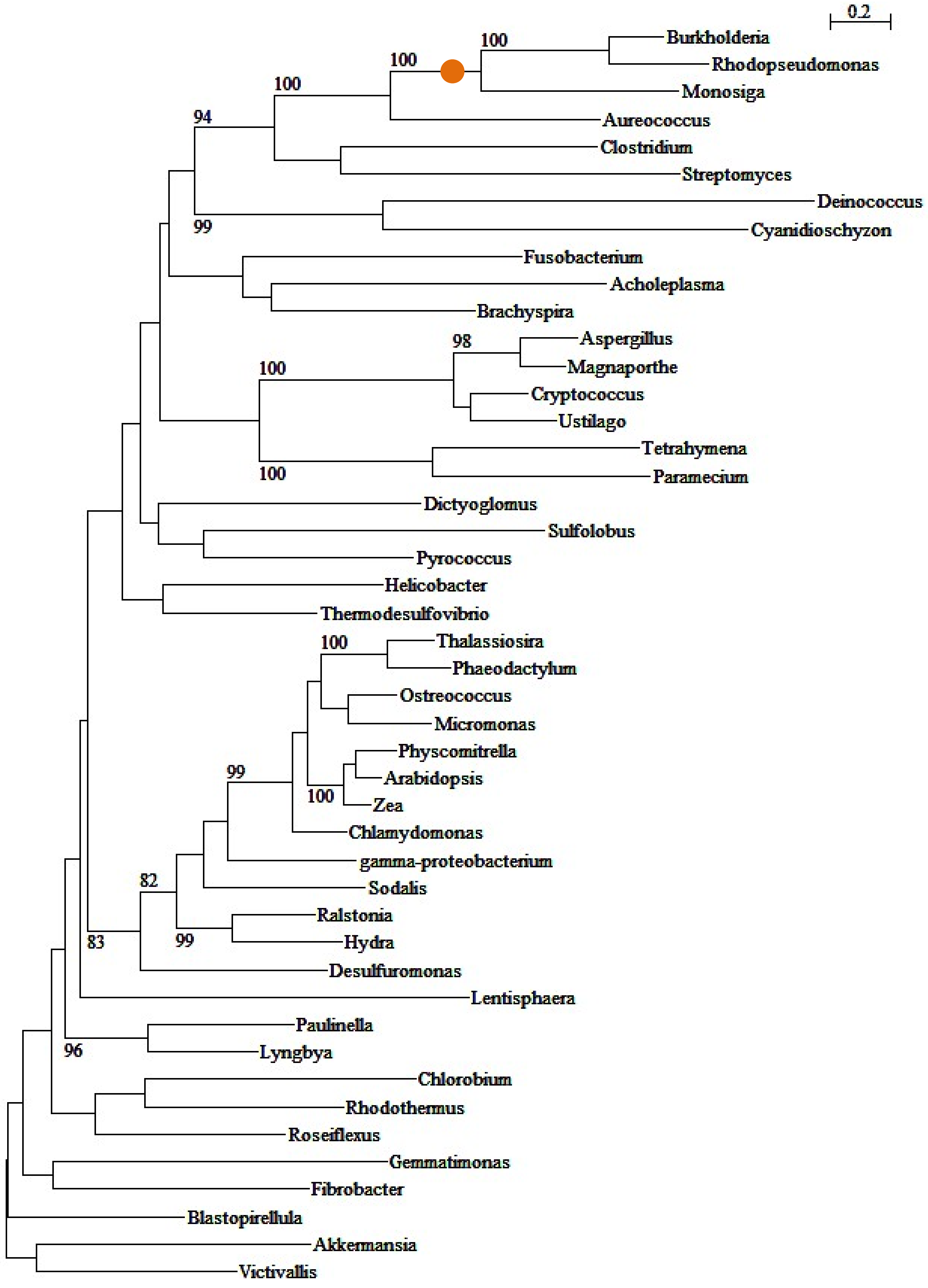

Figure S17. Phylogeny of 3-dehydroquinate synthase (GI No. 167537749).

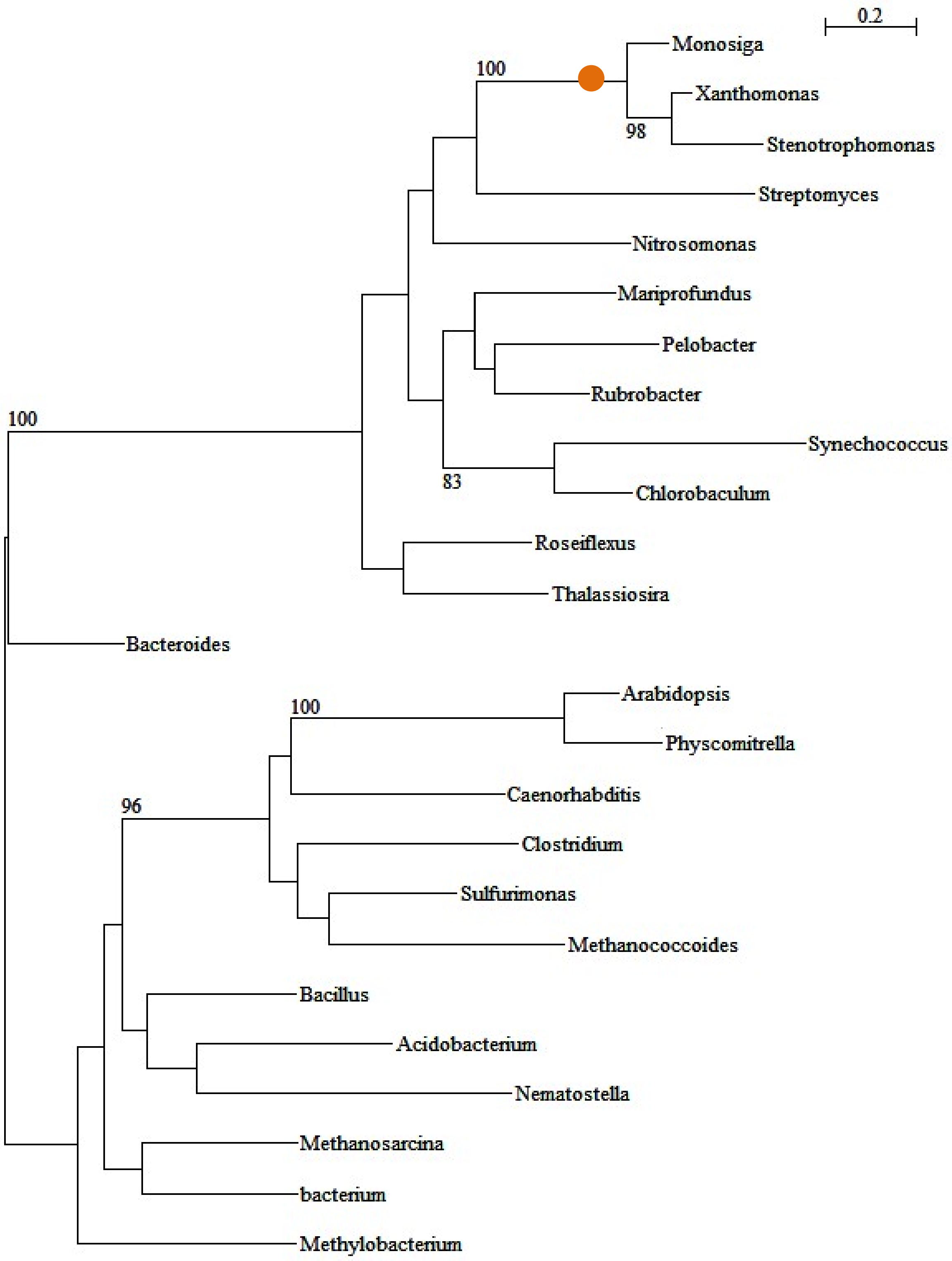

Figure S18. Phylogeny of CMP/dCMP deaminase (GI No. 167538365).

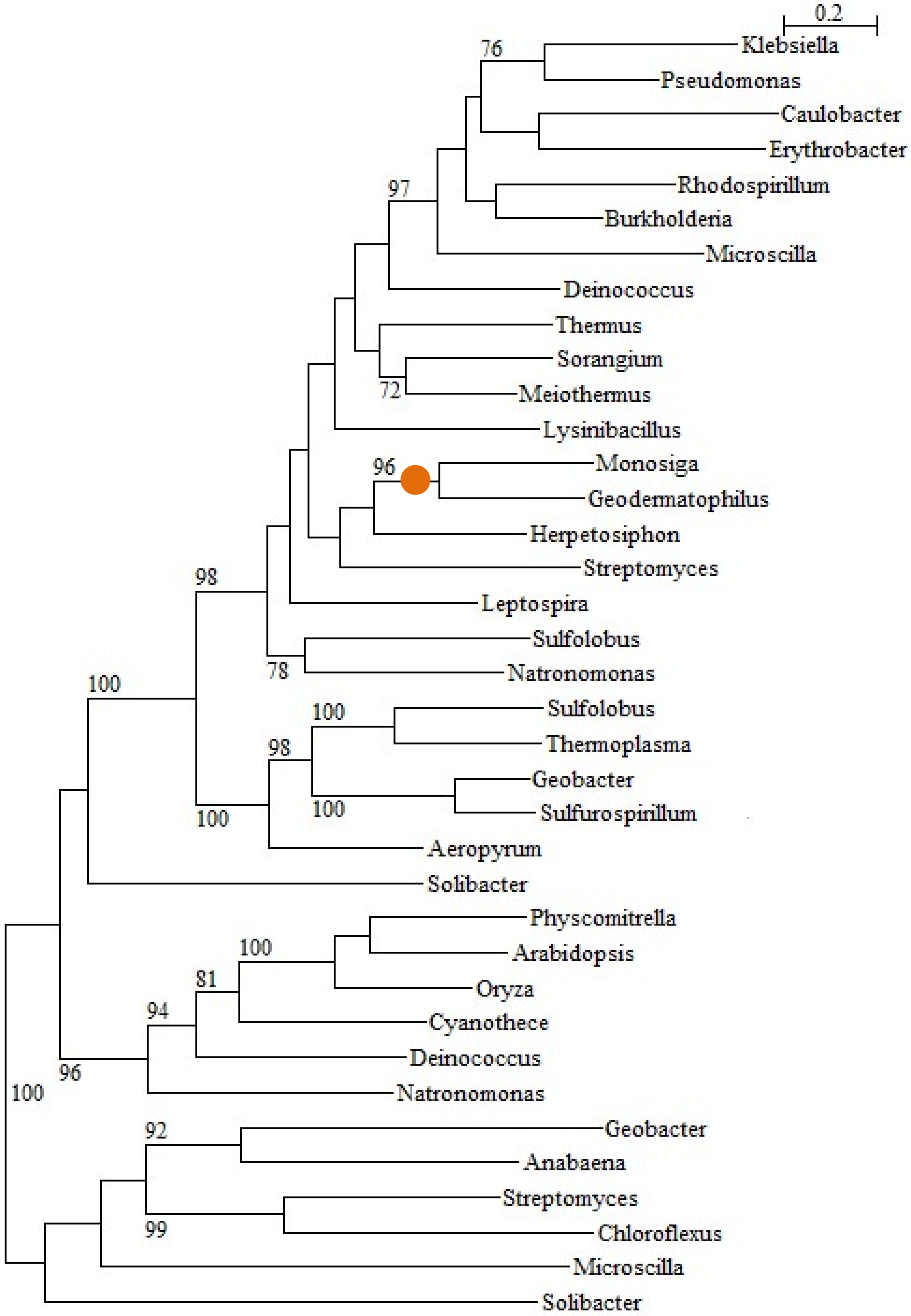

Figure S19. Phylogeny of fatty-acyl-CoA synthetase (GI No. 167516368).

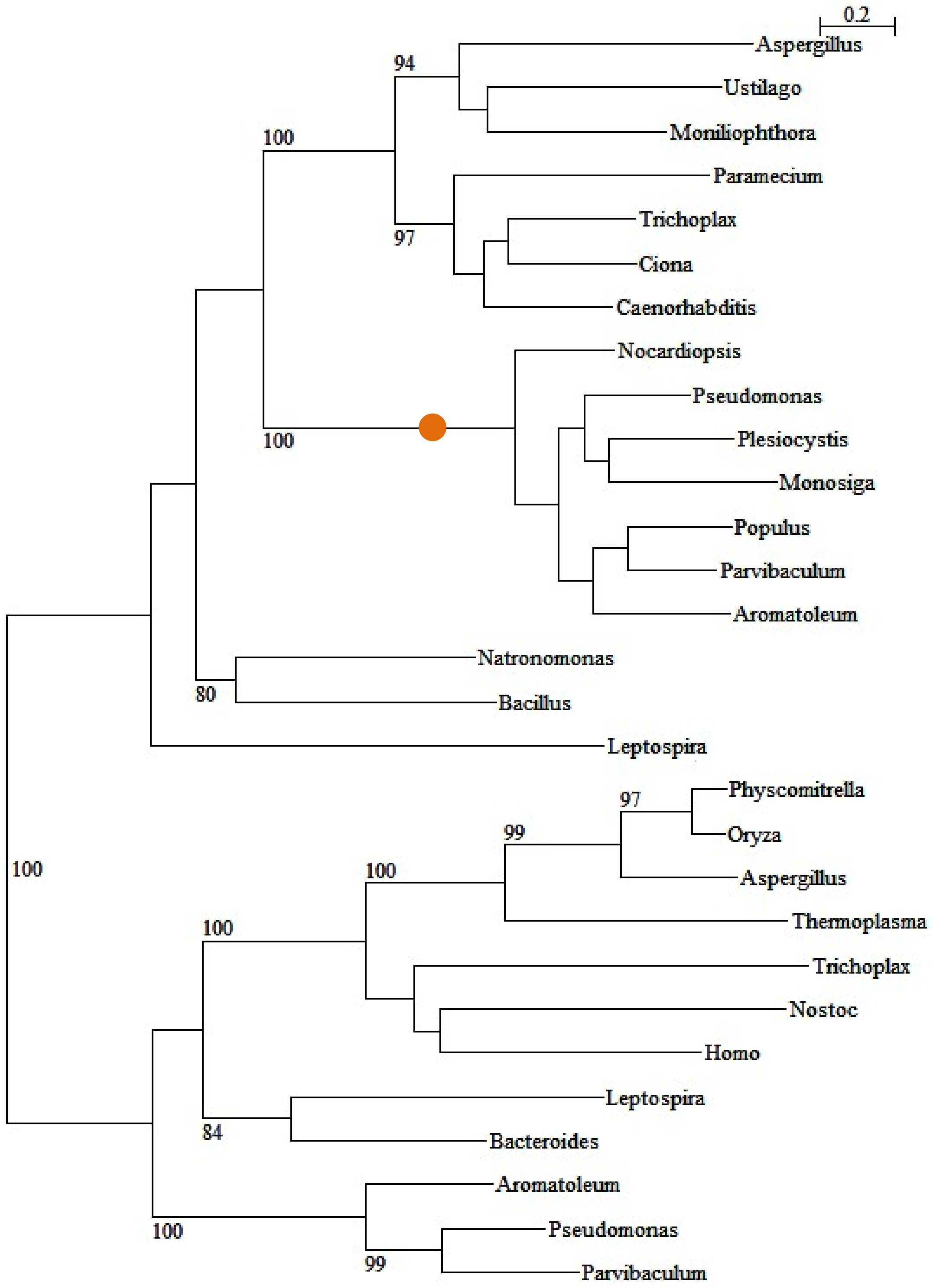

Figure S20. Phylogeny of acyl-CoA dehydrogenase (GI No. 167518089).

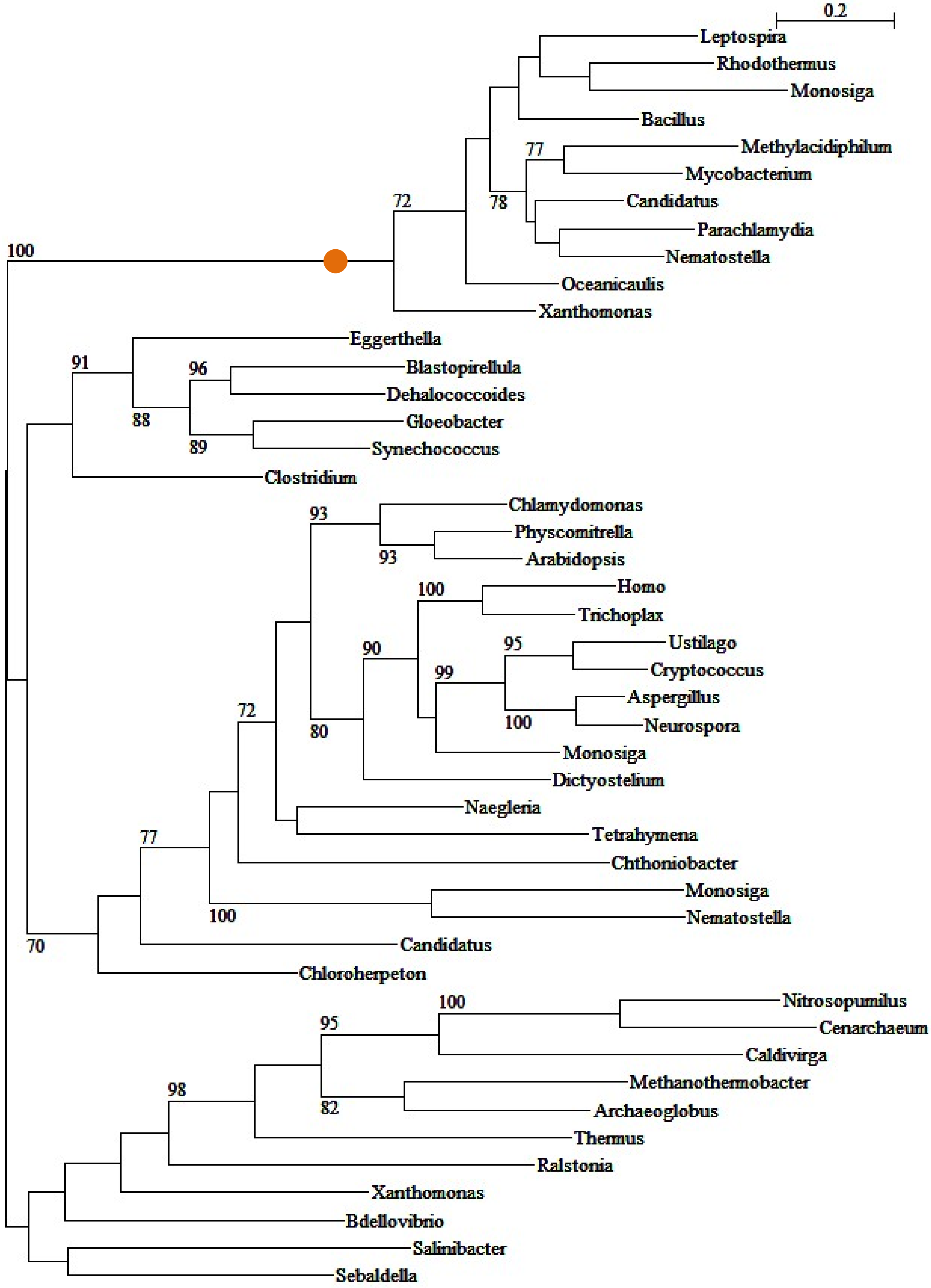

Figure S21. Phylogeny of isocitrate dehydrogenase (NAD^+) (GI No. 167518099).

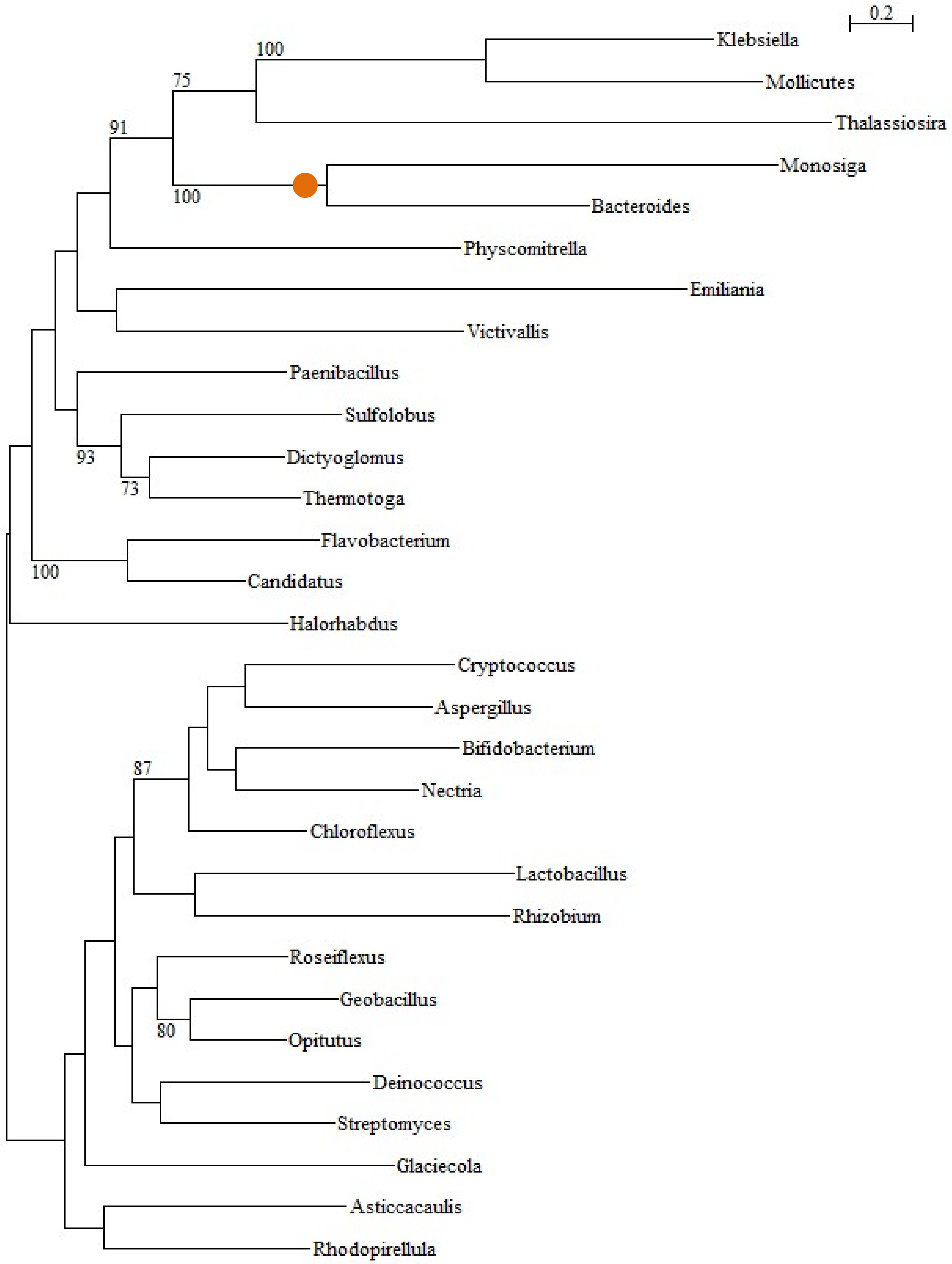

Figure S22. Phylogeny of alpha-rhamnosidase (NAD^+) (GI No. 167524457).

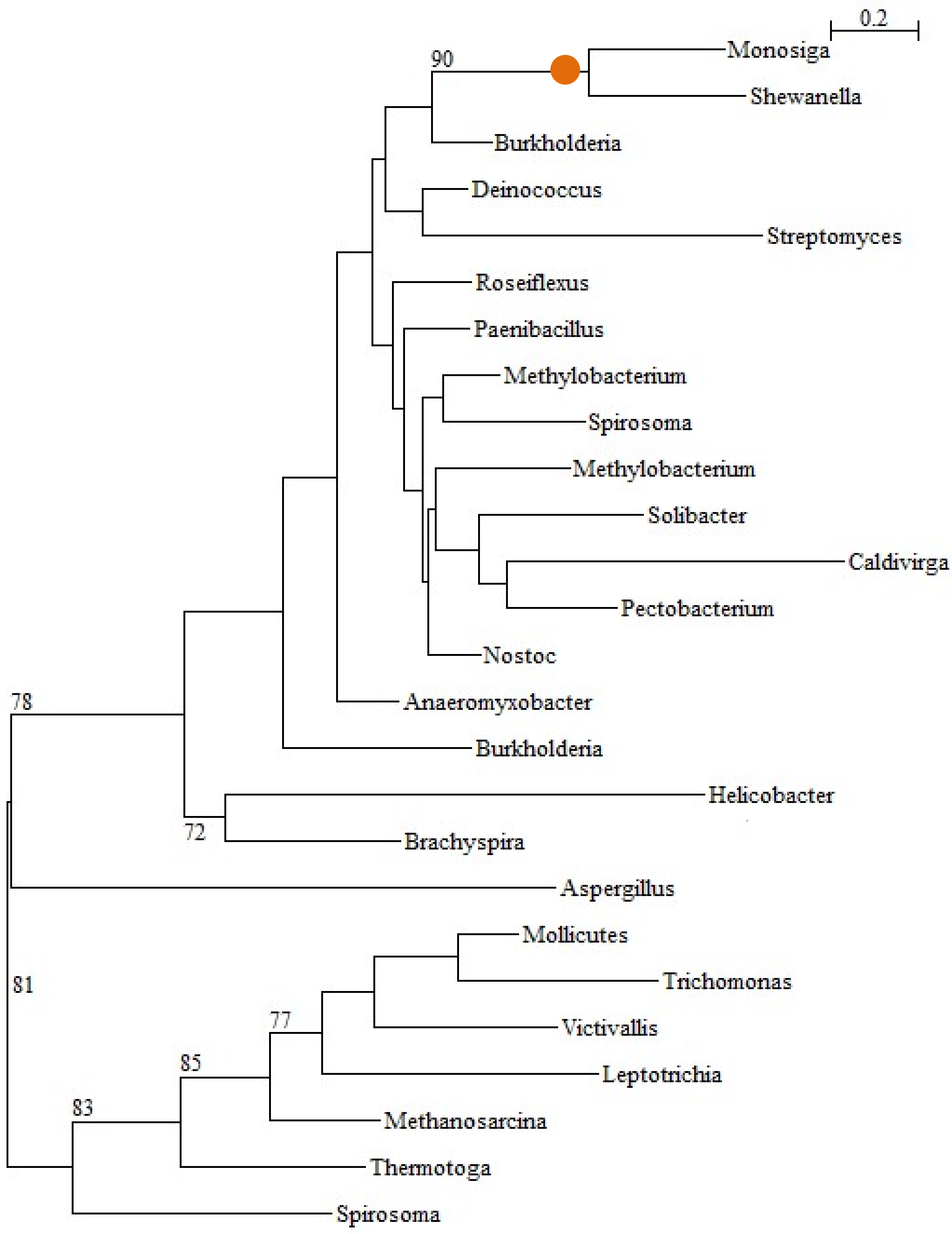

Figure S23. Phylogeny of cupin 2 domain-containing protein (GI No. 167527452).

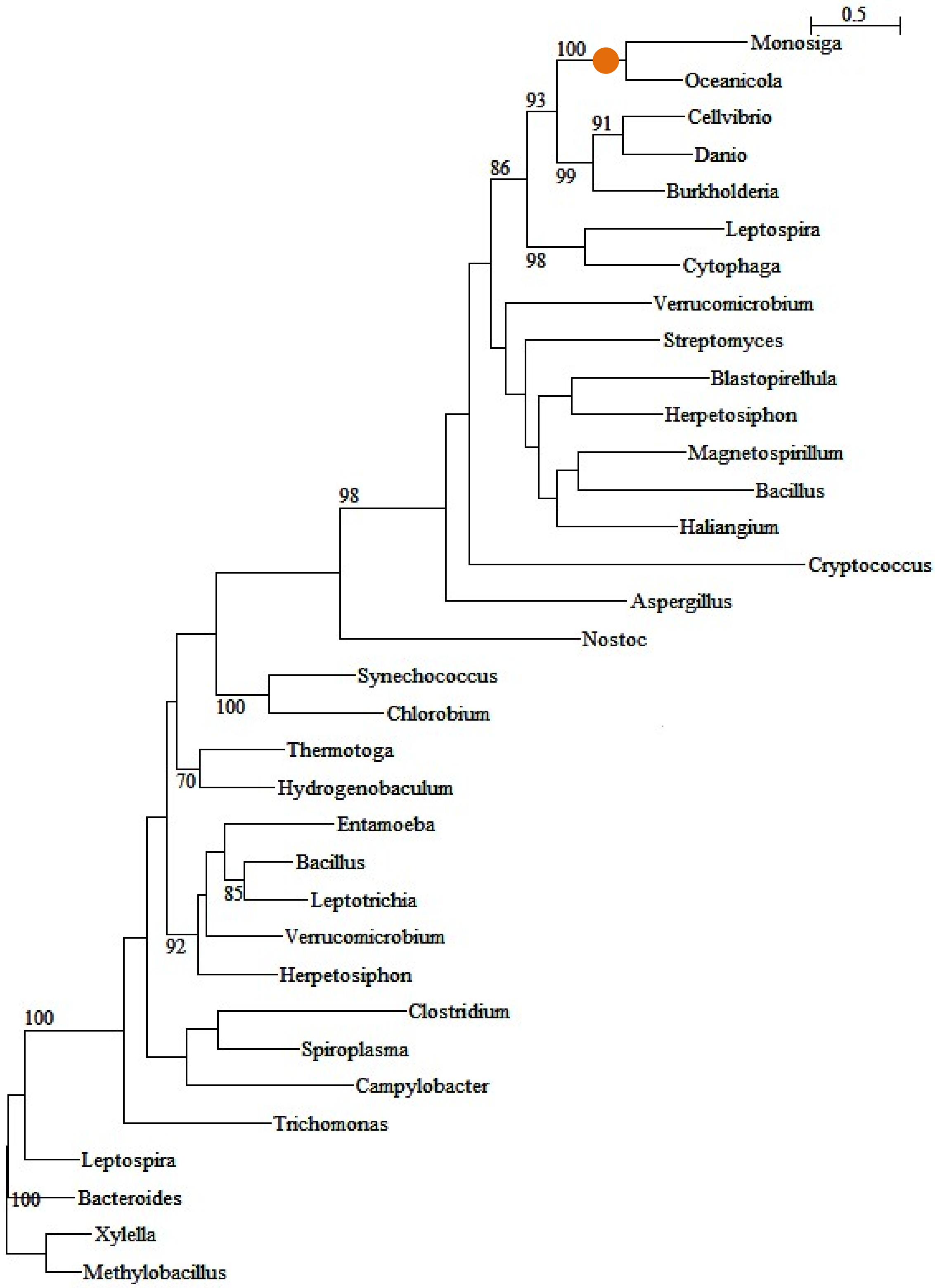

Figure S24. Phylogeny of FAD-dependent pyridine nucleotide-disulphide oxidoreductase (GI No. 167523218).

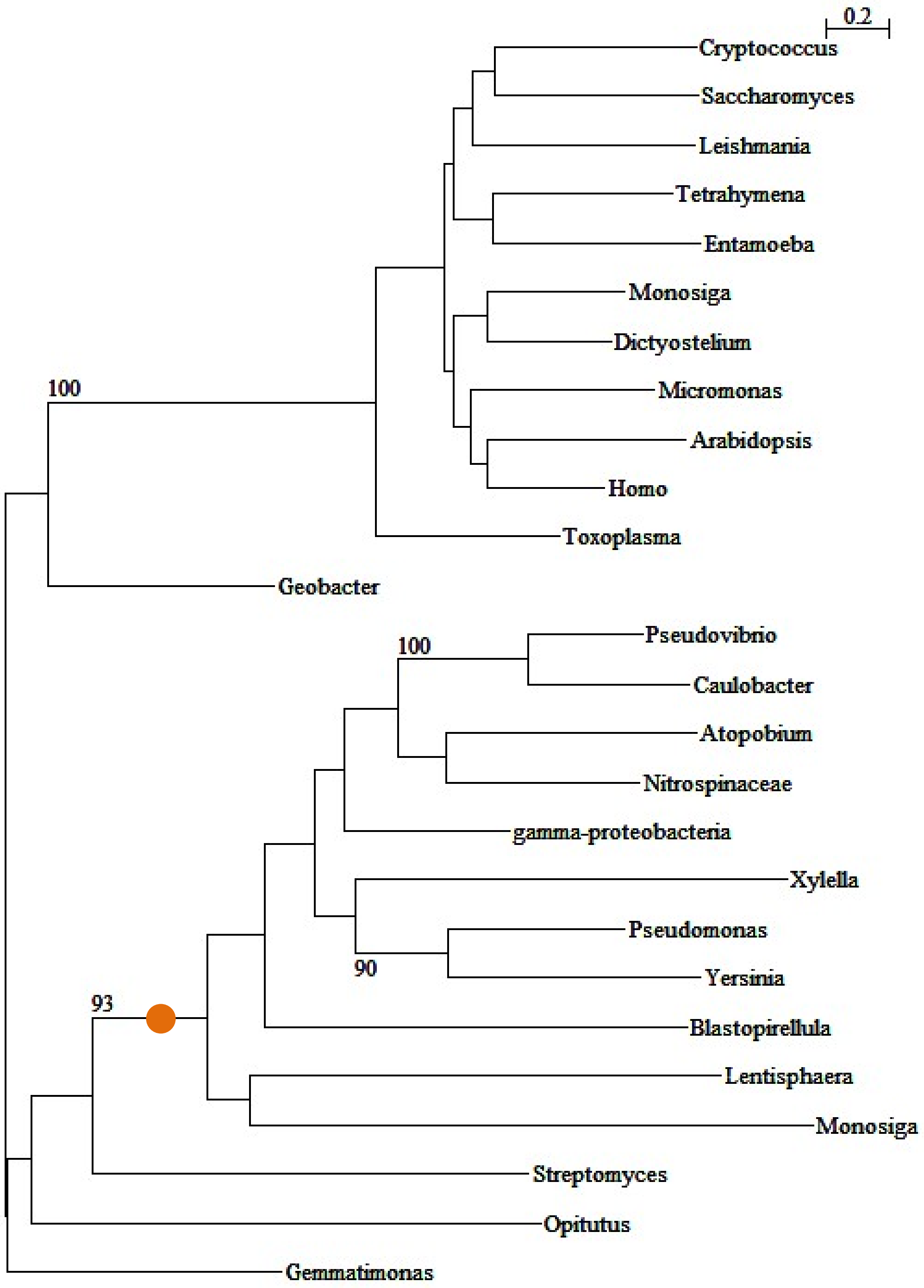

Figure S25. Phylogeny of ribonuclease D (GI No. 167533742).

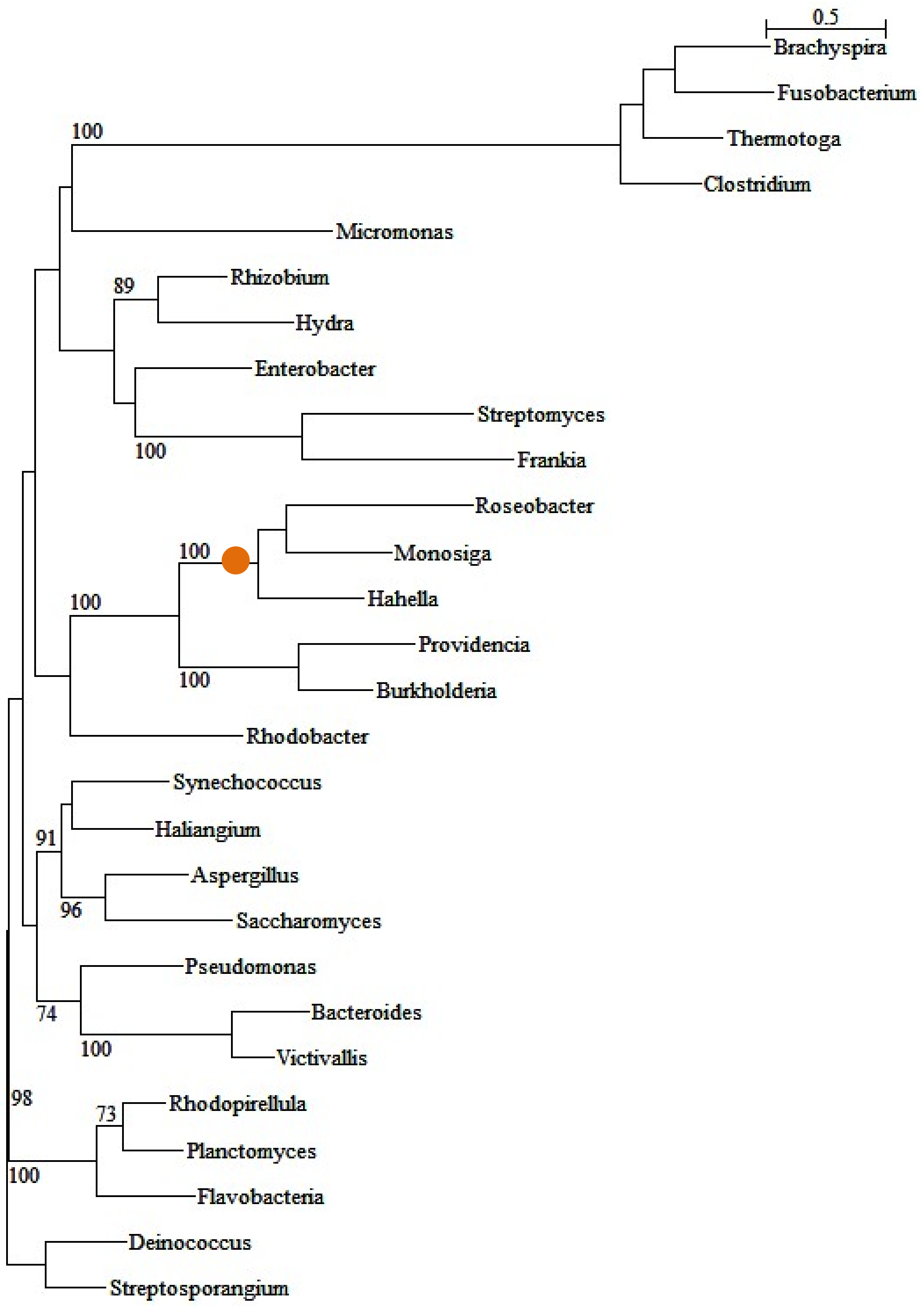

Figure S26. Phylogeny of mannitol-1-phosphate/altronate dehydrogenase (GI No. 167537946).

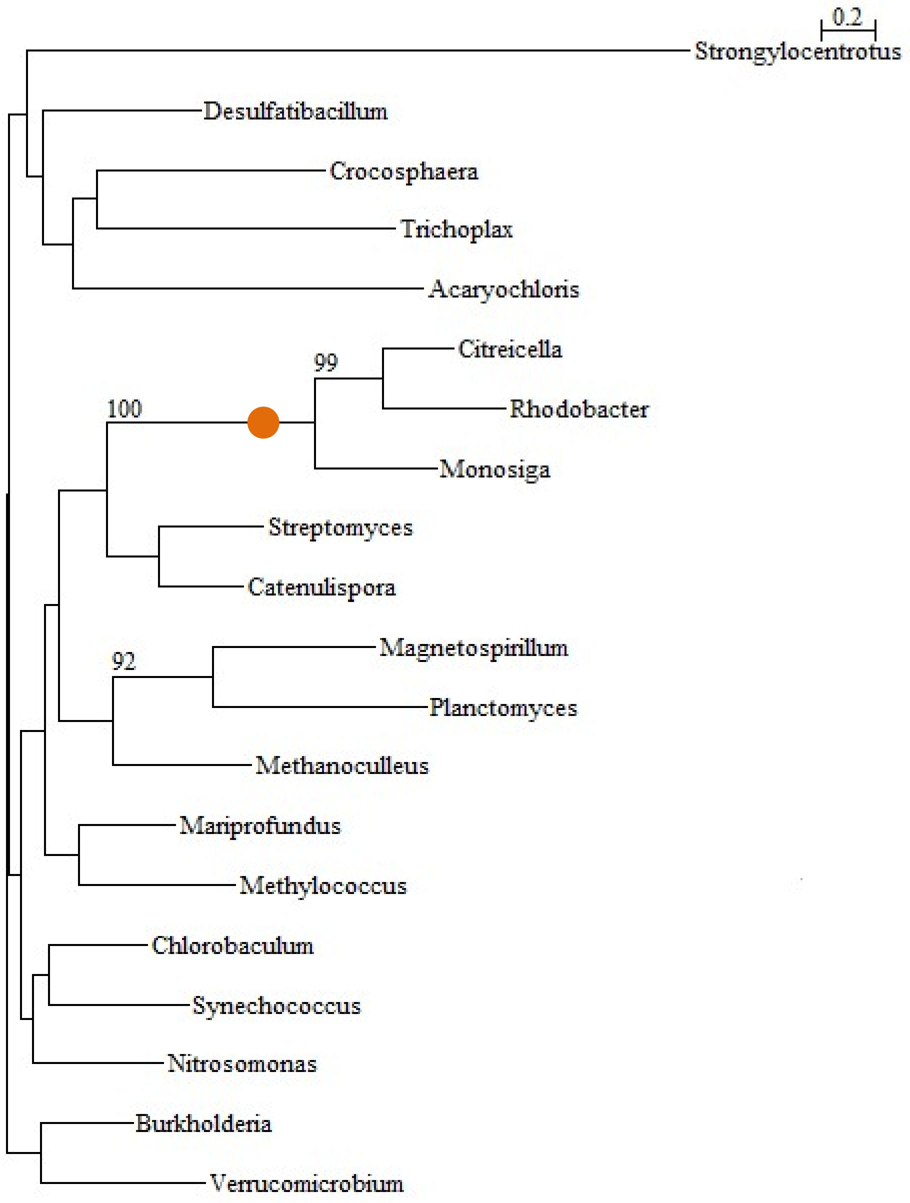

Figure S27. Phylogeny of acetyltransferase (GI No. 167525825).

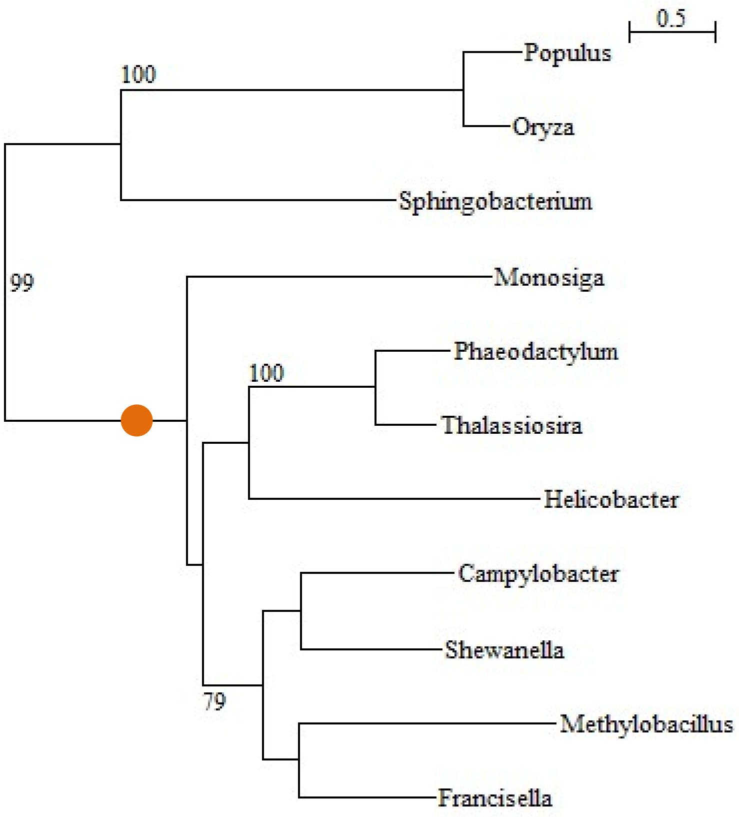

Figure S28. Phylogeny of one hypothetical protein (GI No. 167534537).

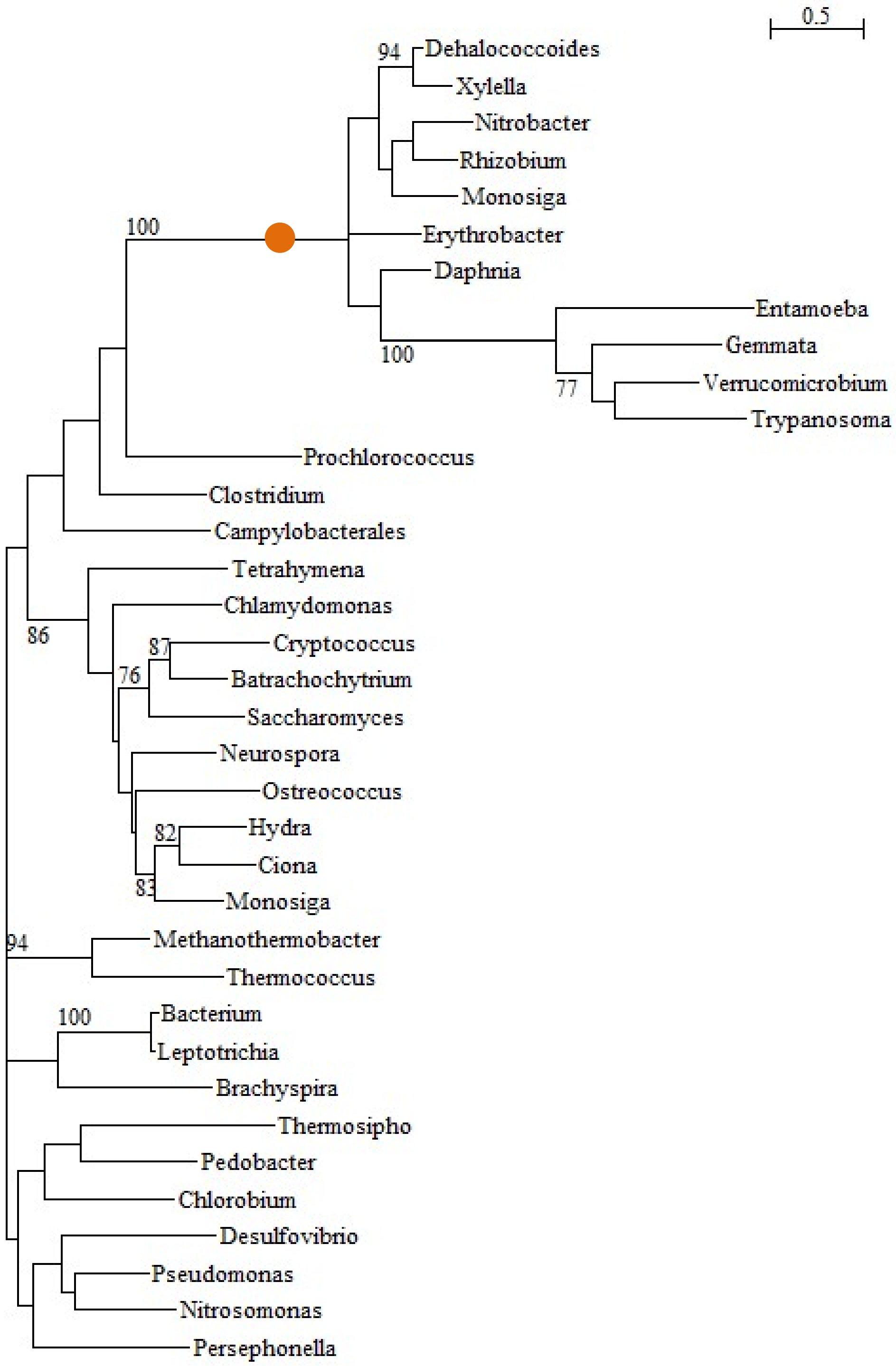

Figure S29. Phylogeny of inorganic polyphosphate/ATP-NAD kinase (GI No. 167516892).

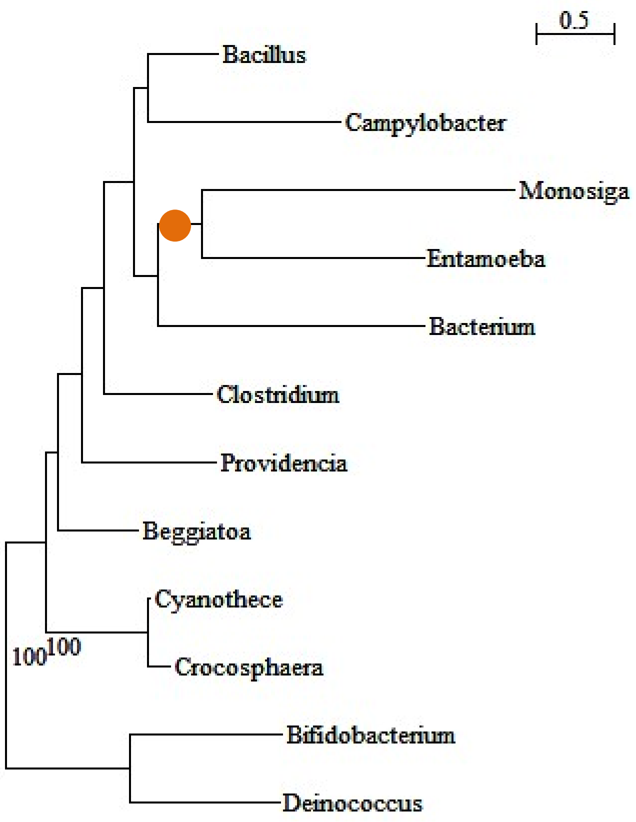

Figure S30. Phylogeny of one hypothetical protein (GI No. 167519801).

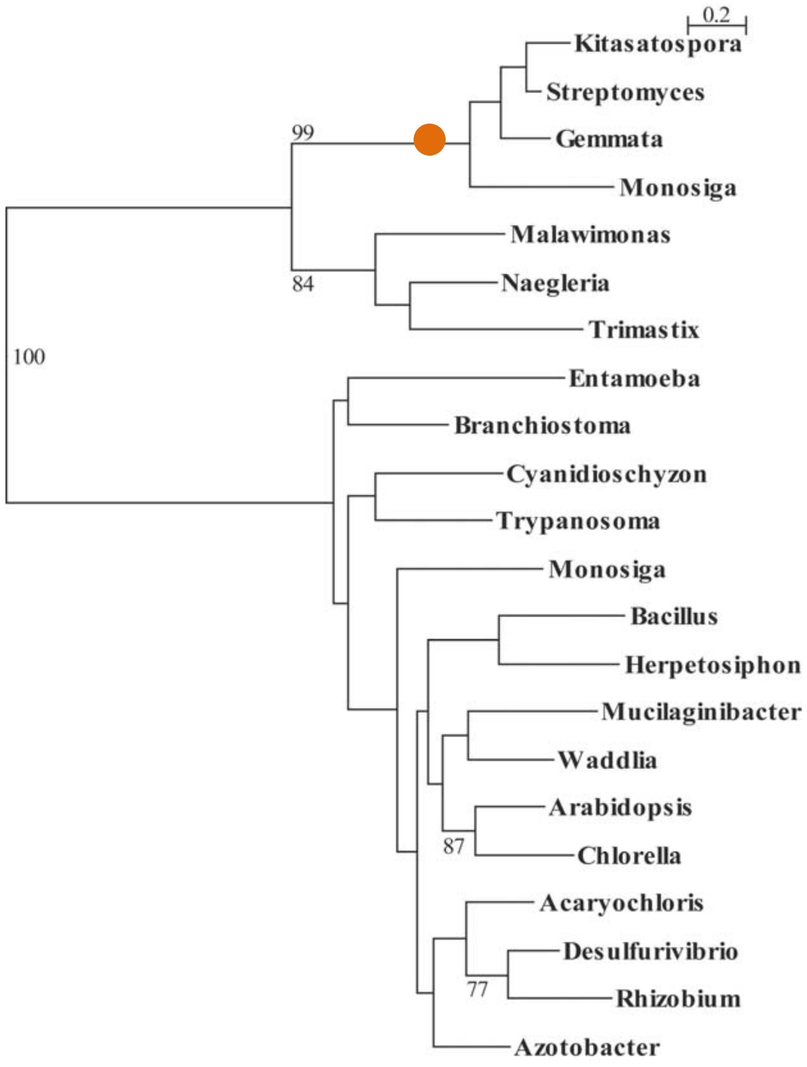

Figure S31. Phylogeny of Uracil-DNA glycosylase (GI No. 167535167).

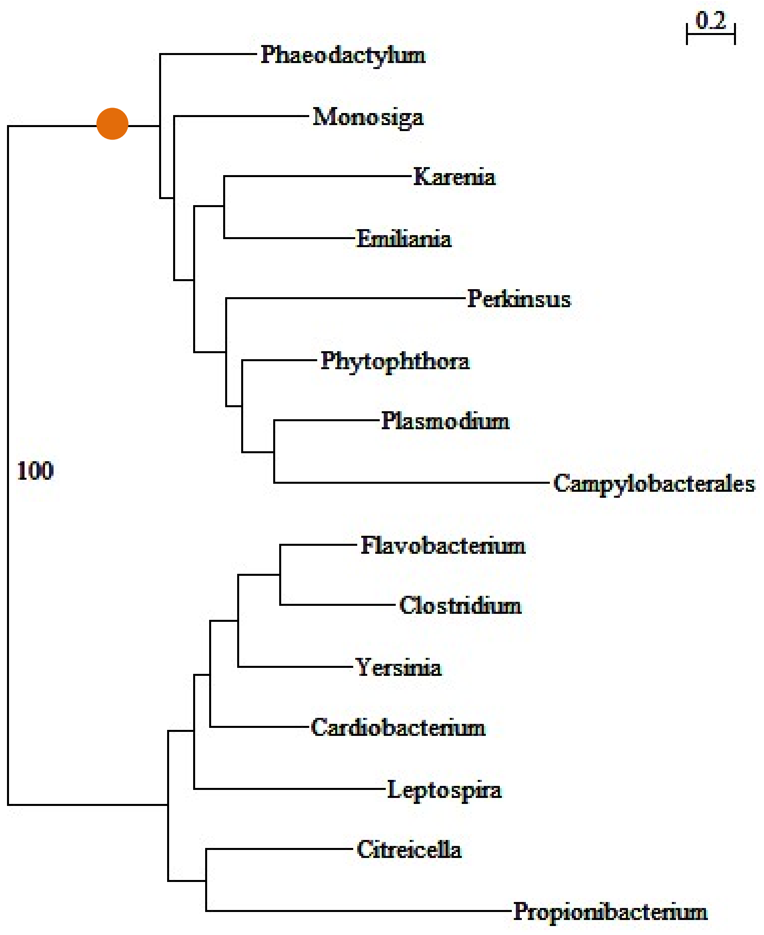

Figure S32. Phylogeny of Leu/Phe-tRNA protein transferase (GI No. 167536799).

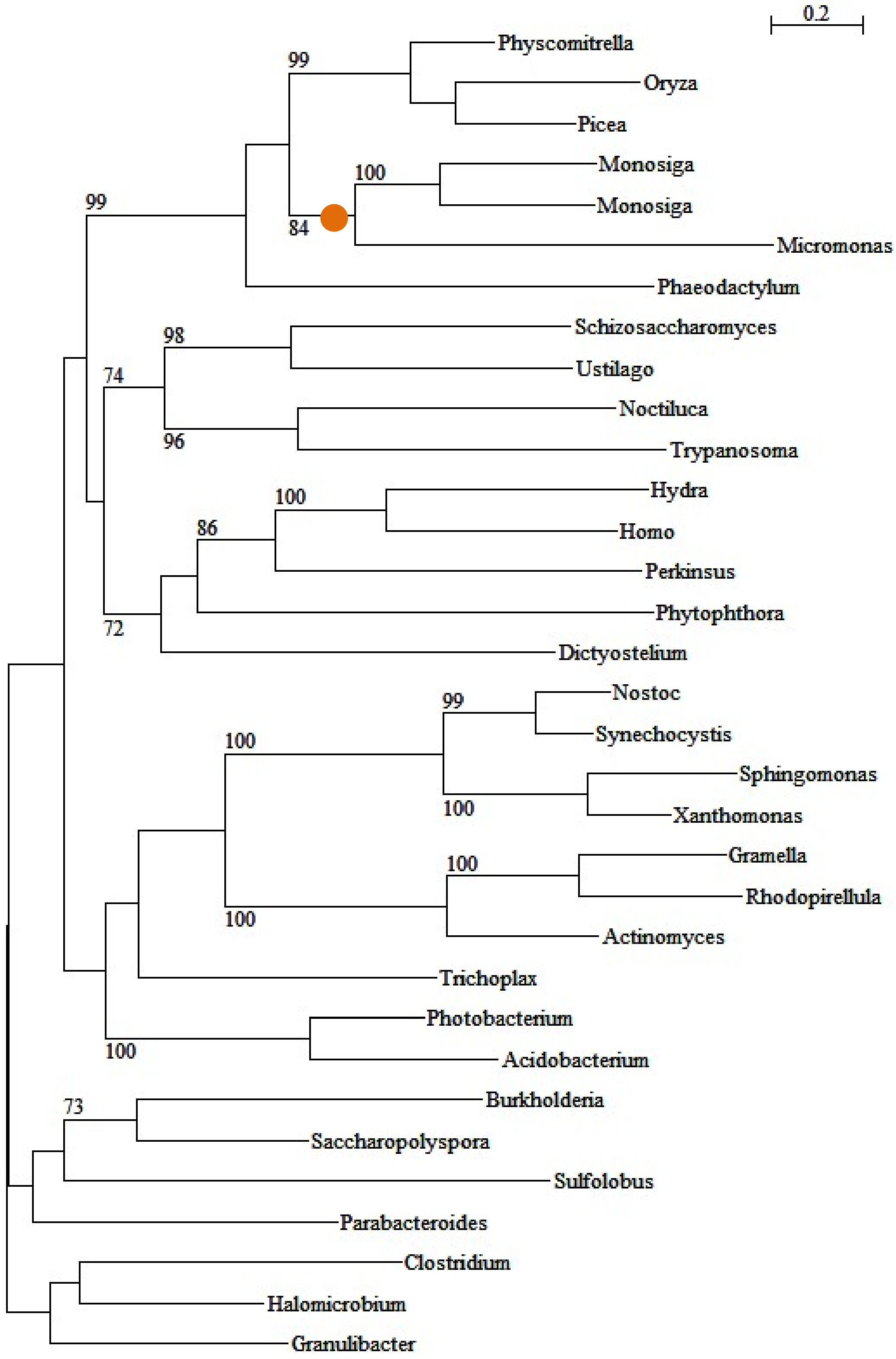

Figure S33. Phylogeny of sorbitol transporter (GI No. 167523619 and 167521355).

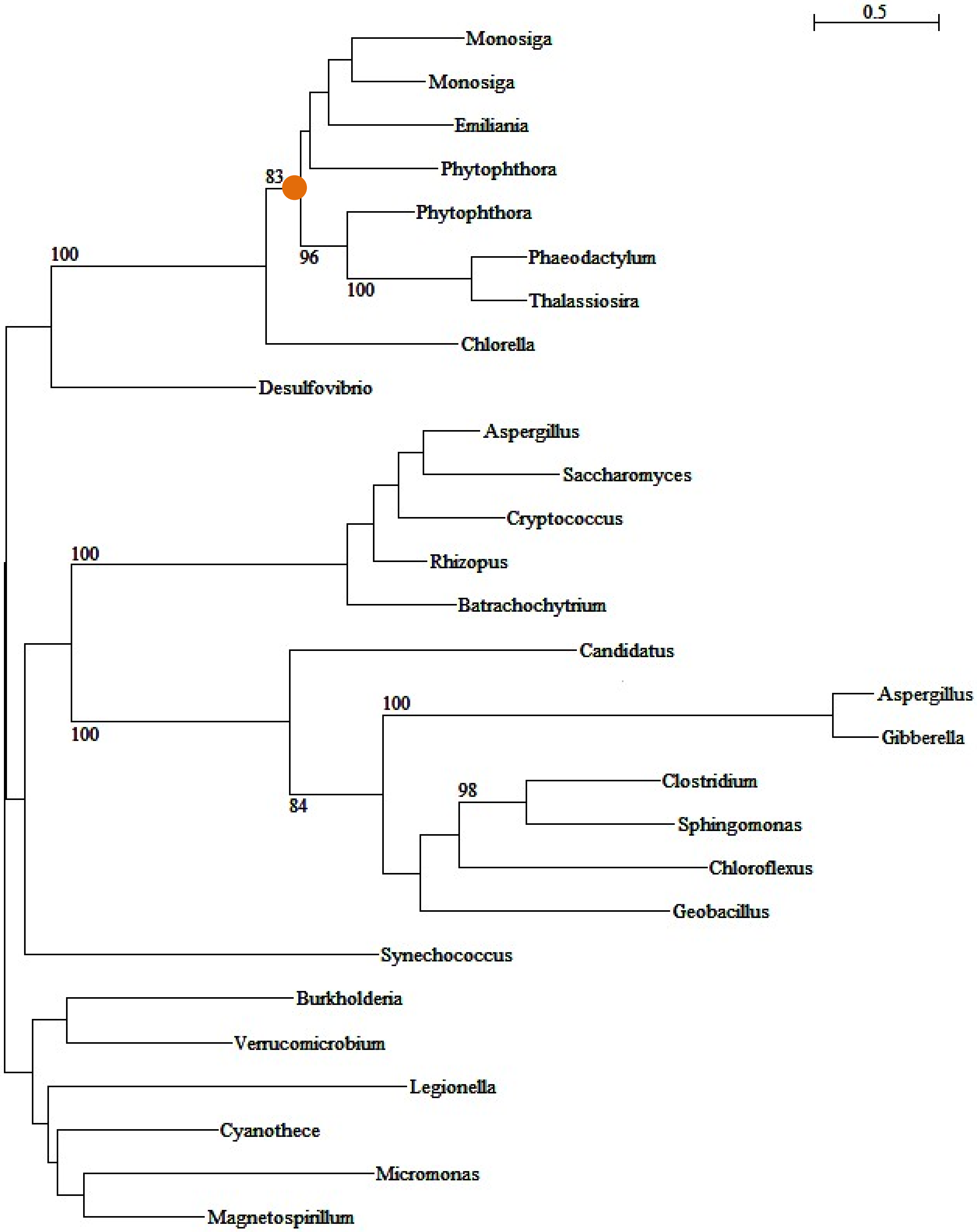

Figure S34. Phylogeny of sulphate transporter (GI No. 167534551 and 167518067).

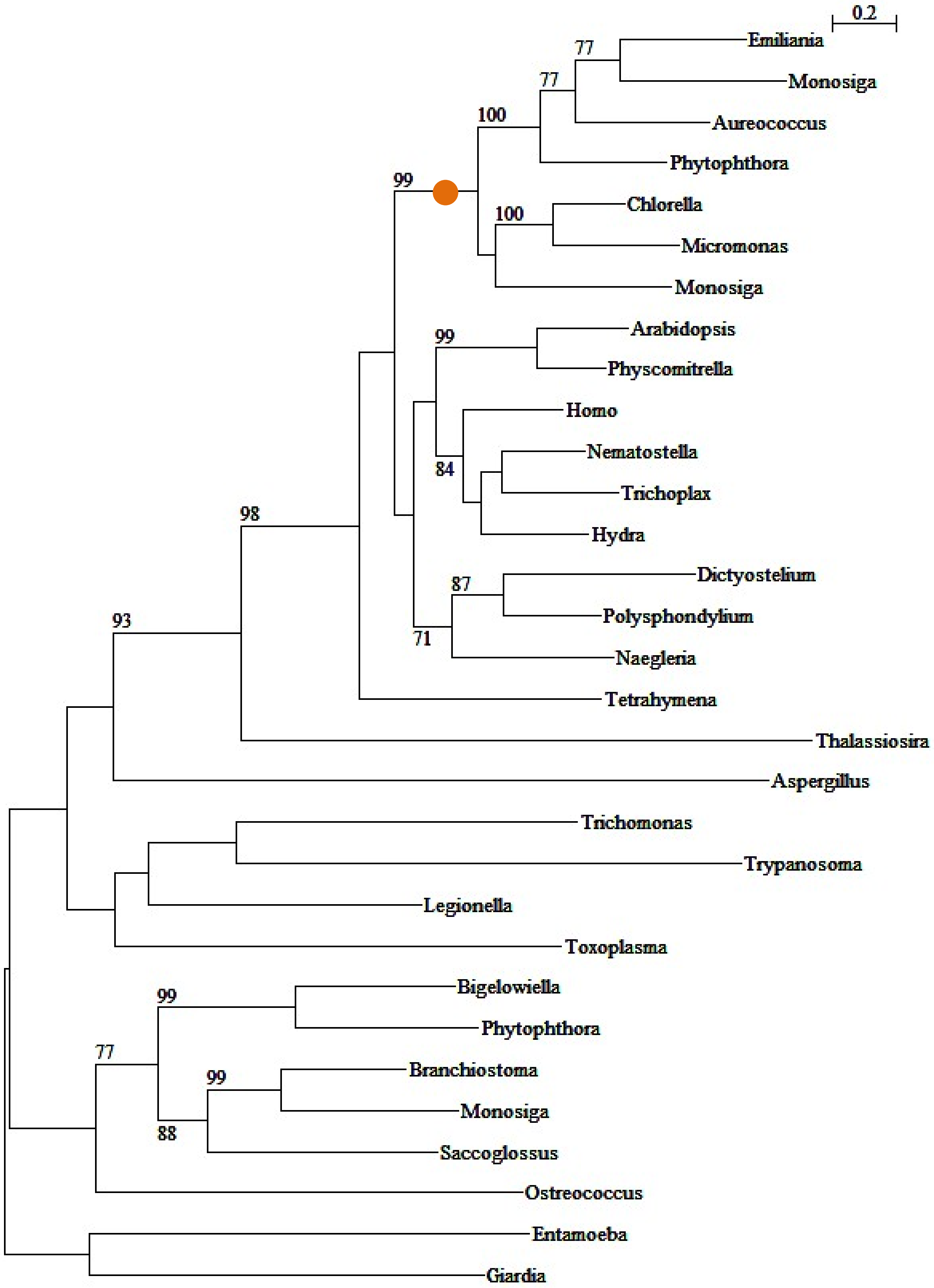

Figure S35. Phylogeny of serine carboxypeptidase (Gi No. 167534702 and 167533602).

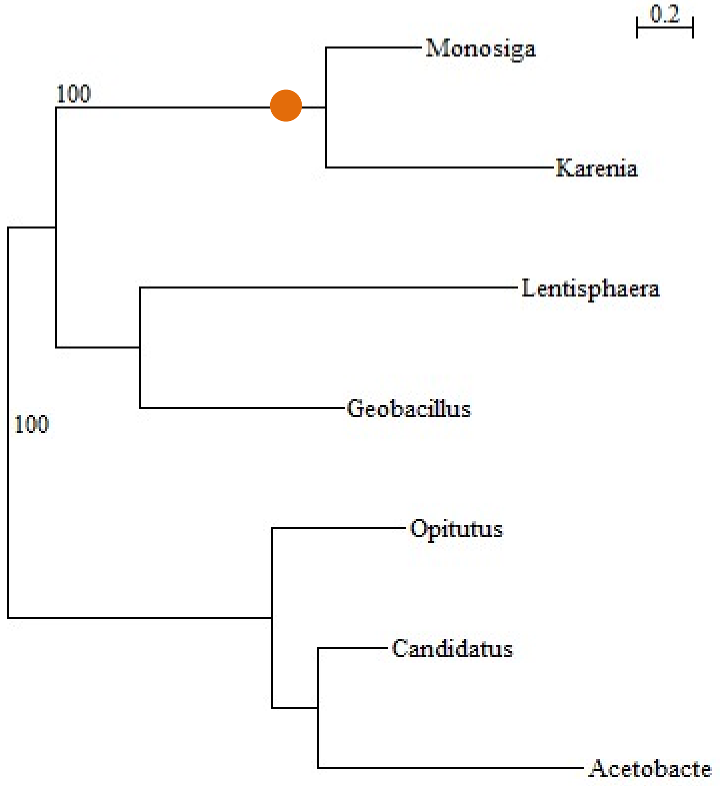

Figure S36. Phylogeny of one hypothetical protein (GI No. 167519849).

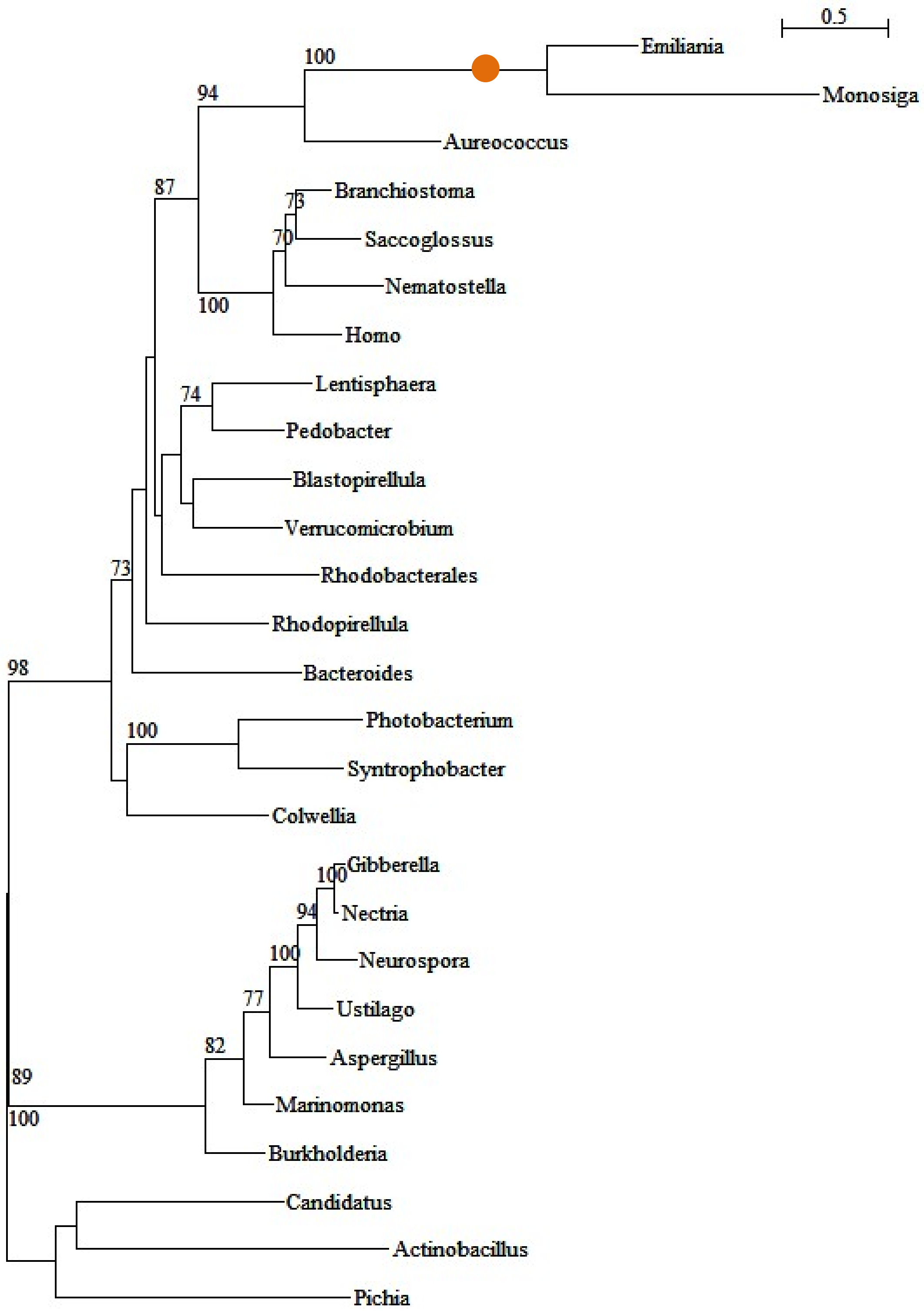

Figure S37. Phylogeny of iduronate-2-sulfatase (GI No. 167524240).

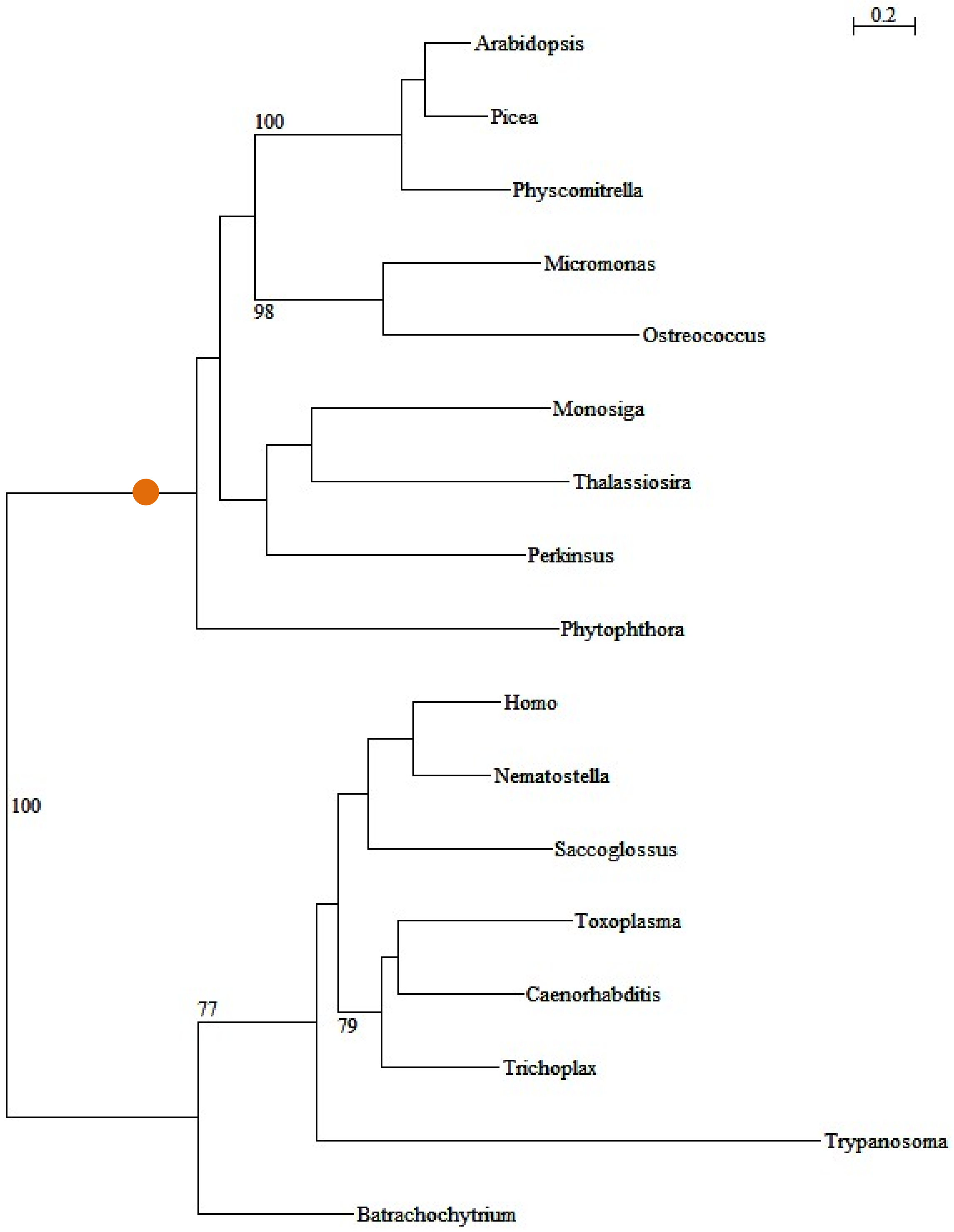

Figure S38. Phylogeny of UDP-galactose translocator (GI No. 167527303).

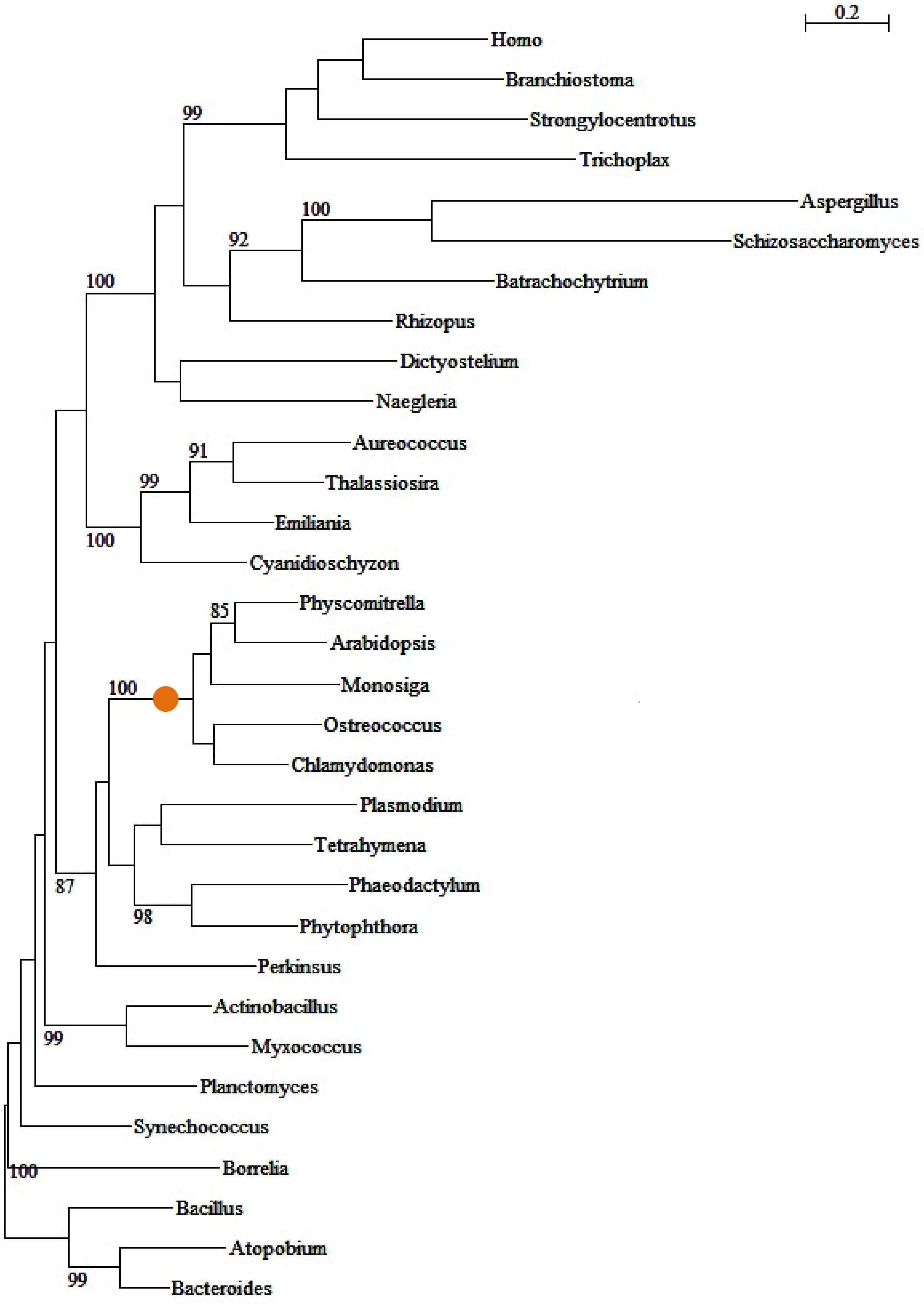

Figure S39. Phylogeny of asparaginyl-tRNA synthetase (GI No. 167527404).

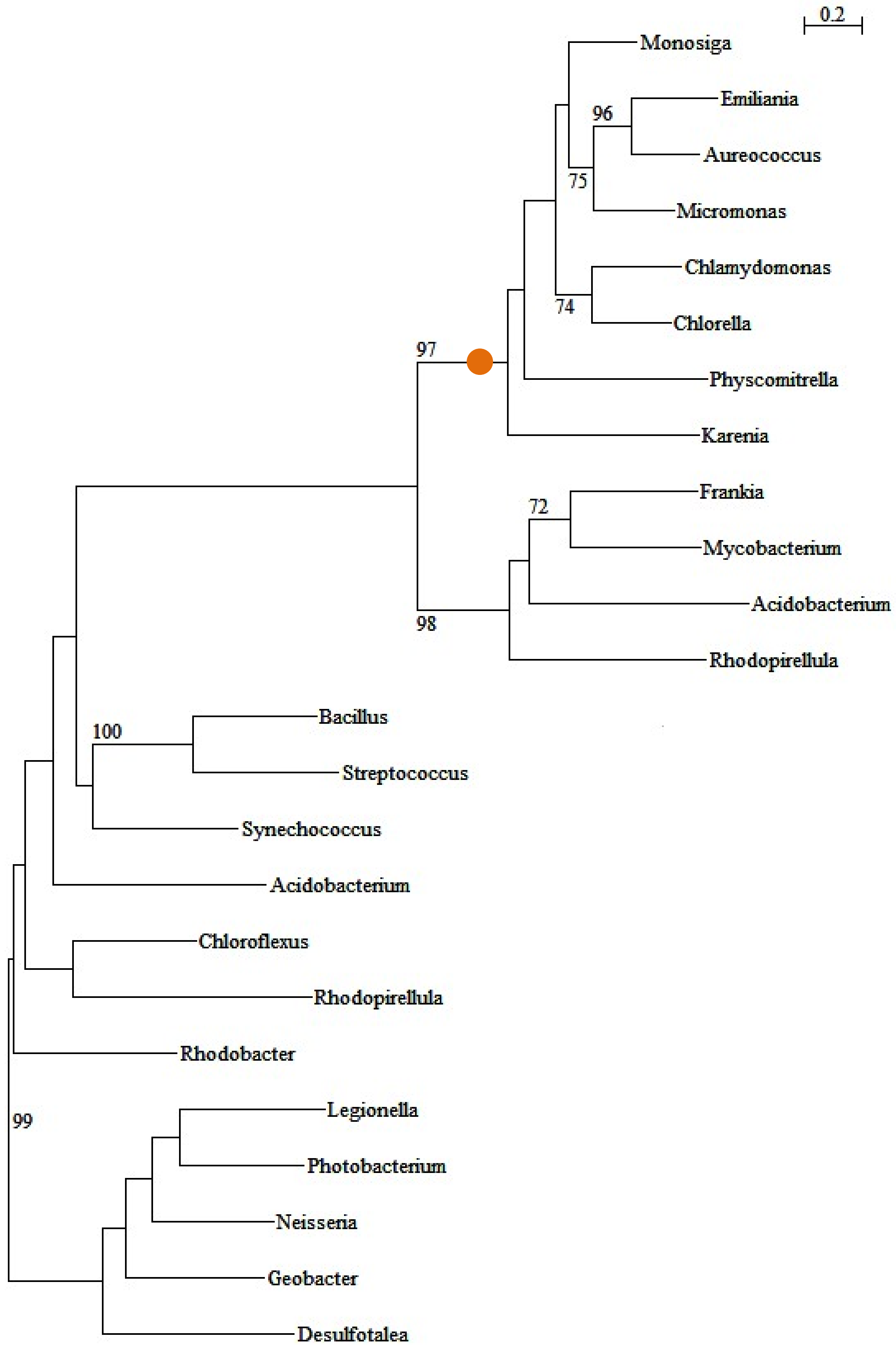

Figure S40. Phylogeny of DNA glycosylase (GI No. 167535061).

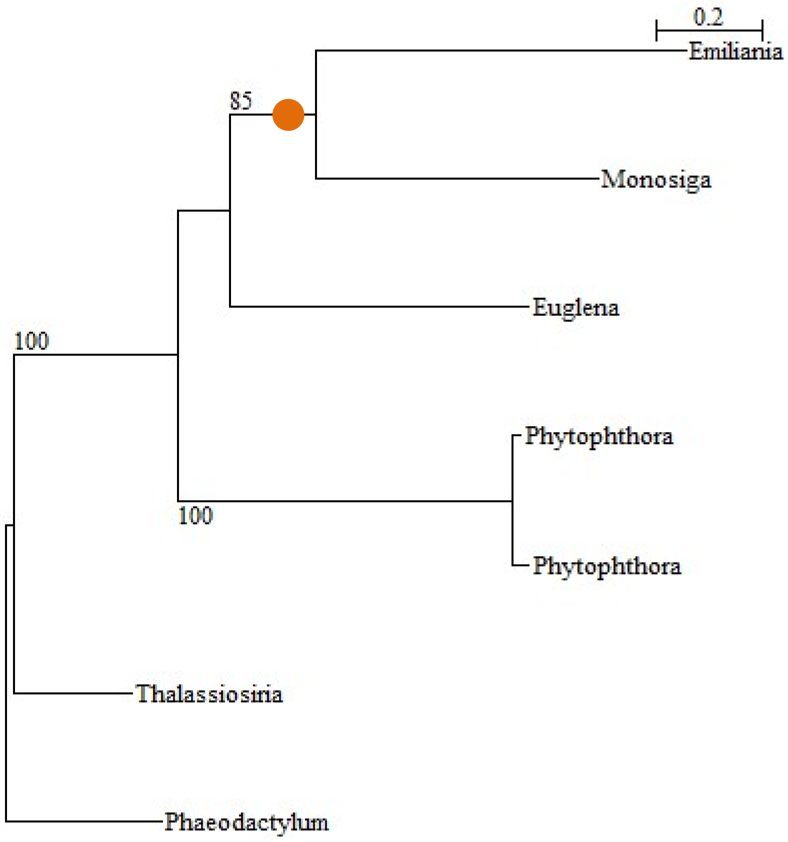

Figure S41. Phylogeny of one hypothetical protein (GI No. 167536775).

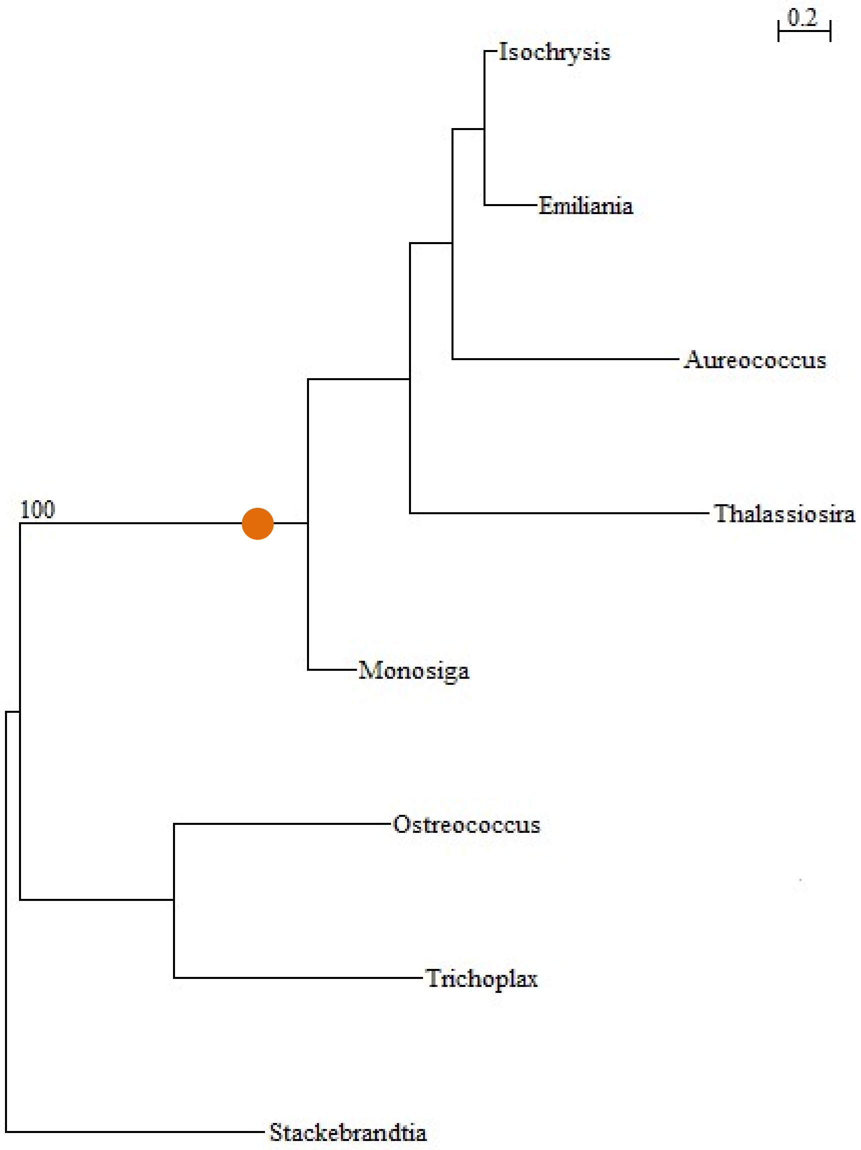

Figure S42. Phylogeny of one hypothetical protein (GI No. 167524032).

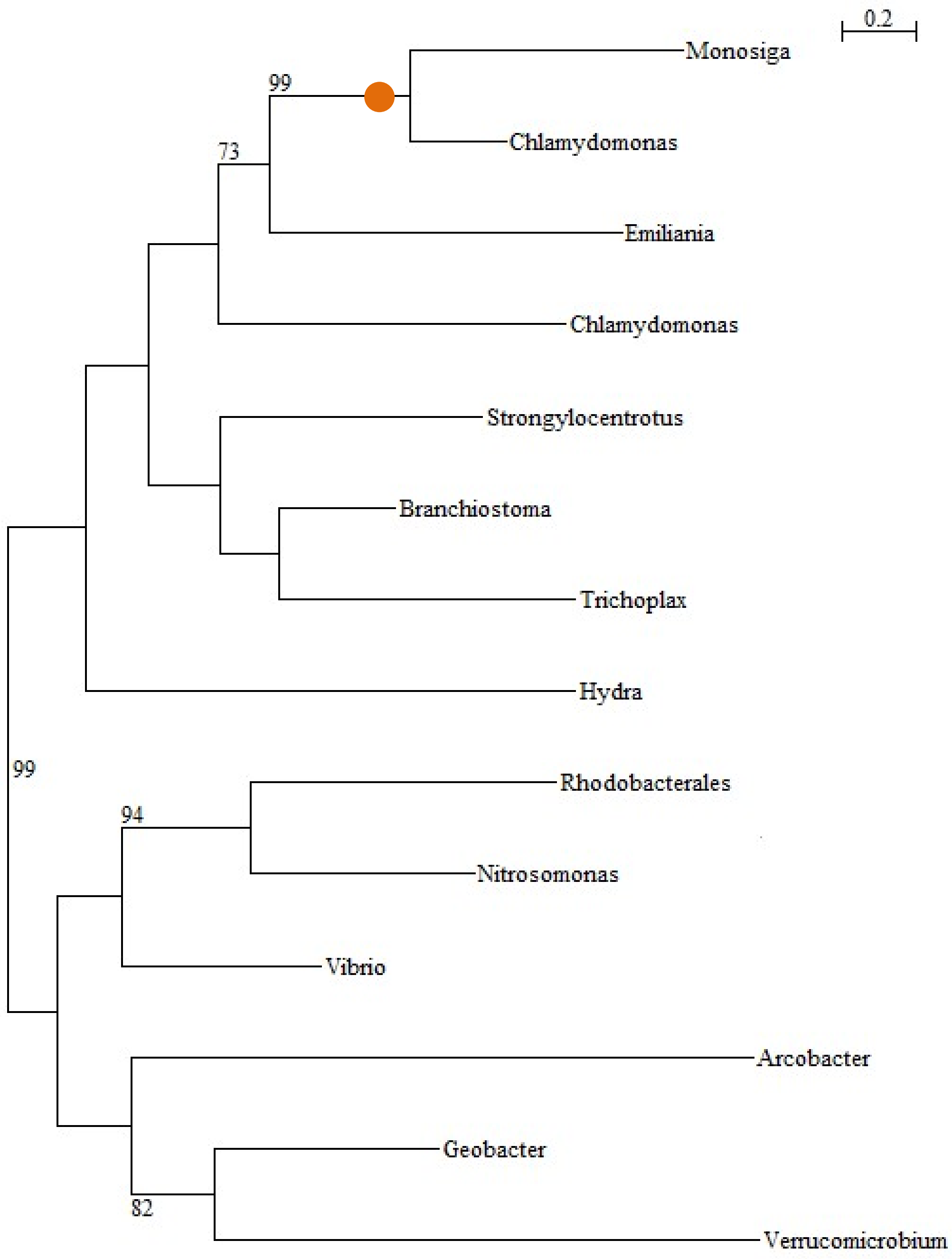

Figure S43. Phylogeny of one hypothetical protein (GI No. 167534491).

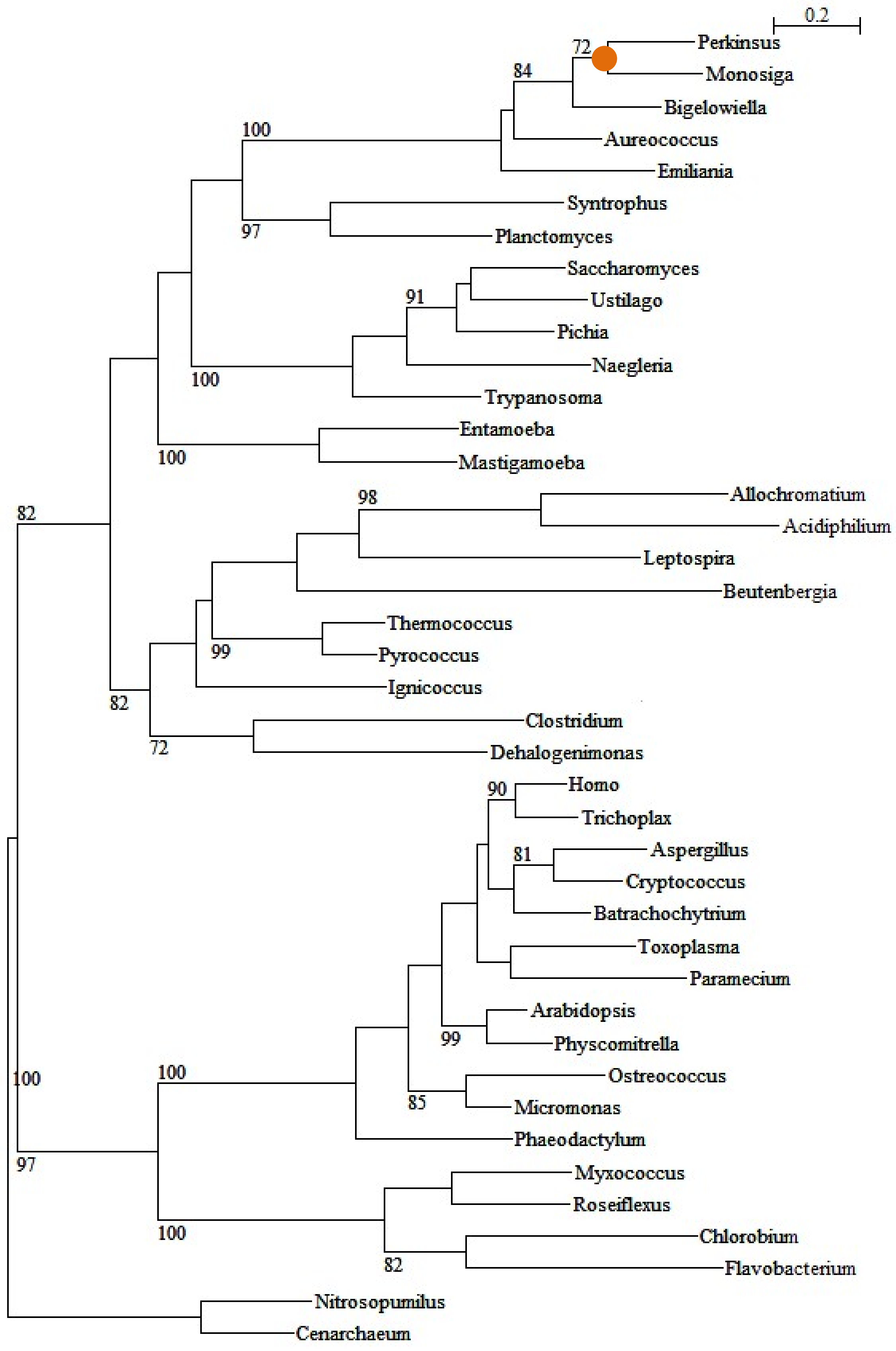

Figure S44. Phylogeny of dolichol phosphate mannose synthase (GI No. 167535637).

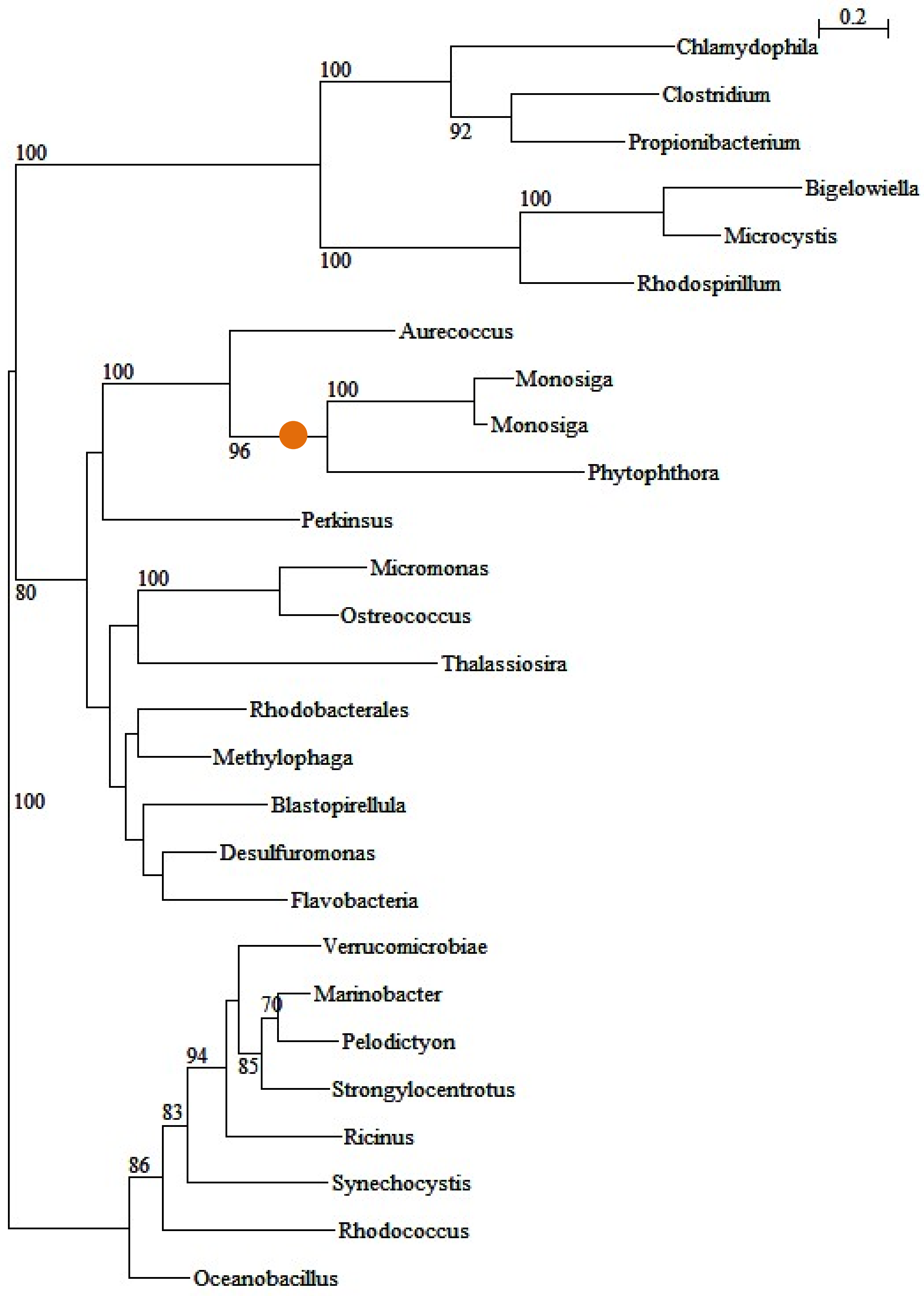

Figure S45. Phylogeny of sulphate transporter (Gi No. 167535659 and 167538557).

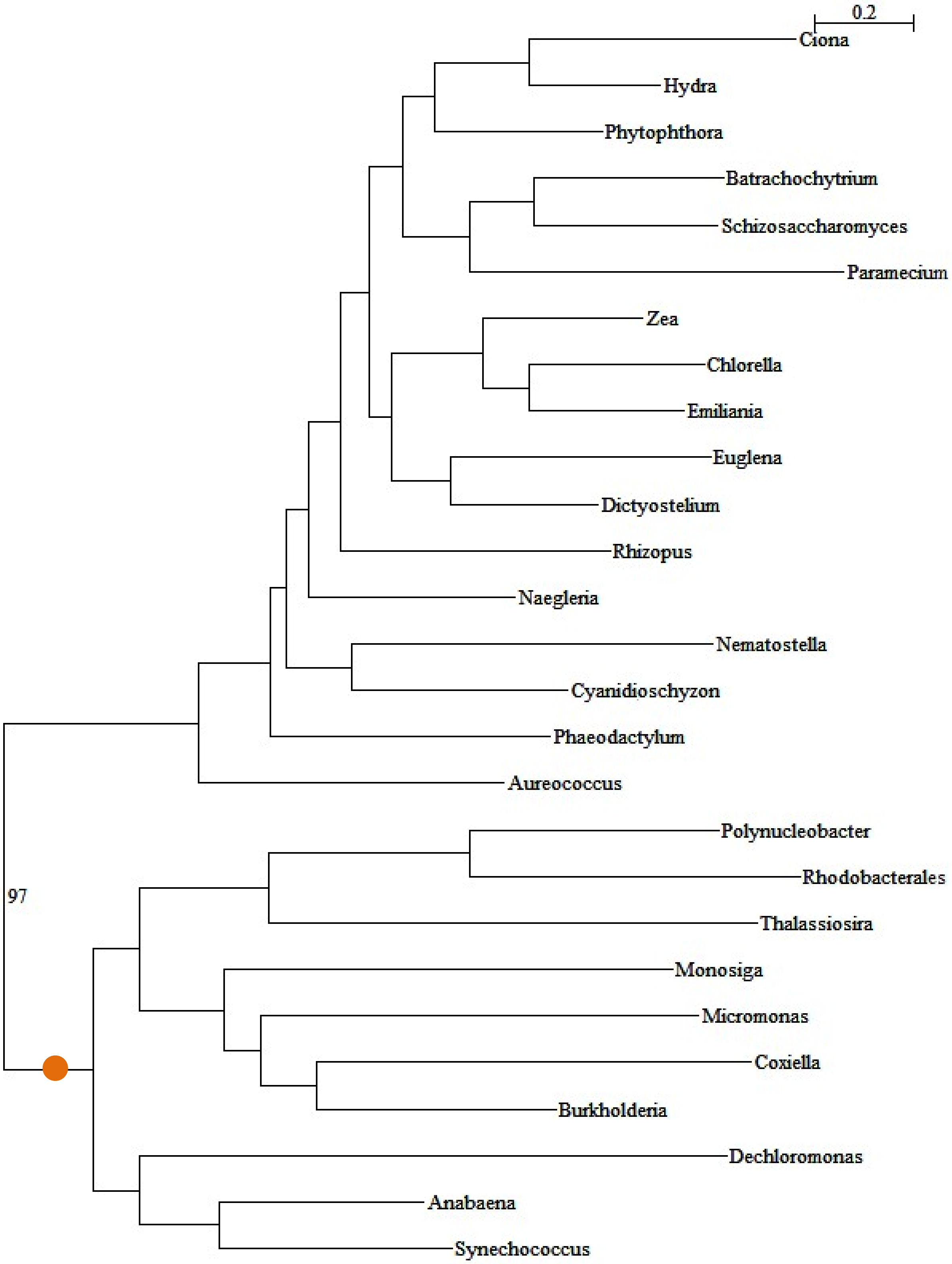

Figure S46. Phylogeny of phytochelatin synthase (GI No. 167537519).

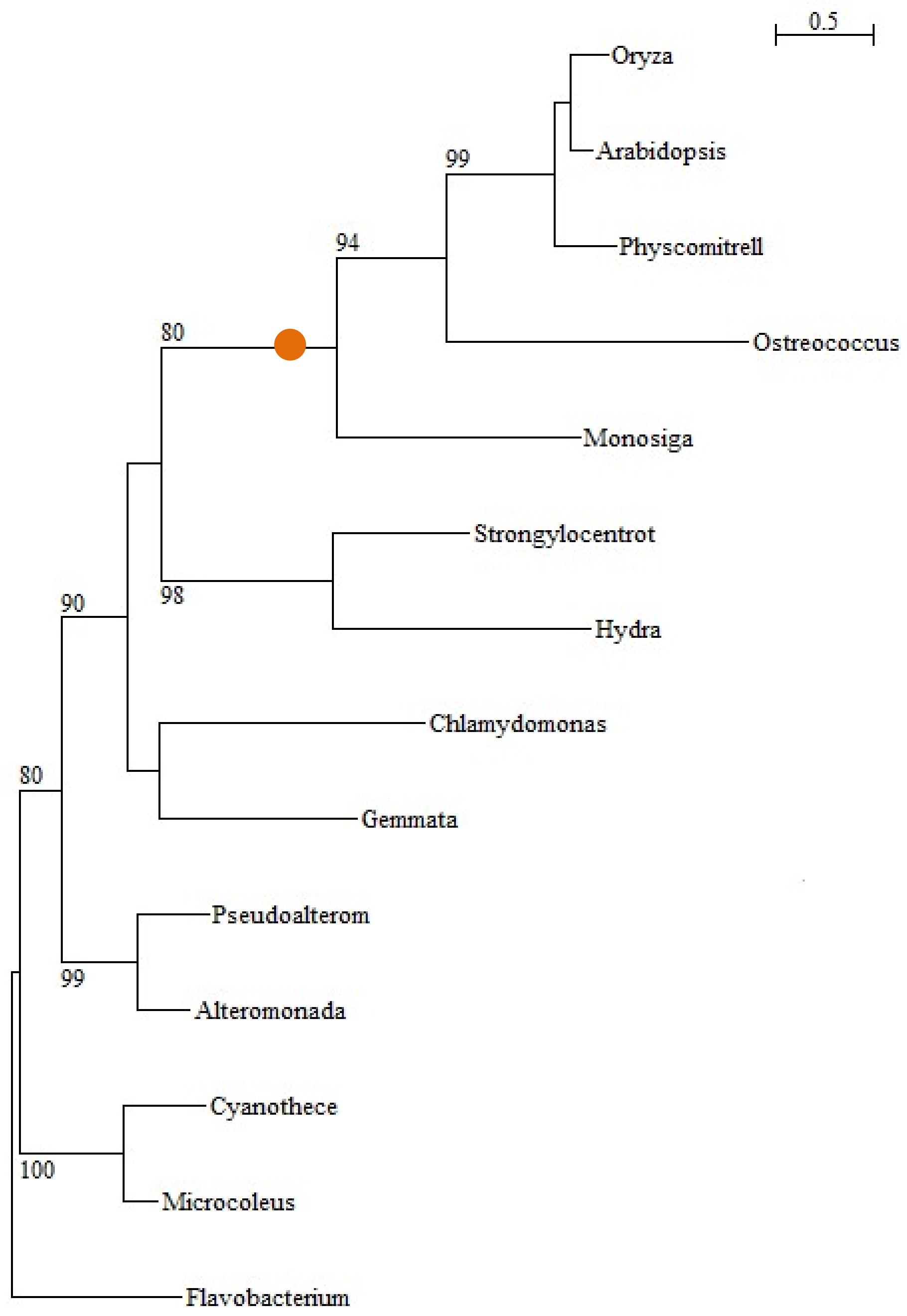

Figure S47. Phylogeny of one hypothetical protein (GI No. 167537477).

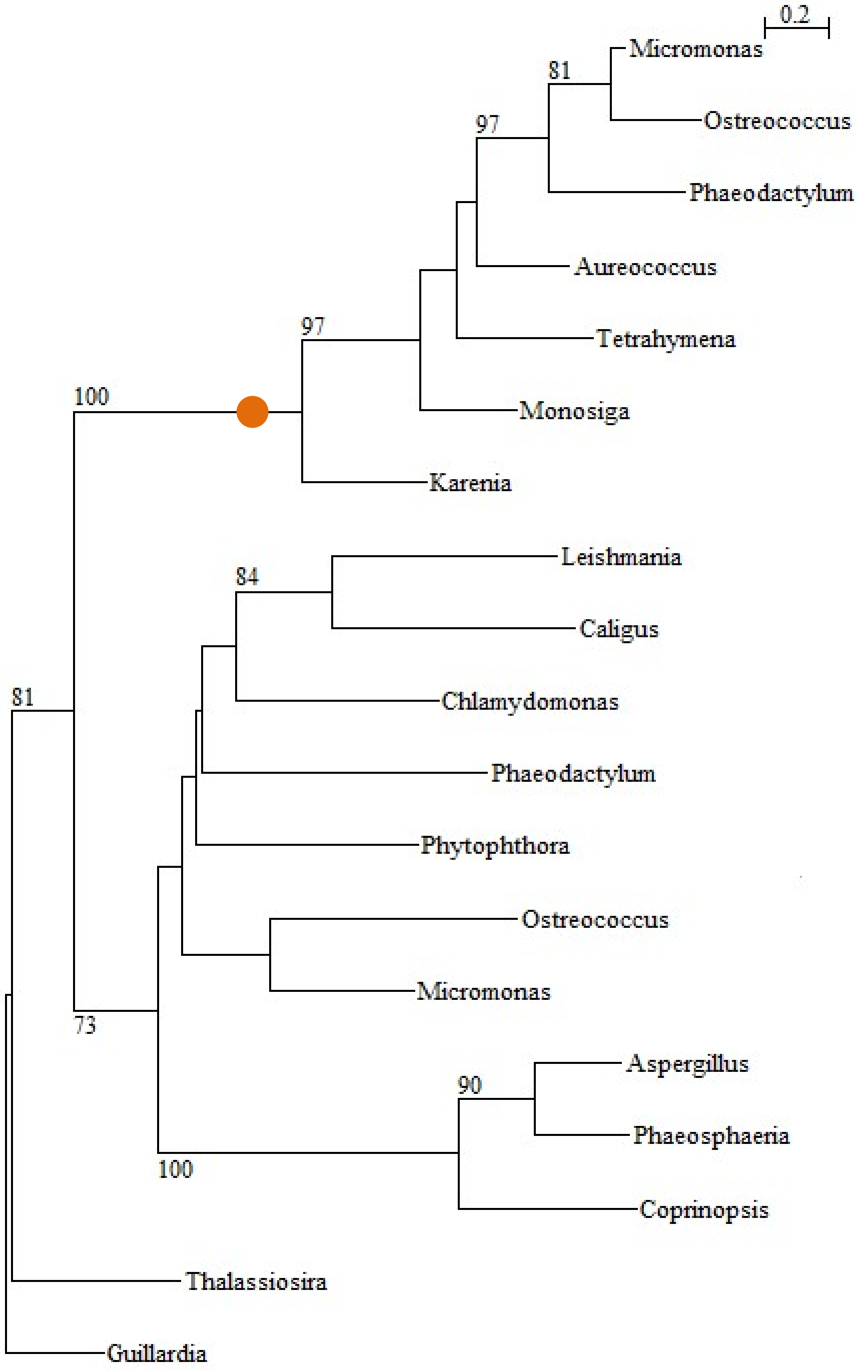

Figure S48. Phylogeny of one hypothetical protein (GI No. 167537588).

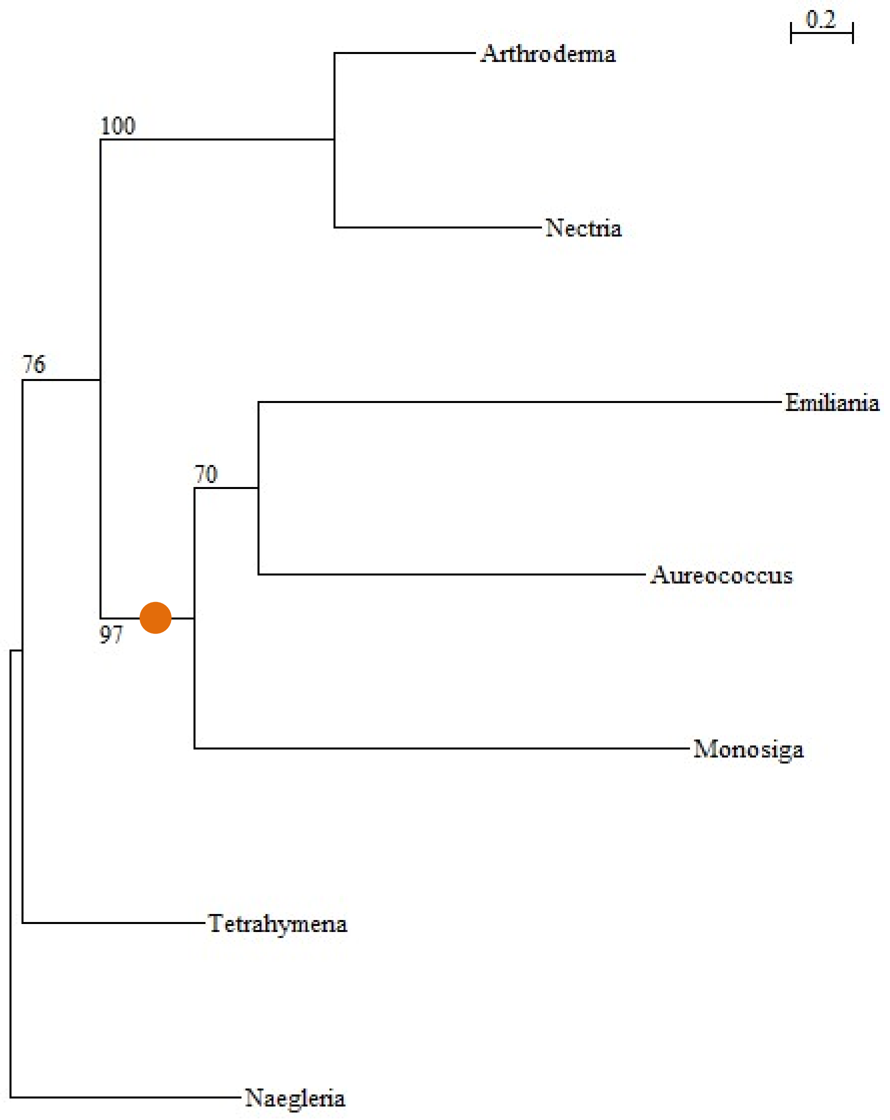

Figure S49. Phylogeny of one hypothetical protein (GI No. 167538097).

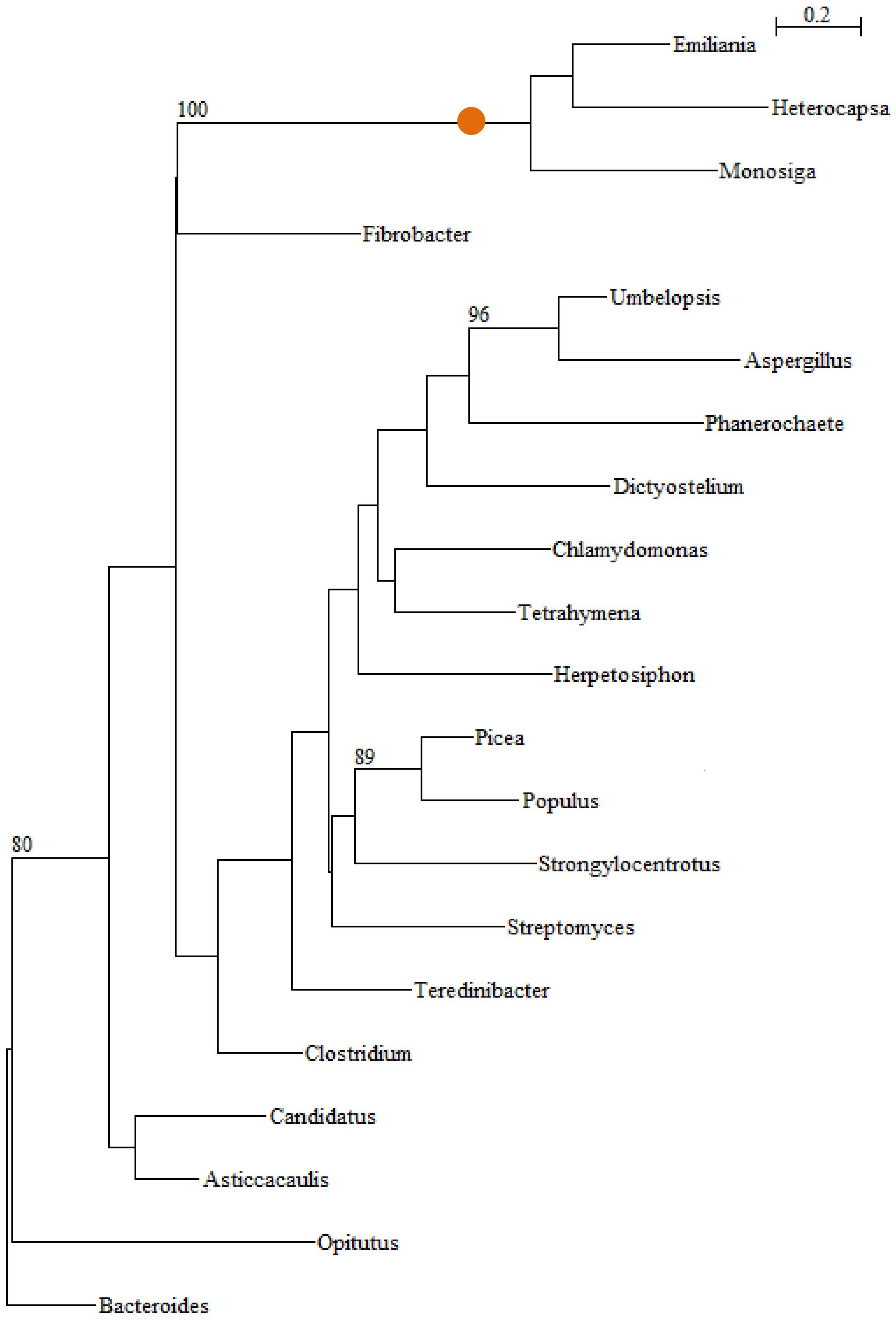

Figure S50. Phylogeny of one hypothetical protein (GI No. 167520320).

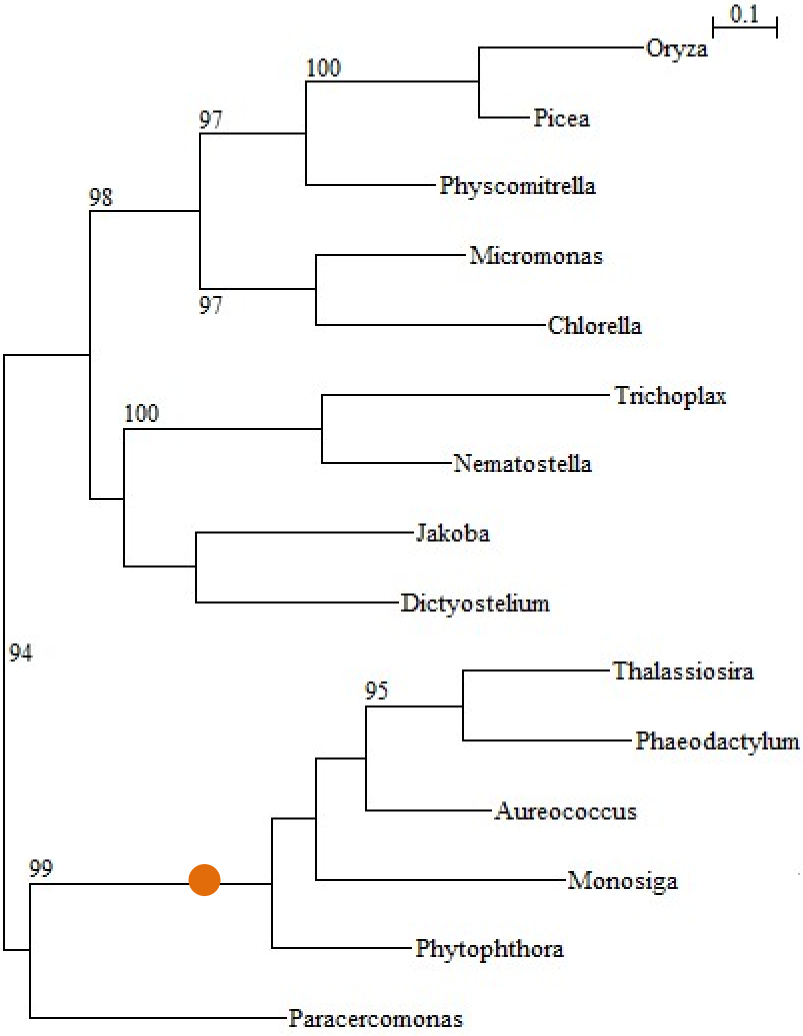

Figure S51. Phylogeny of Ca2 :Cation Antiporter (CaCA) Family (GI No. 167516696).

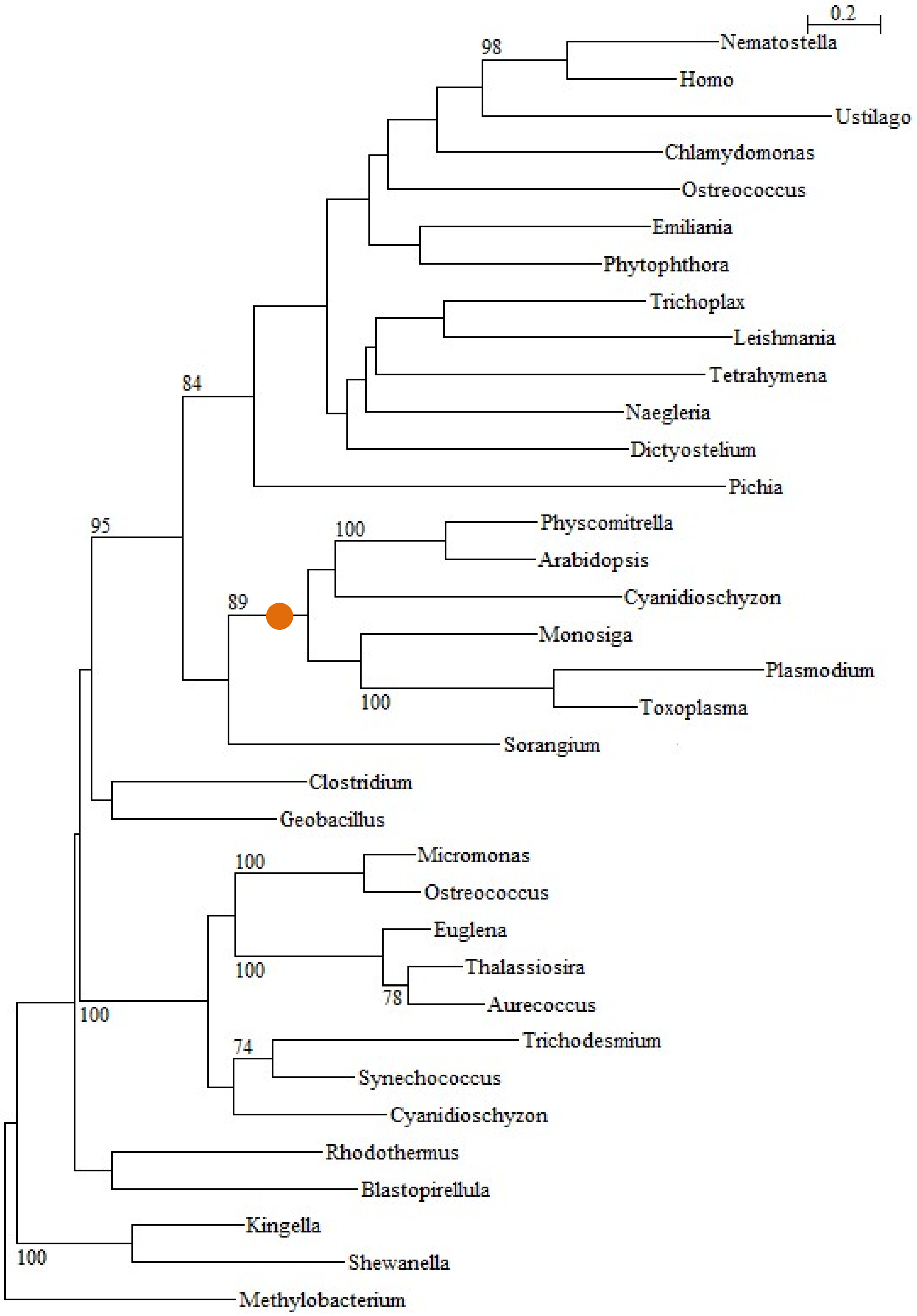

Figure S52. Phylogeny of ubiquinone biosynthesis protein coq-8 or ABC1 family protein (GI No. 167519152).

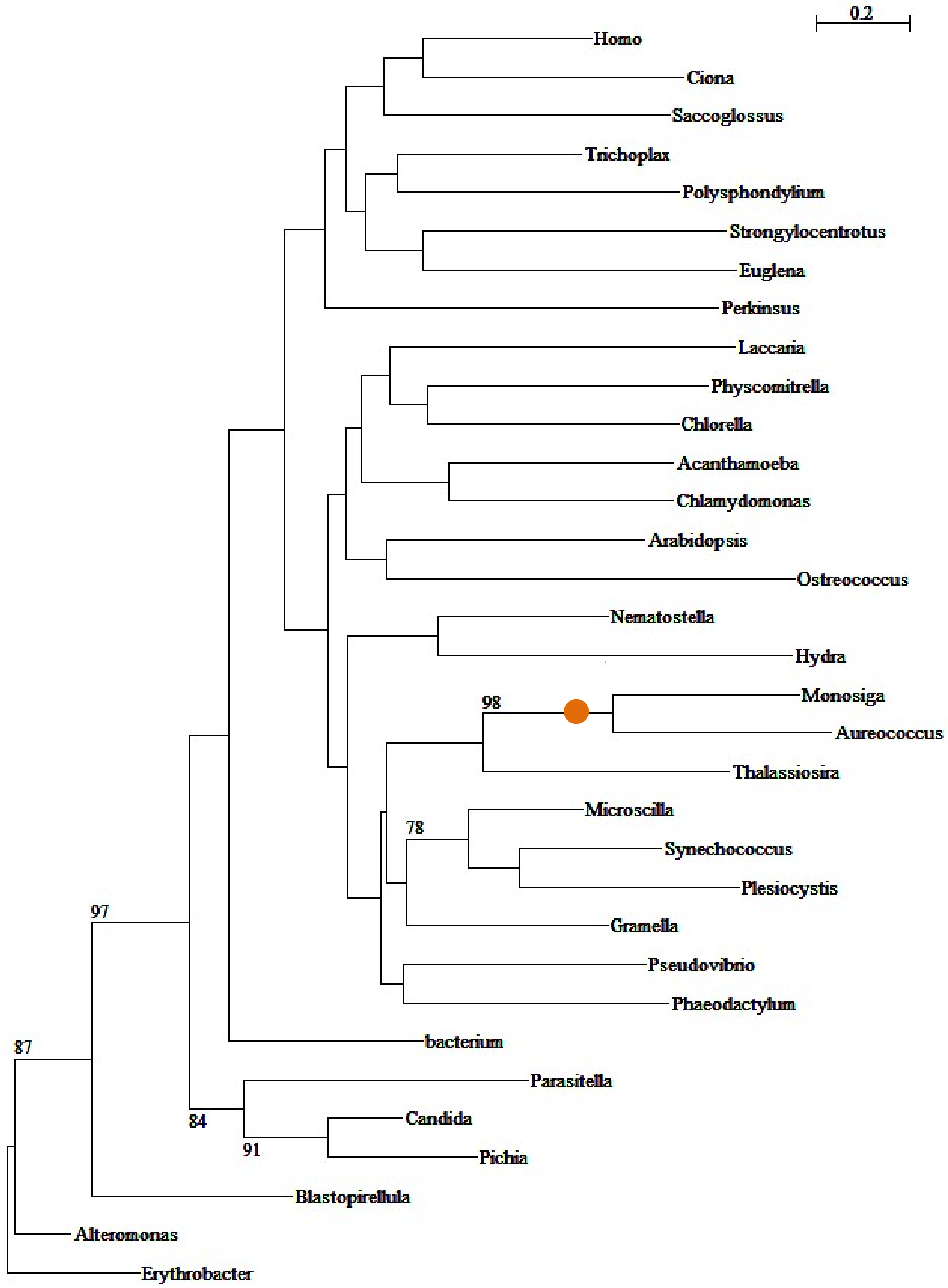

Figure S53. Phylogeny of aldehyde reductase (GI No. 167519881).

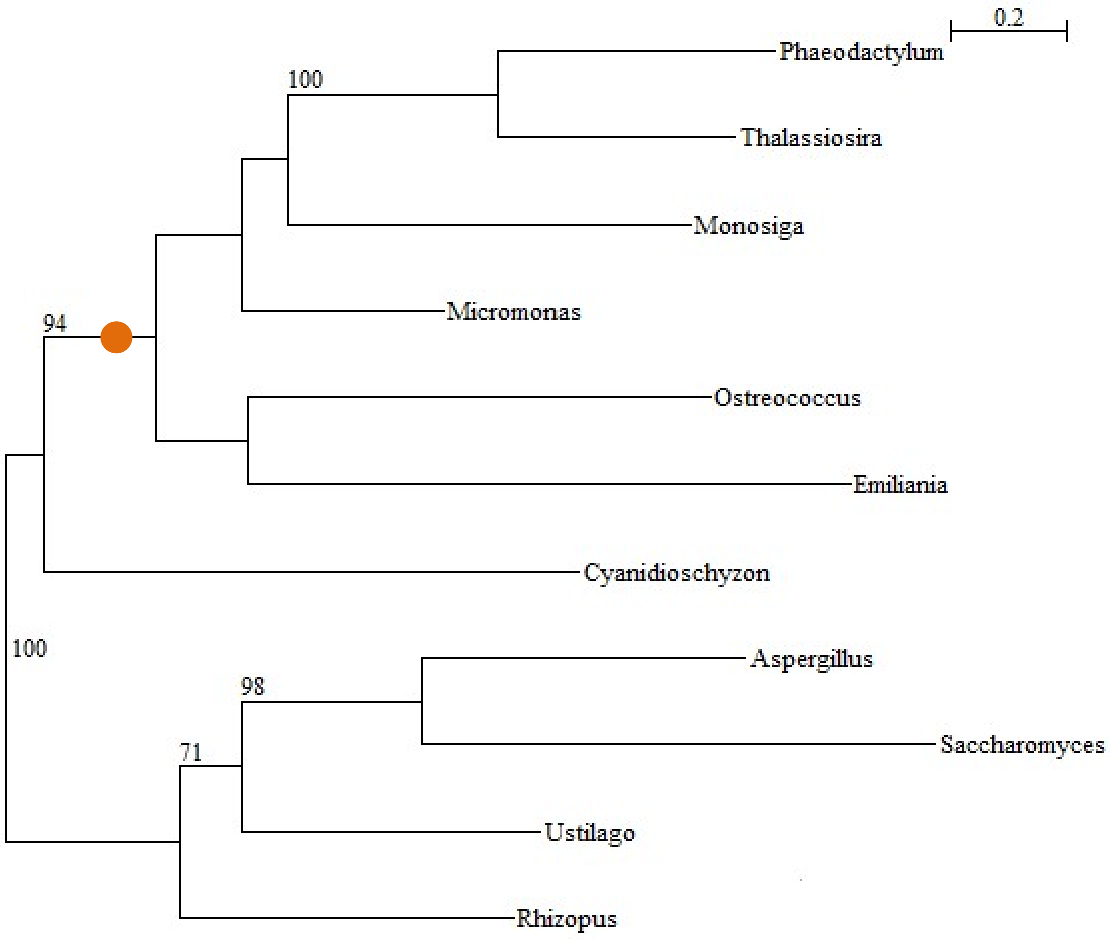

Figure S54. Phylogeny of oxidoreductase (GI No. 167520798).

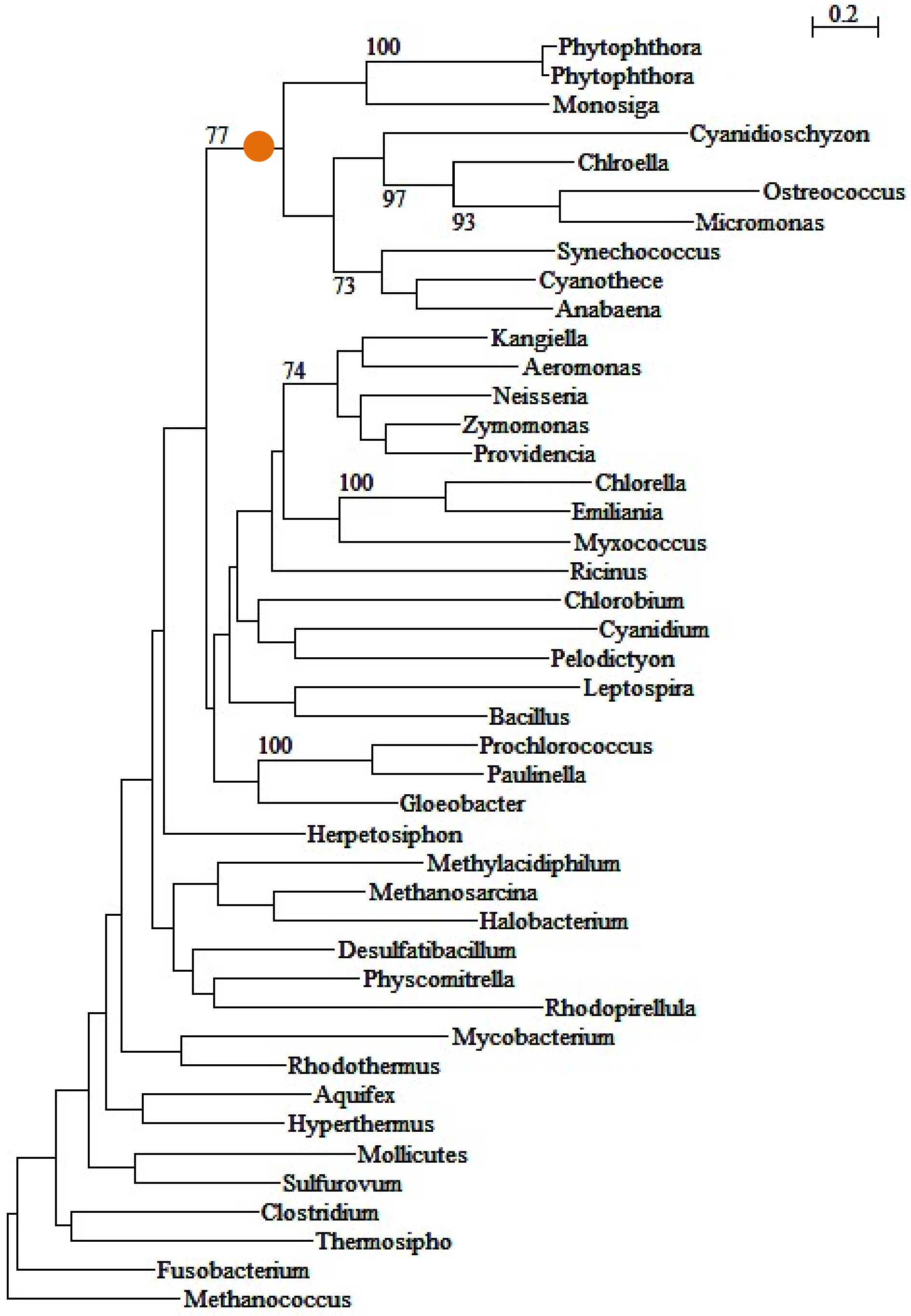

Figure S55. Phylogeny of uroporphyrinogen III synthase/methyltransferase (GI No. 167515658).

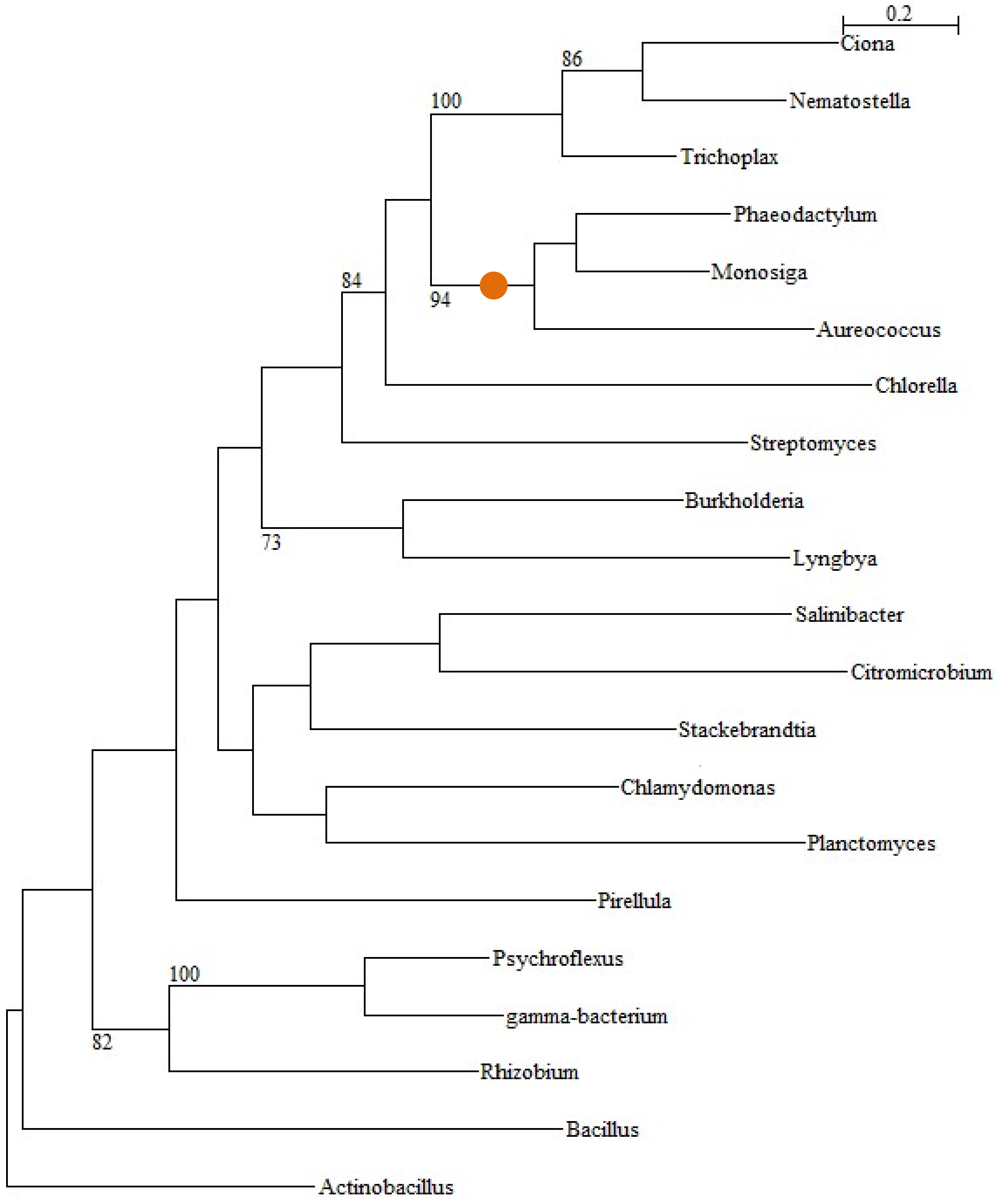

Figure S56. Phylogeny of short-chain dehydrogenease (GI No. 167516176).

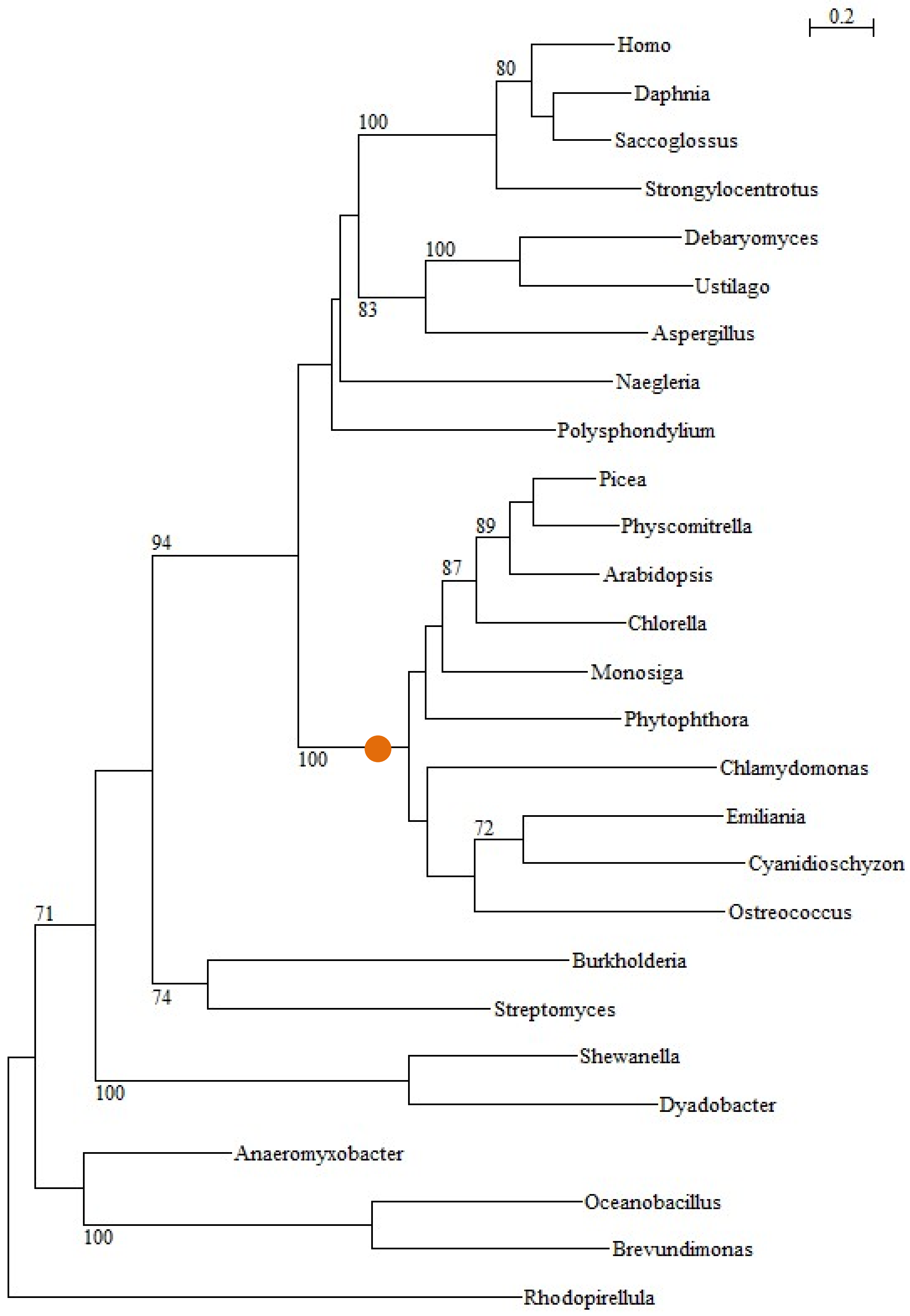

Figure S57. Phylogeny of acid phosphatase (GI No. 167518307).

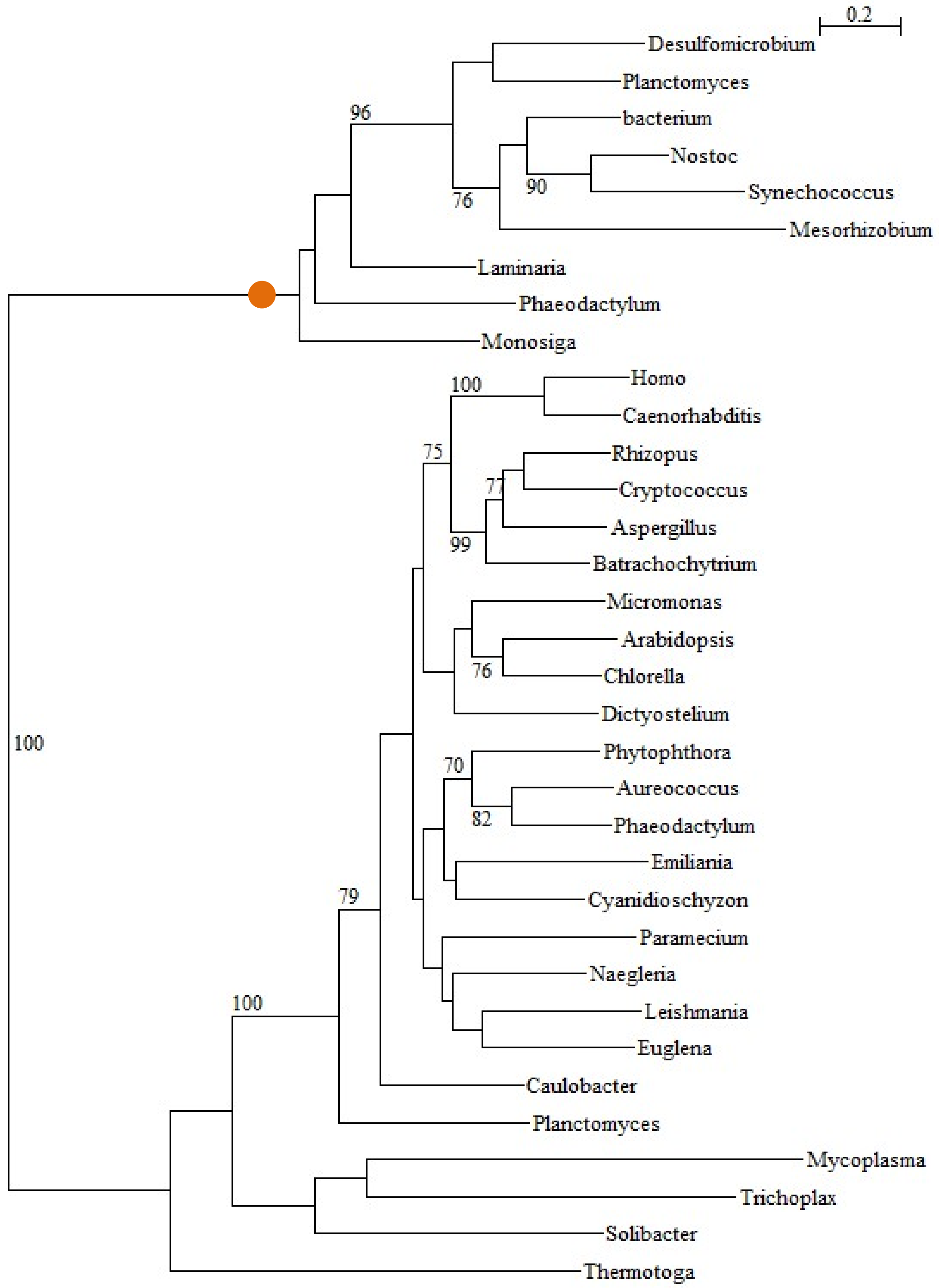

Figure S58. Phylogeny of mercuric reductase (GI No. 167519689).

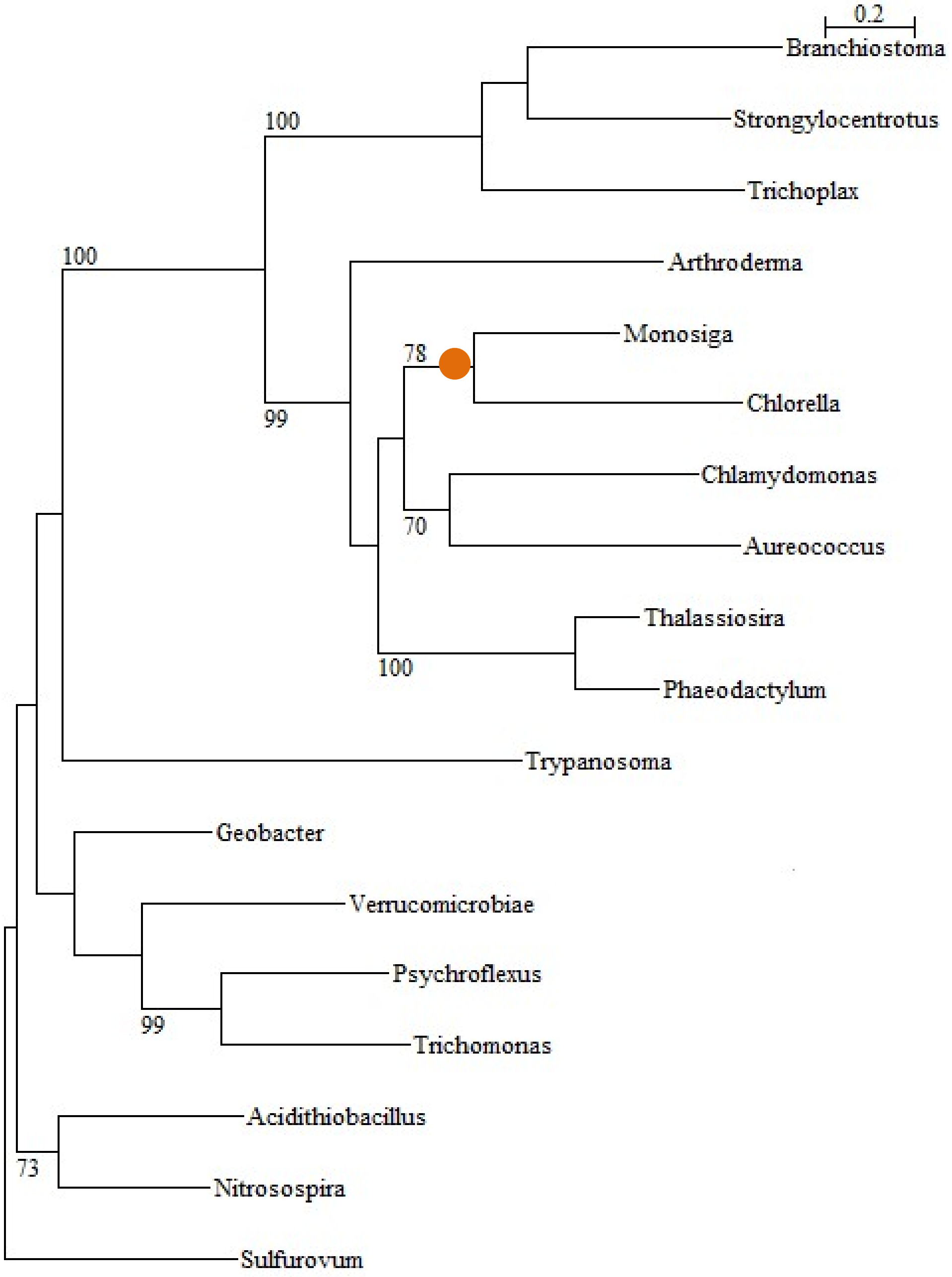

Figure S59. Phylogeny of methyltransferase (GI No. 167518566).

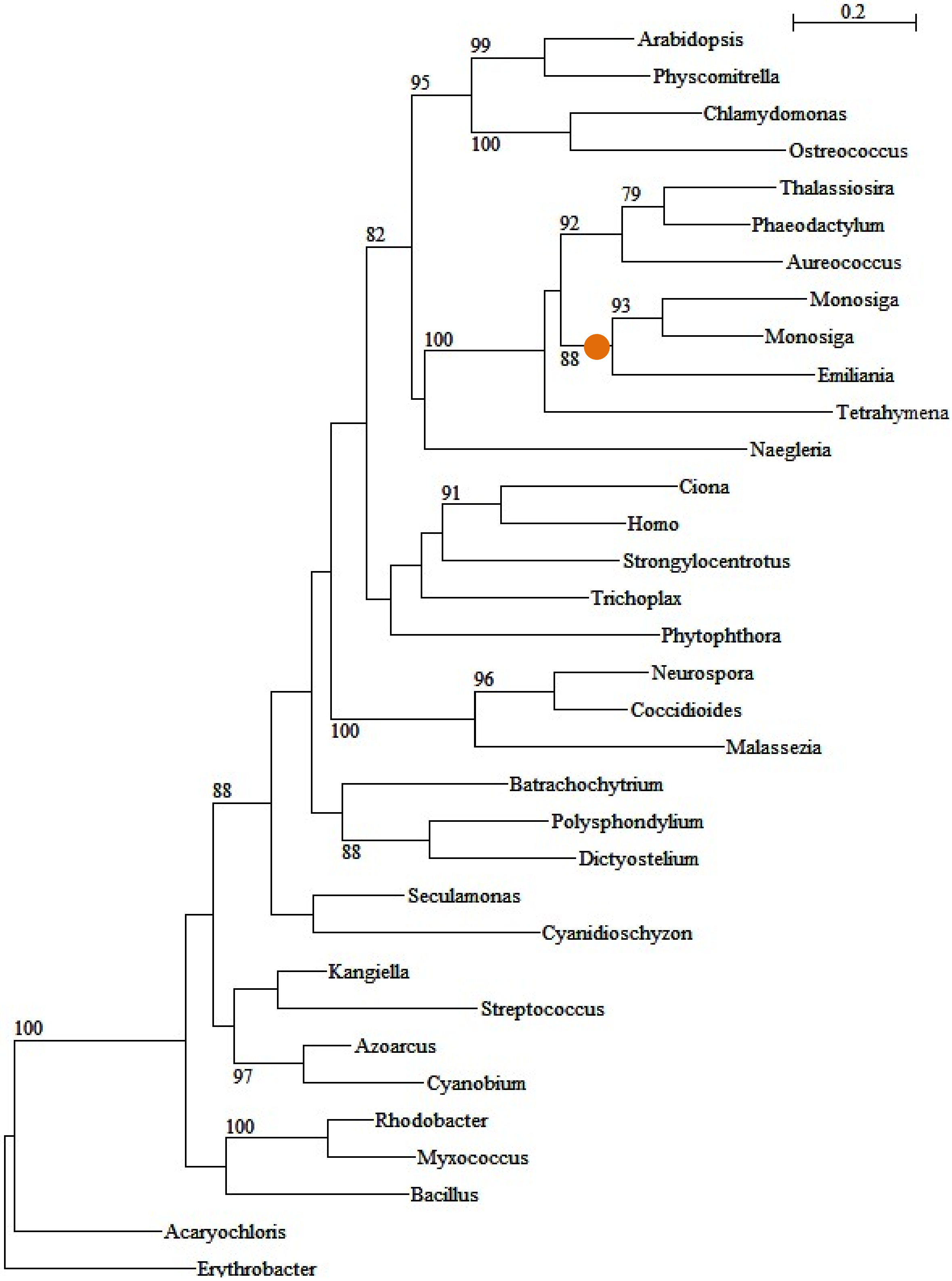

Figure S60. Phylogeny of alcohol dehydrogenase class III (Gi No. 167521587 and 167522892).

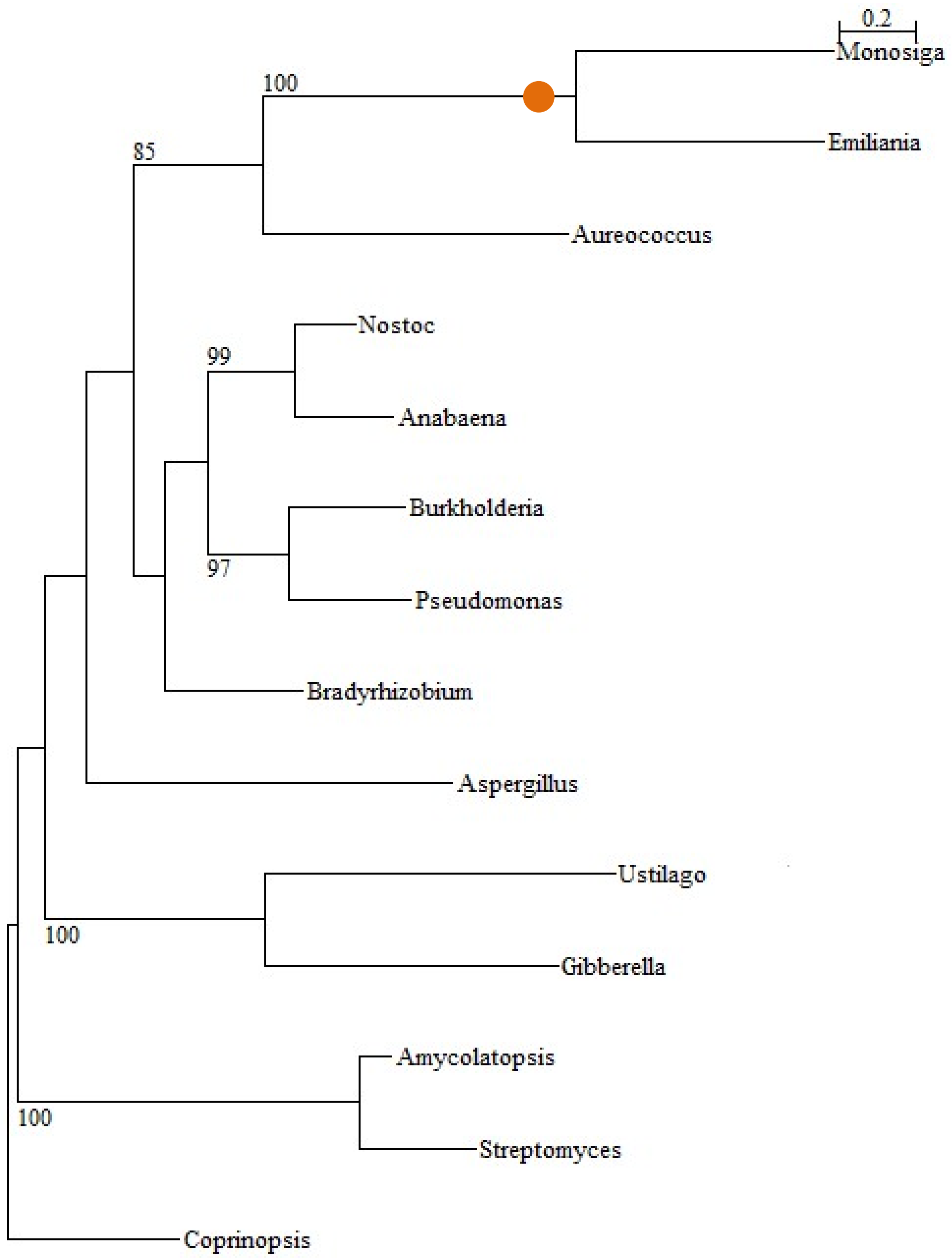

Figure S1. Phylogeny of one hypothetical protein (GI No. 167515494).

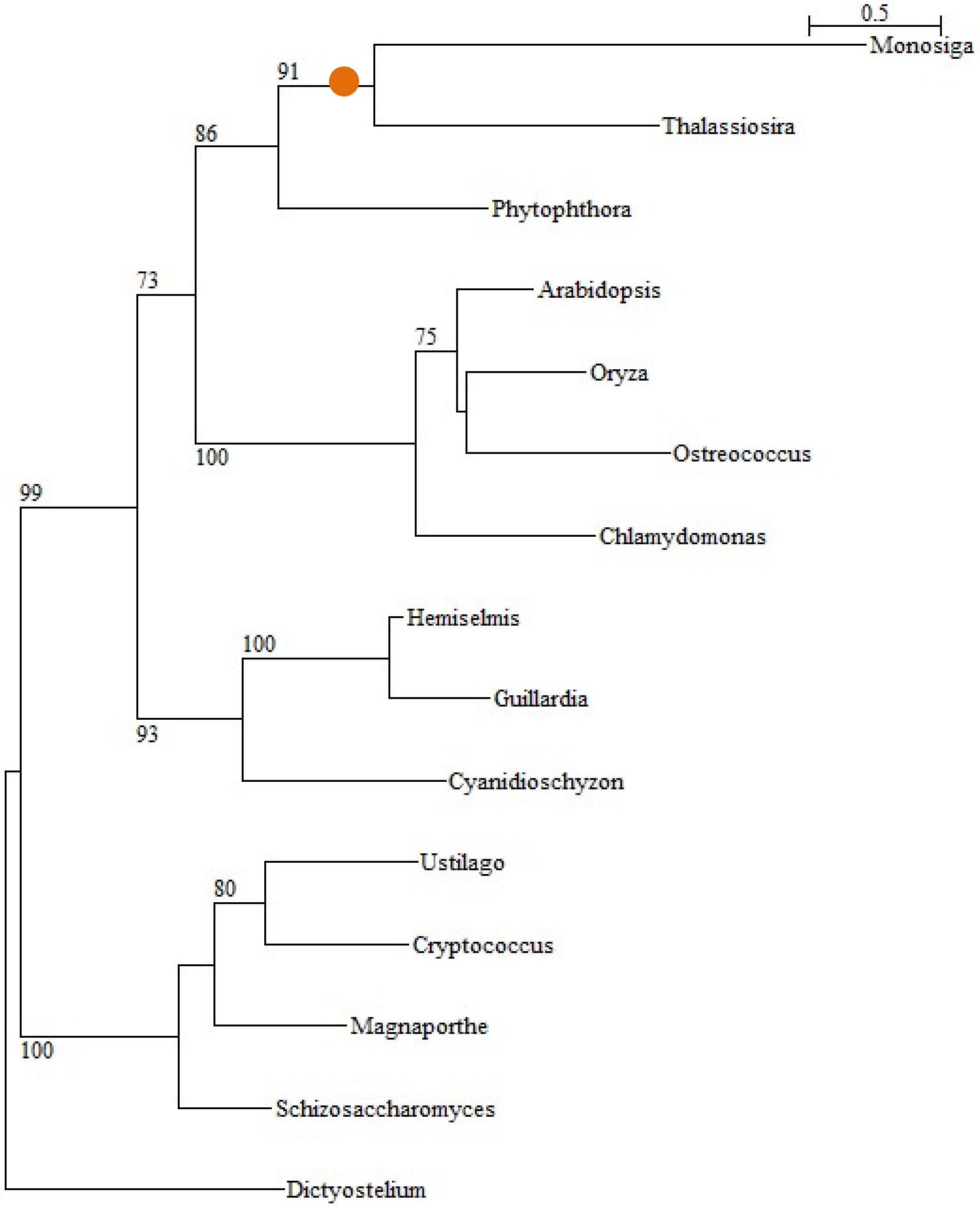

Figure S62. Phylogeny of CorA metal ion transporter (GI No. 167516550).

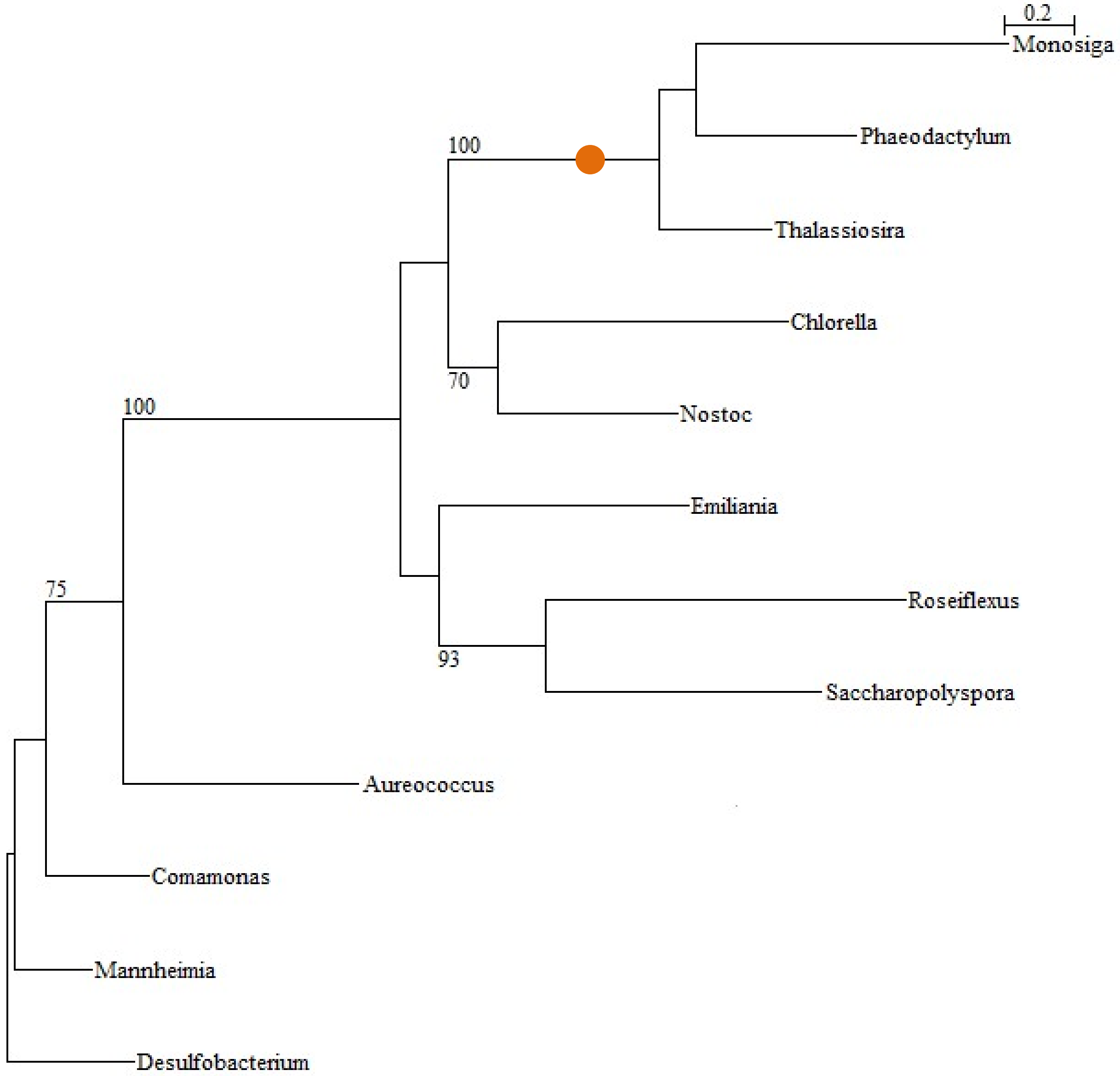

Figure S63. Phylogeny of one hypothetical protein (GI No. 167516062).

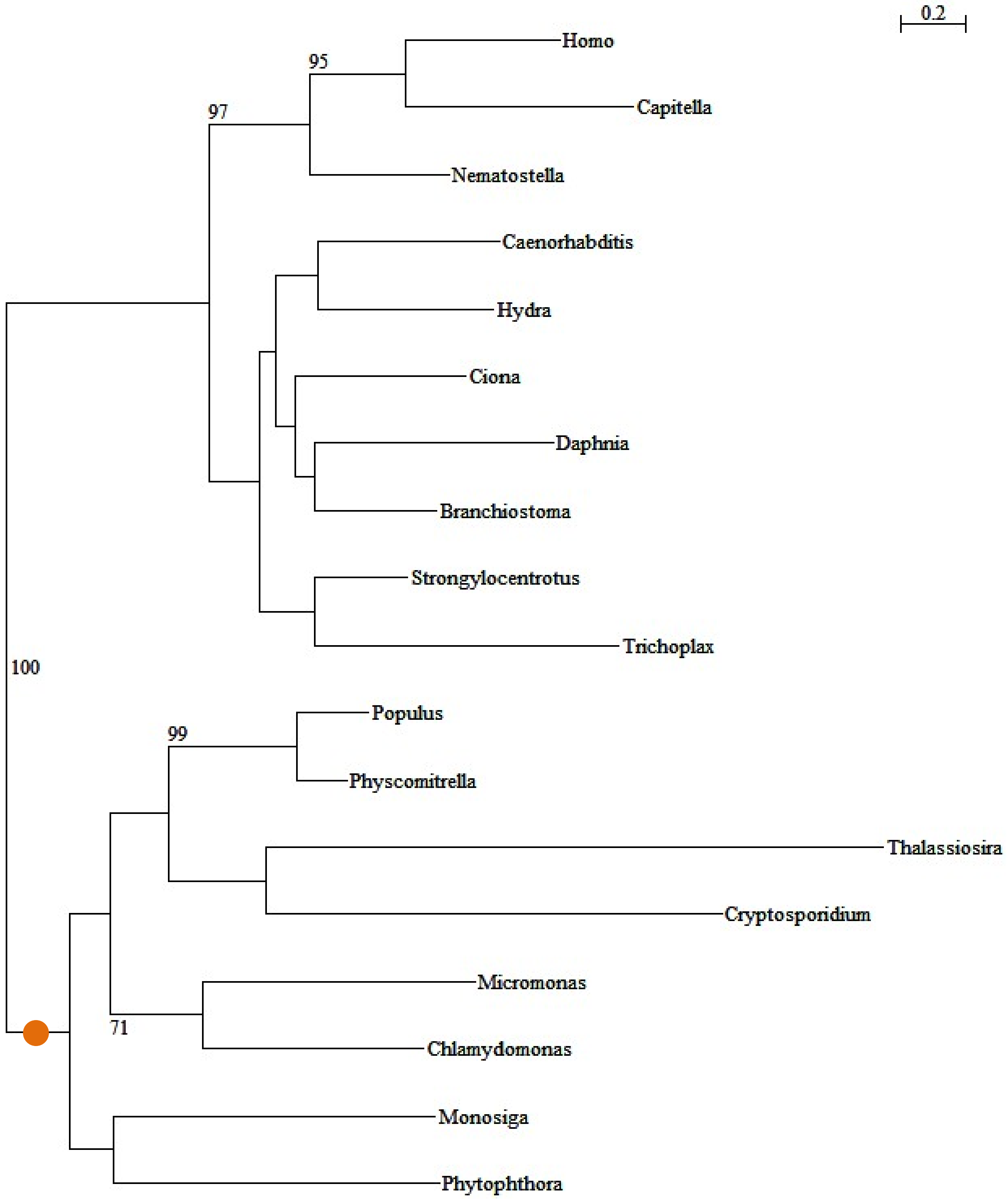

Figure S64. Phylogeny of one hypothetical protein (GI No. 167519256).

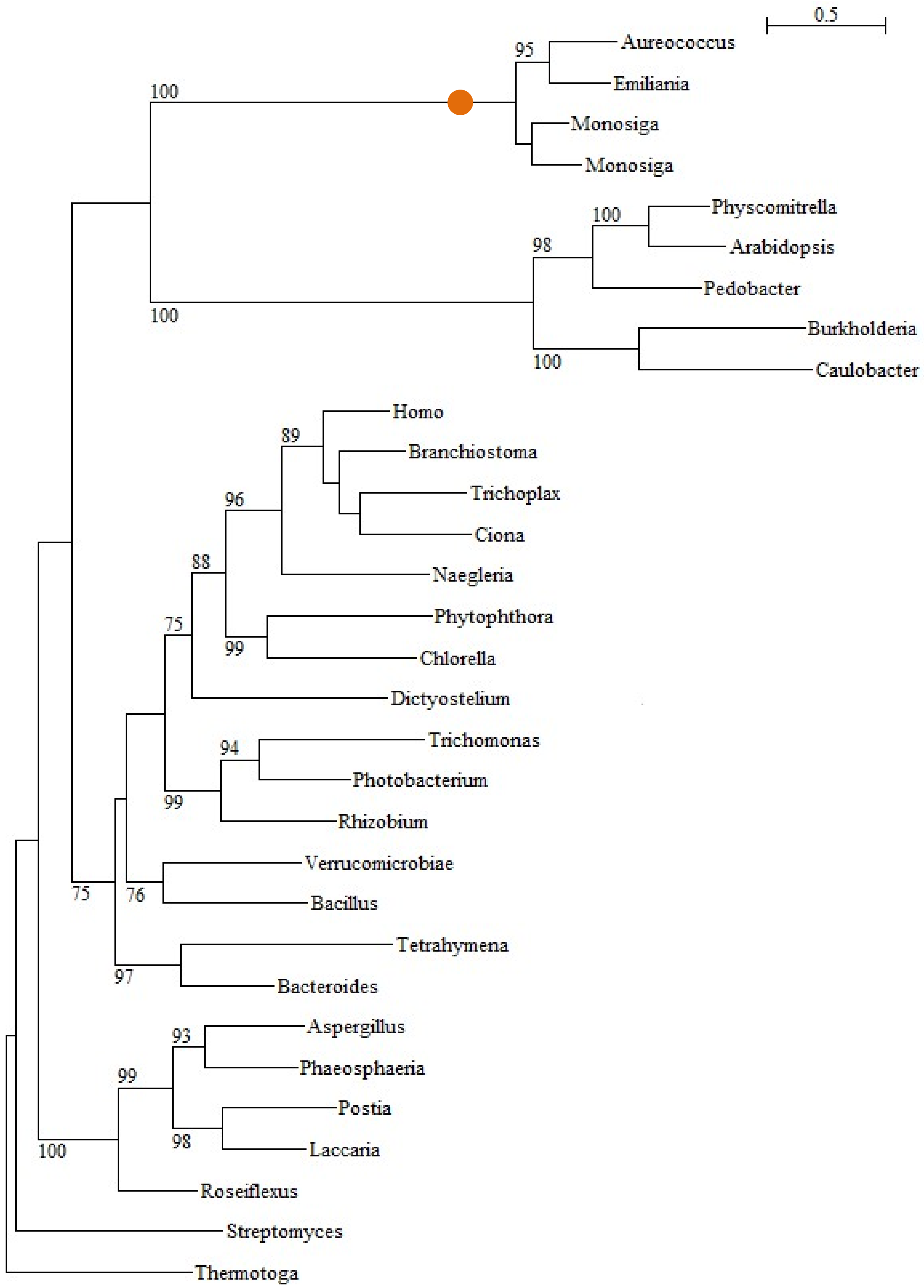

Figure S65. Phylogeny of beta-mannosidase (Gi No. 167523877 and 167522846).

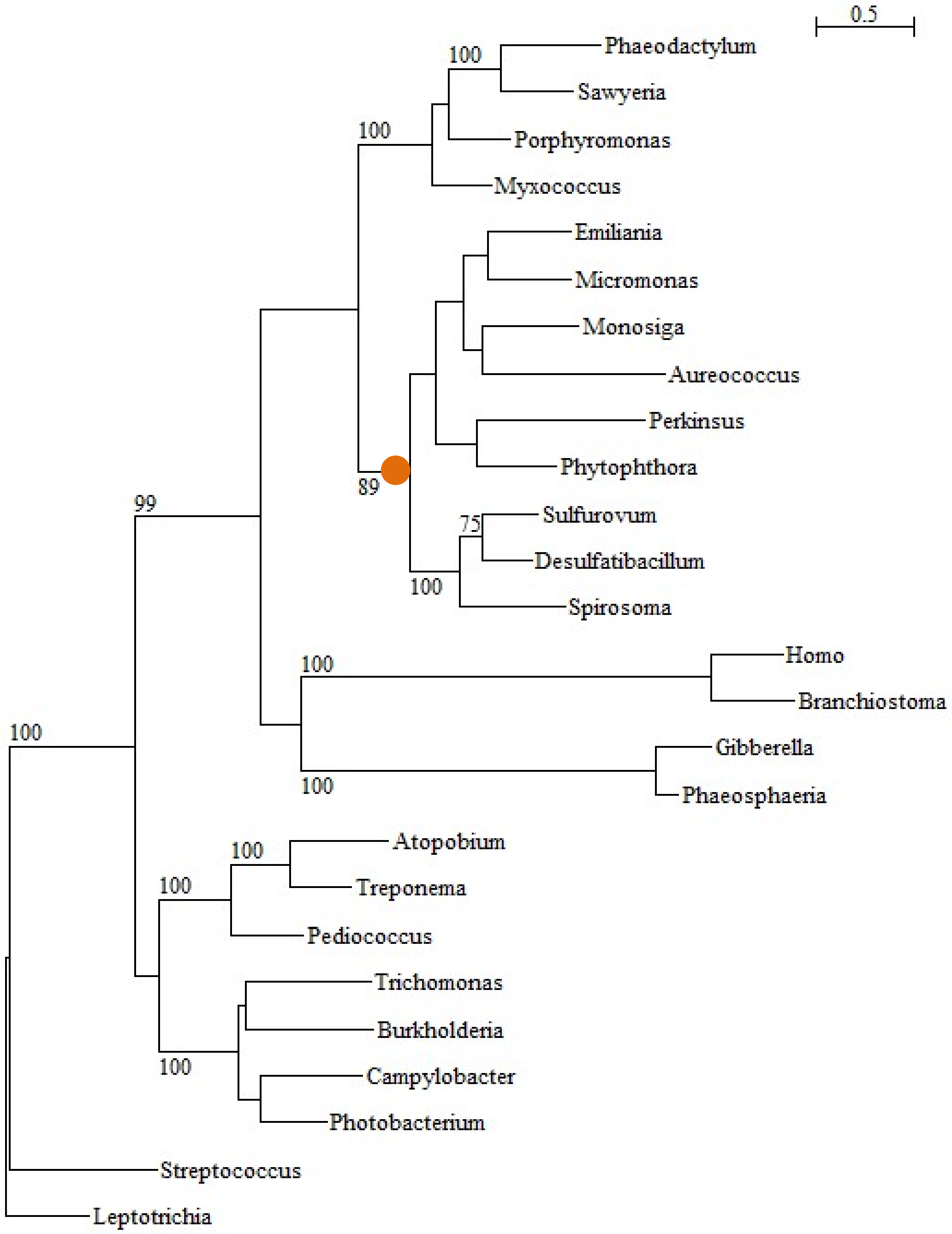

Figure S66. Phylogeny of peptidase U34 dipeptidase (GI No. 167524214).

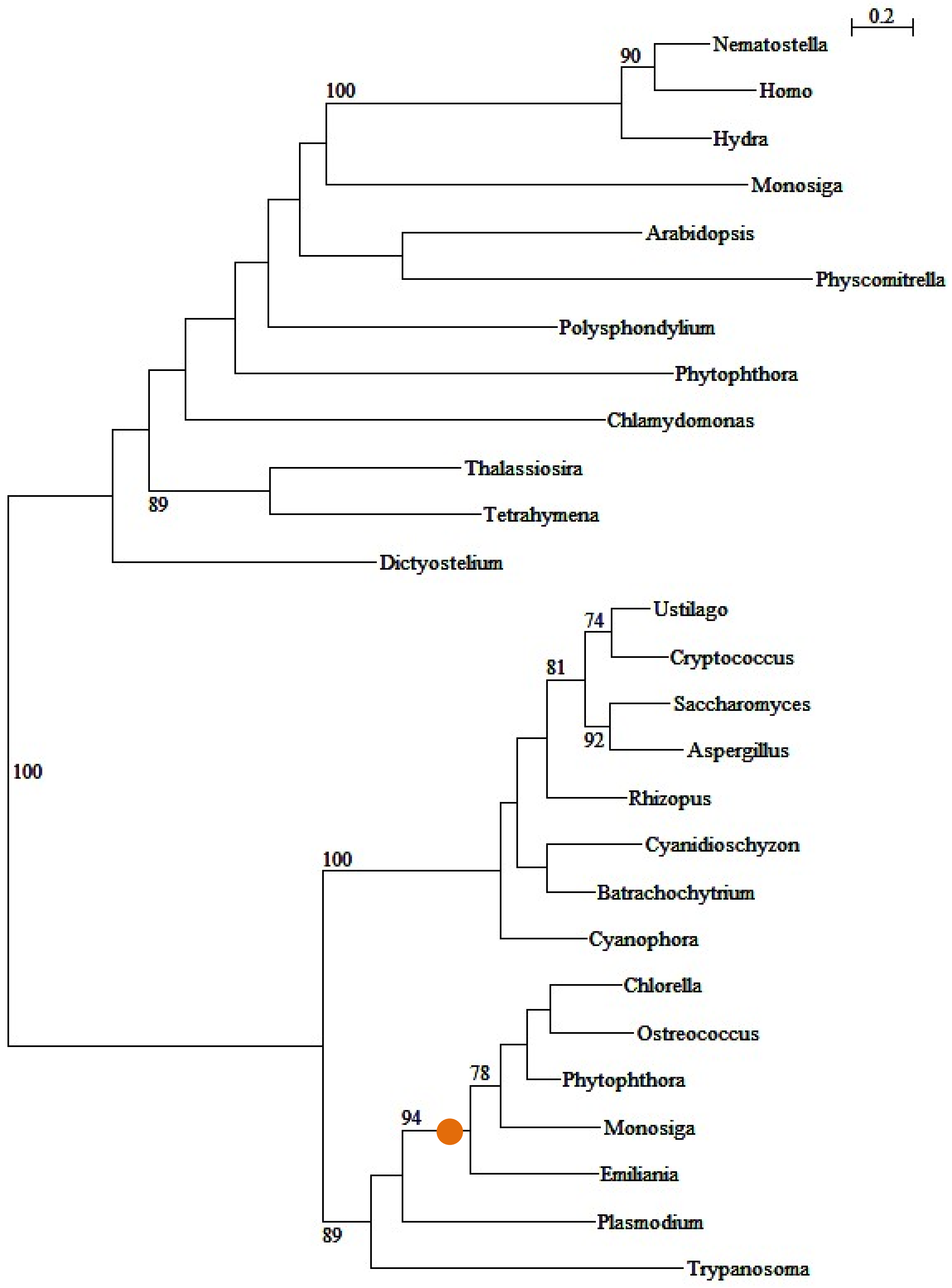

Figure S67. Phylogeny of mitochondrial carrier protein (GI No. 167524336).

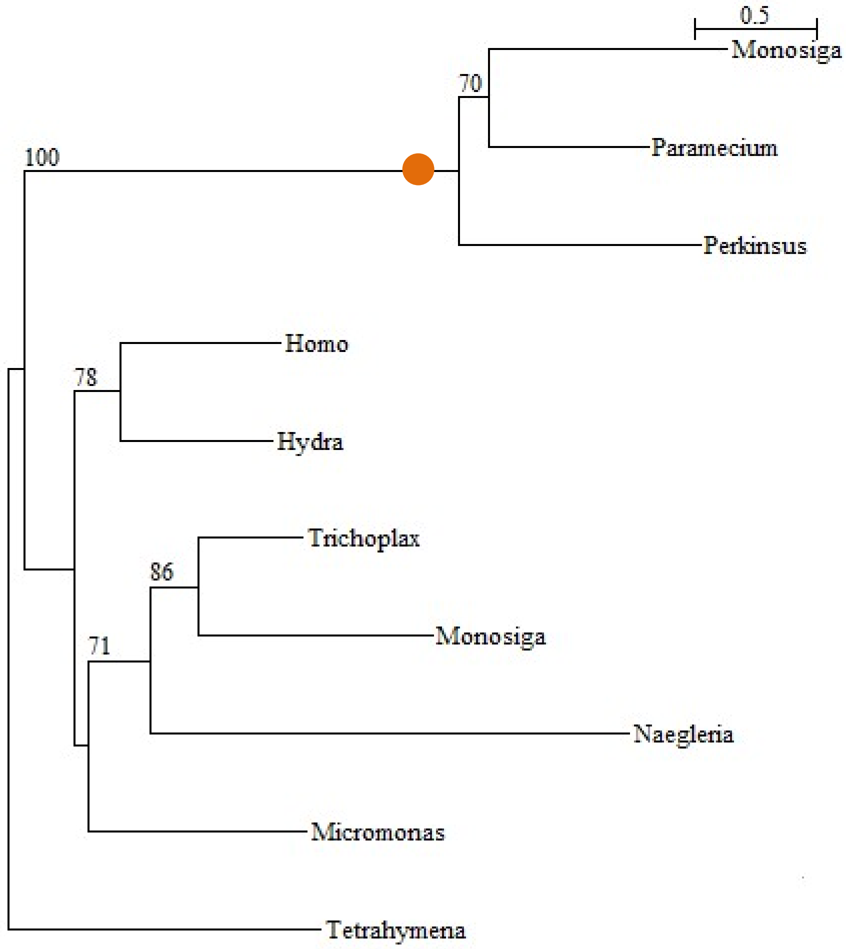

Figure S68. Phylogeny of acid ceramidase (GI No. 167517231).

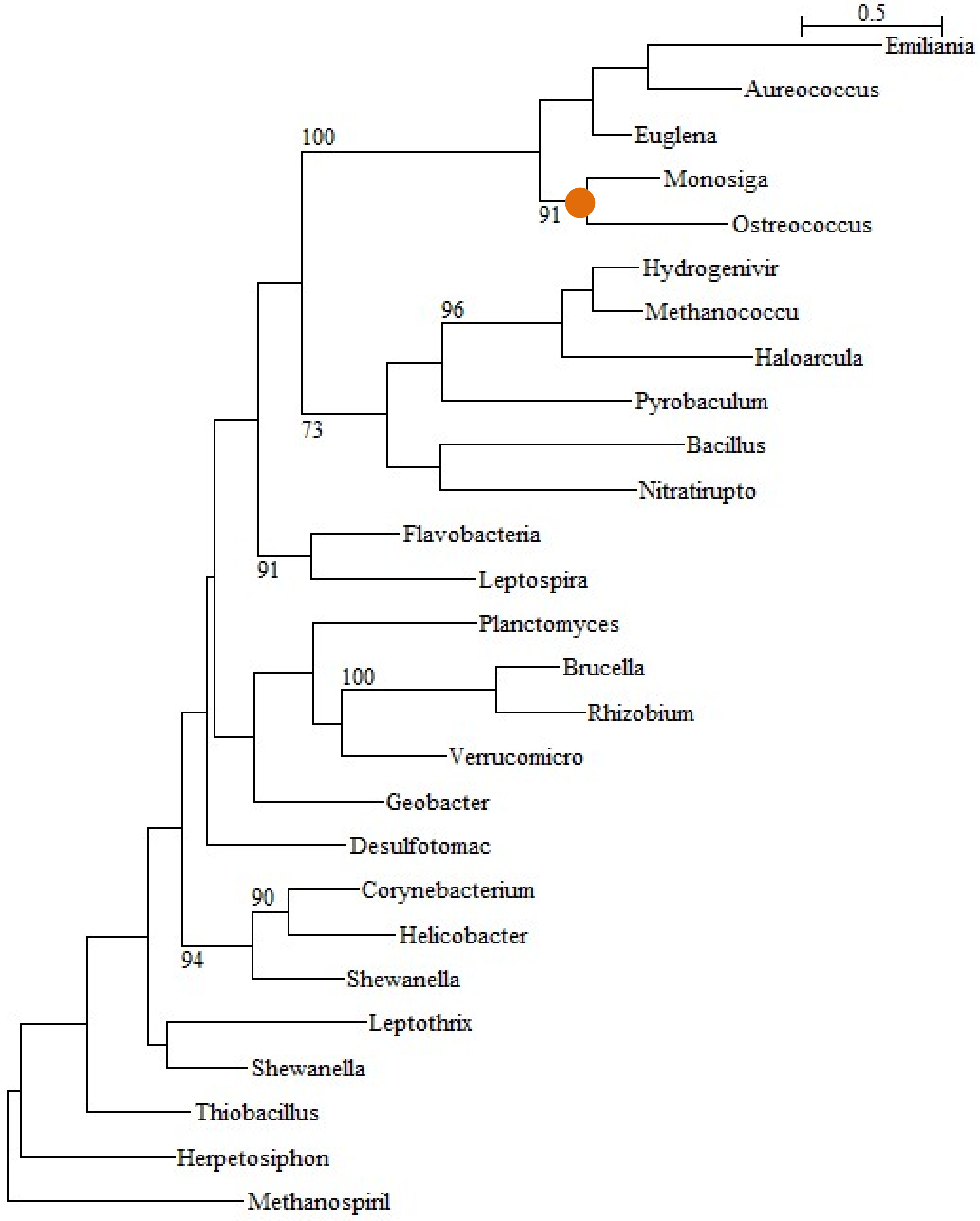

Figure S69. Phylogeny of one hypothetical protein (GI No. 167525567).

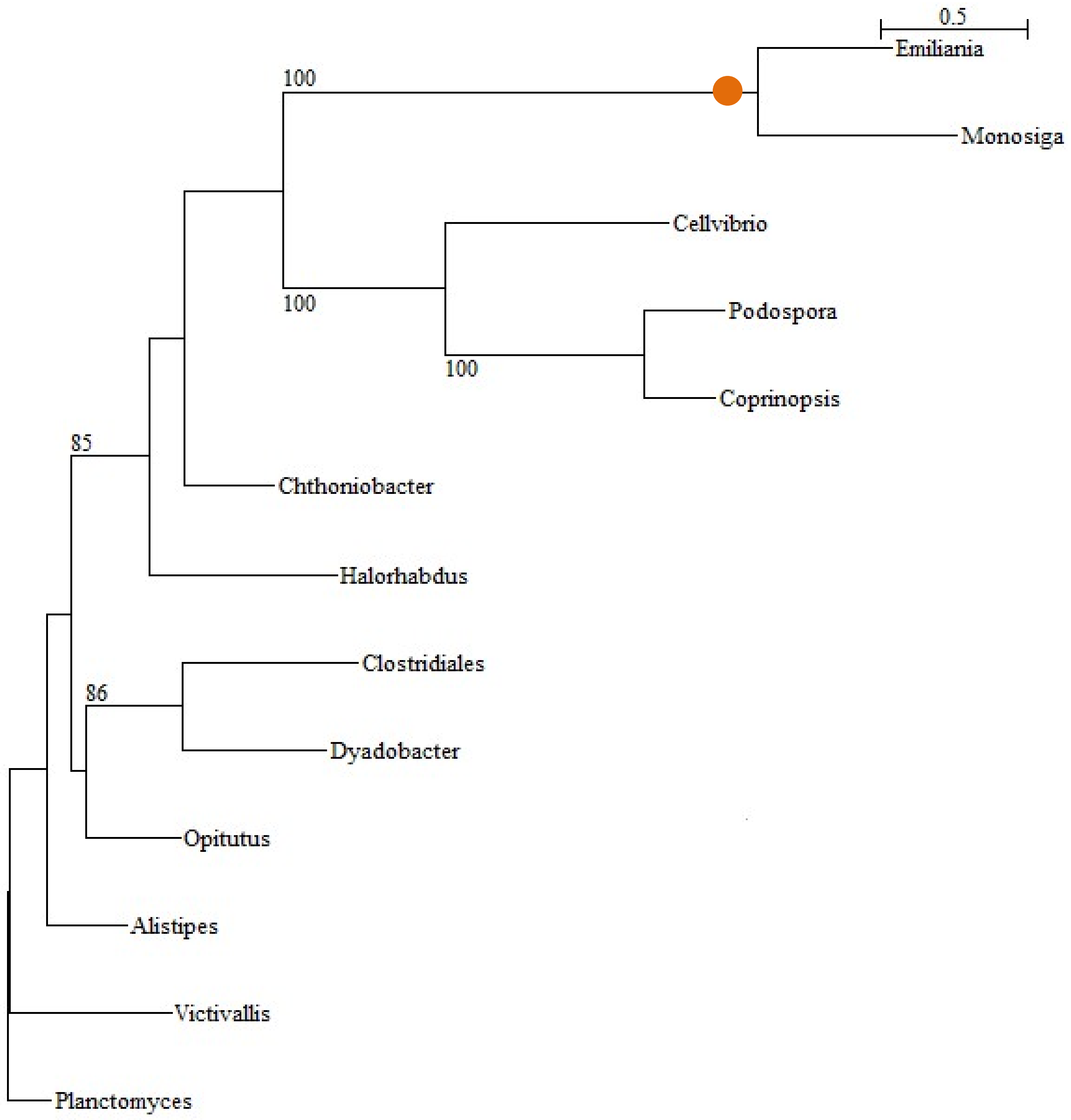

Figure S70. Phylogeny of one hypothetical protein (GI No. 167527021).

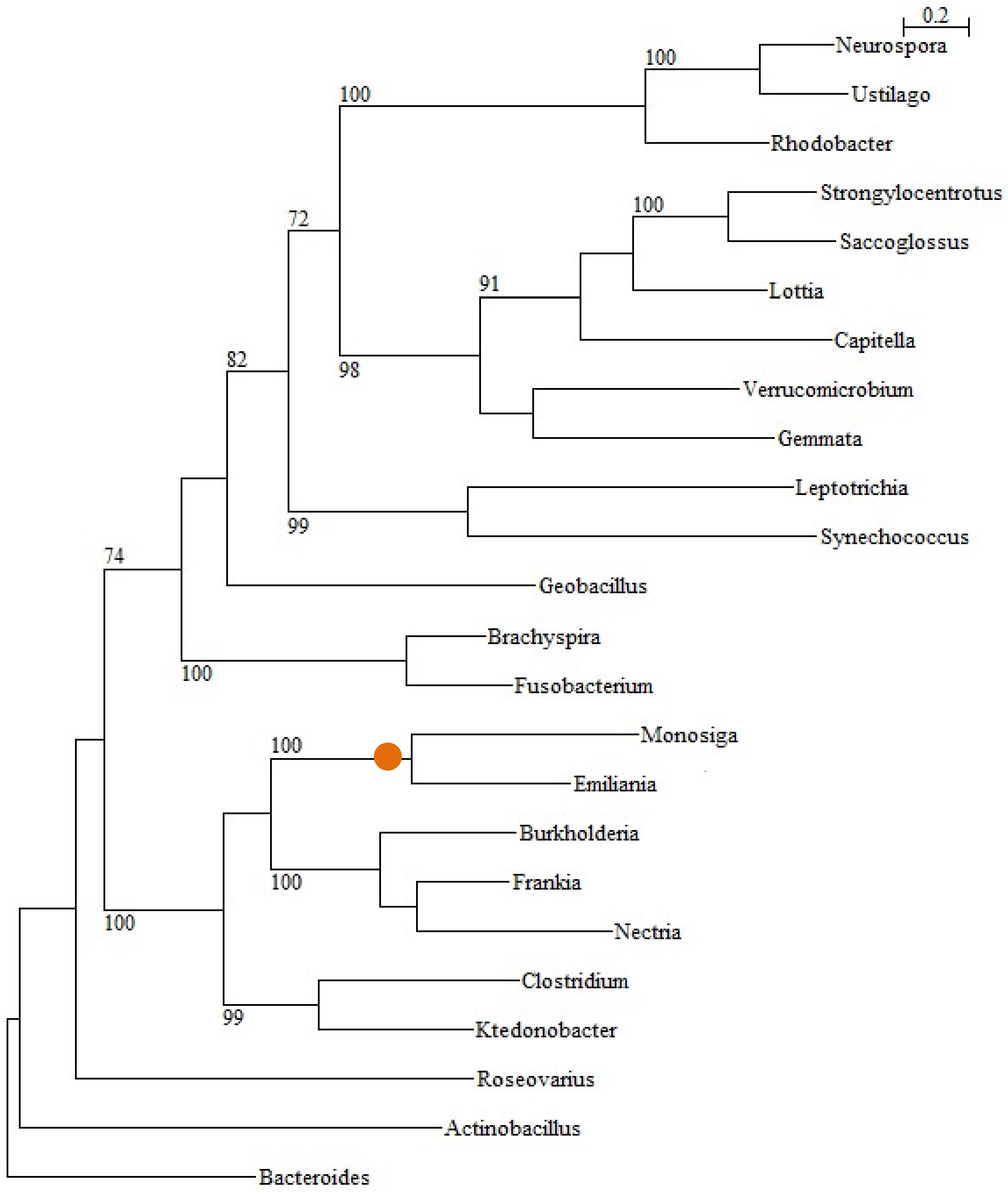

Figure S71. Phylogeny of sulfatase (GI No. 167527083).

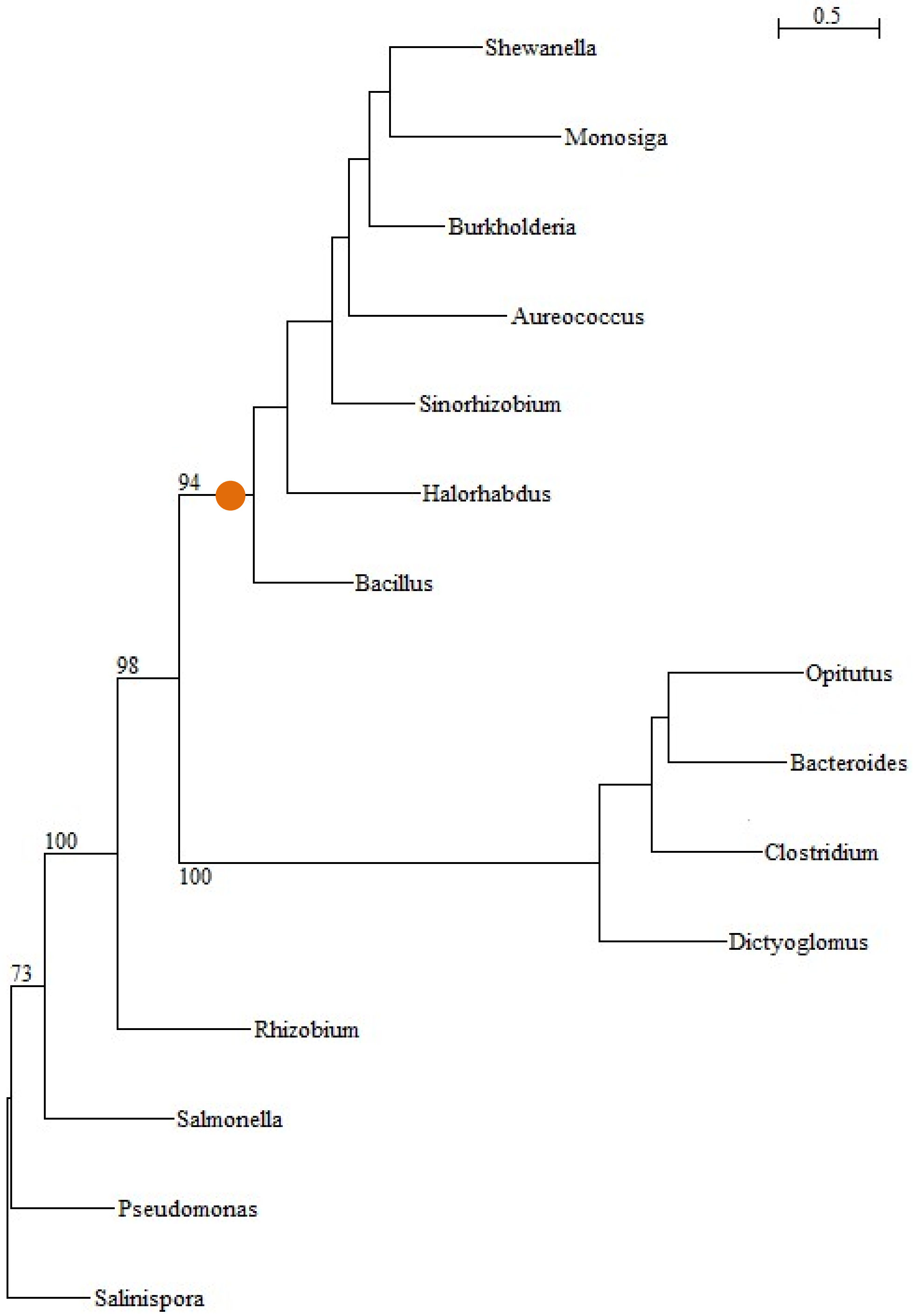

Figure S72. Phylogeny of N-acylglucosamine 2-epimerase (GI No. 167527127).

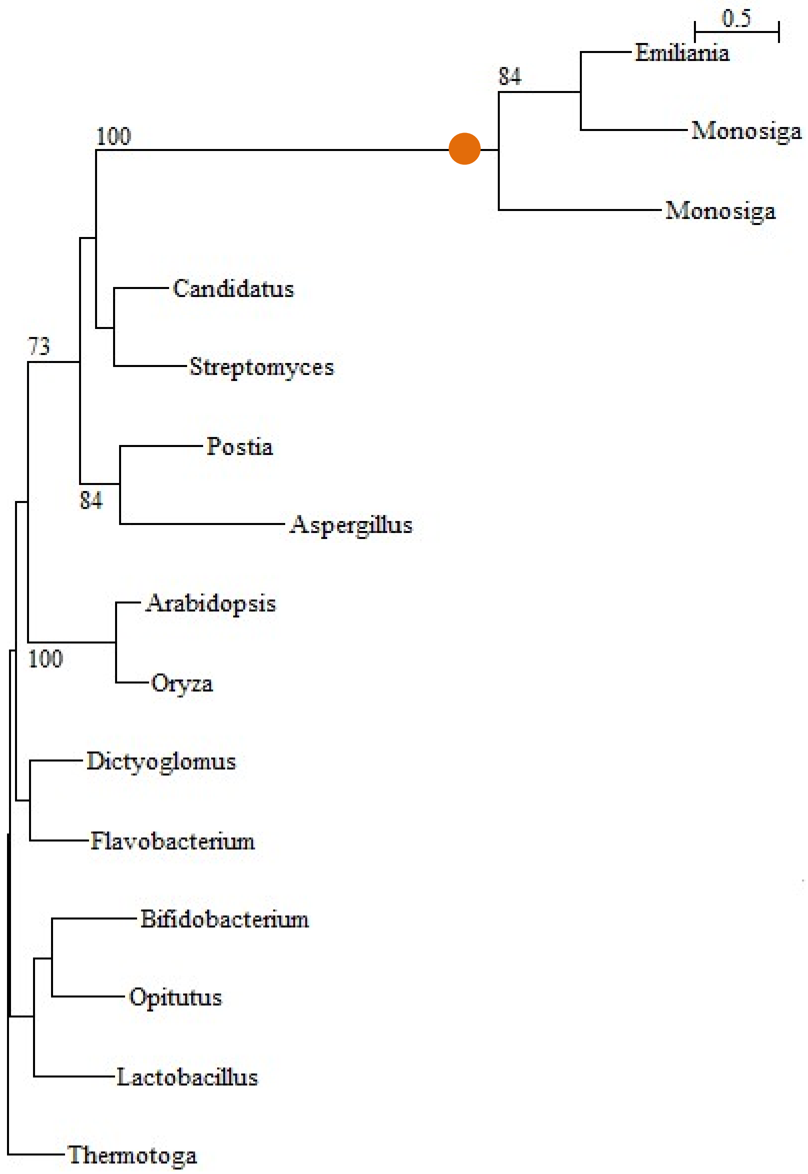

Figure S73. Phylogeny of alpha-L-arabinofuranosidase (GI No. 167533053 and 167519743).

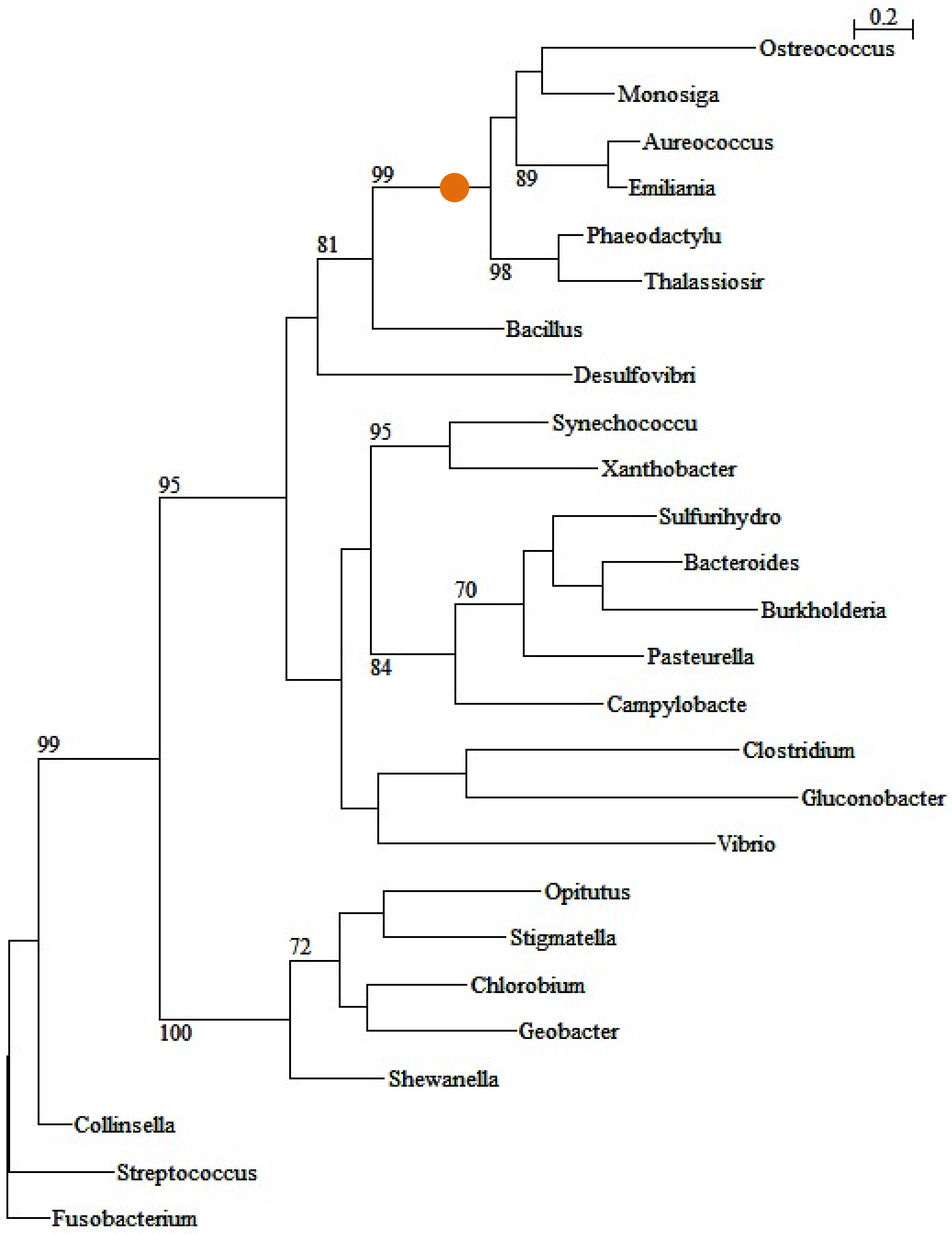

Figure S74. Phylogeny of one hypothetical protein (GI No. 167534047).

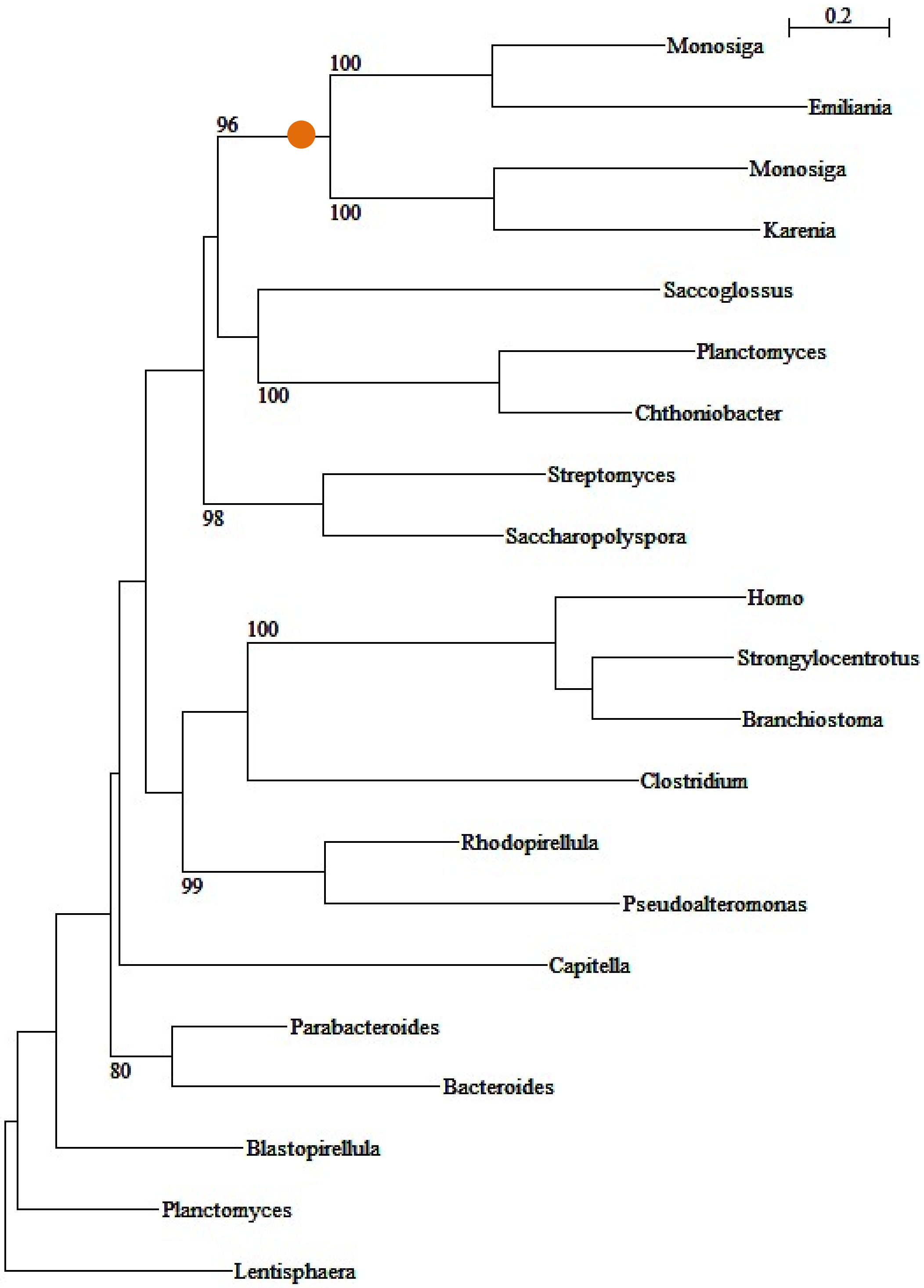

Figure S75. Phylogeny of sialidase (Gi No. 167535145 and 167534965).

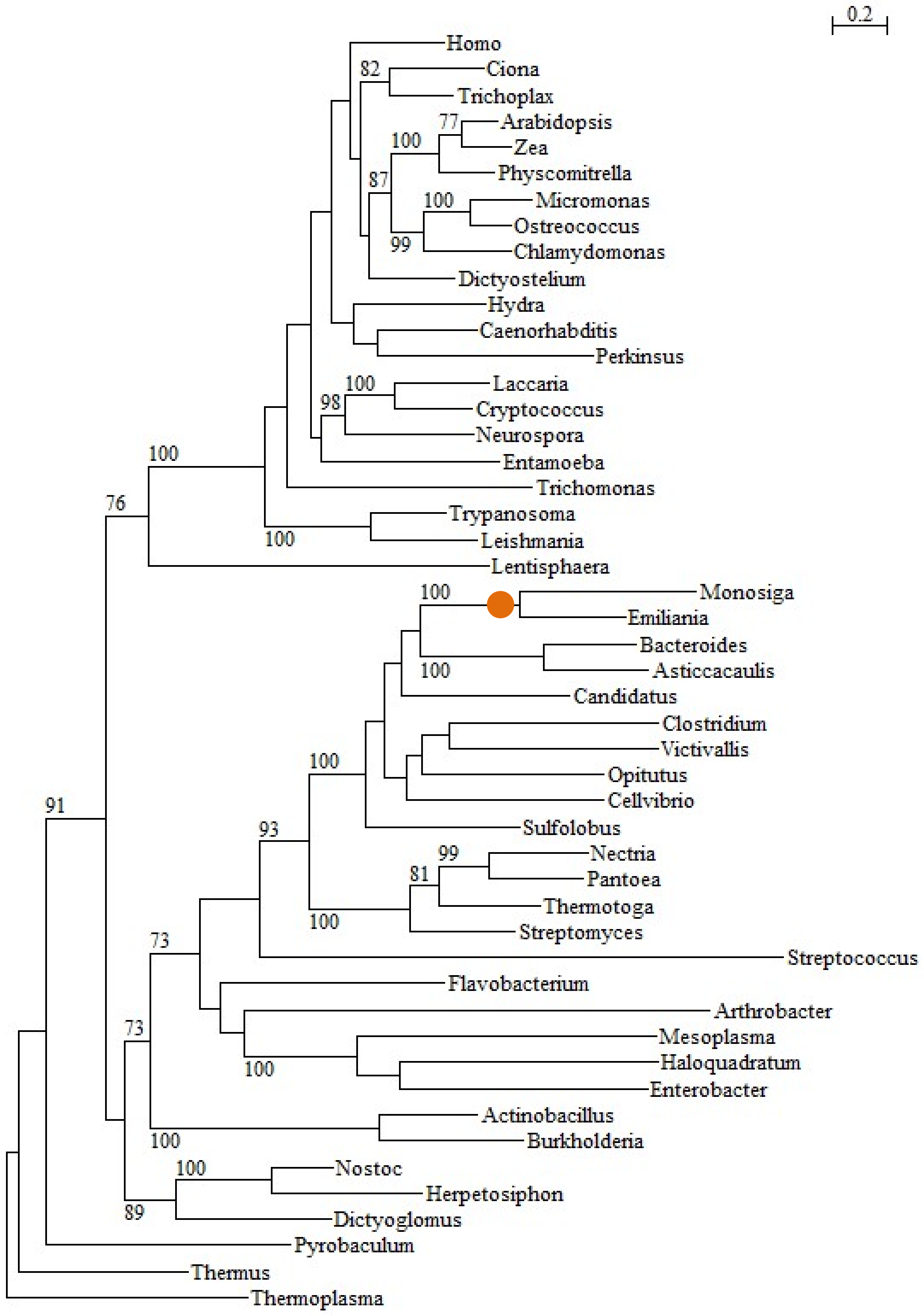

Figure S76. Phylogeny of alpha-glucosidase (GI No. 167536698).

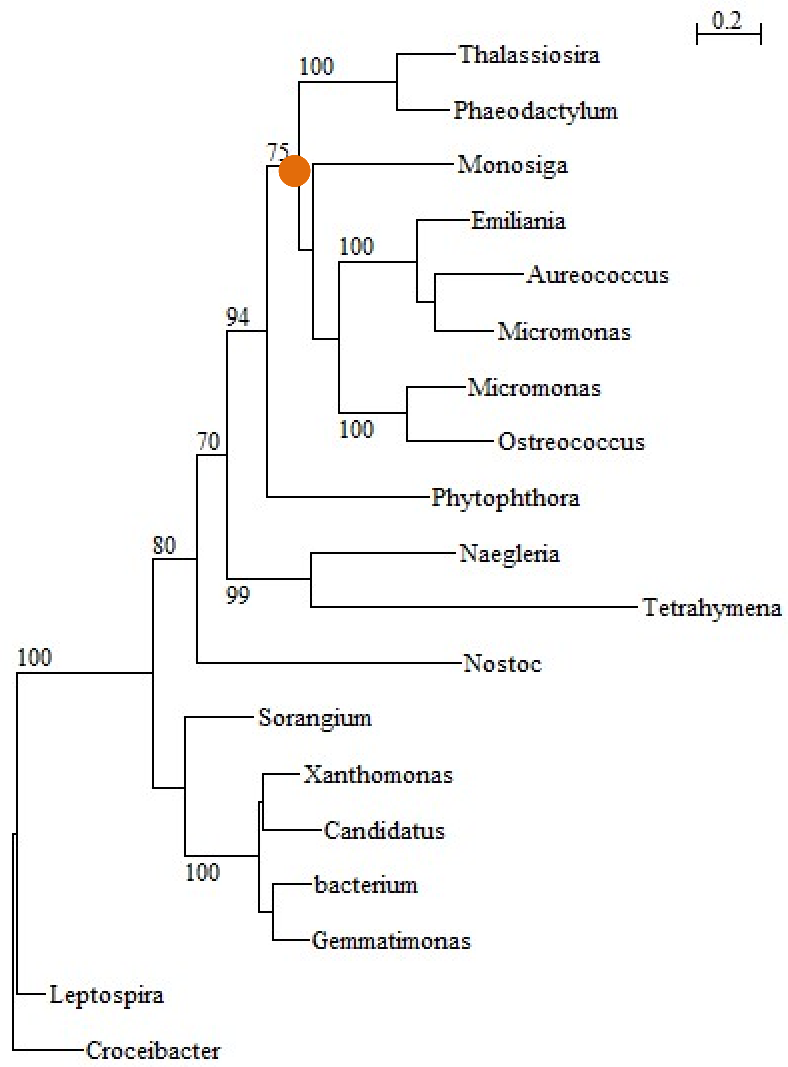

Figure S77. Phylogeny of oxidoreductase (GI No. 167538446).

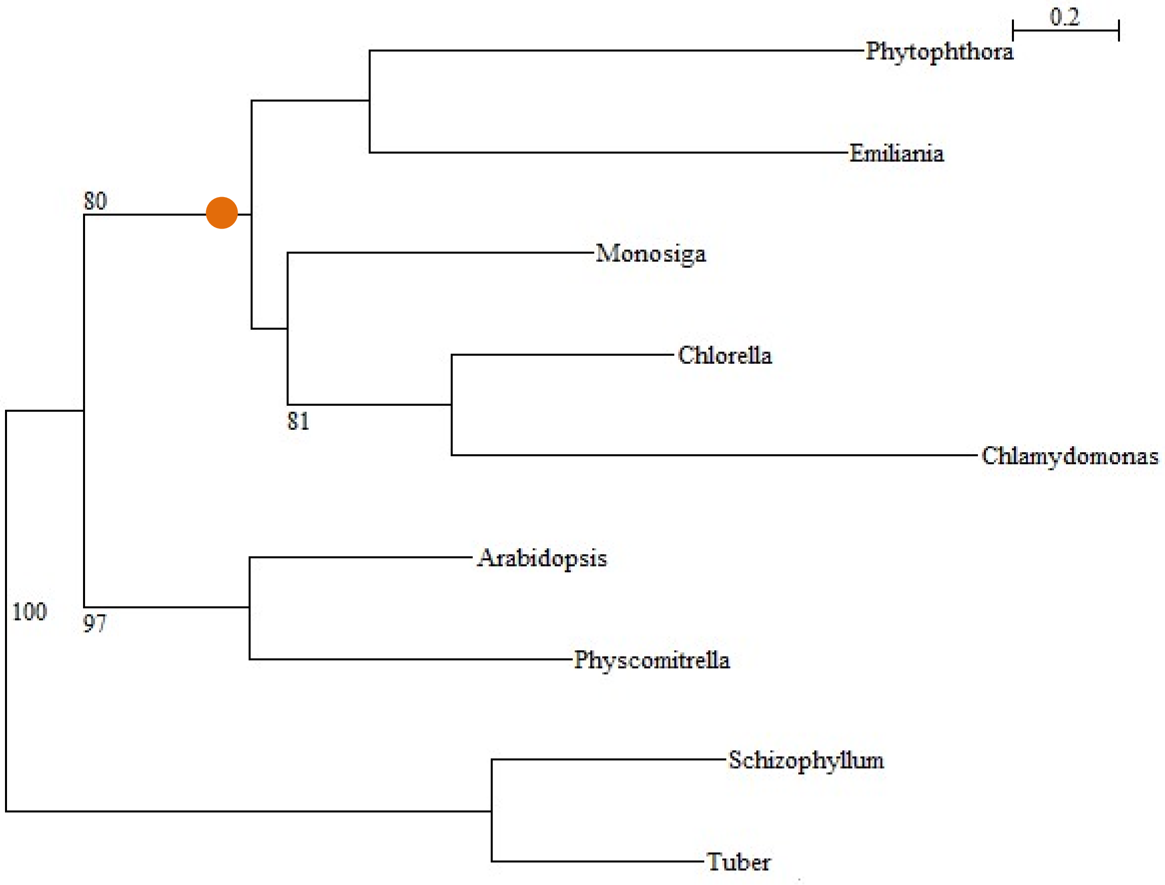

Figure S78. Phylogeny of one hypothetical protein (GI No. 167516576).

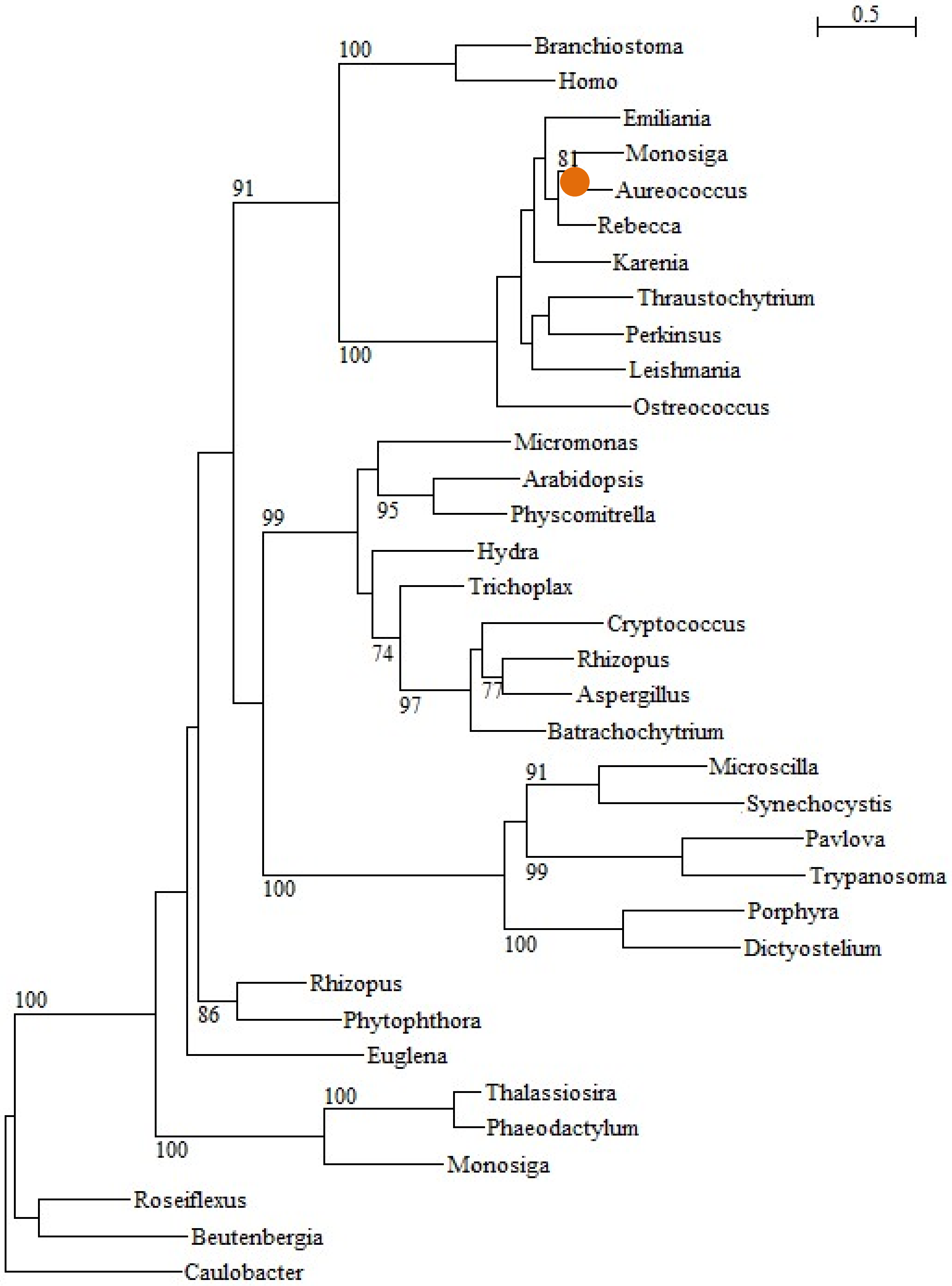

Figure S79. Phylogeny of delta-5 desaturase (GI No. 167518353).

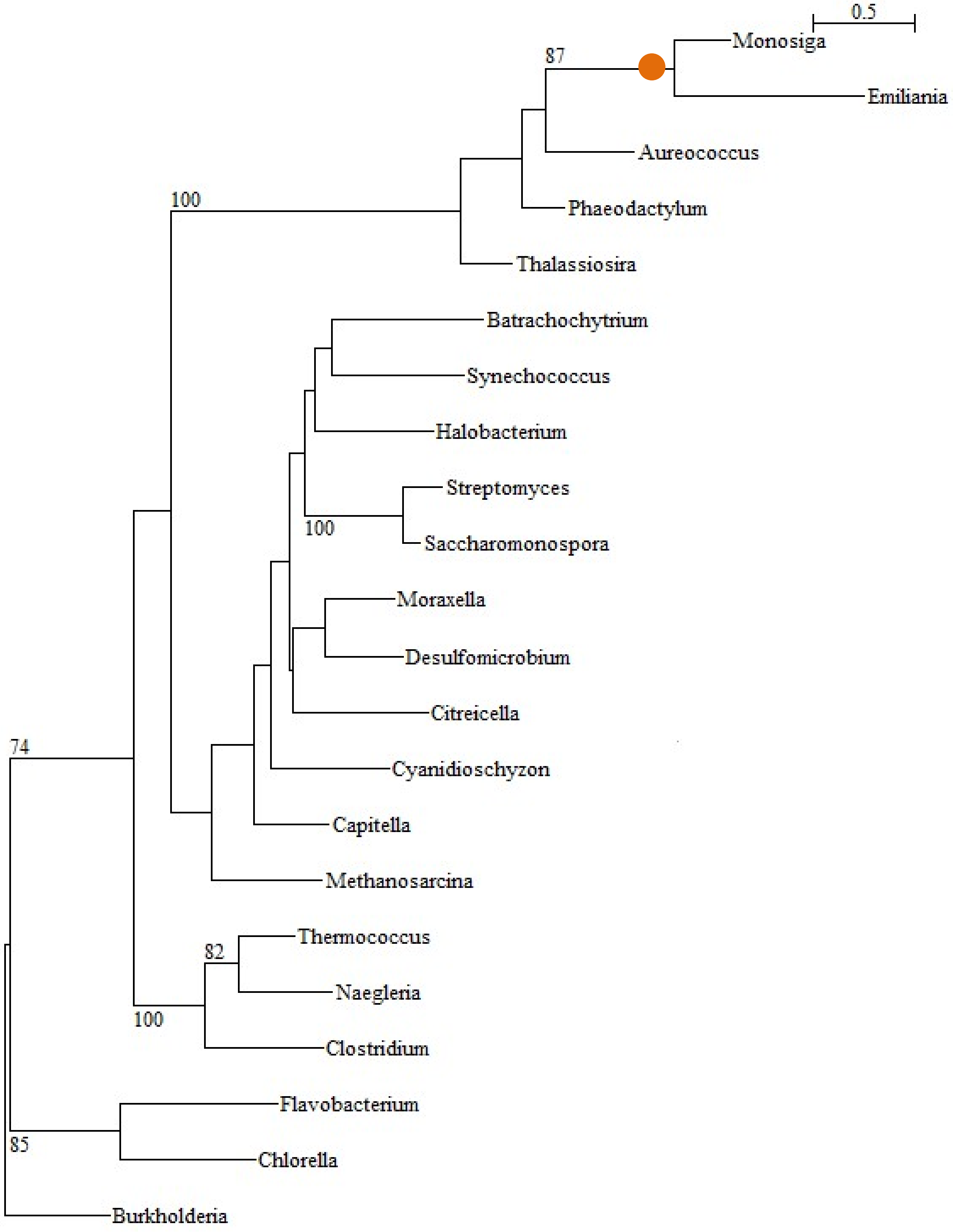

Figure S80. Phylogeny of one hypothetical protein (GI No. 167520576).

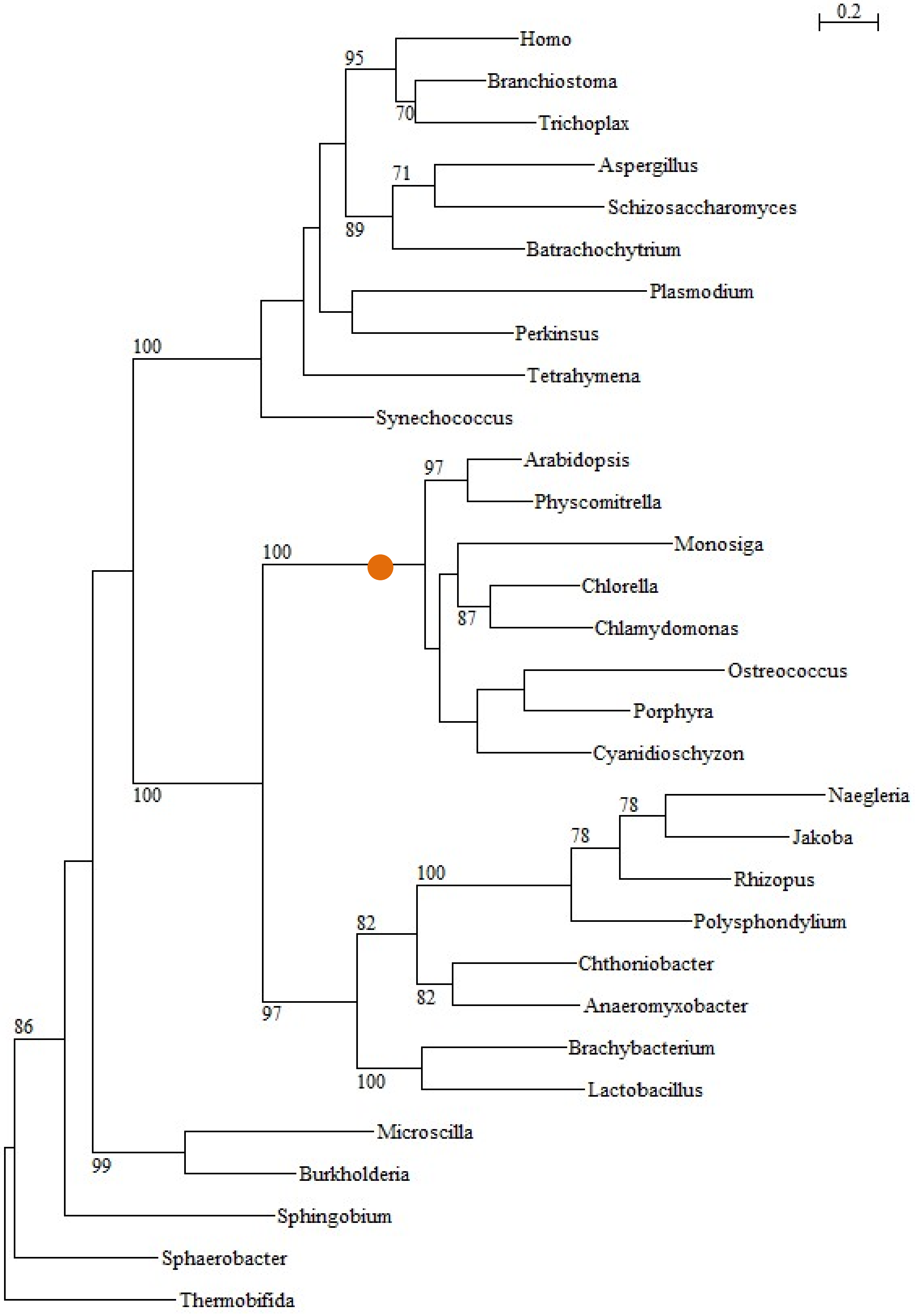

Figure S81. Phylogeny of monodehydroascorbate reductase (GI No. 167522575).

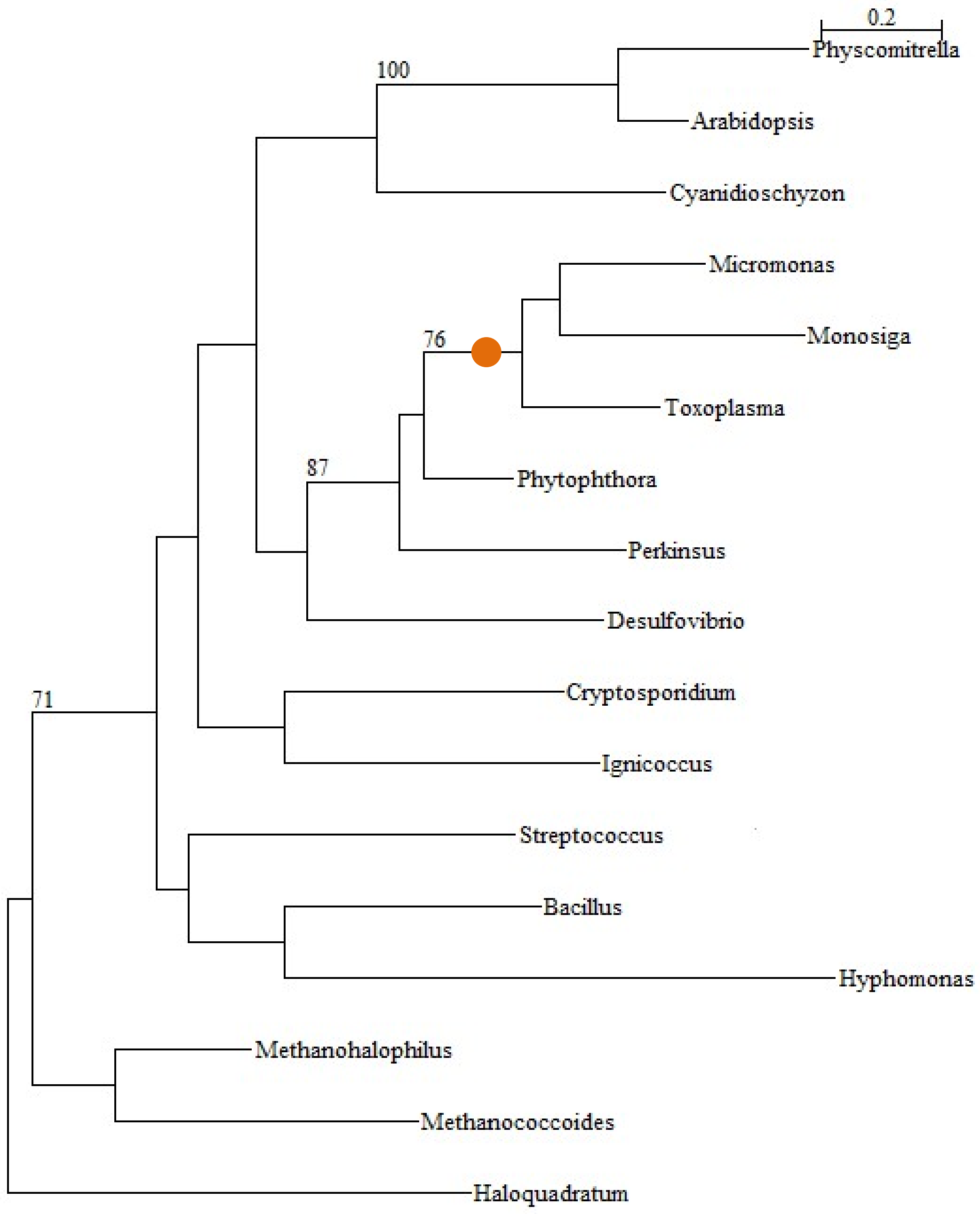

Figure S82. Phylogeny of one hypothetical protein (GI No. 167522591).

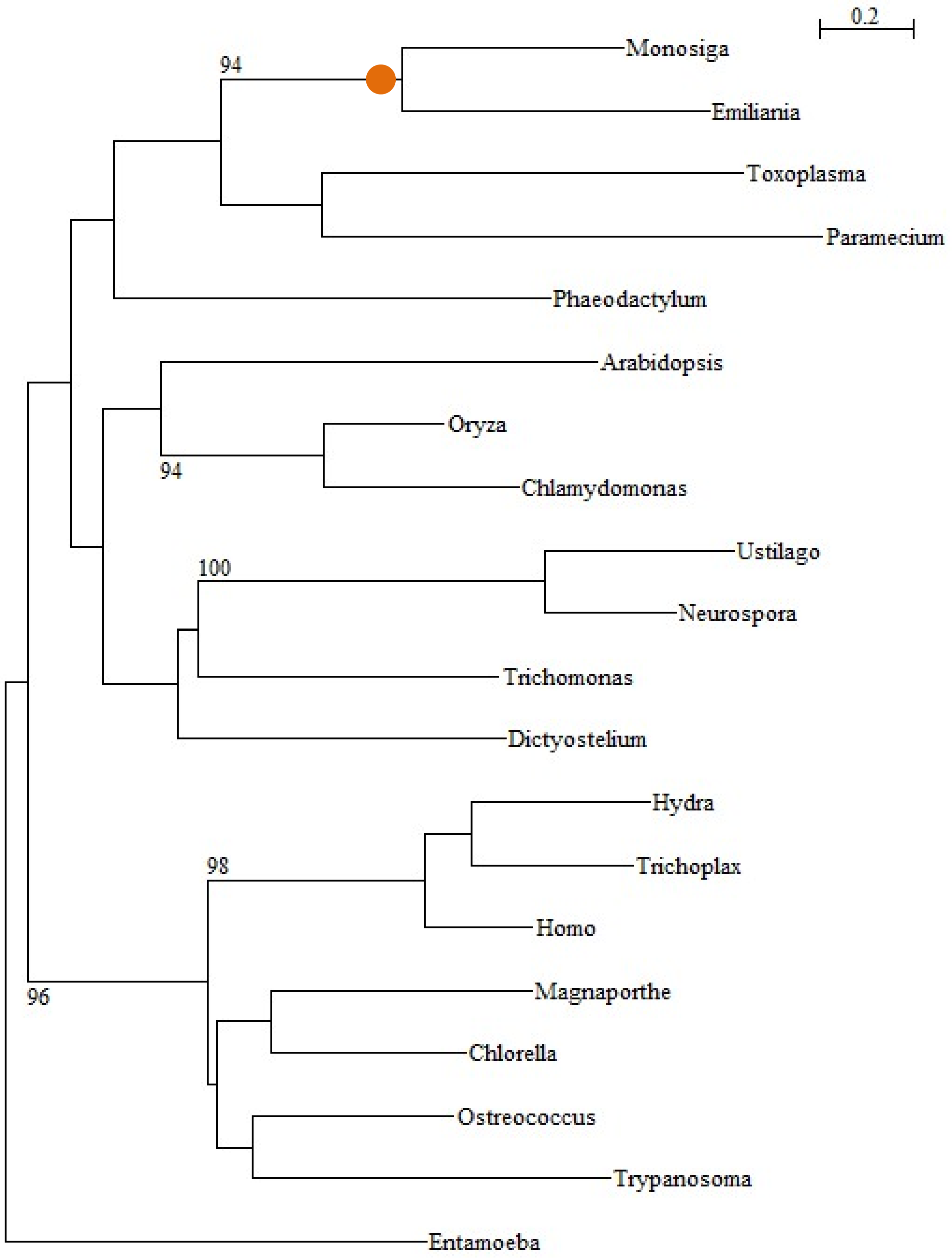

Figure S83. Phylogeny of protein phosphatase 2C (GI No. 167522497).

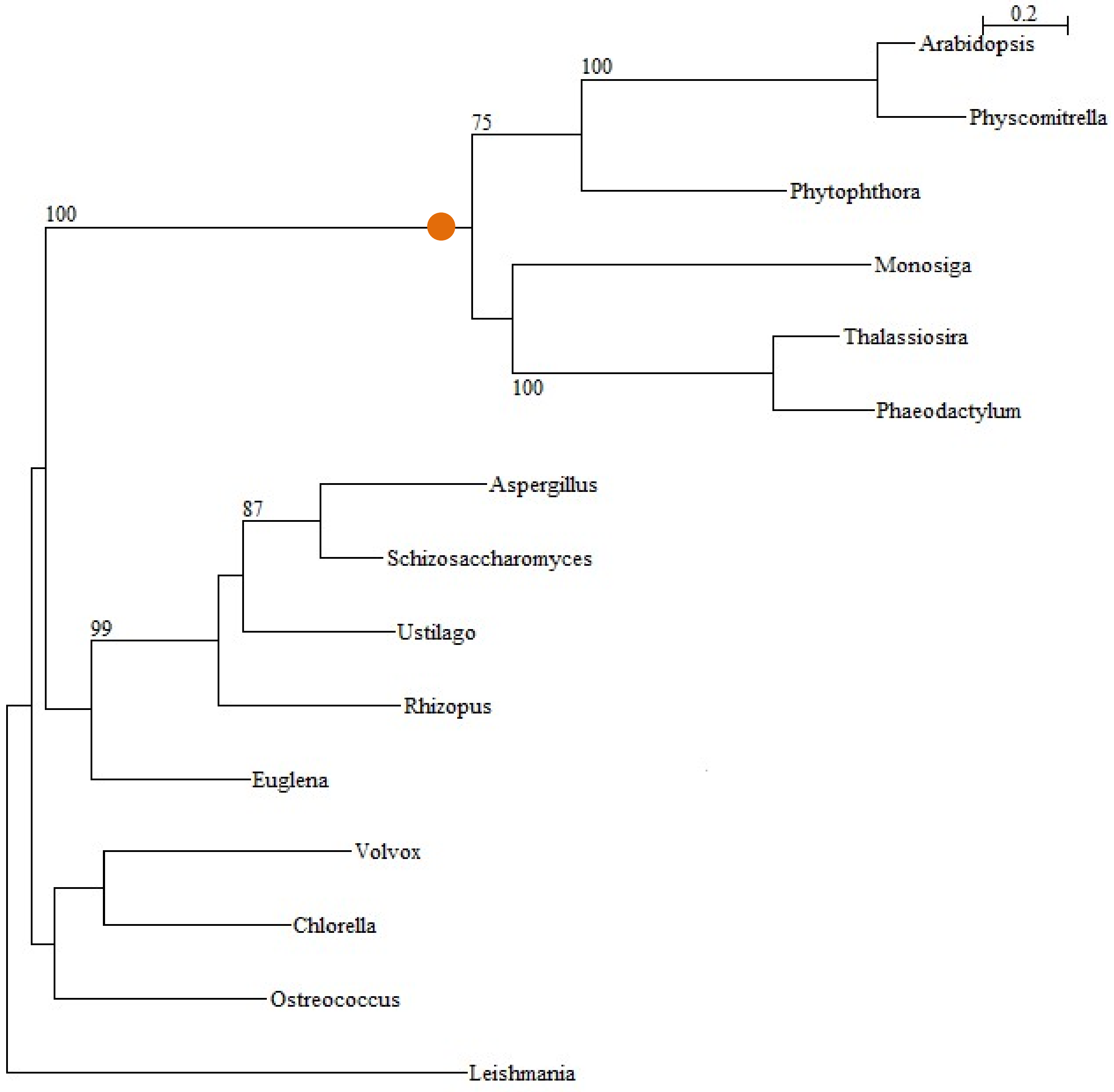

Figure S84. Phylogeny of phosphatidate-sterol O-acyltransferase (GI No. 167525286).

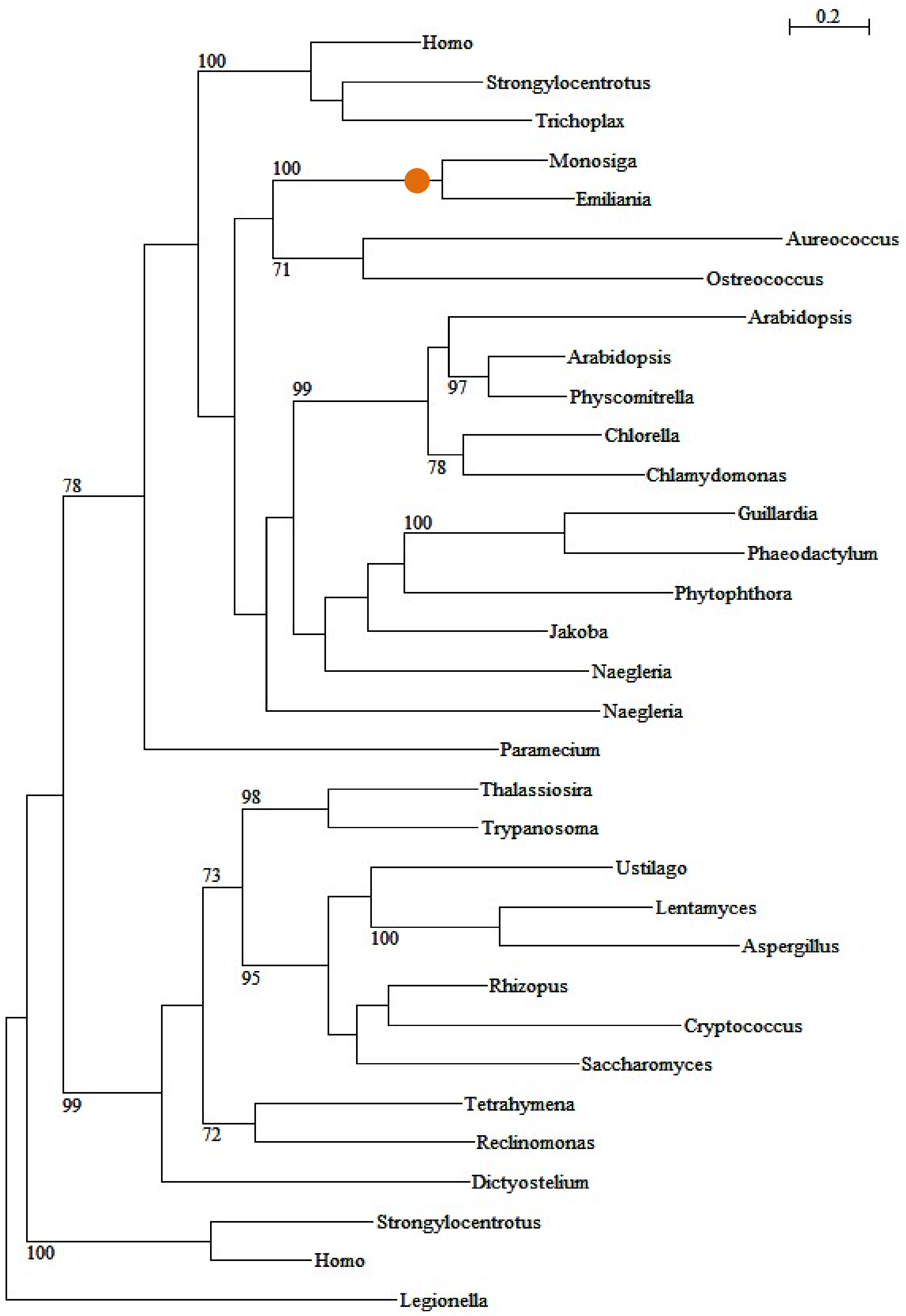

Figure S85. Phylogeny of serine carboxypeptidase (GI No. 167526595).

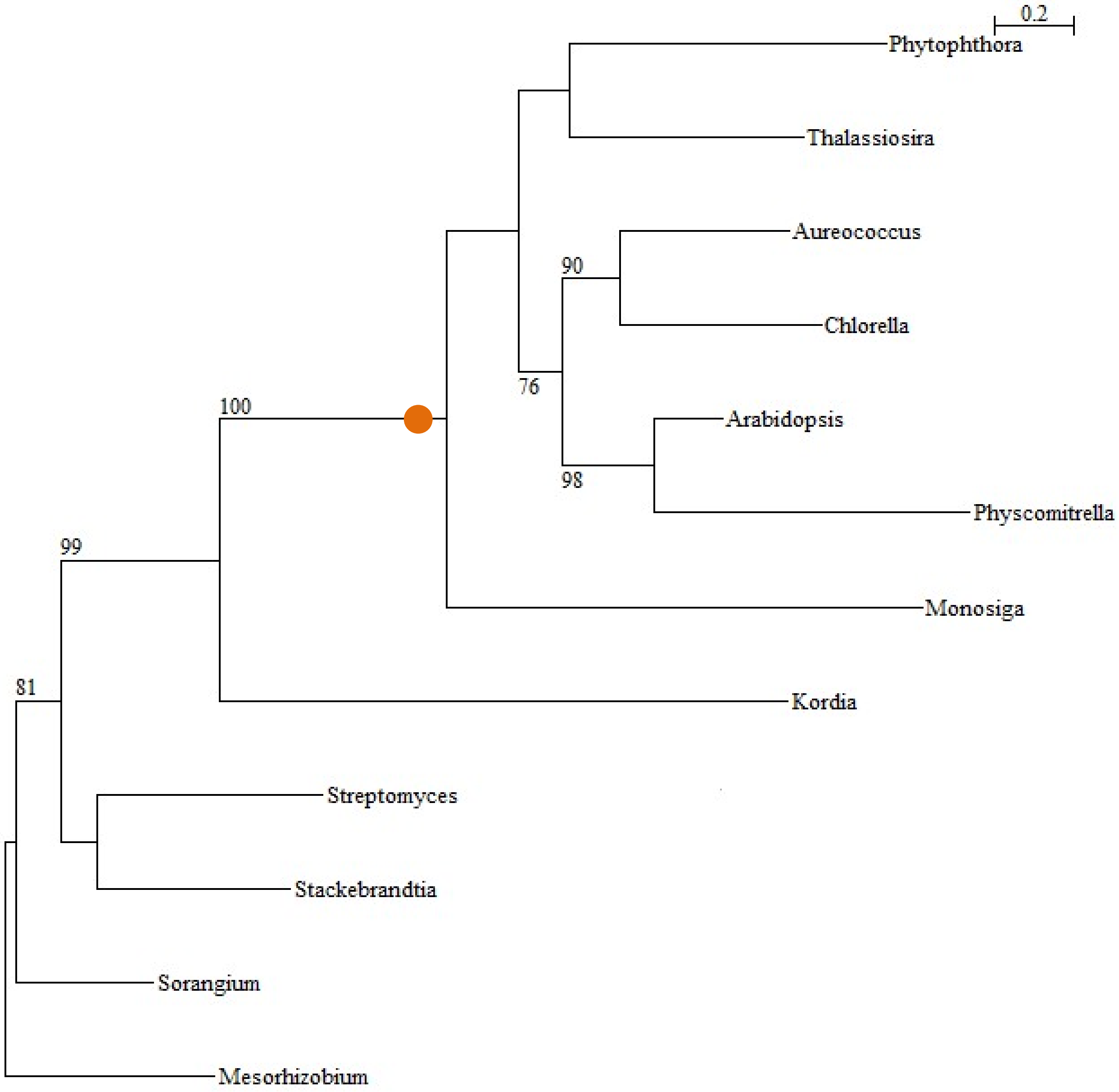

Figure S86. Phylogeny of ion channel protein (GI No. 167534102).

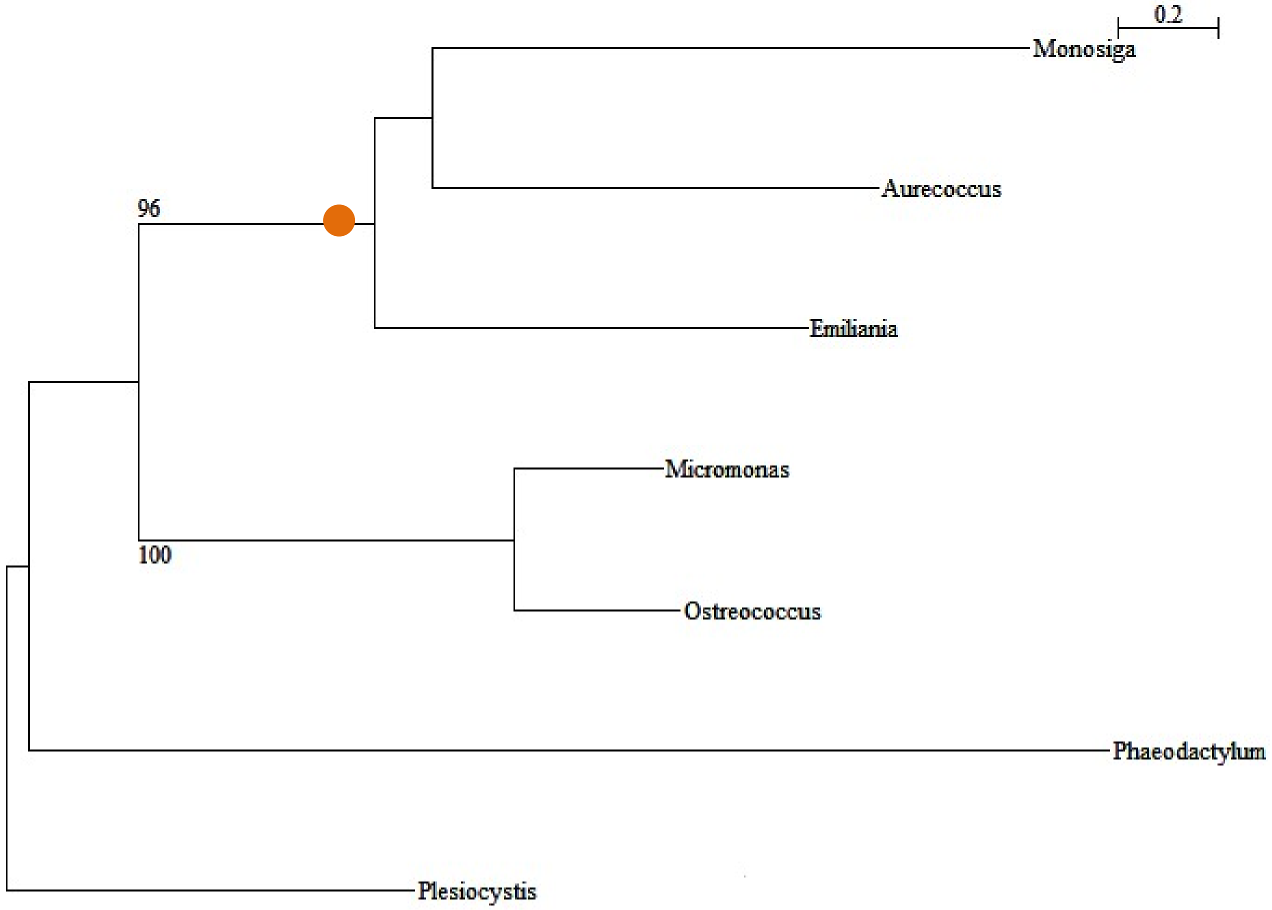

Figure S87. Phylogeny of one hypothetical protein (GI No. 167534124).

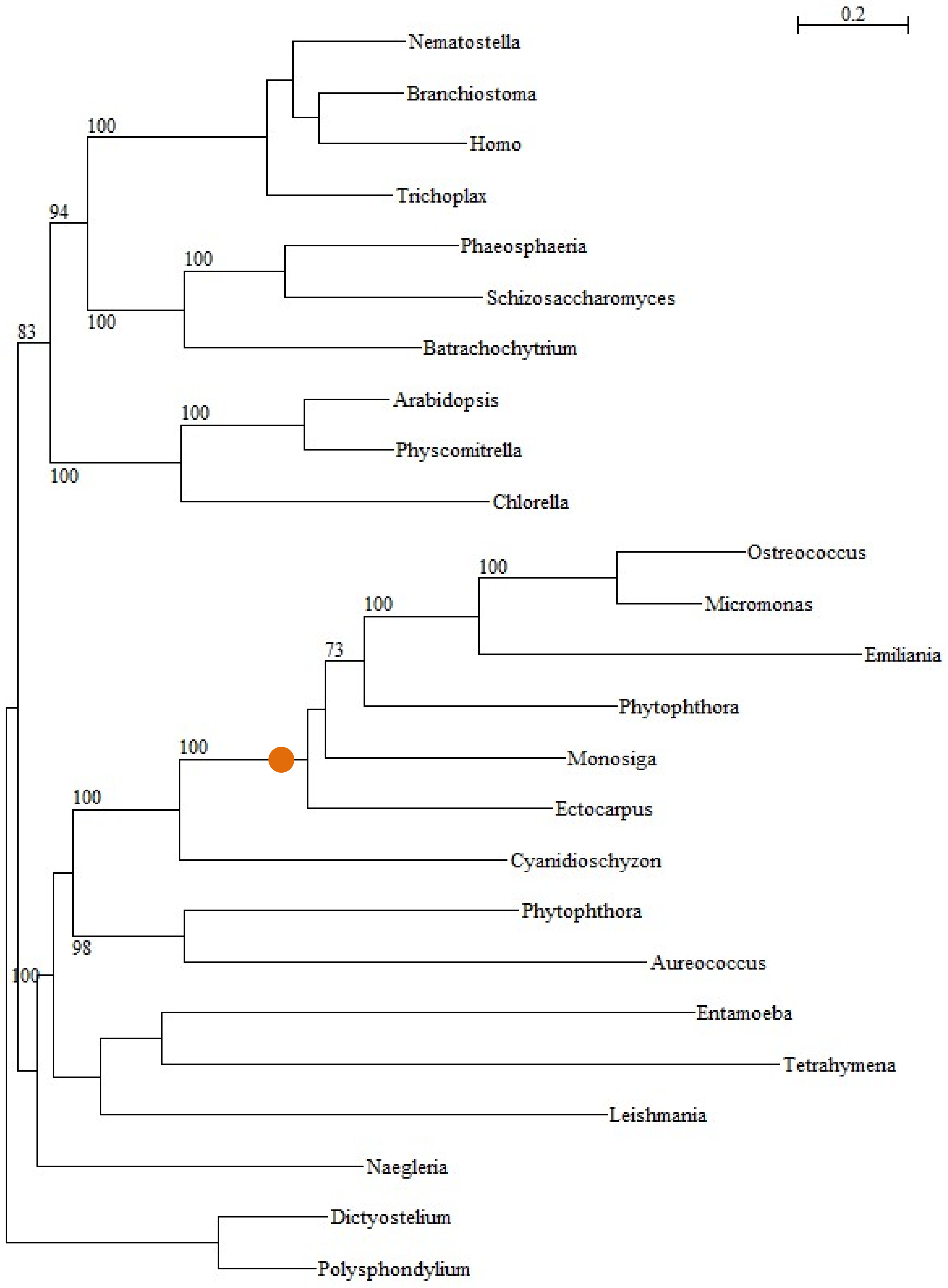

Figure S88. Phylogeny of p-type ATPase (GI No. 167534304).

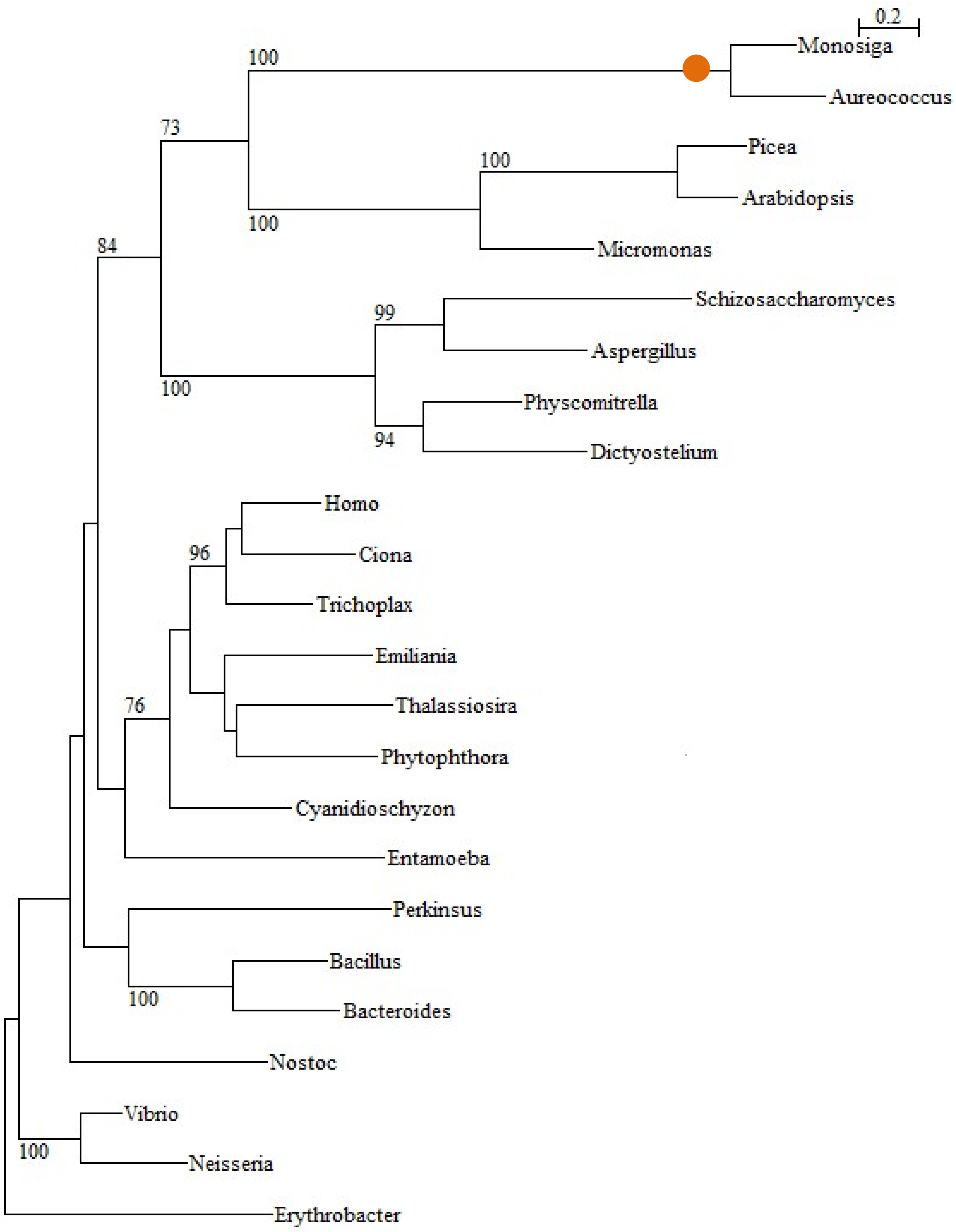

Figure S89. Phylogeny of galactokinase (GI No. 167535085).

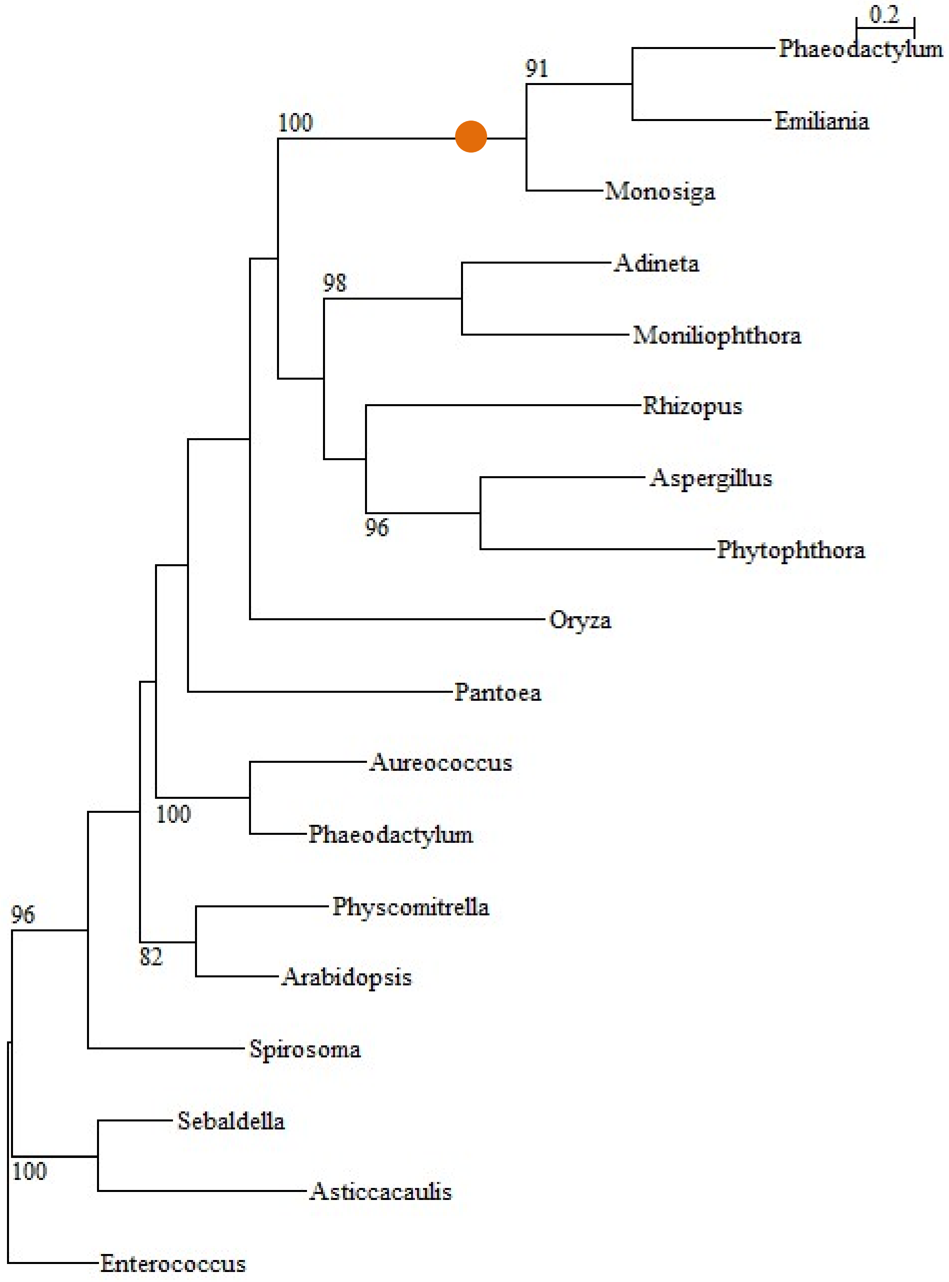

Figure S90. Phylogeny of one hypothetical protein (GI No. 167535947).

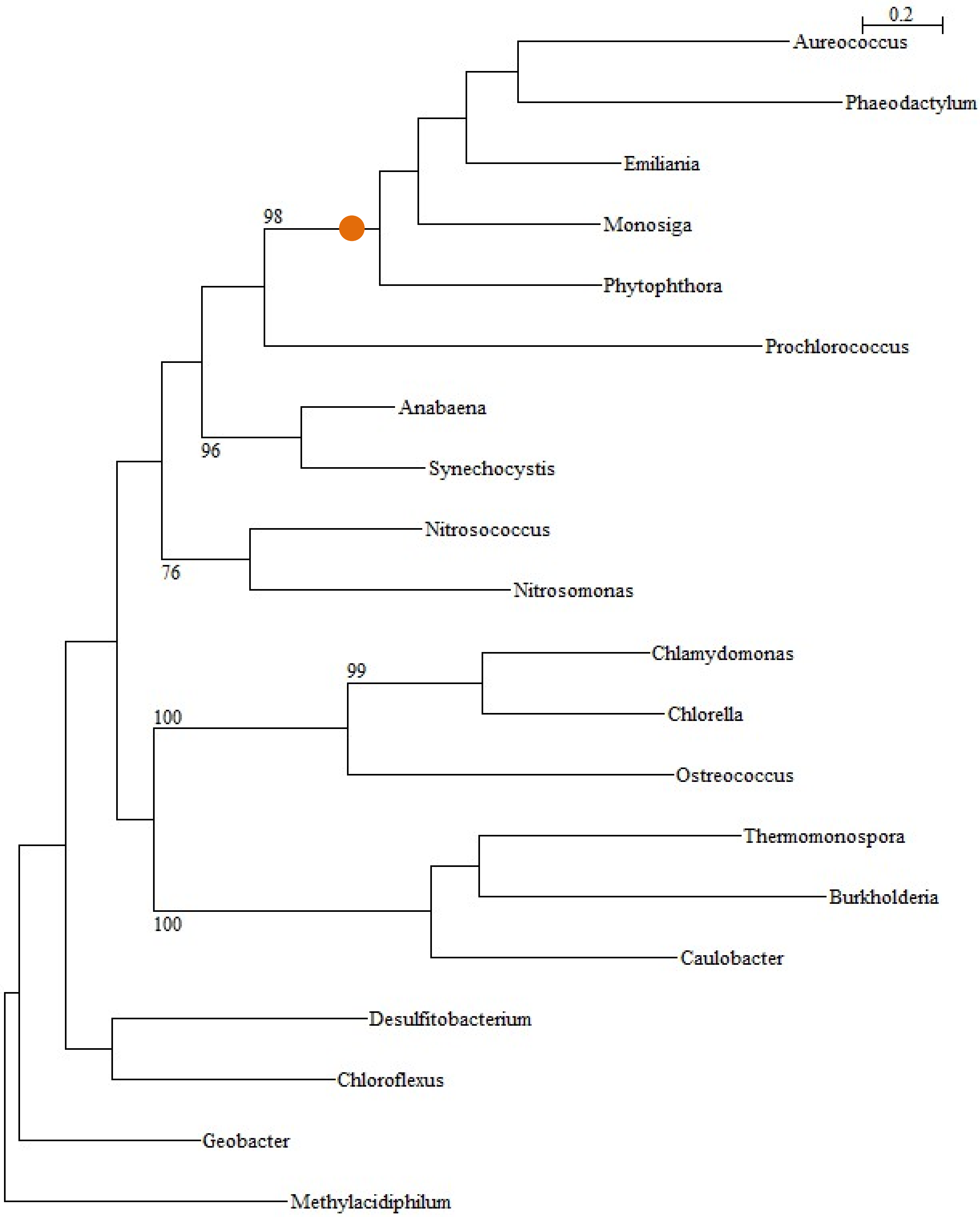

Figure S91. Phylogeny of glucokinase (GI No. 167537338).

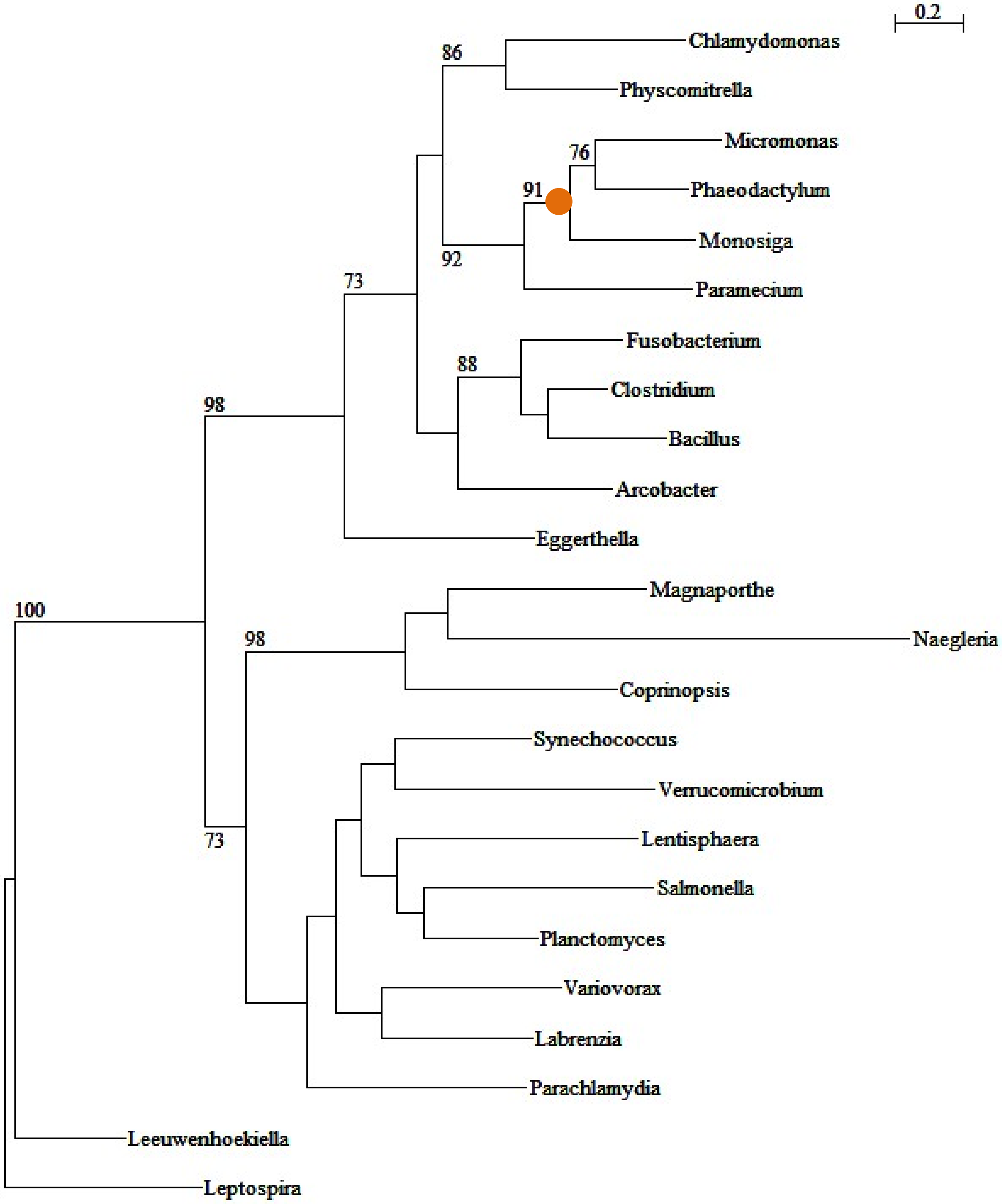

Figure S92. Phylogeny of pirin-related protein (GI No. 167538121).

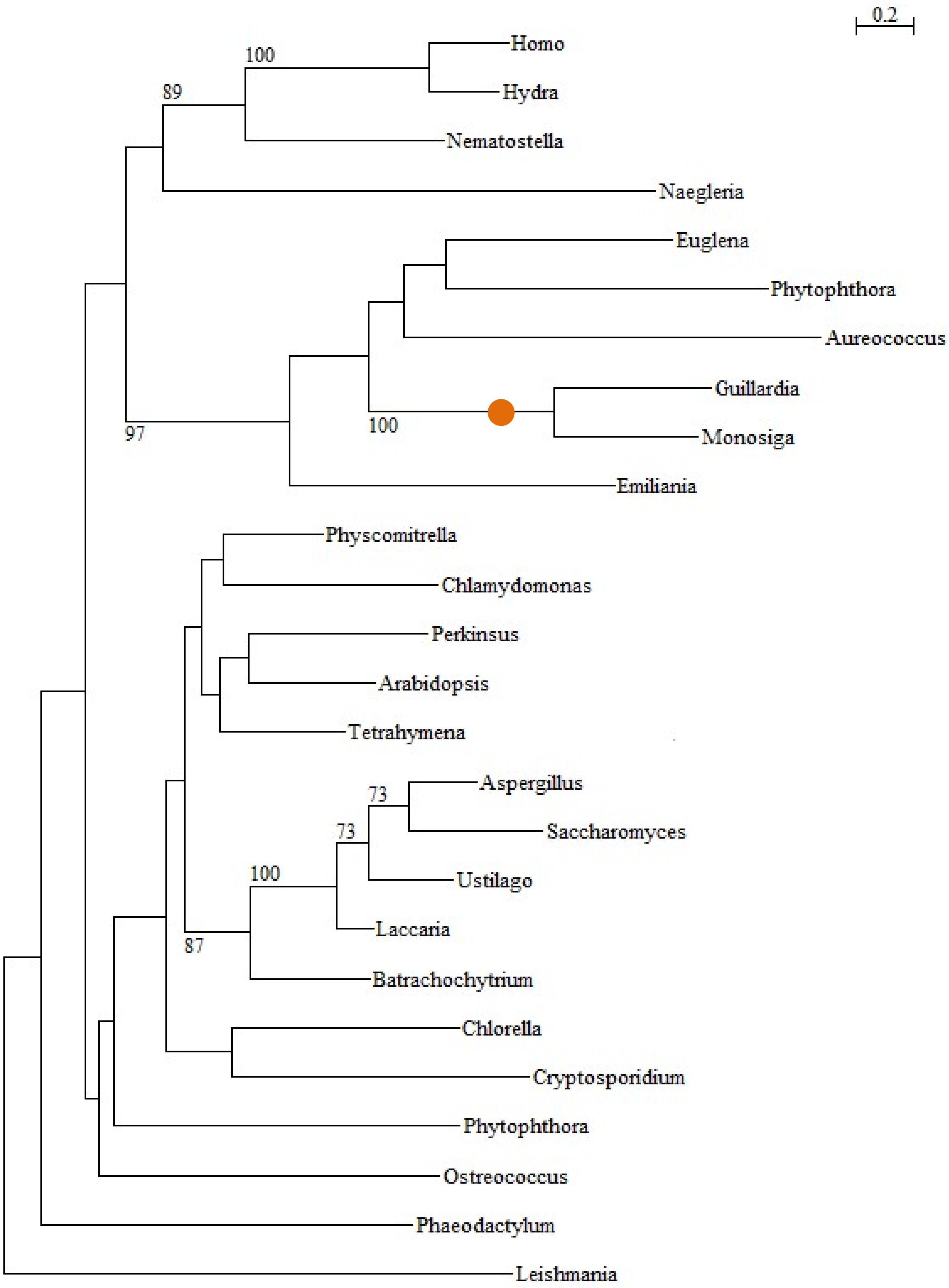

Figure S93. Phylogeny of one hypothetical protein (GI No. 167538169).

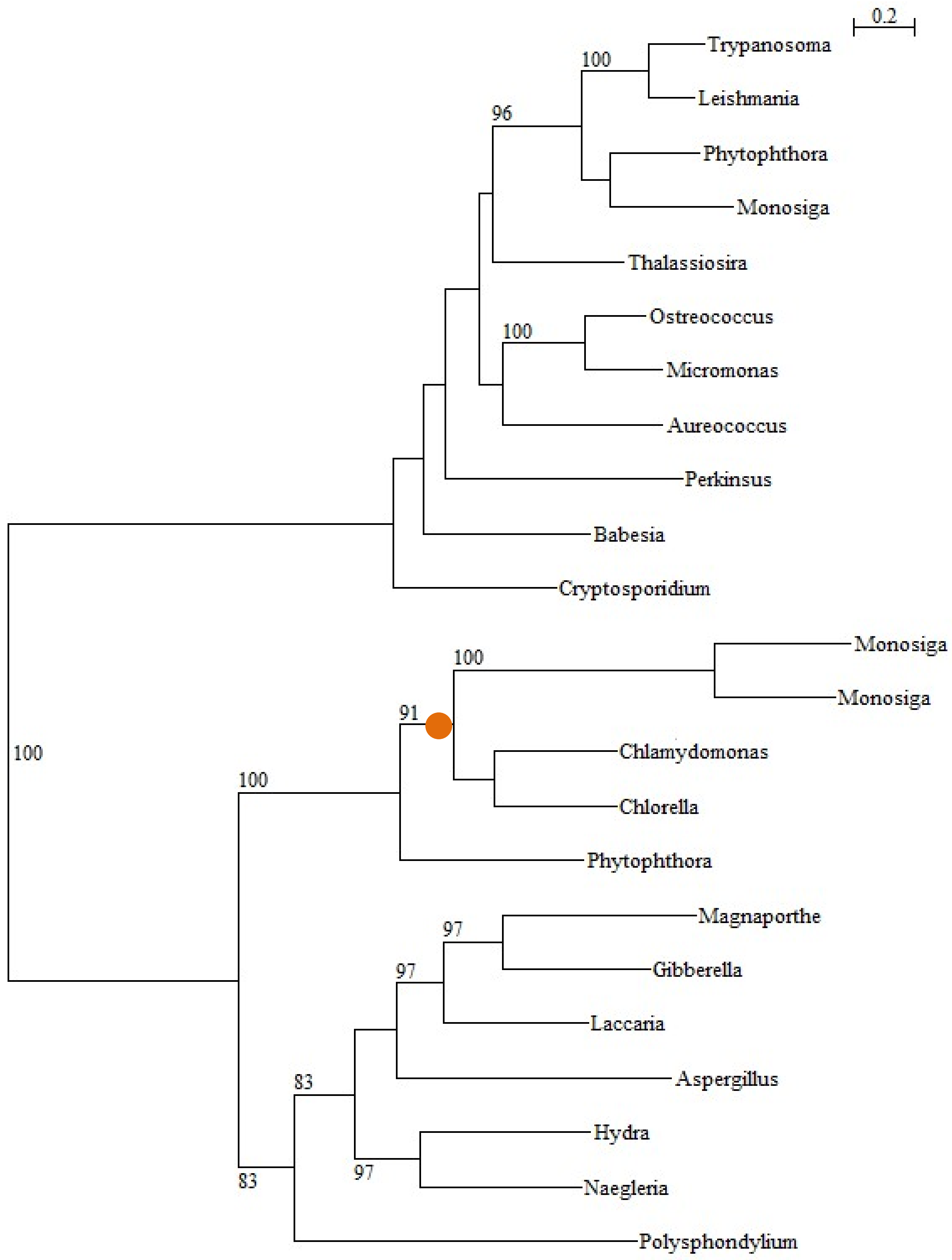

Figure S94. Phylogeny of one hypothetical protein (Gi No. 167515382 and 167538595).

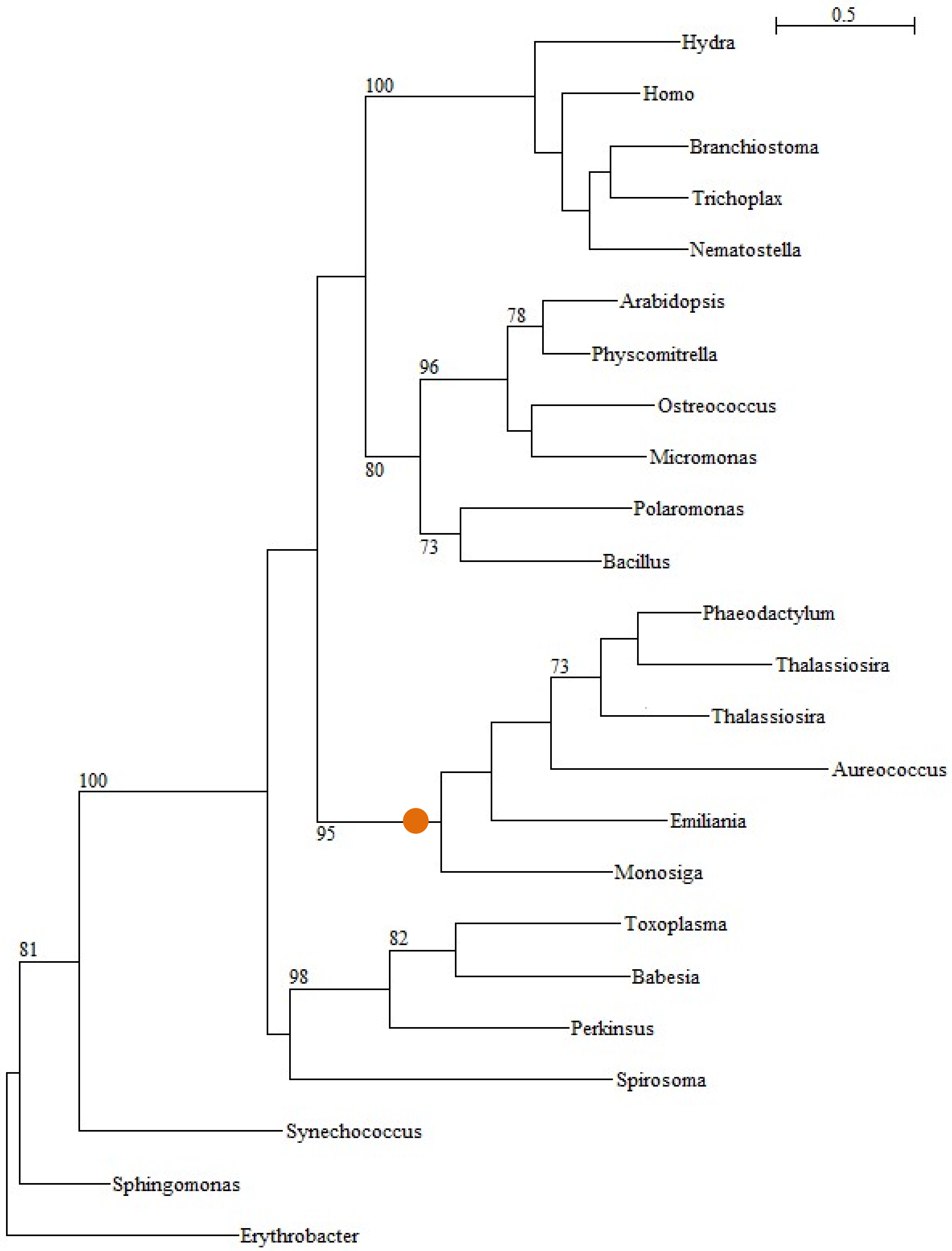

Figure S95. Phylogeny of prolyl 4-hydroxylase (GI No. 167524906).

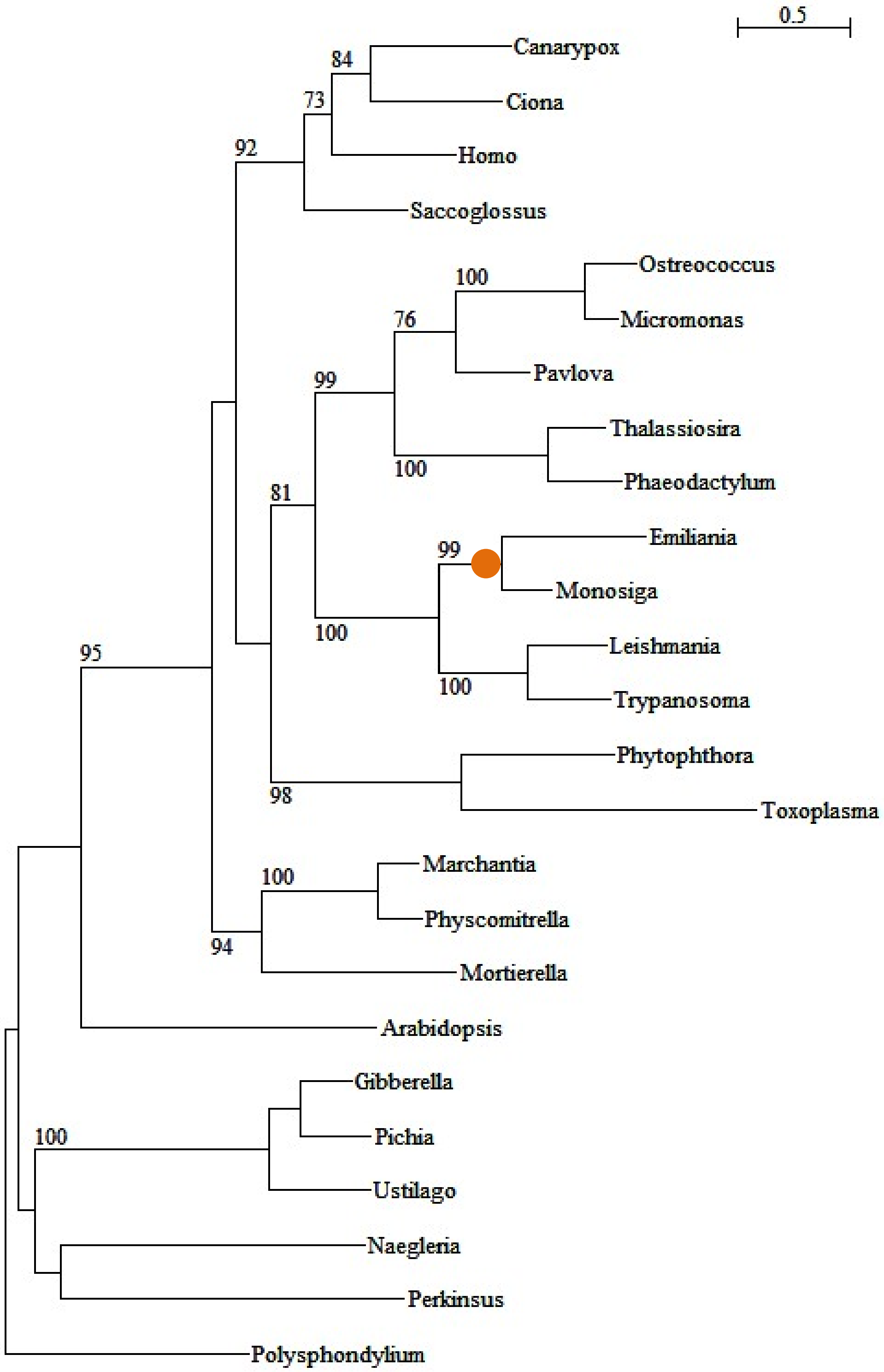

Figure S96. Phylogeny of fatty acid elongase (GI No. 167526168).

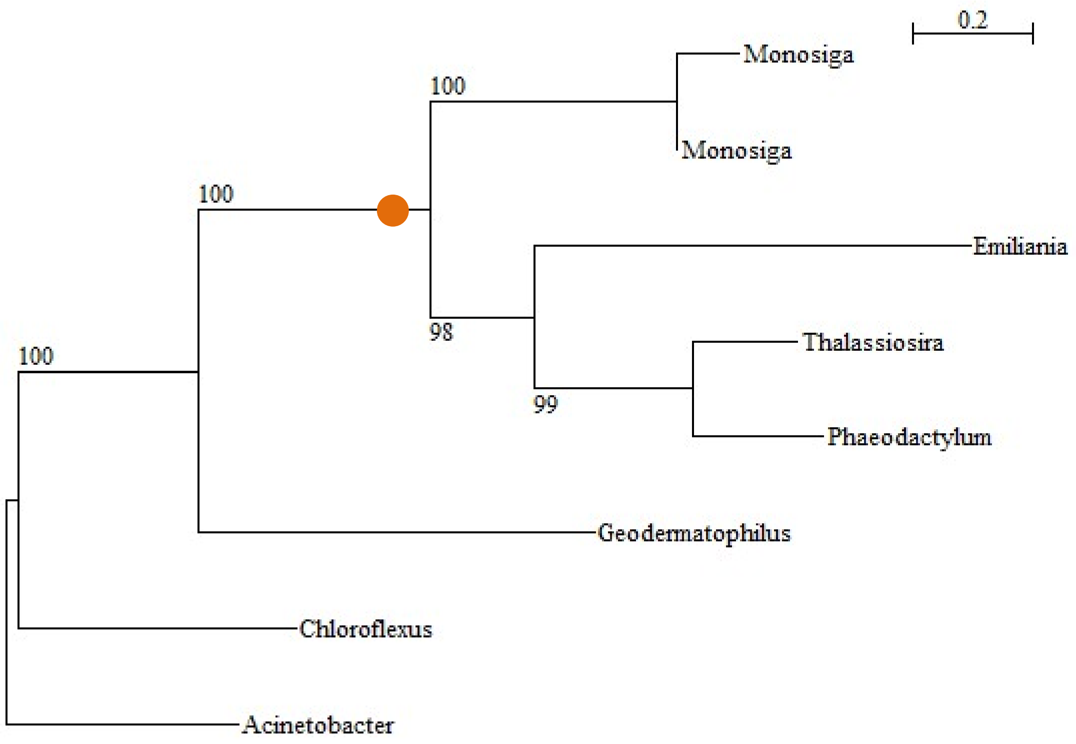

Figure S97. Phylogeny of one hypothetical protein (GI No. 167526275 and 167538724). Sequences of *Monosiga* and Chromists form a clade, and their close relationship is also supported by the shared characters in multiple sequence alignment.

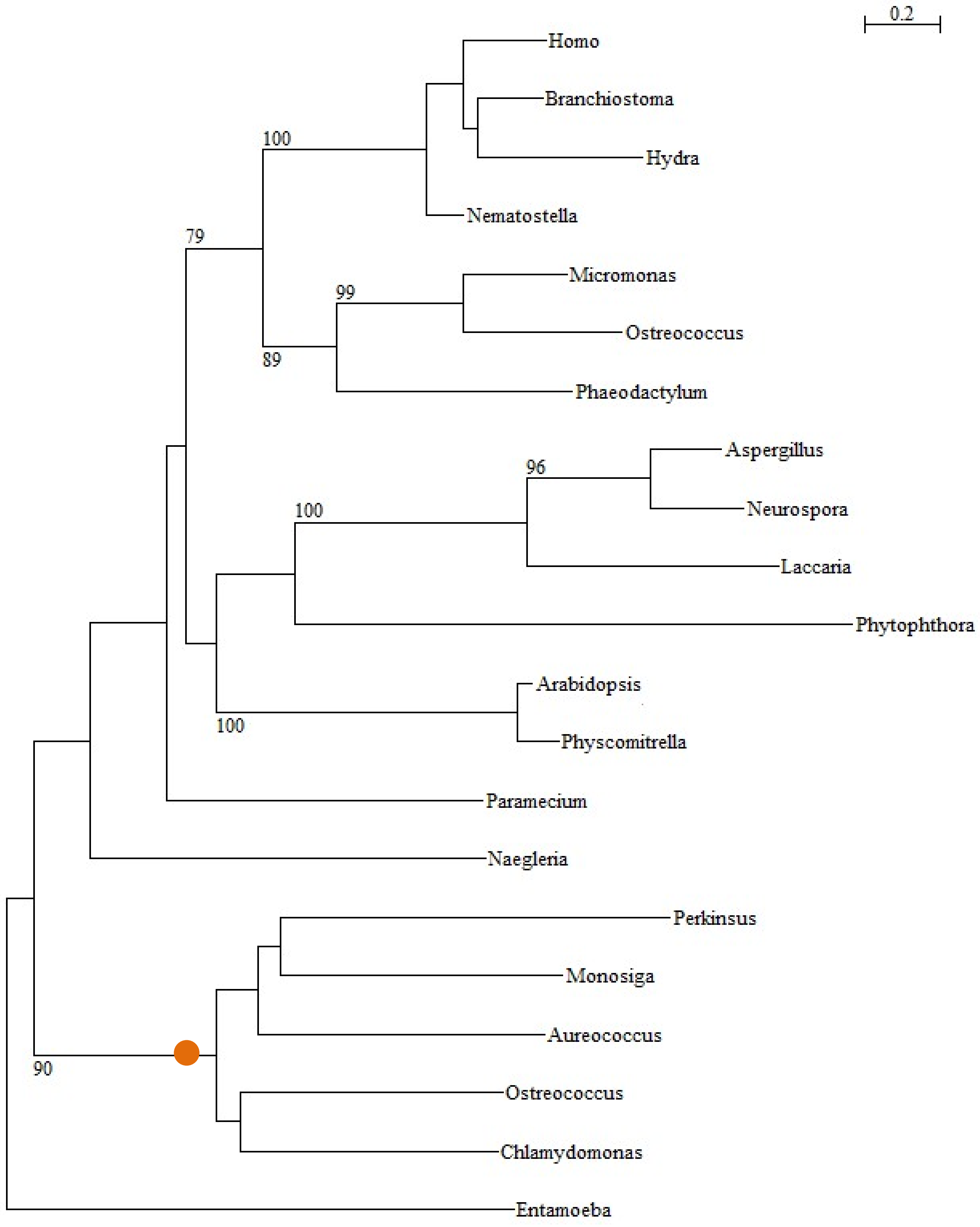

Figure S98. Phylogeny of cysteine protease (GI No. 167522705).

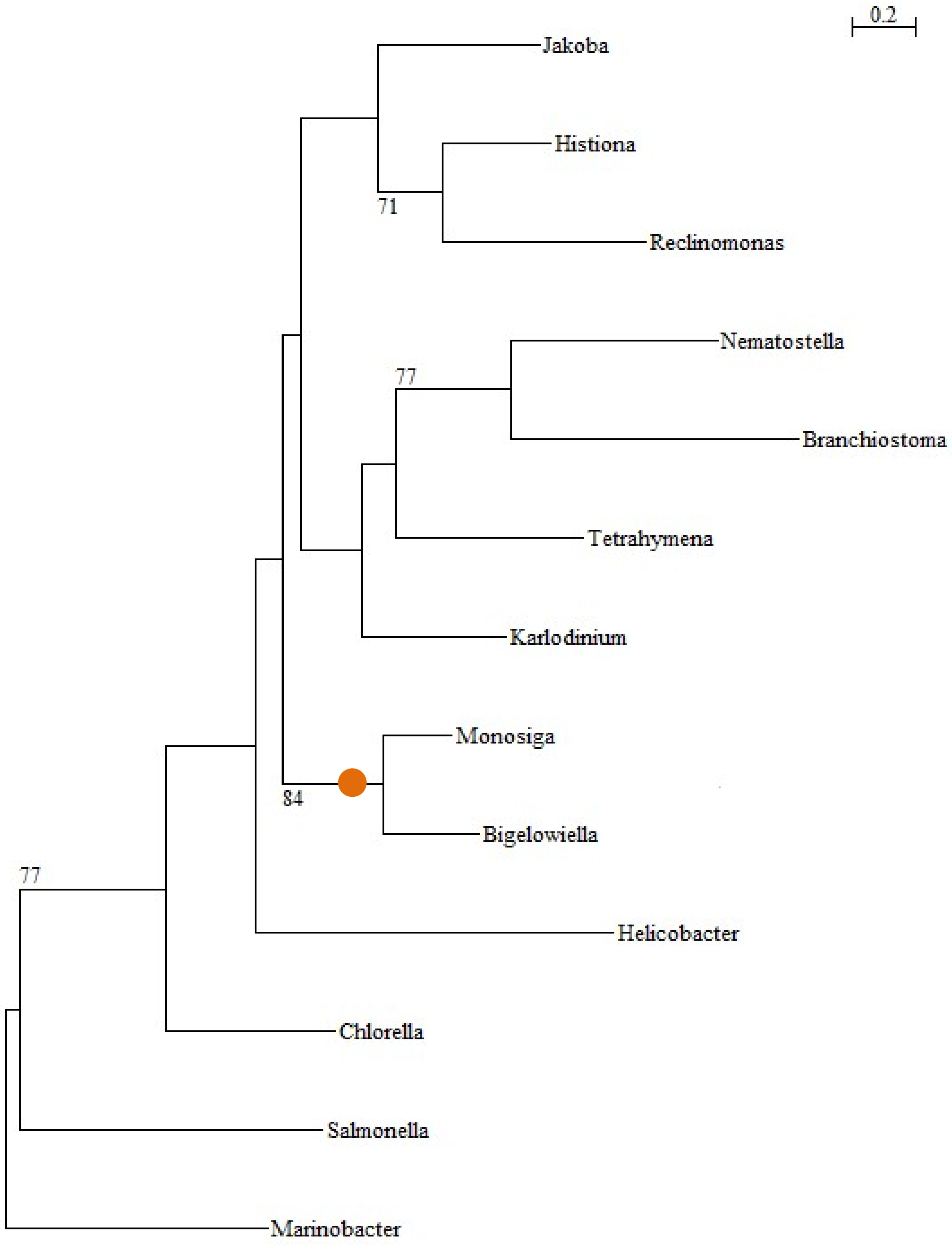

Figure S99. Phylogeny of one hypothetical protein (GI No. 167534439).

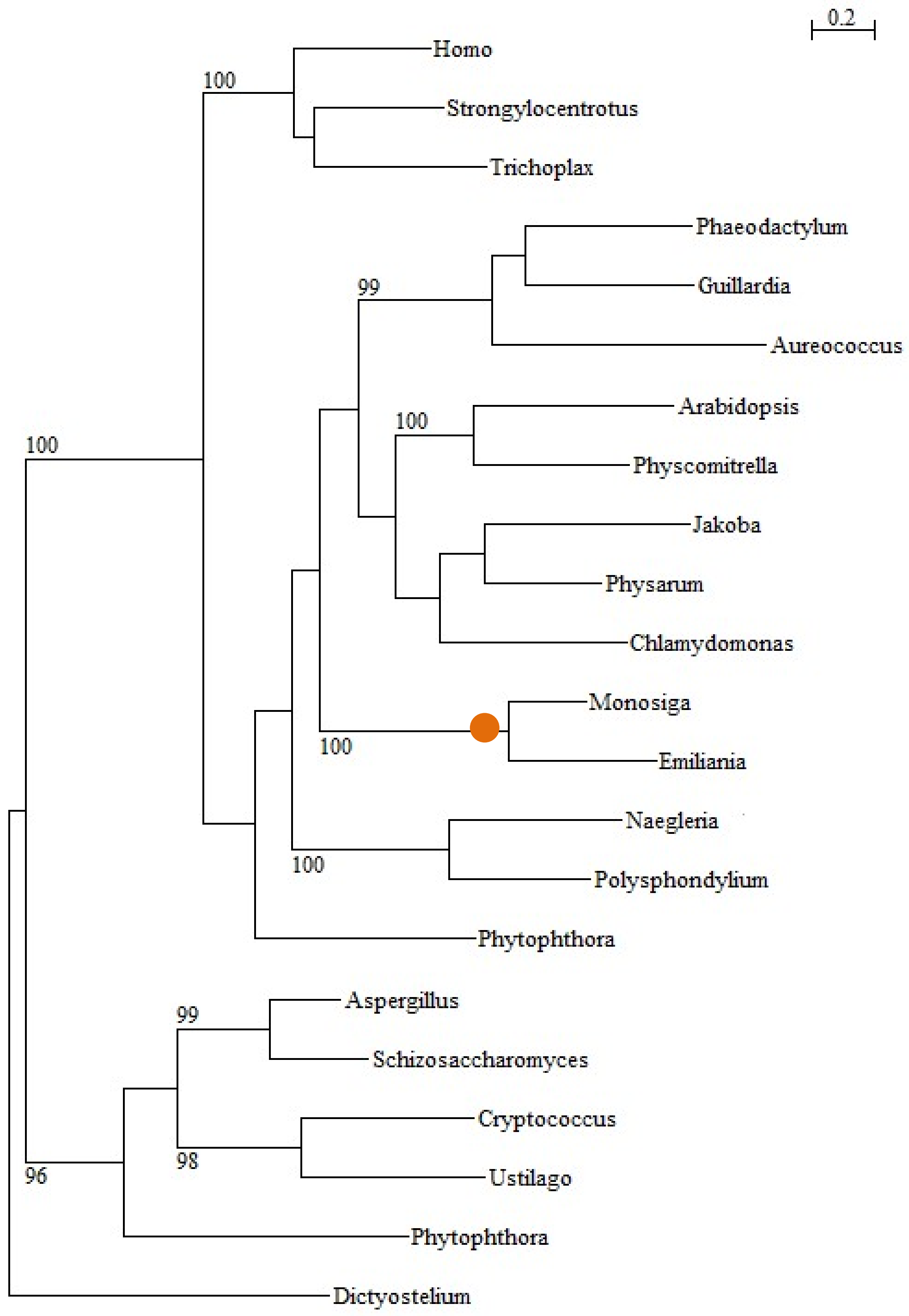

Figure S100. Phylogeny of serine carboxypeptidase (GI No. 167534625).

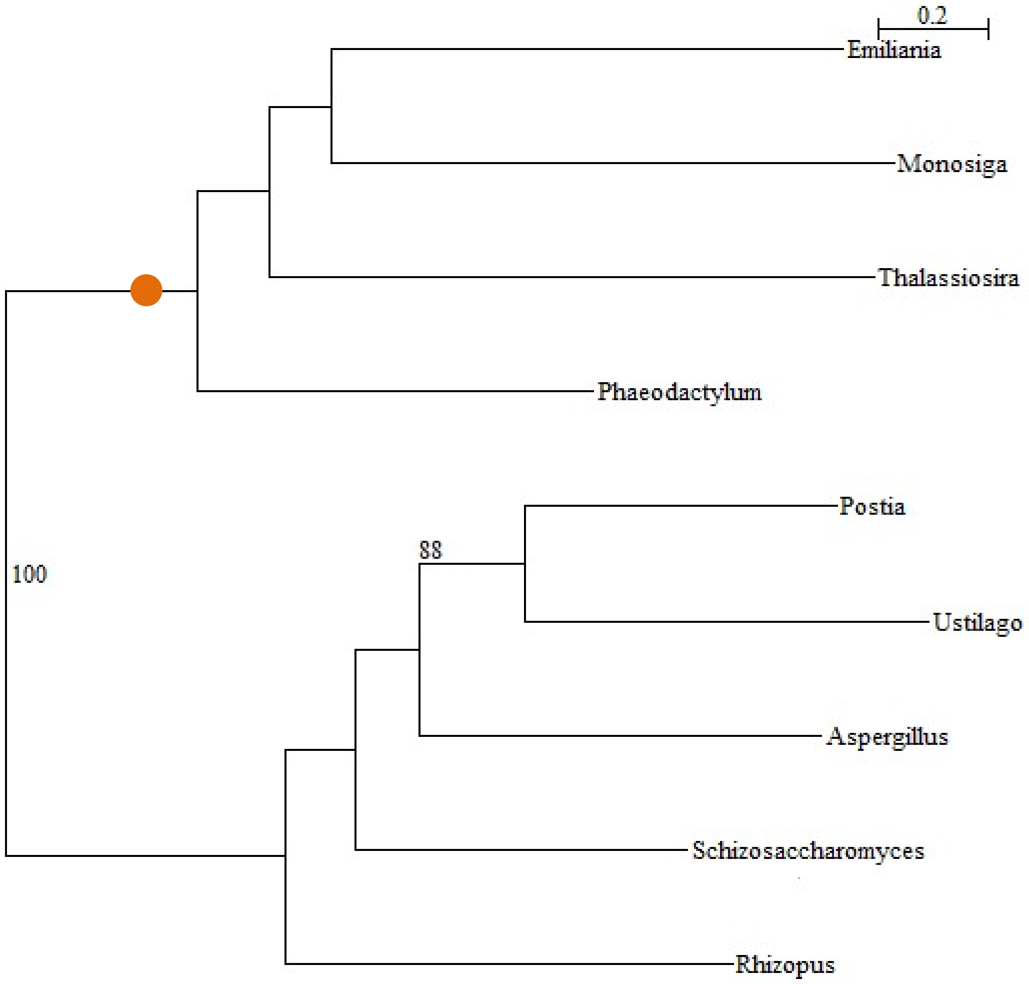

Figure S101. Phylogeny of Ser/Thr protein phosphatase (GI No. 167534824).

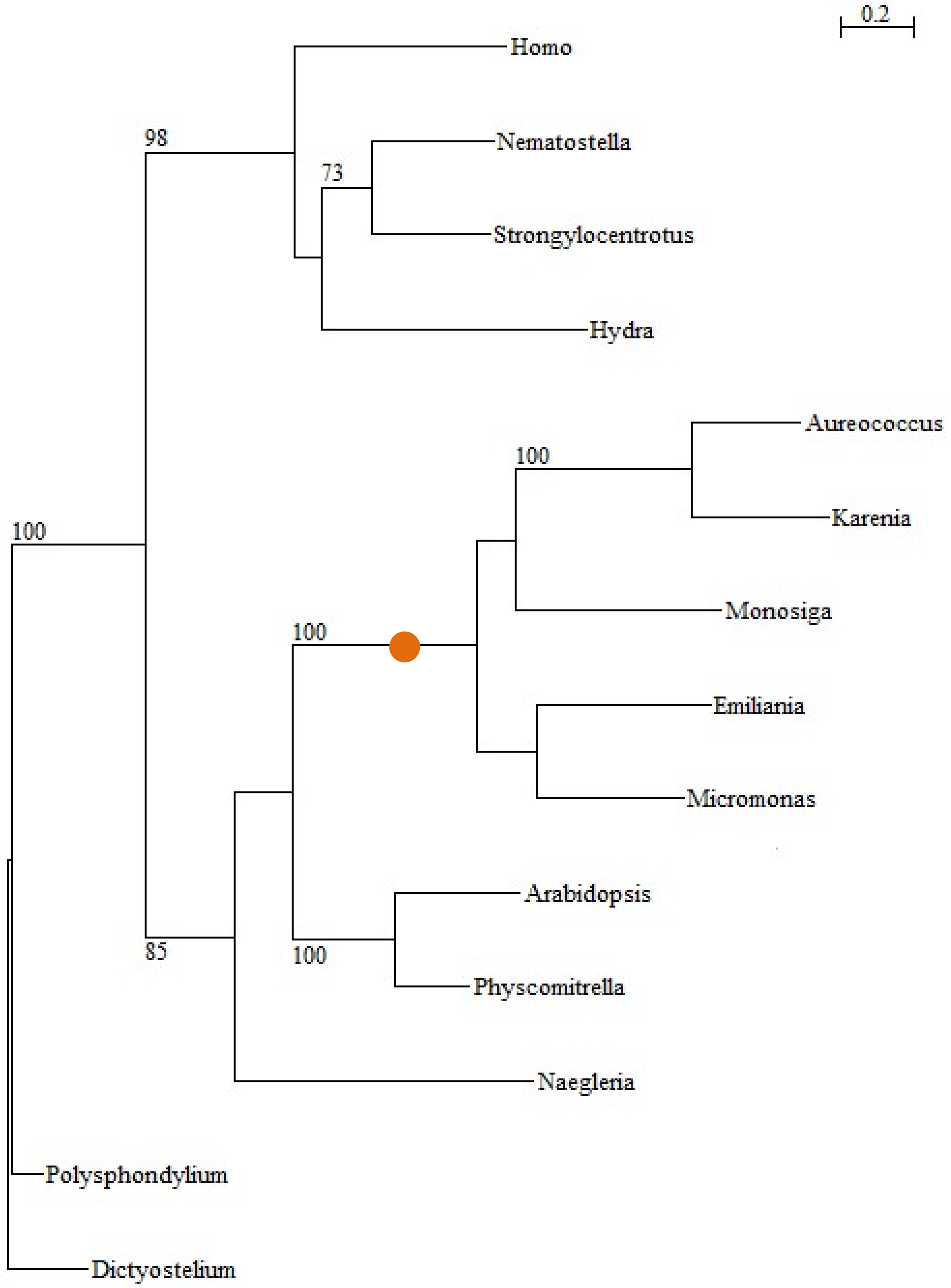

Figure S102. Phylogeny of 2OG-Fe (II) oxygenase (GI No. 167535670).

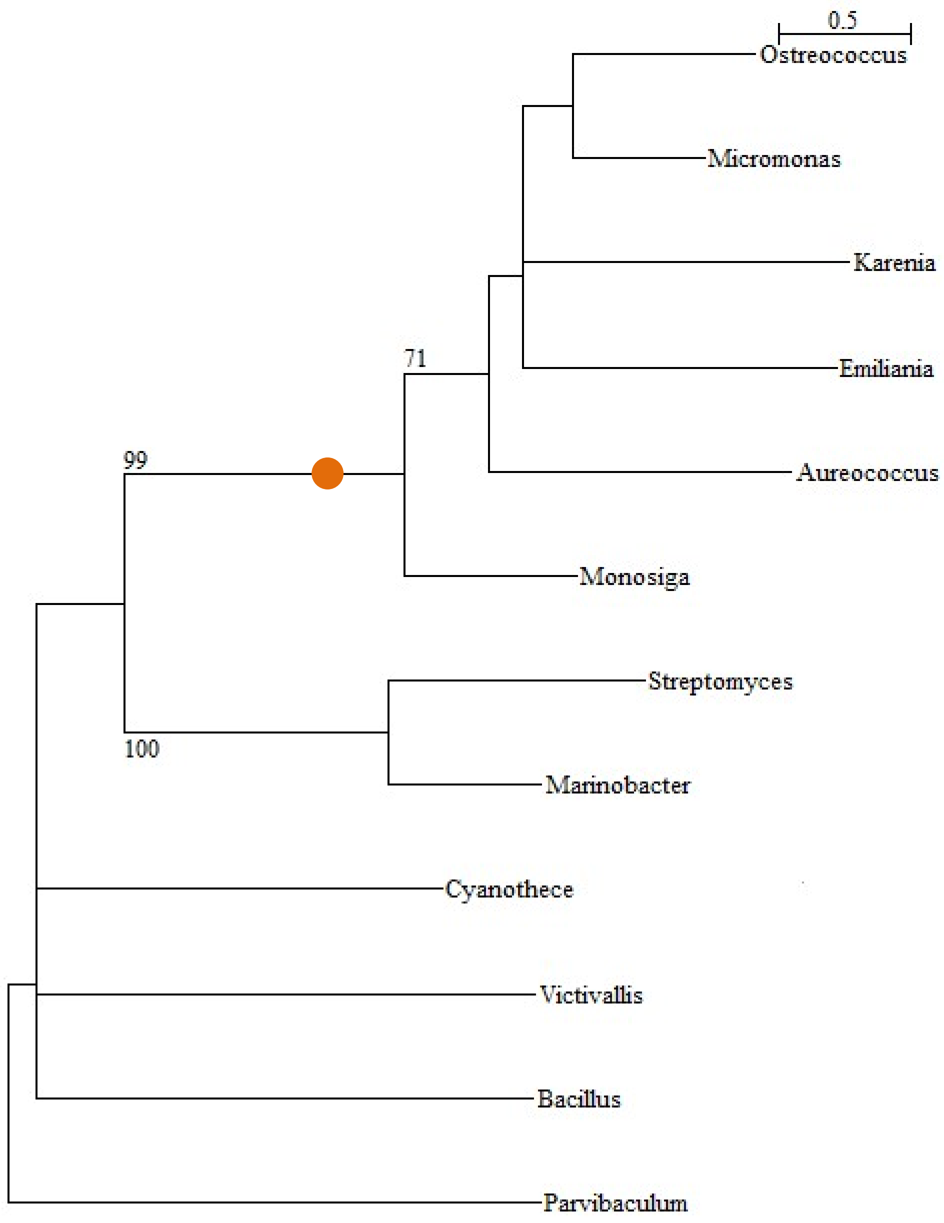

Figure S103. Phylogeny of one hypothetical protein (GI No. 167522493).

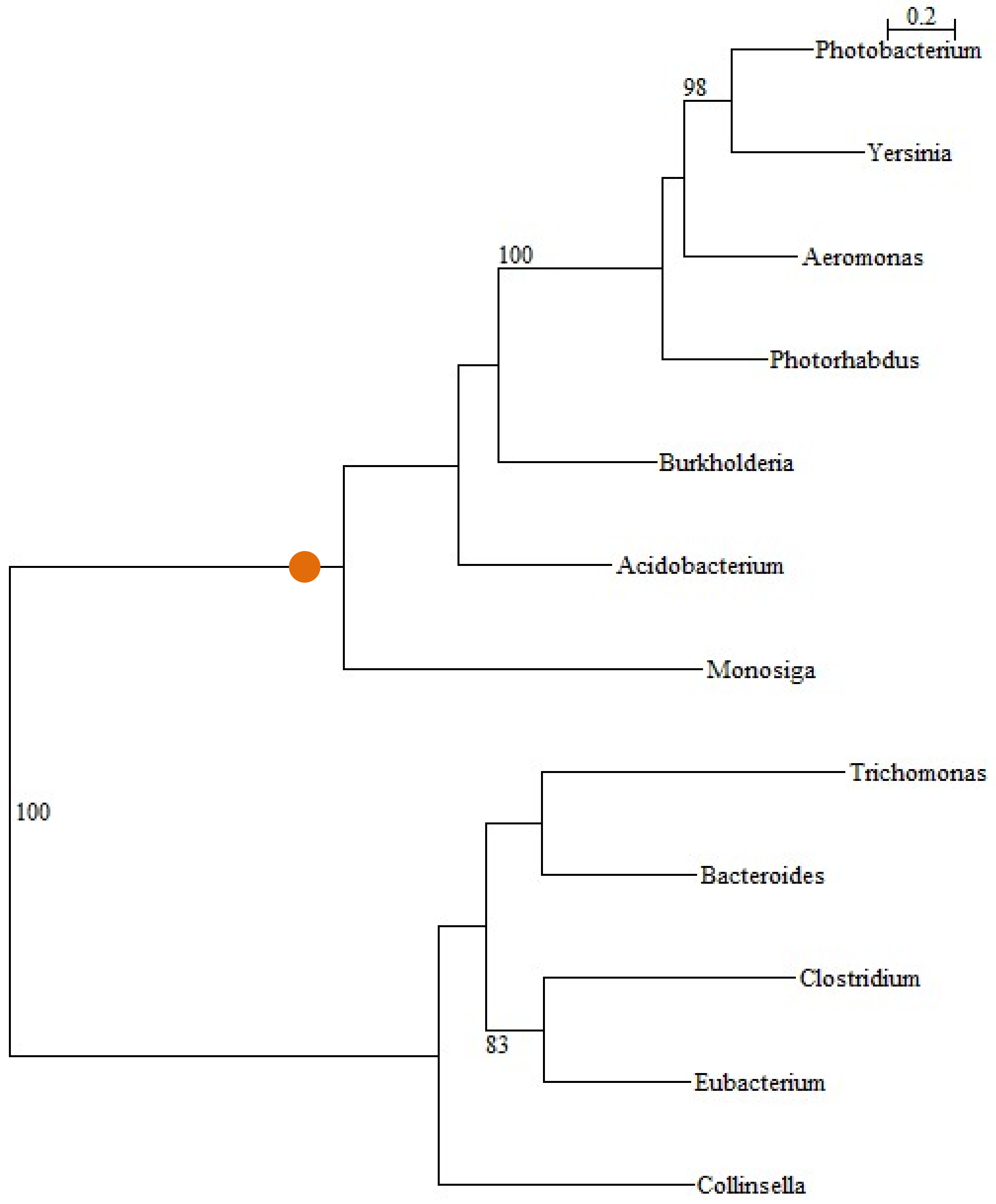

Figure S104. Phylogeny of enterotoxin (GI No. 167537376).

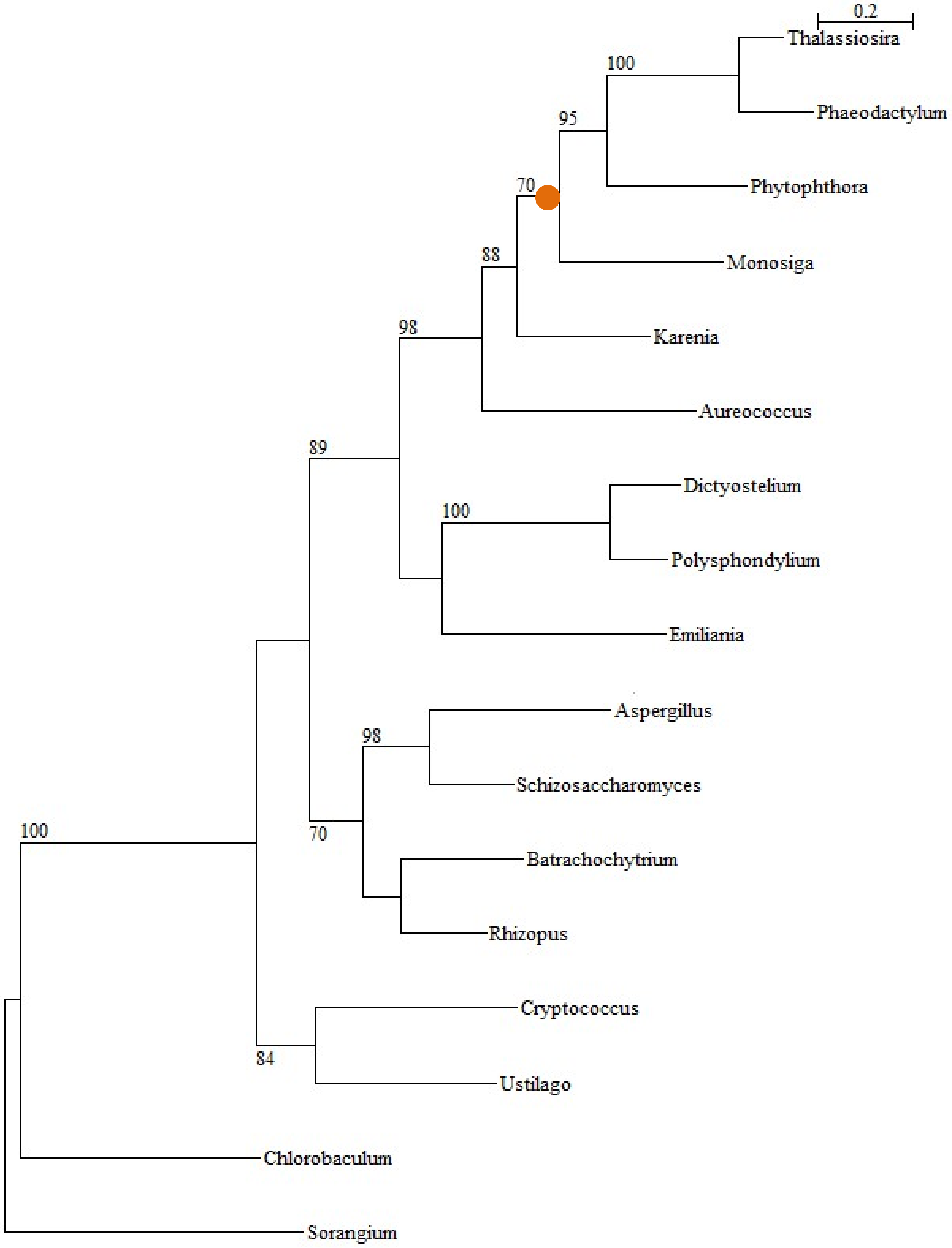

Figure S105. Phylogeny of methylenetetrahydrofolate dehydrogenase (GI No. 167523990).

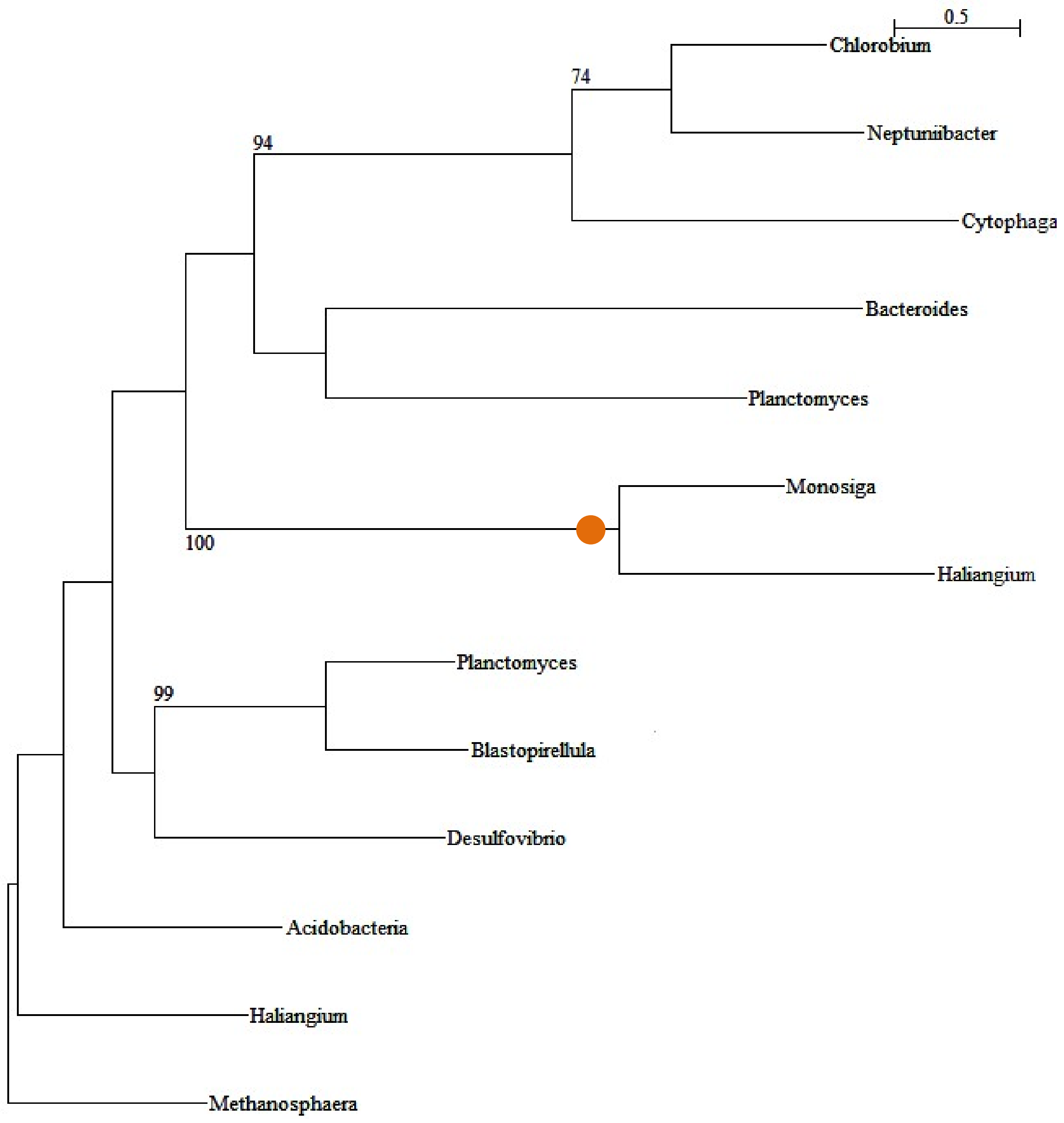

Figure S106. Phylogeny of glycosyltransferase (GI No. 167522307).

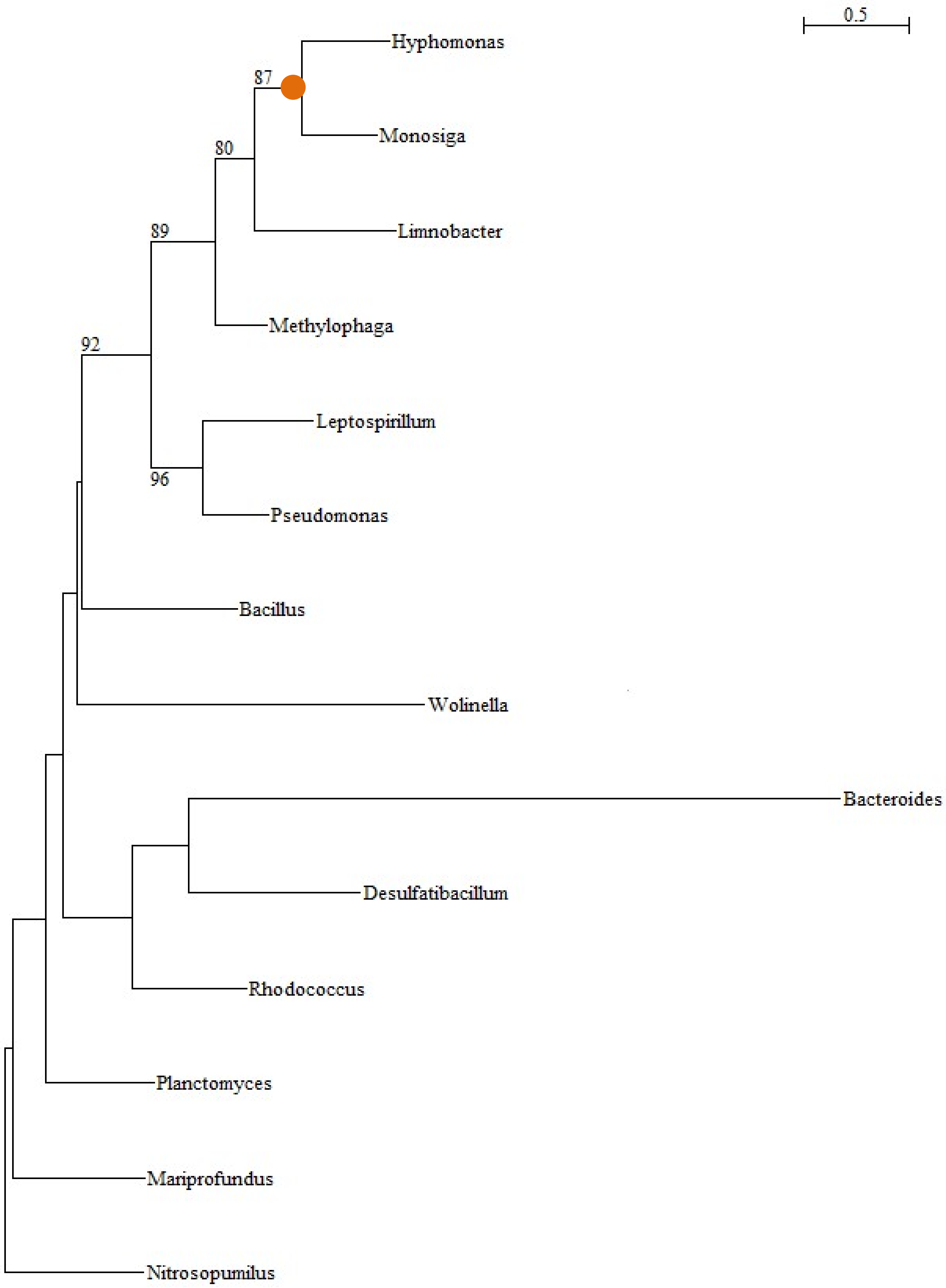

Figure S107. Phylogeny of L-2,4-diaminobutyric acid acetyltransferase (GI No. 167517491).

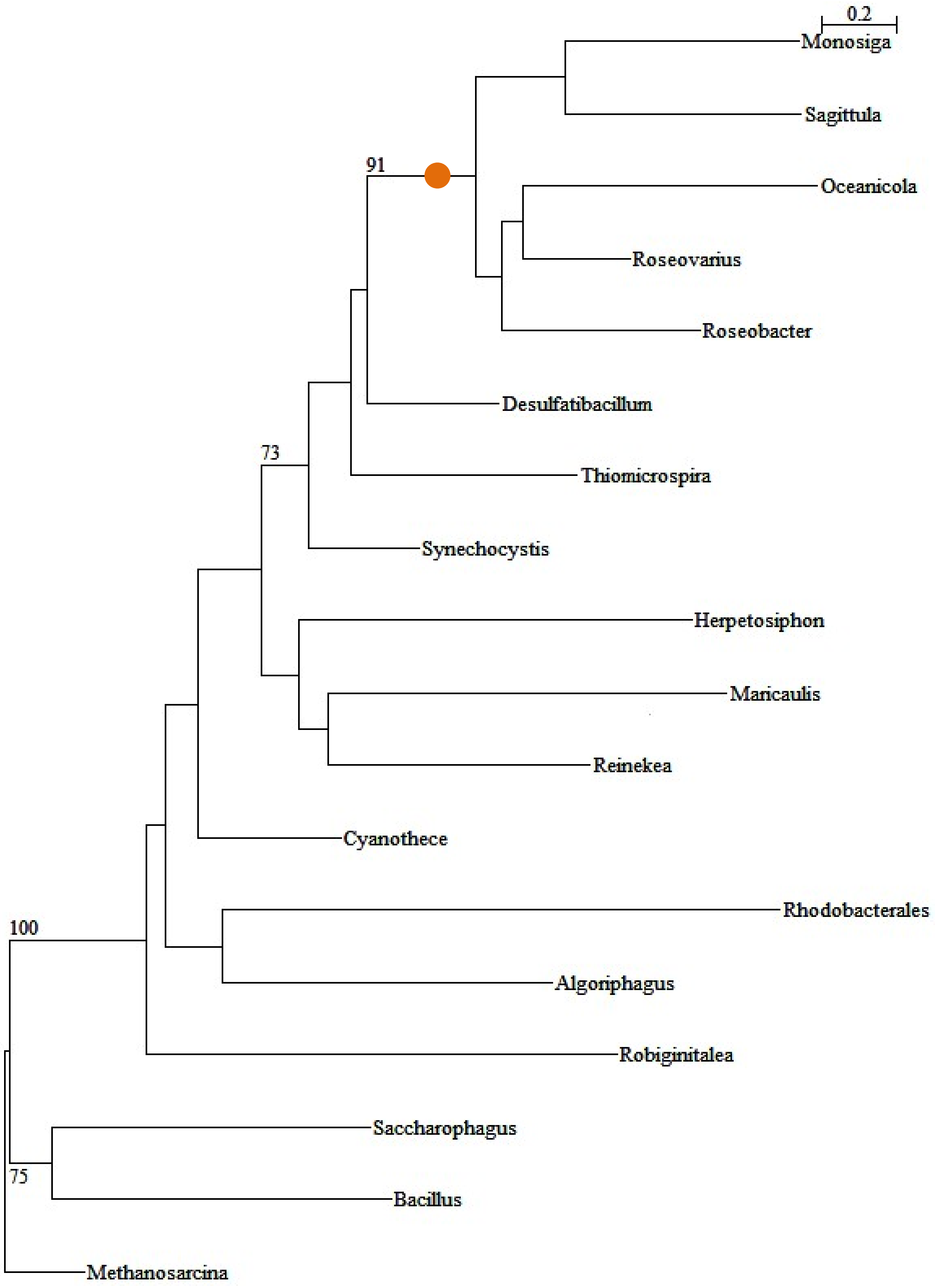

Figure S108. Phylogeny of one hypothetical protein (GI No. 167534872).

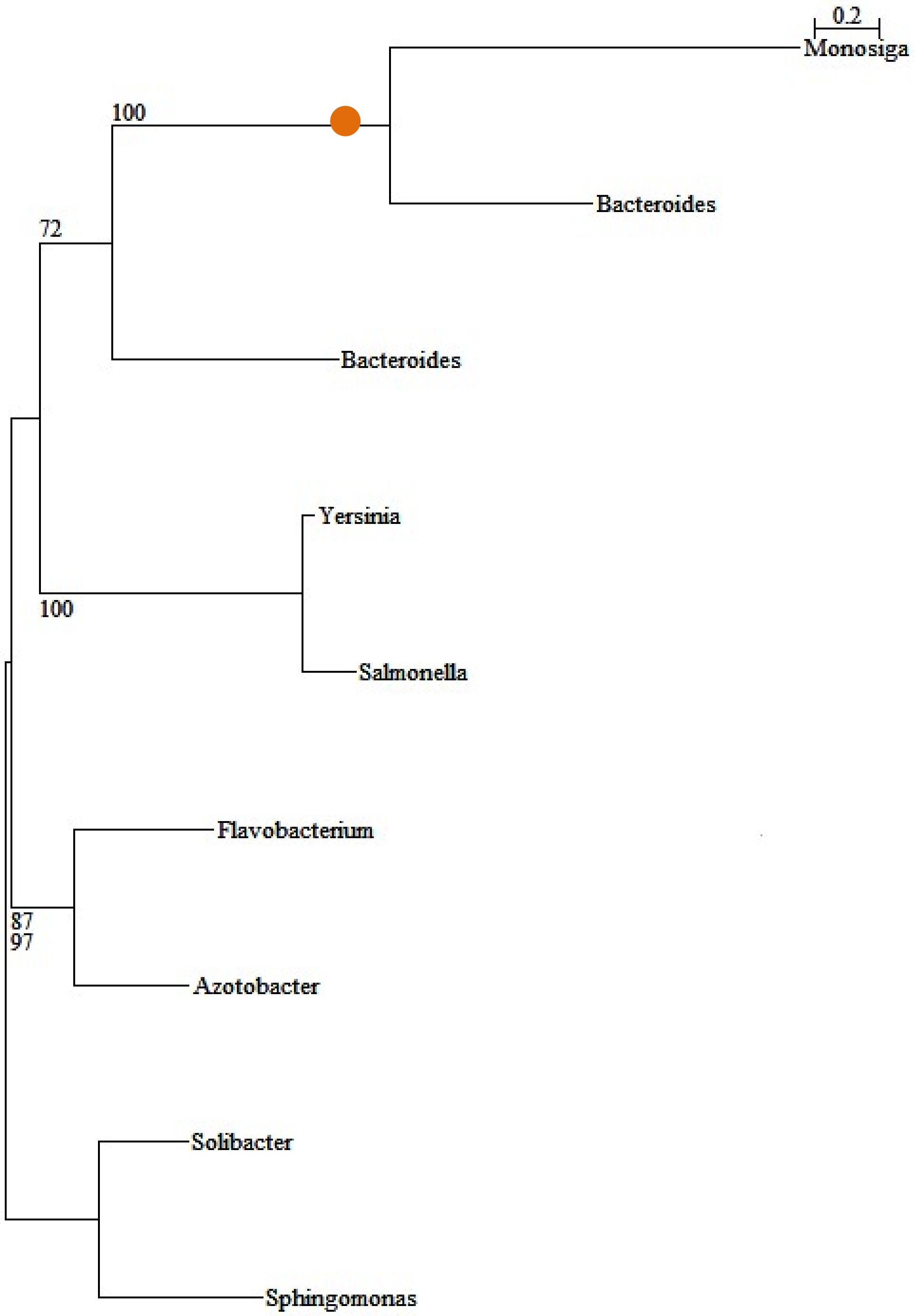

Figure S109. Phylogeny of rhamnose-proton symporter (GI No. 167515428).
